# The Science of Polymer Chemical Recycling Catalysis: Uncovering Kinetic and Thermodynamic Linear Free Energy Relationships

**DOI:** 10.1021/jacs.5c04603

**Published:** 2025-06-23

**Authors:** Thomas M. McGuire, David Ning, Antoine Buchard, Charlotte K. Williams

**Affiliations:** † Department of Chemistry, Chemistry Research Laboratory, 6396 University of Oxford, 12 Mansfield Road, Oxford OX1 3TA, U.K.; ‡ Department of Chemistry, Green Chemistry Centre of Excellence, 8748 University of York, York YO10 5DD, U.K.

## Abstract

Polymer recycling must accelerate to limit ever-growing wastes and carbon dioxide emissions. Polymer chemical recycling to monomer enables multiple closed recycling loops, tackling the material and property losses endemic to mechanical recycling. Polyesters and polycarbonates, derived from 6- and 7-membered heterocycles, are leading sustainable materials produced by equilibrium polymerizations that can be reversed for selective and efficient chemical recycling to monomer. A systematic understanding of the depolymerization kinetic and thermodynamic structure-recycling relationships is needed; in particular, studies should focus on the low-energy and minimal-chemical additive conditions required for any larger-scale processes. Here, the depolymerization kinetic parameters, including rate constants and transition state energy barriers, are measured for a systematic series of leading aliphatic polyesters and polycarbonates. These recycling experiments are conducted under common conditions using neat polymer melts, at temperatures from 90 to 190 °C and with low loadings (1:100–1000) of a fast, selective, and commercial zinc­(II)­bis­(2-ethylhexanoate) catalyst. The systematic kinetic measurements quantify the influences of different repeat units, substituents, and end-group chemistries on the recycling process. All the polymers conform to a linear free energy relationship between the depolymerization kinetic (Δ*G*_d_^⧧^) and thermodynamic (Δ*G*_d_) energy differences. The discovery of recycling catalysis linear free energy relationships allows for the rational selection of the lowest temperature (and energy) recycling conditions, operable using neat polymers, to deliver both high monomer conversions and rates. The quantified structure-recycling relationships are also used to efficiently and selectively separate mixtures of structurally similar polymers by their quantitative chemical recycling into pure monomers.

## Graphical abstract

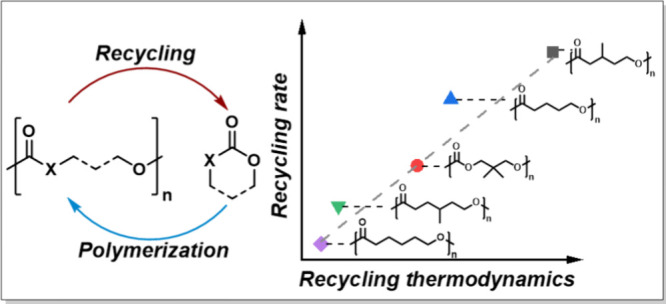

## Introduction

Improving polymer sustainability is a very important scientific challenge with recent studies highlighting the combined threats to us all of mismanaged plastic wastes and the ever-growing carbon dioxide emissions across polymer lifecycles.^1–4^ Since the majority of these CO_2_ emissions arise during raw material and polymer manufacturing life-cycle stages, one very important waste mitigation strategy is to implement fast, efficient and selective closed-loop polymer recycling. Polymer chemical recycling to monomer is particularly valuable since it tackles the material losses and property degradation which challenge mechanical recycling.^5,6^ Efficient chemical recycling to monomer could help to limit virgin monomer production, preserve the material embedded energy and cut greenhouse gas emissions.^7,8^

Selecting both the right polymer chemistries and recycling conditions for chemical recycling is currently somewhat empirical and driven by polymerization thermodynamic data. The chemical recycling conditions are identified by reversing those used for polymerizations so as to favor chain depolymerization.^9^ Thermodynamically, polymerization is almost always driven by an enthalpy gain (Δ*H*_p_ < 0) and is entropically disfavored (Δ*S*_p_ < 0).^10^ Understanding the reaction thermodynamics helps explain why current aliphatic hydrocarbon polymers (polyolefins) are poorly suited to chemical recycling to monomer; such chemistries have very high ceiling temperatures, i.e. the minimum temperature at which depolymerization is thermodynamically feasible.^11,12^ For example, recent reports of polyethylene thermolysis required temperatures >500 °C, and resulted in poor selectivity yielding just 25 wt % ethene, with the remaining products being other hydrocarbons, gases, wax and char.^13^ In comparison, aliphatic polyesters and polycarbonates are already commercially manufactured using equilibrium polymerizations and, hence, depolymerization should be thermodynamically feasible at much lower temperatures, typically from 100 to 350 °C.^14^ Polymers made from 6 and 7-membered monocyclic esters/carbonates are particularly interesting since several monomers are already commercialized and biobased, their properties match those of conventional hydrocarbon polymers and their thermodynamics should favor chemical recycling to cyclic monomers.^15–17^ Their polymerization/depolymerization equilibria are understood to depend empirically on the monomer ring size. Generally, 6-membered monocyclic rings have more favorable depolymerization equilibria than analogous 7-membered structures.^10^ In such heterocycles, monomer-substituents and their positions also impact the polymer/monomer thermodynamics, generally more substituents stabilize the monomers (i.e., promote depolymerization).^18^ Chen and co-workers have been especially interested in exploiting such polymerization/depolymerization equilibria to produce high-performance, chemically recyclable polyesters that have equivalent or better properties than current plastics such as polyethylene and polypropylene.^19^ They showed that poly­(δ-valerolactone) (PVL, **PE-6a**, Figure 1),^20^ and its substituted derivatives,^21,22^ have tensile mechanical properties equivalent to both high and low density polyethylene grades but in contrast these polyesters can be chemically recycled to monomer (recycling: 90–150 °C, ZnCl_2_ catalyst). Cai, Zhu and co-workers developed poly­(ε-caprolactones) (PCL, **PE-7a**) that exhibit toughness and ductility comparable to isotactic polypropylene but are chemically recyclable (recycling: 0.02 M PCL, toluene, 140 °C, 2 mol % Zn catalyst).^23^ Hillmyer and co-workers pioneered block polyester thermoplastic elastomers showing thermal-mechanical properties equivalent to those of polyolefin or polystyrene elastomers.^24^ The same team also showed related systems can act as degradable adhesives.^25^ Coates and Chen independently reported upon substituted γ-butyrolactones which show mechanical toughness comparable to polyethylene and polypropylene but with improved recyclability (recycling: 200–250 °C, 1–5 wt % catalyst).^26,27^ Aliphatic polycarbonates also show mechanical properties similar to polyethylene and polystyrene,^28,29^ and, in block polymers, are as tough and resilient as vulcanized rubber-, urethane- and olefin-elastomers with wide operating temperatures.^30^ In addition to these academic studies, the commercial production of these polyesters and carbonates continues to accelerate and their range of products have moved beyond packaging into other sectors. As the above literature examples demonstrate, despite the significant promise of these materials the recycling investigations tend to be conducted under different conditions. This prevents easy comparisons and limits any quantitative understanding of structure-recycling performance relationships. This study aims to systematically investigate and compare the catalyzed chemical recycling of these leading aliphatic polyesters and polycarbonates.

**Figure 1:**
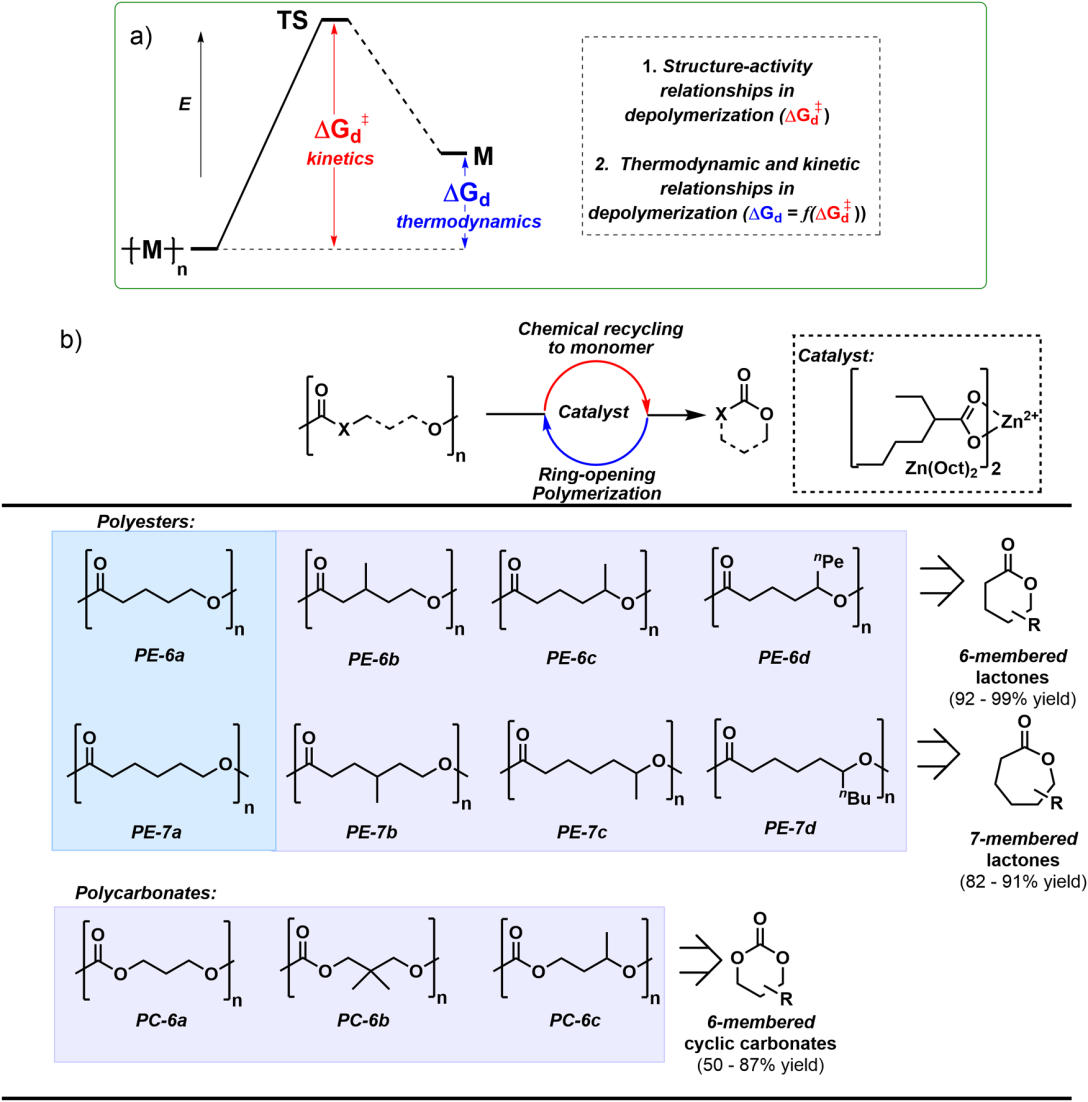
(a) Schematic illustrating the kinetic (Δ*G*_d_^⧧^) and thermodynamic (Δ*G*_d_) barriers to polymer depolymerization. (b) The structures of the catalyst (zinc bis­(2-ethyl hexanoate), Zn­(Oct)_2_) and polymers (all chains are dihydroxyl end-capped); blue shading identifies substitutes for polyolefin plastics while purple shading identifies replacements for elastomers. Typical thermodynamic yields, from depolymerizations, are shown in brackets (further details in the Supporting Information (SI)).

While understanding the thermodynamics of depolymerization provides an excellent starting point for designing chemical recycling processes it is also essential to quantify their kinetic parameters. In the literature, recycling conditions are often selected on the basis of the depolymerization thermodynamics, i.e., using very dilute polymer solutions in carefully selected organic solvents, which requires high catalyst loadings and complicates monomer separations.^31–36^ On the other hand, many large-scale chemical processes are operated under kinetically, not thermodynamically, optimal conditions, for example the Haber Bosch ammonia process or methanol synthesis catalysis.^37,38^ It is also important to select recycling conditions to minimize energy and external chemical usage. Accordingly, the most effective recycling processes should apply neat polymers, limit any solvent use, operate at the lowest temperatures to achieve the highest monomer conversions and rates, use low catalyst loadings and exploit external stimuli (gas flow/vacuum) to drive depolymerization equilibria since the monomers are almost always more volatile than polymers.^39–42^ The polymer structure-recycling investigation in this work will apply such scalable conditions targeting efficient and selective recycling at the lowest possible temperature (energy input).

In recycling catalysis, there is no prior investigation or reporting of how the depolymerization thermodynamics might correlate with the process kinetics.^43^ In other fields of chemistry, these parameters can be correlated through linear free energy relationships. In such circumstances, the rate of the chemical transformation, i.e., the rate constant or transition state Gibb’s free energy, is quantitatively correlated to a thermodynamic parameter, i.e., the equilibrium constant or the process Gibb’s free energy, via Hammett-type or Evans-Bell-Polanyi relationships.^44–46^ So far, there are not yet any investigations into polymer recycling kinetic-thermodynamic relationships. Nonetheless, uncovering such relationships should both accelerate recycling reactions and help guide future material selection. For heterocycle ring-opening polymerizations, there are very few examples of kinetic-thermodynamic relationships and all examples are catalyst dependent. For instance, Duda, Kobayashi and co-workers showed that the Zn­(II) 2-ethylhexanoate (Zn­(Oct)_2_) catalyzed polymerization of 6-membered δ-valerolactone (**6a**, in this work) was significantly faster than the equivalent 7-membered ring, ε-caprolactone (**7a**, in this work), and other larger ring-size monomers.^47^ Using lipase catalysts for polymerizations of the same monomers showed exactly the opposite trend, i.e., the larger ring-monomers polymerized faster than smaller ones.^48^ In other fields of polymer chemistry, linear free energy relationships have been observed in the anionic polymerization of styrene,^49^ various acrylate free radical polymerizations,^50^ and in acrylate atom transfer radical polymerizations but have not been related to recycling reactions.^51^

Here, a series of leading aliphatic polyesters and polycarbonates were selected for the first investigation of polymer recycling catalysis structure-performance relationships (Figure 1) and linear free energy analysis. The series is designed to investigate influences of polymer repeat unit, substituent and end-group chemistry upon depolymerization kinetics. The polymers all feature 6- and 7-atom ester or carbonate linkages; polyesters are named **PE-6** and **PE-7** and polycarbonates are **PC-6**, letters denote substituent positions. Within the series, these materials have variable polymerization equilibrium constants (Table S1). By systematically varying the depolymerization equilibrium constant of the polymer substrate, and measuring their depolymerization rate, the study aims to uncover potential correlations between the rate and equilibrium position of depolymerization. Importantly, the properties of the polymers match those of current hydrocarbon plastics and elastomers—i.e., they are/could be future replacement materials.^20,23,24,30^ Several polymers and monomers are already commercialized products and others could be bioderived.^15^ The academic interest, commercial production volumes and applications for these polymers are fast-growing which underscores the need for an intellectual rationale for their chemical recycling. Importantly, to limit future greenhouse gas emissions, all these polymer recycling processes are thermodynamically feasible at much lower temperatures (>200–300 °C lower) than current hydrocarbon polymers.

## Results and Discussion

All polymers were synthesized via 6- or 7-membered cyclic ester/carbonate ring-opening polymerization (**PE-6**, **PE-7** or **PC-6** series, Table S2). All polymers are dihydroxy terminated (initiated from diols), with degrees of polymerization of *ca* 100, as evidenced using ^1^H NMR spectroscopy and size exclusion chromatography (SEC). The residual polymerization catalysts were carefully removed by repeat precipitations and purifications (see SI for details). Thermogravimetric analysis (TGA) showed that all polymers are stable to temperatures >200 °C, consistent with effective polymerization catalyst removal. The polymers are grouped by substituents: **PE-6a**, **PE-7a**, and **PC-6a** are unsubstituted, **PE-6b**, **PE-7b**, and **PC-6b** have ‘midchain’ methyl substituents (2 atoms from the acyl oxygen for **PE-6b** and **PE-7b**, and one atom for **PC-6b**), **PE-6c**, **PE-7c**, and **PC-6c** have methyl groups adjacent to an acyl oxygen. The recycling catalyst zinc bis­(2-ethylhexanoate) (Zn­(Oct)_2_) was selected because of the strong precedent for zinc complexes in efficient oxygenated polymer depolymerizations.^42,52,53^ Additional benefits of Zn­(Oct)_2_ are that it is commercial, inexpensive, colorless, soluble in the polymer melt and stable to high temperature. To establish low-energy recycling test conditions, depolymerizations were conducted systematically using neat polymer films, containing 1:100–1:1000 Zn­(Oct)_2_:polymer repeat unit loadings. These polymer/catalyst films were heated, inside round bottomed flasks, at constant temperatures, from 130 to 190 °C, under dynamic vacuum (1–20 mbar). Under these catalyzed recycling conditions, the polymers were selectively transformed into their respective lactones or cyclic carbonates with very high selectivity (>99% in all cases). The monomers were isolated in excellent yields (>50–99%) and high purity (>90%), as determined by ^1^H NMR spectroscopy and GC-MS, respectively (Table S3, Figures S2–S26). To demonstrate that the isolated monomers could be used to reform equivalent polymers, **6a** was repolymerized to form **PE-6a** of slightly higher molar mass, with near identical thermal properties (Figures S27–29).

### Depolymerization Kinetics

Having established that Zn­(Oct)_2_ is a highly effective polyester depolymerization catalyst, we next sought to understand the recycling chemistries under common conditions. The depolymerization kinetics were investigated using an iso-thermal recycling methodology reported previously,^40,42^ whereby conversion vs time data is continually monitored using a TGA instrument equipped with an in-line IR spectrometer to characterize products. All polymers were studied using the TGA method and shown to exhibit similar behavior. As a representative example, the depolymerization of **PE-6a** will be discussed in detail. Accordingly, the polymer film comprising 1:1000 loading of Zn­(Oct)_2_:**PE-6a** (poly­(δ-valerolactone)) was heated at 130 °C, with a nitrogen gas flow of 25 mL min^–1^, and the mass loss vs. time data monitored. The reaction resulted in quantitative **PE-6a** depolymerization to its cyclic ester **6a** in 20 min, with the maximum depolymerization rate occurring at ∼30% mass loss. Under these conditions, the polymer chemical recycling activity or turnover frequency (TOF, at 30% conversion) is very high at 7000 h^–1^ (Figure S30, Table S4). In-line FTIR spectroscopy confirmed the selective formation of only the desired monomer **6a** (Figure S31). Using a TGA instrument for these measurements assumes that the mass loss rate correlates with the depolymerization rate. It is important that the depolymerization rates occur more slowly than other physical processes required for effective recycling, i.e., diffusion of the monomer through the film and monomer volatilization must be faster than monomer formation. As test reactions, the mass loss rates from films of only the monomer, **6a**, and of monomer/polymer mixtures, **6a**/**PE-6a,** were measured (Figure S30 and Table S4). The volatilization and diffusion rates for **6a** are considerably (3–20×) faster than the equivalent rates measured for the catalyzed **PE-6a** depolymerizations. Thus, the rates of depolymerization can be properly measured using the TGA instrument, with minimal contributions from other physical processes.

To appropriately model the depolymerization kinetics, it is helpful to consider how the polymer:catalyst film evolves during the recycling. Thus, a series of films comprising **PE-6a** and cobalt­(II) bis­(2-ethylhexanoate) Co­(Oct)_2_, at 1:100, [Co­(Oct)_2_]_0_:[**PE-6a** repeat unit]_0_, were depolymerized using the TGA procedures. The experiments were stopped (by opening the pan to air) at regular 20% mass loss intervals, and the pan contents were photographed. The cobalt­(II) catalyst was chosen since it shows nearly equivalent rates to Zn­(Oct)_2_ (Table S4) but it is highly colored providing a visible marker of the film edge (volume). Qualitatively, the photographs reveal that the polymer films remain intact throughout the depolymerization but as the recycling progresses the films contract which should increase the local catalyst concentration; such a variable catalyst concentration is expected since the monomer is continually removed throughout the depolymerization (Figure S32). To account for the increased catalyst concentration, sigmoidal functions were used to fit the mass loss vs time data for the **PE-6a** depolymerization (1:1000, Zn­(Oct)_2_:**PE-6a**, at 130 °C Figure S33). Such sigmoidal functions are most appropriate to model the kinetics for reactions with variable (increasing) catalyst concentration, e.g., autocatalysis.^54,55^ The kinetic model shows an excellent fit to the experimental Zn­(II) catalyzed polymer recycling data (*R* ^2^ > 0.99). Next, the **PE-6a** catalyzed depolymerization was monitored at reaction temperatures between 100 and 140 °C (Figure S33). Using Friedman isoconversional analysis, the activation energy, *E*_a_, was estimated at intervals of 10% polymer conversion by taking the gradient of plots of ln of the time taken to reach the conversion interval vs reciprocal temperature (Figure S34 and Table S5).^56^ The average *E*_a_ was determined to be +65.7 ± 1.7 kJ mol^–1^ with a standard deviation of <3%. This variation is well within ±10%, indicating that the depolymerization can be effectively modeled using a single process, and suggesting influences of changing polymer molar mass and viscosity appear to have minimal effect on the depolymerization rate during the reaction.^54^ Across this temperature range, the experimental data also all fit very well to the sigmoidal kinetic model (*R* ^2^ > 0.99, Figure S33). The resulting rate constants, measured at the maximum rate (see SI for details) were used to determine recycling catalyst activation energies, via Arrhenius analysis; the depolymerization activation energy is identical to values calculated using Friedman iso-conversion analysis and very close to literature values reported for similar polymers and catalysts (Figure S35, Tables S6 and S7).^56^ Thus, the sigmoidal kinetic method should enable determination and comparison of depolymerization rates over a range of different conditions.

Depolymerization reactions may occur via two general types of pathway: catalysis occurring from the polymer chain-end, in which monomer extrudes from an active catalyst-chain-end group, or via random polymer chain-scission reactions, where the monomer is formed at any position along the polymer chain. To distinguish between these two mechanisms, a sample of **PE-7b** featuring acetyl end-groups was prepared (**PE-7b-OAc**). These ester end-capped samples were subjected to the same depolymerization conditions used for the equivalent dihydroxyl end-capped polyesters (1:100, Zn­(Oct)_2_:[**PE-7b-OAc**], 130 °C). Over 1 h, there was negligible depolymerization of the acetyl-sample (<5% mass loss) whereas the hydroxyl-end-capped polymer **PE-7b** showed >95% mass loss over the same time scale (Figure S36).

These results suggest that most recycling catalysis occurs via a polymer chain-end mechanism. They also indicate that the catalyst reacts rapidly with the polymer hydroxyl end-groups to form the zinc alkoxide active species: these initiation reactions likely occur faster than depolymerization as initiation with Zn­(Oct)_2_ is known to occur quickly at temperatures of 80 °C.^57^ The depolymerization is proposed to follow a series of sequential reactions whereby the polymer chain-end zinc alkoxide undergoes intramolecular transesterification to extrude the lactone monomers and reform, after every lactone release, a polymer chain-shortened Zn-alkoxide species (Figure 2a). There are clear parallels between the proposed depolymerization mechanism and the coordination–insertion mechanism for lactone ring-opening polymerization catalysis. Indeed, the initiation reaction (formation of the polymer–O-Zn active site) is the same as that known for Zn­(Oct)_2_, and other metal bis­(2-ethylhexanoate), catalysts in lactide or other cyclic ester ring-opening polymerizations.^58,59^ To investigate the order in catalyst concentration, **PE-7b,** poly­(4-methyl-ε-caprolactone), was selected since it shows intermediate rates within the series of polymers (Table S20). As such, it should enable accurate depolymerization rate measurements, at 130 °C, over a wide range of catalyst concentrations. In these experiments, the loading of Zn­(Oct)_2_, in the films with **PE-7b**, was varied by a factor of 10, from 1:100 to 1:1000 [Zn­(Oct)_2_]_0_:[**PE-7b** repeat units]_0,_ [Zn­(Oct)_2_]_0_ = 7.80 × 10^–3^–7.80 × 10^–2^ M). As expected, at lower catalyst loadings the recycling occurred with slower rates. The plot of ln­(*k*_obs_) vs ln­(catalyst concentration) has a gradient ≈ 1, indicating that the depolymerization reaction is first order with respect to catalyst concentration, i.e., *k*_obs_ = *k*_d_′[Zn­(Oct)_2_]_0_ (Table S8, Figures S37–S39). Assuming the same rate law should apply to all the polymers enables quantified comparisons of their depolymerization rates.

**Figure 2:**
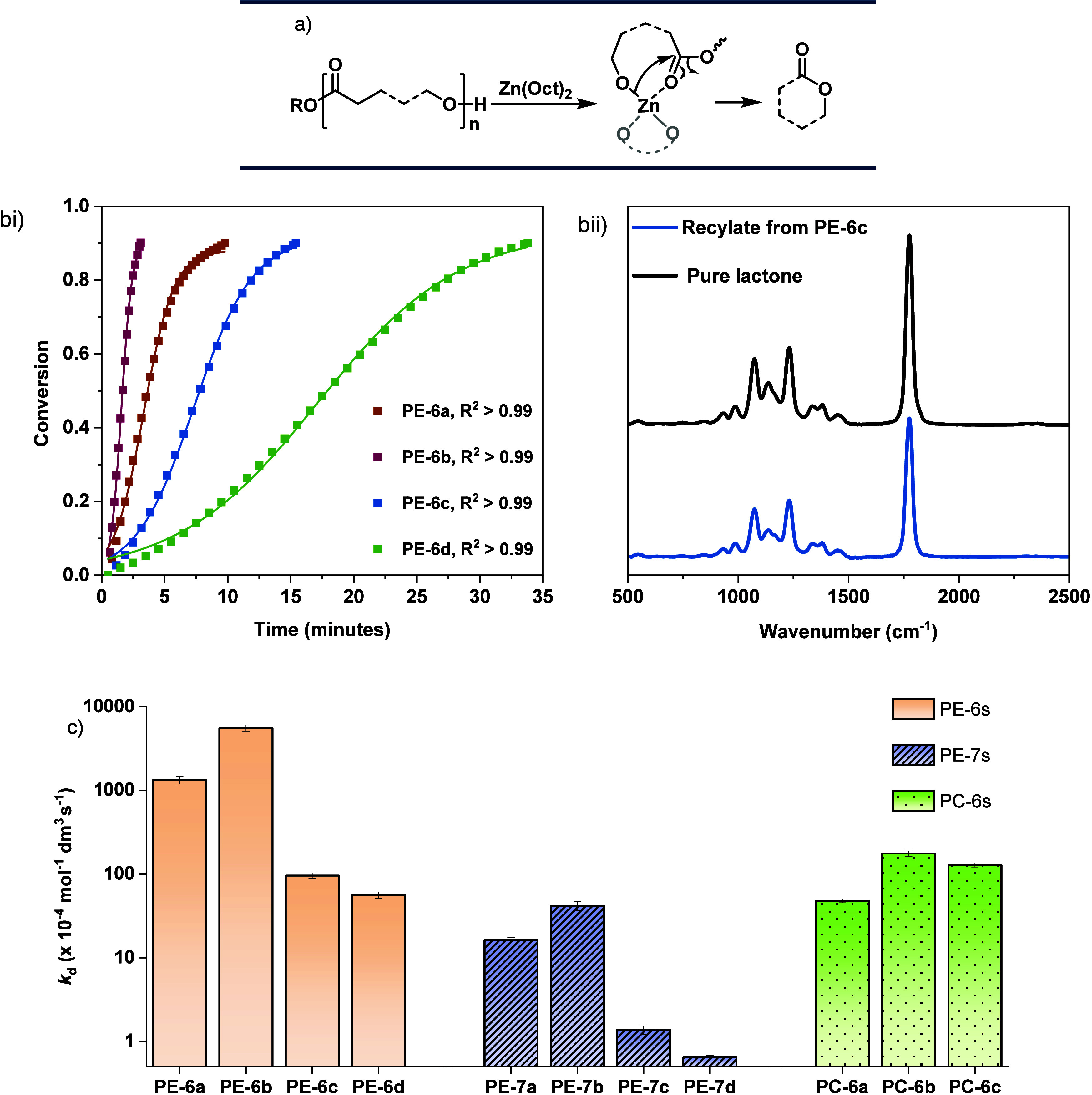
(a) Proposed mechanism for the Zn­(Oct)_2_-catalyzed depolymerization of aliphatic polyesters. (b) Chemical recycling of neat polymer catalyst films to monomers. (bi) Plots of conversion (where conversion = 1 – *m*_polymer,*t*_/*m*_polymer,0_) vs time and sigmoidal fits. Recycling conditions: Zn­(Oct)_2_ (at 1:1000, [catalyst]_0_: [**PE-6a**]_0_ or [**PE-6b**]_0_ repeat unit, and 1:100 catalyst: [**PE-6c**]_0_ or [**PE-6d**]_0_ repeat unit), at 130 °C, N_2_ flow = 25 mL min^–1^. (bii) FTIR spectra comparing the **PE-6c** recycled product and a pure sample of the lactone, **6c**. (c) Depolymerization rate constants for recycling in neat polymer films, at 130 °C, catalyzed by Zn­(Oct)_2_ and N_2_ flow 25 mL min^–1^. [Zn­(Oct)_2_]_0_: [polymer]_0_ = 1:1000–1:100 (see Table S19). Rate = *k*_d_2­[Zn­(Oct)_2_]_0_ where *k*_d_ = *k*_obs_/(2­[Zn­(Oct)_2_]_0_).

The series of 8 polyesters (**PE-6a-d** and **PE-7a-d**) and 3 polycarbonates (**PC-6a-c**) were all assessed for catalyzed chemical recycling to lactone or cyclic carbonate. In all experiments, the Zn:polymer loadings were in the range 1:100–1:1000 and temperatures were all 130 °C, with N_2_ flows of 25 mL min^–1^. The recycling temperature of 130 °C was chosen to ensure that all reactions were conducted at temperatures at least >30 °C above the *T*_g_ or *T*_m_ of the polymers. All the polymers were effectively and highly selectively recycled to their monomers, with kinetic fits showing excellent correlations with the experimental data (*R* ^2^ > 0.99, Figure 2bi, Tables S9–S19, Figures S40–S50). The reaction products were all analyzed using in-line FTIR spectroscopy which confirmed the selective formation of only the monomers, i.e., lactones or cyclic carbonates (Figure 2bii, Figures S51–S60).

The recycling rates, as assessed by both the depolymerization rate constant and point-activity values (TOF), are clearly influenced by the polymer structure (Figure 2c). This is consistent with each polymer forming a different type of zinc-alkoxide (catalyst-polymer) active site. Polyesters with 6-atom repeat units, **PE-6** series, all depolymerized fastest, followed by the 6-repeat unit polycarbonates, **PC-6** series, and the 7-repeat unit polyesters, **PE-7** series, depolymerizing the slowest. For example, under comparable conditions, the polyester **PE-6a** (PVL) has a recycling activity of 7000 h^–1^ while the analogous polycarbonate, **PC-6a** (poly­(trimethylene carbonate), PTMC) shows an activity of 400 h^–1^ and the related polyester **PE-7a** (PCL) shows an activity of just 60 h^–1^. Polymers featuring midchain methyl substituents (**PE-6b**, **PE-7b**, and **PC-6b**), depolymerize faster than analogous polymers without the substituents, under equivalent conditions. This effect is clearly demonstrated by comparing the recycling activity for the polyester **PE-6b** with its TOF of 15,900 h^–1^ vs the TOF of 7000 h^–1^ for unsubstituted **PE-6a**. The position of the methyl substituent is important since polymers featuring methyl, or alkyl, substituents adjacent to the acyl oxygen (of the ester repeat unit), rather than midchain, show slower recycling rates than those without substituents. This effect is clearly demonstrated by comparing the TOF values for methyl-substituted **PE-6c,** TOF = 370 h^–1^ under equivalent conditions to the unsubstituted polyester **PE-6a**, TOF = 7000 h^–1^. Comparing the recycling (depolymerization) rate constants to the systematic changes to the polymer structures reveals several general trends (Table S20). (1) Changing the repeat unit from 7- to 6- atoms, with all other features remaining constant, increases the depolymerization rates by a factor of ∼100. (2) Changing the repeat chemistry from carbonate to ester increases the depolymerization rates by a factor of ∼25. (3) Polymers featuring ‘midchain’ substituents show faster depolymerization rates than those without any substituents by a factor of ∼5. (4) Polymers featuring alkyl substituents adjacent to the acyl oxygen in the repeat unit show slower rates, by a factor of ∼10 (∼20 for larger alkyl substituents), compared to those without any substituents. A key implication of these differences in rate is that where polymers show otherwise similar physical-chemical properties, it would be beneficial to select chemistries resulting in faster recycling. It may also be feasible to exploit these structure-recycling relationships to enable reactive recycling as a method to sort/separate structurally similar polymers.

### Polymer Structure-Recycling Rate Relationships

The kinetic depolymerization barriers were next evaluated using Eyring analysis. For each polymer, the depolymerization rate constant was determined at five temperatures between 90 and 190 °C, using [Zn­(Oct)_2_]_0_:[Polymer]_0_ = 1:100–1:1000 (Figure 3a, Tables S6, S9–S19). Plots of ln­(*k*_d_/*T*) vs reciprocal temperature (1/*T*) enabled determination of depolymerization transition-state (kinetic) enthalpy (Δ*H*_d_^⧧^, gradient), entropy (Δ*S*_d_^⧧^, *y*-intercept) and free energy barriers (Δ*G*_d_^⧧^, Figure 3b, Table S21, Figures S61–74). For all the polymer recycling reactions, the transition state enthalpy barrier, Δ*H*_d_^⧧^, is positive and the entropy barrier, Δ*S*_d_^⧧^, is negative. Considering the depolymerization pathway, the enthalpy barrier should relate to the nucleophilicity of the Zn-alkoxide­(polymer) and/or electrophilicity of the polymer carbonyl group.

**Figure 3:**
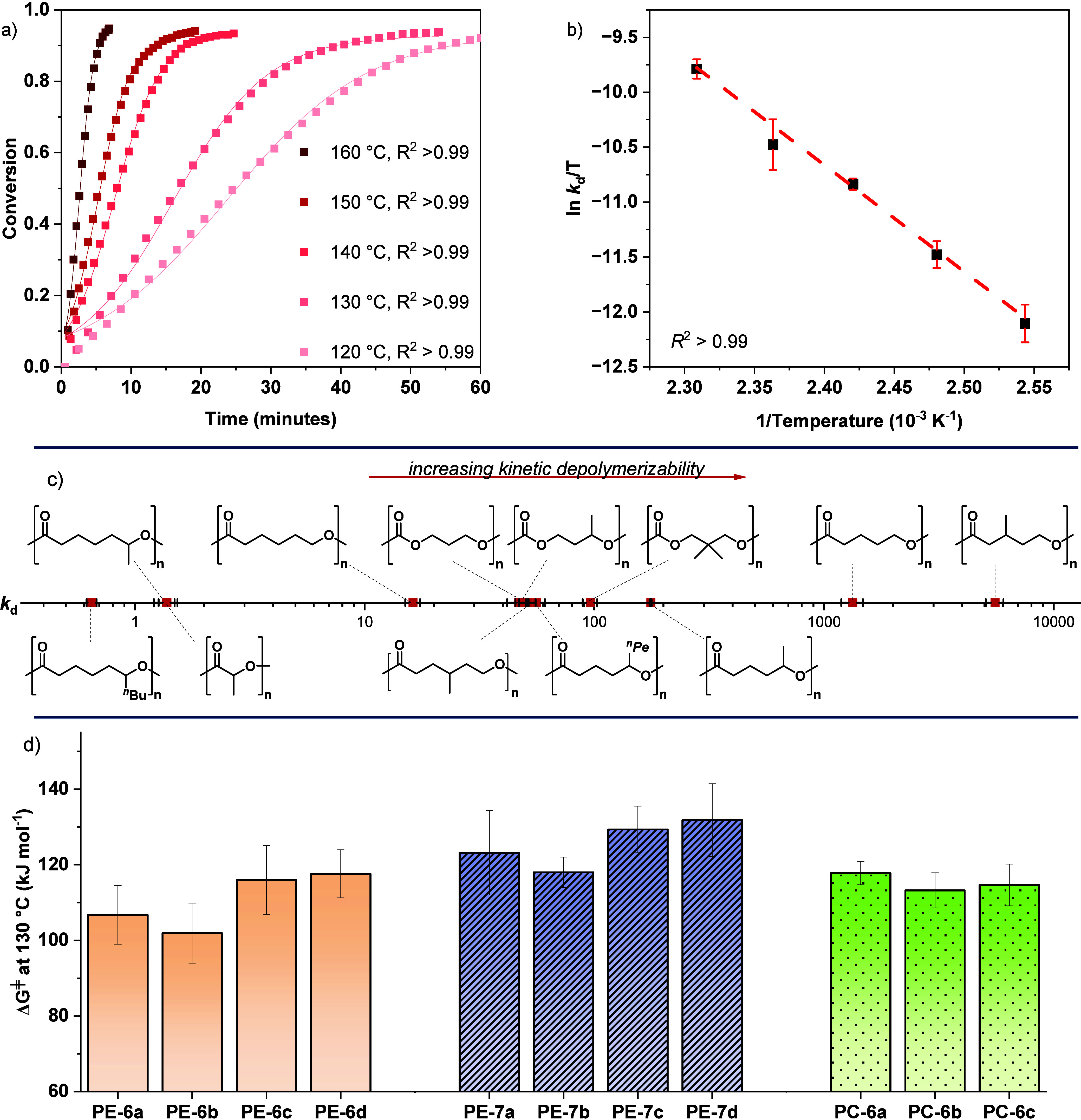
(a) Chemical recycling rate data for catalyzed depolymerization of **PE-7b** to **7b** at temperatures from 120 to 160 °C (conditions: [Zn­(Oct)_2_]_0_:[**PE-7b**]_0_ = 1:100). Data are collected in triplicate and in each case are fit using the sigmoidal function (for rate constants, see Table S14). (b) Plot of ln­(*k*_d_/*T*) vs 1/*T* for the **PE-7b** recycling. The data was linearly fit and used to determine kinetic barriers, i.e., Δ*H*_d_^⧧^, Δ*S*_d_^⧧^, and Δ*G*_d_^⧧^ (reactions repeated in triplicate; the errors are determined as the standard deviations of the mean). (c) Plot showing the change in rate constant at 130 °C, *k*_d_ (×10^–4^ mol^–1^ dm^3^ s^–1^), across the polymer series on a log scale. (d) Plot illustrating the experimentally determined kinetic transition state barriers at 130 °C; Δ*G*_d_^⧧^ determined from Eyring analysis for each of the polymers used in this study, see Table S21.

The entropy barrier arises because the transition state, to cyclic monomer formation, requires the polymer chain to be arranged into a pseudocyclic structure (preordering of the transition state). Based on experimental evidence, it is not possible to conclusively identify the rate determining-step. However, it is tentatively proposed that the large entropy values across the series are potentially consistent with a ‘late’ transition state,^41^ i.e., a significant polymer-catalyst conformational change likely occurs between the resting state and rate-determining steps in the catalytic cycle. Finally, as expected from the Eyring equation, it is emphasized that the recycling rates differ from 1 to ∼10,000×, despite relatively small differences between the transition state free energy values, Δ*G*_d_^⧧^ (Figure 3c,d).

### Polyesters with 6-Atom vs 7-Atom Linkers

In general, the kinetic barriers, as assessed by the transition-state free-energy difference, Δ*G*_d_^⧧^, for the 6-atom polyester, **PE-6** series, were ∼15 kJ mol^–1^ lower than for the same materials featuring 7-atom repeat units, **PE-7**s, consistent with their faster recycling (Table 1, entries 1–8. Figure 3c). For example, **PE-6b** depolymerizes around ∼100× faster than **PE-7b**, at 130 °C, with a kinetic barrier difference of 16 kJ mol^–1^.

**Table 1:** Kinetic Recycling Barriers, Δ*H* ^⧧^, Δ*S* ^⧧^, and Δ*G* ^⧧^, Determined from Eyring Analysis^a^

entry	polymer	Δ*H* ^⧧^ ^b^ (kJ mol^–1^)	Δ*S* ^⧧^ ^c^ (J mol^–1^)	Δ*G* ^⧧^ ^d^ (kJ mol^–1^)	*k*_d_ (×10^–4^ mol^–1^ s^–1^ dm^3^)
1	**PE-6a**	62 ± 5	–111 ± 15	107 ± 8	1300
2	**PE-6b**	77 ± 7	–63 ± 9	102 ± 8	5500
3	**PE-6c**	82 ± 7	–85 ± 14	116 ± 9	96
4	**PE-6d**	96 ± 6	–53 ± 5	118 ± 6	56
5	**PE-7a**	71 ± 5	–128 ± 24	123 ± 11	16
6	**PE-7b**	81 ± 3	–93 ± 7	118 ± 4	51
7	**PE-7c**	78 ± 3	–127 ± 13	129 ± 6	1.4
8	**PE-7d**	78 ± 4	–133 ± 21	132 ± 10	0.65
9	**PC-6a**	96 ± 2	–55 ± 5	118 ± 2	48
10	**PC-6b**	88 ± 3	–62 ± 8	113 ± 4	47
11	**PC-6c**	87 ± 3	–67 ± 7	114 ± 3	180

a For further information, see Table S21, Figures S61–S74.

b Δ*H* ^⧧^ = −*m* × 8.314.

c Δ*S* ^⧧^ = 8.314 (*c* – ln *k*_b_/*h*) where *k*_b_ = Boltzmann constant, *h* = Planck constant.

d Δ*G* ^⧧^ = Δ*H* ^⧧^ – 403.14 × Δ*S* ^⧧^.

Examining the kinetic data reveals that the transition state barrier difference arises from a lower entropy penalty associated with cyclic monomer formation from the **PE-6** series. Notably all these 6-atom repeat unit polyesters have lower Δ*S*_d_^⧧^ values compared with analogous **PE-7** structures (Figure 4a). The data are interpreted by a reduced transition state preordering when forming a 6- vs 7-membered lactone.

**Figure 4:**
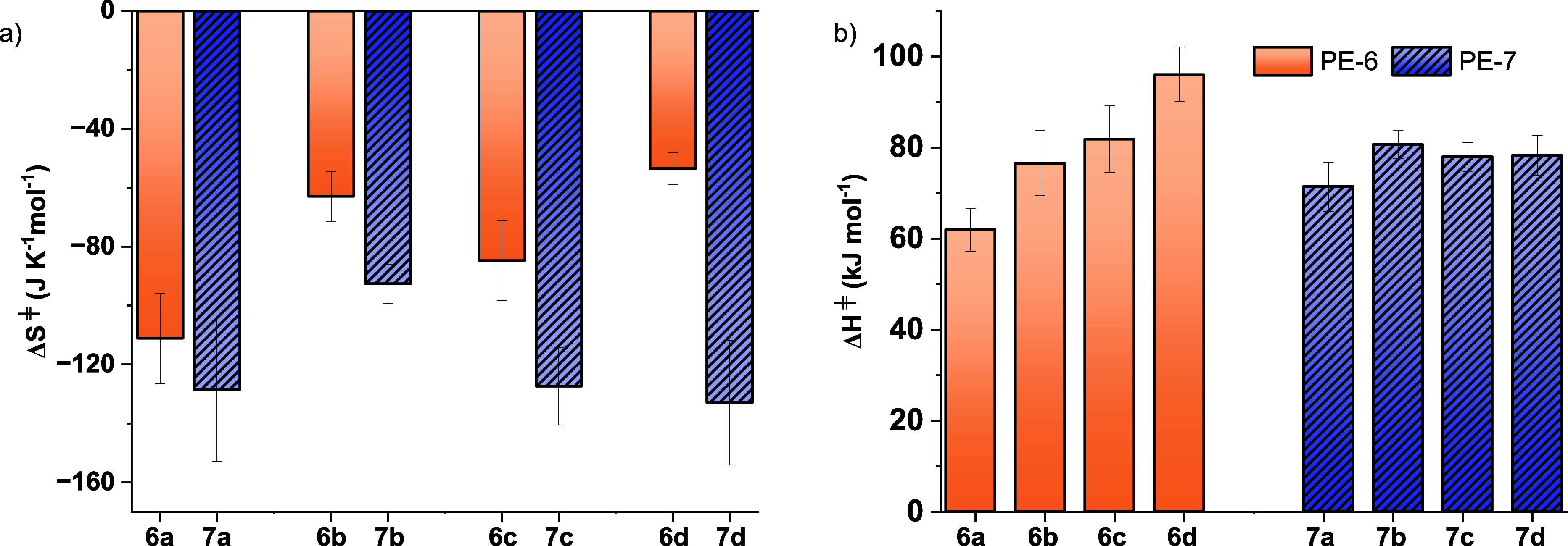
Comparisons of transition state barriers for the **PE-6** (orange) and **PE-7** (purple) polyester series. (a) illustrates the entropy barriers, Δ*S*_d_^⧧^ for the analogous 6- and 7-atom repeat unit polyesters and (b) illustrates the enthalpy barriers, Δ*H*_d_^⧧^ for the same 6- and 7-atom repeat unit polyesters.

### Polycarbonates vs Polyesters

Comparing the polyester and carbonate series, **PE-6** vs **PC-6**, reveals depolymerization transition state barriers which are ∼11 kJ mol^–1^ lower for the former, i.e., the polyesters have faster depolymerization rates. Comparison of the specific data for **PE-6a** (PVL) and **PC-6a** (PTMC) suggests the lower barrier for the polyesters stems from changes to the transition state enthalpy, Δ*H*_d_^⧧^ which are lower for the esters vs carbonates (96 kJ mol^–1^ vs 62 kJ mol^–1^, Figure S73, Table 1, entries 1 and 9). This transition state enthalpy difference is tentatively attributed to the higher electrophilicity of the ester groups (carbonyls) compared with the equivalent carbonate carbonyl group.

### Primary vs Secondary Alkoxides

The position of the methyl (or other alkyl) substituent also significantly impacts the recycling kinetics. For both the 6- and 7-atom polyester **PE-6** and **PE-7** families, methyl substituents adjacent to the acyl oxygen increase the overall transition state barriers by around 6–9 kJ mol^–1^. For example, at 130 °C, unsubstituted **PE-6a** depolymerizes *ca* 10 × faster than methyl substituted **PE-6c** and shows a 9 kJ mol^–1^ lower barrier. The difference is attributed to changing the steric hindrance of the (de)­propagating alkoxide, i.e., formation of a secondary (methyl-substituted) rather than primary (unsubstituted) zinc-alkoxide nucleophile. The hypothesis is supported by higher Δ*H*_d_^⧧^ values for **PE-6a** than **PE-6c** (62 kJ mol^–1^ vs 82 kJ mol^–1^, Figure 4b, Table 1 entries 1 and 3). Larger alkyl substituents at this position also further increase the transition state barrier (and thereby decrease the rate). It is noted that **PC-6a/PC-6c** do not follow this trend likely because **PC-6c** contains mixed primary and secondary chain-ends which dilute the impact of chain-end sterics on the Δ*H*_d_^⧧^.

### ‘Mid-Chain’ Methyl Substituents

Polymers that feature midchain methyl substituents have lower overall transition state barriers by ∼5 kJ mol^–1^, than those without any substituents, resulting in faster recycling. For example, at 130 °C, **PE-6b** depolymerizes 4 × faster than **PE-6a**, which corresponds to a difference between transition state barriers of ∼5 kJ mol^–1^. For the polyesters, the reduction appears to be caused by lower entropy change values, as shown by lower Δ*S*_d_^⧧^ for **PE-6b** vs **PE-6a** (−63 vs 111 J K^–1^ mol^–1^) and **PE-7b** vs **PE-7a** (−93 vs 128 J K^–1^ mol^–1^, Figure 4a, Table 1, entries 1, 2, 5, and 6). The reduced entropy penalty compensates for the increased enthalpy penalty associated with these structures, and suggests that midchain substituents facilitate transition state preordering.

In contrast, the midchain substituted polycarbonates, e.g., **PC-6b**, show lower barriers which appear to correlate with lower enthalpy differences (96 kJ mol^–1^ vs 88 kJ mol^–1^, Table 1, entries 9 and 10, Figure S74). This difference is tentatively ascribed to the increased torsional strain in PC-6b as a consequence of the geminal dimethyl substituents in the repeat unit. This increases the ground-state energy of the substituted polymer decreasing the enthalpy cost to intramolecular cyclization. This effect is less pronounced for the polyesters which are only singly substituted in the repeat unit. Overall, the data highlights the benefit of making comparisons between the polymers, under equivalent conditions, and how main chain, substituent and end-group chemistries influence depolymerization kinetics.

### Computational, DFT-Study

To further investigate the depolymerization pathway, computational modeling of the heterocycle ring closure (proposed rate-determining step) was undertaken using DFT calculations. Based on the polymer chain end-capping experiments, which suggest that the catalyst active depolymerization species is a Zn­(II) alkoxide, and on the solid-state structures of zinc carboxylates,^60^ the active catalyst was modeled as a tetrahedral Zn­(II) alkoxide. There is a significant rate difference between the depolymerization of **PE-6a** (poly­(δ-valerolactone)) and **PE-7a** (poly­(ε-caprolactone)), so these two materials were selected for the ring-closing mechanism investigation. Methyl 5-hydroxypentanoate was used to model the transition state for **PE-6a**, and methyl 6-hydroxyhexanoate for **PE-7a**. The ring-closure was modeled via a two-step addition and elimination mechanism (Figure S75 and Table S22). In the model, the transition state involves the Zn-alkoxide undergoing a nucleophilic attack at an ester carbonyl group, along the polymer chain, to form a Zn-acetal intermediate. The Zn-acetal intermediate reacts to ring-close the target lactone and extrude it, forming a Zn-methoxide species. The DFT calculations suggest that the ring closure of 6-hydroxyhexanoate has a significantly higher barrier (∼15 kJ mol^–1^) than the methyl 5-hydroxypentanoate. These calculations are consistent with the overall decreased rate observed with **PE-7a** compared to **PE-6a**. Finally, for both substrates, the DFT calculated barriers for the addition and elimination steps are within error. It is therefore not possible assign either step as rate-determining and thus the transition state is drawn to show it may have character of both (Figure 5cii).

**Figure 5:**
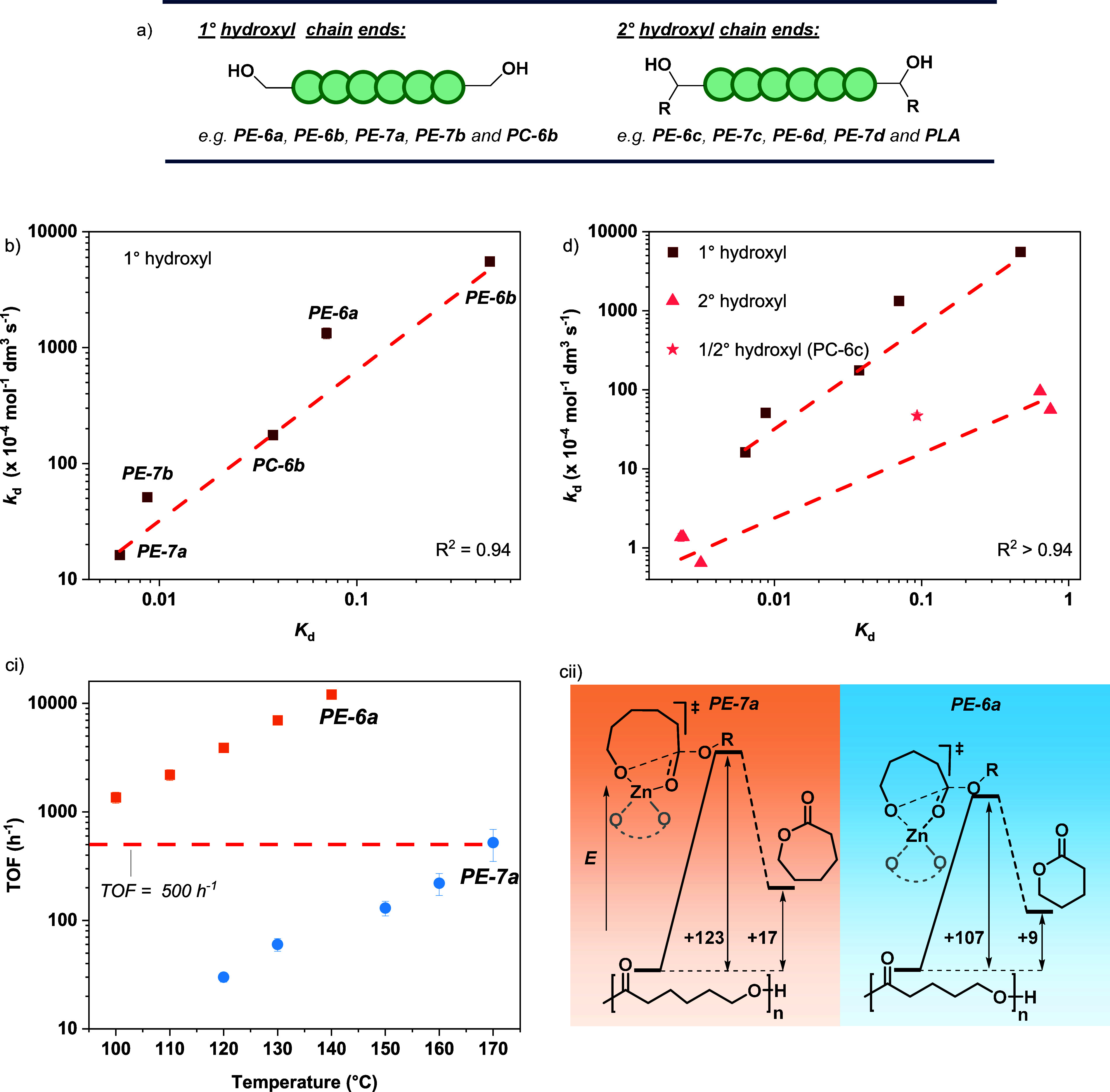
Correlations of catalyzed chemical recycling thermodynamic and kinetic parameters, resulting in clear linear free energy relationships. (a) Schematic illustrating primary and secondary hydroxyl chain-ends. (b) Plots of the depolymerization rate constant, *k*_d_, vs the depolymerization equilibrium constant, *K*_d_ at 130 °C for **PE-6a**, **PE-6b**, **PE-7a**, **PE-7b**, and **PC-6b**; reactions conducted at [Zn­(Oct)_2_]_0_: [polymer]_0_, 1:1000. (ci) Plots of recycling activity, as assessed by TOF, against temperature for **PE-6a** and **PE-7a**. (cii) Schematic illustrating the measured kinetic and thermodynamic parameters, Δ*G*_d_^⧧^ and Δ*G*_d_ at 130 °C for poly­(ε-caprolactone), **PE-7a**, and poly­(δ-valerolactone), **PE-6a**. (d) Plot of the depolymerization rate constant, *k*_d_ vs the depolymerization equilibrium constant *K*_d_ at 130 °C for all polymers in this study. N.B. an additional sample, polylactide (racemic), is included in this data set.

Further DFT calculations were undertaken to understand why the depolymerizations are selective for reformation of the monomer rather than larger (e.g., dimeric) heterocycles. The intramolecular cyclization of a 5-methoxy-5-oxopentyl 5-hydroxypentanoate to form the monomer (**6a**) or dimer­(**6a**_**2**_) was thus modeled via a two-step addition and elimination mechanism (Figure S76 and Table S23). The formation of **6a** was found to be both kinetically and thermodynamically favored by –18.7 and –36.8 kJ mol^–1^, respectively. This is tentatively ascribed to differences in the enthalpy and entropy when forming **6a**_**2**_ vs **6a**. Reaction to **6a**_**2**_ is expected to have a larger enthalpy cost due to the formation of a high-strain, 12-atom pseudocyclic conformer during cyclization. Similarly, the high strain of **6a**_**2**_ likely thermodynamically destabilizes the dimer as compared with the monomer. The formation of **6a**_**2**_ is also likely less entropically favored as more atoms lose conformational freedom in the cyclic dimer vs the cyclic monomer. Overall, these results are fully consistent with experiments which show the reactions are highly selective for formation of monomer.

### Linear Free Energy Relationships in Recycling Catalysis

The detailed investigation of the catalyzed chemical recycling kinetic parameters provides an opportunity to understand any correlations with the depolymerization thermodynamic parameters (Figure 1a). Therefore, the depolymerization rate constants (i.e., kinetic, *k*_d_) were plotted against the depolymerization equilibrium constants (thermodynamic, *K*_d_). As conditions significantly influence the position of the monomer/polymer equilibrium, and hence the thermodynamic parameters, all the thermodynamic data were selected from experiments conducted in neat polymer/monomer, i.e. the same conditions as were used in the kinetic measurements (Table S1).^9^ It has already been established that polymer substituents adjacent to the chain-end (acyl group) exert a significant influence on the depolymerization kinetics (transition state barrier and rate). Thus, in making comparisons, the polymers are grouped by primary or secondary alkoxide (catalyst end-group) chemistry (Figure 5a).

Remarkably, all the polymers show a clear exponential relationship between the depolymerization kinetic rate constants (*k*_d_) and the thermodynamic depolymerization equilibrium constants (*K*_d_) (Figure 5b). The data confirm that polymers with the largest thermodynamic driving force for depolymerization, such as **PE-6b** (*K*_d_ = 0.47, ∼35% polymer conversion at equilibrium), have the fastest rates of depolymerization (*k*_d_ = 0.56 mol^–1^ dm^3^ s^–1^). At the other end of the relationship, polymers with smaller driving forces for depolymerization, such as **PE-7a** (*K*_d_ = 0.0063, ∼1% polymer conversion at equilibrium), have the slowest rates of depolymerization (*k*_d_ = 1.0 × 10^–3^ mol^–1^ dm^3^ s^–1^). There is a clear linear free energy relationship between the kinetic transition state barrier Δ*G*_d_^⧧^, and thermodynamic barrier, Δ*G*_d_ (Figure S77). The practical consequence is that polymers which are thermodynamically likely to depolymerize (i.e., Δ*G*_d_ < 0) also show low transition state barriers at 130 °C (i.e., smaller Δ*G*_d_^⧧^).

One interpretation of this data, supported by the significant entropy barriers to depolymerization, is that the transition-state is ‘late’ and resembles a ‘pseudomonomer’. In other words, the propensity of a particular monomer to form is controlled by a balance between its enthalpy and entropy contributions, which together comprise the overall energetic requirements of the depolymerization process. The consequence of the linear free energy relationship is that the same factors that influence the stability of a monomer appear to influence the stability of the depolymerization transition-state.

There are a number of important practical implications resulting from the linear relationship between the kinetic and thermodynamic free energy barriers.

One significant impact is to use the data to accurately predict the lowest temperatures that can be used to enable efficient recycling for the most challenging polymers, i.e., those with high free energy barriers. One such material is the commercial polyester poly­(ε-caprolactone), **PE-7a**, which is already sold for uses in consumer goods and packaging. Within the series of polymers, its depolymerization is among the most thermodynamically disfavored, showing a polymer/monomer equilibrium constant of *K*_d_^130 °C^ = 0.0063, resulting in ∼1% polymer conversion at equilibrium (note that quantitative depolymerization conversion to ε-caprolactone is feasible using gas flow to drive the reaction). This polyester has a slow depolymerization rate and high depolymerization transition state barrier. We considered an effective recycling rate to occur when recycling catalyst activity exceeds TOF > 500 h^–1^.

Using the linear free energy relationship data enables the determination of the recycling temperature needed to deliver such rates to be >170 °C (Figures 5c and S78). These temperatures are quite accessible, particularly compared to those which would be required for any equivalent commercial hydrocarbon plastic.^11–14^ In comparison, PVL **PE-6a**, which also shows properties and promise as a polyolefin plastic replacement,^20^ shows much faster and more thermodynamically feasible depolymerizations, with *K*_d_^130°C^ = 0.070, resulting in ∼6% polymer conversion at equilibrium (noting quantitative δ-valerolactone conversion is readily feasible by driving the equilibrium with gas flow).

For **PE-6a**, highly efficient catalyzed depolymerization, with TOF ≫ 500 h^–1^, is possible at just 100 °C. It is essential to appreciate these are the lowest temperatures for fast and selective catalyzed recycling: the pure polymers are thermally stable and show significantly higher temperature stability to enable common processing, uses and applications (Table S2). Both PCL (**PE-7a**) and PVL (**PE-6a**) show very similar thermal properties, with melting temperatures, *T*_m_ ≈ 50 °C, and glass transition temperatures, *T*_g_ ≈ −60 °C, and closely related mechanical properties, e.g. tensile strength values, **PE-6a** ∼ 20–60 MPa and **PE-7a** ∼ 10–30 MPa.^20,61^ As such, it may be interesting for future applications to explicitly consider their different relative recycling rates. Quantitative chemical recycling of **PE-6a** requires lower temperatures (lower energy input) than are needed for **PE-7a**. Considering the polymer series featuring substituents adjacent to the acyl oxygen (secondary alkoxides) reveals similar trends. These alkyl-substituted polymers are elastomers, showing favorable low glass transition temperatures (Table S2). They are important as components of block polymer thermoplastic elastomers and adhesives—both material classes would benefit from efficient chemical recycling to monomer(s).^24^ In this series of polymers, the linear free energy relationship (exponential correlation) is clear between the recycling rate *k*_d_ and equilibrium constant *K*_d_ and a linear relationship is observed between the kinetic transition state barrier, Δ*G*_d_^⧧^ and the thermodynamic energy change Δ*G*_d_ (Figures 5d and S79). Where polymers show otherwise similar properties (Table S2), those materials featuring midchain methyl substituents should be prioritized over those with methyl groups adjacent to the acyl oxygen for application development since they are recycled much faster. For instance, polyesters **PE-6b** and **PE-6c** show very similar glass transition temperatures, with **PE-6b** *T*_g_ = −54 °C and **PE-6c** *T*_g_ = −41 °C, and very similar depolymerization equilibria. To achieve fast recycling rates (TOF > 500 h^–1^), **PE-6c** requires 10× more catalyst (1:100) and temperatures above 150 °C, whereas **PE-6b** is efficiently depolymerized (TOF = 1900 h^–1^) using minimal catalyst (1:1000) at 90 °C. Overall, these linear free energy relationships should be used, in future, to predict recycling rates for new polymers using recycling (or polymerization) thermodynamic data. Alternatively, they can be used to predict recycling thermodynamics, for new polymers, using recycling rate data (assuming the same depolymerization mechanisms).

### Catalyzed Recycling to Separate Polymer Mixtures

Another important implication of the recycling kinetic-thermodynamic linear free energy relationship is that it provides scientific understanding to guide recycling of more complex polymer structures, e.g., polymer mixtures or blends. Using either the depolymerization thermodynamic or kinetic data, it should be possible to recommend how to sort or separate polymer mixtures by exploiting differences in depolymerization rates. Using the data, the appropriate conditions can be recommended which enable quantitative and selective chemical separation, by recycling the polymers to monomers and exploiting reactive distillation methods.

To test whether chemical recycling could be used to separate structurally similar polymers, a blend of **PE-6b** and **PE-7c** was prepared, with the Zn­(II) catalyst (1:1 polymer mixture by mass, 1:100 [Zn­(Oct)]_2_:[total polymer repeat unit]_0_). These two polymers show very similar thermal properties and solubilities—they would be expected to be very difficult/impossible to completely separate using conventional solvent extraction and/or precipitation methods. Using the experimentally determined transition state barriers (Δ*G*_d_^⧧^), the chemical recycling rate constants are easily determined for both **PE-6b** and **PE-7c** over the temperature range 90–190 °C (Table S25). Using the kinetic data, a two-step depolymerization process was proposed, in which recycling at 90 °C should selectively depolymerize **PE-6b** over **PE-7c**, with a calculated rate difference, *k*_d_ **^PE‑6b^**/*k*_d_ **^PE‑7c^** ≈ 4000. By increasing the recycling temperature to 190 °C, the remaining **PE-7c** should be effectively depolymerized. To test these calculations, the depolymerization reaction was investigated using the polymer mixtures and the TGA-IR apparatus: at 90 °C, the rapid depolymerization of *ca* 50% of the blend mass was observed (Figure 6a). IR spectroscopy confirmed the selective formation of only lactone **6b**, there were no signals for **7c** (Figure S80). The reaction was then heated to 190 °C and the remaining 50% of polymer rapidly depolymerized and the only product at this point was lactone **7c**, with speciation confirmed by IR spectroscopy. Next, the polymer recycling was scaled to 1 g using a laboratory distillation apparatus (Figure S81, Table S26). On heating the polymer film mixture at 90 °C, only lactone **6b** was recovered in 97% yield and 99% purity, as confirmed by ^1^H NMR spectroscopy and GC-MS (Figure 6b, Figures S82–83). The reaction flask was then heated at 190 °C, yielding pure lactone **7c**, again in excellent yield and selectivity (>97% yield, >98% purity, Figures S84–85). Inspired by the success of the bicomponent blend separation, the catalytic recycling was explored to separate a more complex, 3-component blend of elastomeric polyesters. A mixture of **PE-6b**: **PE-6c**: **PE-7c** was subjected to catalyzed chemical recycling (1:1:1 polymer mixture by mass, 1:100 [Zn­(Oct)]_2_:[total polymer repeat unit]_0_). It is emphasized that separation of these polymers through precipitation or extraction is not possible owing to their near-identical physical properties and solubilities. Once again, the recycling rate constants were used to propose conditions for effective recycling and mixture separation between 90 and 190 °C (Table S25). The catalyzed recycling was conducted at three different temperatures: 90, 120, and 190 °C, resulting in the selective recovery of lactones **6b**, **6c**, and **7c**, respectively as confirmed by IR spectroscopy (Figure S86). Finally, the three-polymer blend recycling was tested at the laboratory-scale (*ca* 1 g total mass), once again enabling isolation of **6b**, **6c** and **7c** as the major products at 90, 120, and 190 °C, respectively, as indicated by ^1^H NMR spectroscopy and GC–MS (Table S27, Figures S87–S92). These proof-of-concept recycling-separation experiments demonstrate the potential for catalyzed chemical recycling to monomer to efficiently sort/separate extremely similar polymers from mixtures. The method is particularly suited to efficient separations of structurally similar polymers; this area of science has been a long-standing challenge since polymer blends, multilayers and polymeric additives often require compatibility, and therefore similar chemistry, to deliver the best material performance.

**Figure 6:**
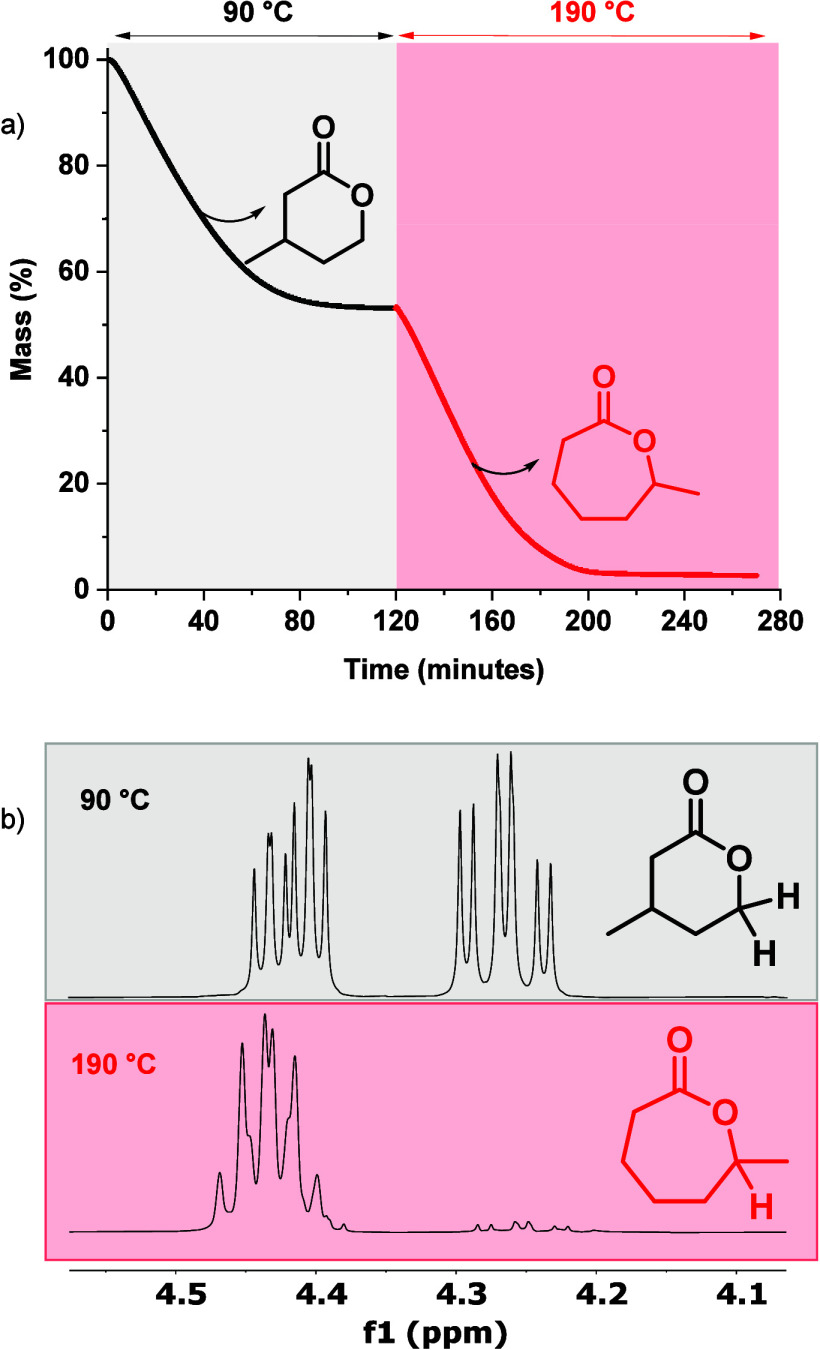
Catalyzed recycling to cyclic esters of mixtures of structurally similar polyesters. (a) Shows the data obtained for recycling of a two-component polyester mixture over time and at different (isothermal) temperatures. The polymers in the blend were **PE-6b** and **PE-7c**. The reaction conditions: [Zn­(Oct)_2_]_0_:[**PE-6b**]_0_:[**PE-7c**]_0_ = 1:50:48 (1:1 polymer, by mass). Stepwise isotherms at 90 °C (black) and 190 °C (red). (b) ^1^H NMR spectra of the recyclate formed in reactive distillation at 90 °C (top) and 190 °C (bottom) showing the selective formation of the two different lactones. For full ^1^H NMR spectra and GC, see Figures S82–S85.

## Conclusions

Quantitative polymer structure-depolymerization rate relationships were identified for the first time and used for low energy, efficient polymer chemical recycling to monomer processes. The polymer chemical recycling kinetic barriers were measured for a series of widely applied aliphatic polyesters and polycarbonates. All the polymers were efficiently and selectively recycled, using a commercial Zn­(II) catalyst, to their respective cyclic ester and carbonate monomers. These catalyzed polymer recycling processes applied conditions that minimize chemical and energy inputs and drive the depolymerization equilibrium. Using these conditions enabled quantitative and selective conversions to monomers in all cases, although with significant differences in overall rates. In all the experiments, neat polymer melts were recycled at low temperatures (90–190 °C), using low loadings of a commercial Zn­(II) catalyst and nitrogen gas flows to remove the monomers and drive the equilibrium. In evaluating the recycling rates, the series of polyesters and carbonates each had the same degree of polymerization and hydroxyl end-groups, enabling quantitative evaluation of kinetic influences from the repeat unit chemistry (6- or 7-membered, carbonate or ester), substituents and their positions. Recycling catalysis was shown to be first order with respect to catalyst and proposed to occur via a chain-end mechanism. Remarkably, the first example of a linear free energy relationship in recycling catalysis was uncovered. Systematic correlations (linear/exponential relationships) applied throughout all the materials between the depolymerization kinetic and thermodynamic barriers. The linear free energy relationship is fully rationalized by the depolymerization mechanism and underpins the structure-performance relationships occurring through the catalysis. In equivalent recycling catalyses, polyesters forming 6-membered rings were recycled 100× faster (or at lower temperature) than those forming 7-membered lactones and 25× faster than those forming 6-membered cyclic carbonates. The position of any (methyl) substituents affects rates with midchain sites enabling faster recycling than substituents at sites adjacent to acyl oxygen or carbonyl groups. The linear free energy relationship allows for proper selection of low-energy recycling conditions and minimized catalyst loading, resulting in fast recycling. It was also used to identify the appropriate temperatures to selectively separate structurally similar polymer mixtures by forming their monomers. The discovery of quantified polymer recycling kinetic-thermodynamic relationships is important for the design of future efficient and selective chemical recycling processes. The concepts and methods demonstrated in this paper using aliphatic polyesters and carbonates should be more broadly applicable to other polymers and mixtures. These recycling science kinetic-thermodynamic relationships should be explored for other polymer chemistries and materials classes.

## Supplementary Material



## Notes

Experimental materials and methods, polymer/monomer purity evaluation data, recycling of polyesters and polycarbonates, ^1^H NMR spectrum, GC-chromatograms, monomer properties, synthesis data, and data for depolymerization reactions (PDF)
