# Supplementary material for: The Science of Polymer Chemical Recycling Catalysis: Uncovering Kinetic and Thermodynamic Linear Free Energy Relationships
Source: J Am Chem Soc. 2025 Jun 23;147(26):22734–46. doi: 10.1021/jacs.5c04603 (PMC12232312; doi:10.1021/jacs.5c04603)
Supplement: Supplementary file 1 [file ja5c04603_si_001.pdf]

Supporting Information for:

The Science of Polymer Chemical Recycling Catalysis: Uncovering Kinetic and Thermodynamic Linear Free Energy Relationships

Thomas M. McGuire,[a] David Ning,[a] Antoine Buchard[b] and Charlotte K. Williams*[a]

[a] Department of Chemistry, Chemistry Research Laboratory, University of Oxford, 12 Mansfield Rd, Oxford, OX1 3TA, U.K

[b] Department of Chemistry, Green Chemistry Centre of Excellence, University of York YO10 5DD, U. K.

Corresponding Authors

Charlotte K. Williams: Email: charlotte.williams@chem.ox.ac.uk

Table of Contents

1. Materials 3
2. Methods 4
3. Monomer, polymer and catalyst properties 8
4. Additional information 11
^{1}H NMR spectra and GC- Chromatograms of isolated monomers 11
Depolymerization data 38
TGA-FTIR data 58
Eyring analysis 64
DFT 72
Linear-Free Energy Relationships 76
PE-6b and PE-7c Blend Recycling 78
PE-6b, PE-6c and PE-7c blend recycling 84
5. Polymer characterisation 91
6. Monomer/polymer equilibrium measurements 122
7. References 123

1. Materials

All experiments were carried out under N_2 using standard Schlenk/glovebox techniques unless otherwise stated. Anhydrous dichloromethane was purchased from Sigma-Aldrich and degassed by N_2 purge prior to use. THF and toluene were obtained from an SPS system, degassed by several freeze-pump-thaw cycles and stored over 3 Å molecular sieves under nitrogen. Zinc(II) 2-ethylhexanoate was purchased from Fluorochem and used as received. Tin(II) 2-ethylhexanoate ($Sn(Oct)_2$) was purchased from Sigma-Aldrich and used as received. 1,4-benzenedimethanol (BDM) was purchased from Sigma Aldrich.; Prior to use, BDM was recrystallised from anhydrous toluene. δ-Valerolactone (6a) was purchased from Alfa Aesar. 3-Methyl-δ-valerolactone (6b) was synthesised according to literature.[1] δ-Hexalactone (6b) was purchased from Sigma Aldrich. δ-Decalactone (6c) was purchased from Sigma Aldrich. ε-Caprolactone (7a) was purchased from Sigma Aldrich. 4-Methyl-ε-caprolactone (7b) was synthesised according to literature.[2]. 6-Methyl-ε-caprolactone (7d) was synthesised according to literature.[3] ε-Decalactone (7d) was purchased from Sigma Aldrich. Butylene carbonate (6c') was synthesised according to literature.[4] Prior to use, all lactones and 6c' were dried over CaH_2 and fractionally distilled under reduced pressure. *rac*-Lactide was purchased from Sigma Aldrich. Prior to use, *rac*-lactide was recrystallised from anhydrous toluene before repeated (x3) sublimation. Trimethylene carbonate (6a') was purchased from Tokyo chemical industry. 2,2-dimethyltrimethylenecarbonate (6b') was synthesised according to literature.[4] Prior to use, 6a' and 6b' were twice recrystallised from anhydrous diethyl ether. All monomers were stored in a glovebox.

Size exclusion chromatography (SEC) was carried out on a Shimadzu LC-20AD instrument using two PSS SDV linear M columns in series, with a $CHCl_3$ eluent. Measurements were conducted at 30 °C with a flow rate of 1 mL/min. Samples were detected with a differential refractive index (RI) detector. Number-average molar mass ($M_{n,SEC}$) and dispersities ($Đ_M$ (M_w/M_n)) were calculated against a polystyrene calibration (molar mass range 500 – 1000000 g mol^{-1}) . The polymer samples were dissolved in HPLC-grade THF at a concentration of *ca* 10 mg/mL and filtered through a 0.2 µm microfilter prior to analysis

Differential scanning calorimetry (DSC) was performed using a TA discovery 25 auto. Experiments were performed under N_2 flow (50 mL/min) using aluminium TZERO pans. Samples (2–5 mg) were equilibrated at 40 °C, then heated, at a rate of 20 °C/min, to 200 °C and held at 200 °C, for 5 minutes. The sample was then cooled, at a rate of 20 °C/min, to –80 °C and held at –80 °C, for 5 minutes. The sample was then heated at a rate of 10 °C/min, to 200 °C and cooled, at a rate 10 °C/min, to –80 °C for 2 successive cycles. Thermal data is reported from the second heating cycle.

Thermal gravimetric analysis – Fourier Transform Infrared (TGA-FTIR) were collected on a TGA5500 System (TA Instruments), equipped with a Nicolet iS20 FTIR spectrometer (Thermo Scientific Instruments). The TGA temperature was calibrated against standards of alumel (Curie Point = 153.0 °C), nickel (Curie point = 358.2 °C), nickel-83:cobalt-17 (Curie point = 554.4 °C) and nickel-63:cobalt-37 (Curie point = 746.4). The TGA mass was calibrated against standards of 100.0000 mg and 1000.0000 mg. Detailed procedures for the TGA analysis are given in the protocol section. The FTIR spectrometer was equipped with a KBr/Ge beamsplitter, fast-recovery deuterated triglycine sulfate KBr detector and solid-state diode laser. FTIR Spectra were recorded, between 400–4000 cm^{-1} , with 10 scans per spectrum, at a resolution of 8 cm^{-1} .

NMR spectra were obtained using a Bruker AVIII HD nanobay NMR spectrometer. Coupling constants are given in Hertz. Selectivities were determined by ^1H NMR spectroscopy.

GC-MS spectra were recorded on an Agilent 7820A, equipped with a HP5-MS ultra inert column (30 m length, 0.25 mm internal diameter, 0.25 µm film thickness), a 5977B single quad mass spectrometer, a liquid injection autosampler and He carrier gas. Data was processed using MassHunter software. Samples of 5 mg/mL were prepared in dichloromethane with 1µL injected into the instrument. Samples were loaded on to the column in 1:100 sample:solvent splitter ratio and injection port temperature of 300 °C.The column was pressurised, at 9.1 PSI, with a column flow of 1.2 mL/min and total flow of 22.12 mL/min. Following equilibration of the column oven at 40 °C for 1 minute, the temperature was increased from 40 °C to 300 °C, at a rate of 10 °C/min, and held at 300 °C, for 3 minutes. The MS source and quadrupole temperature was 230 °C and 150 °C, respectively.

Turnover Frequency (TOF): calculations were performed at 30% conversion of the polymer, using the formula, moles of polymer converted/moles of catalyst added/time.

Error analysis all reactions were repeated at least in triplicate. For a given reaction, errors were taken as the standard deviation of the mean and a percent error was calculated as standard deviation/average of run *100. The average percent error was calculated across each polymer series. If the percent error for a given reaction was very small (i.e. smaller than 2 standard deviations away from the average percent error), the error for that run was instead calculated as the average percent error of the series.

2. Methods

N.B. all polymer concentrations are calculated as concentration of the repeat unit. For PLLA, concentrations are calculated as the concentration the lactic acid repeat unit.

For details on catalyst and monomer concentrations in polymer synthesis, see table S2.

2.1 Polymerization of monomers using diphenyl phosphate catalyst

In a nitrogen-filled glovebox, delta valerolactone (1.00 g, 10 mmol, 100 equiv.) benzene dimethanol (13.8 mg, 0.1 mmol, 1 equiv.) and diphenyl phosphate (DPP, 25.0 mg, 0.1 mmol, 1 equiv.) were added to a polymerization vial. After stirring for 3 h, the reaction mixture had solidified. The reaction was quenched with excess triethyl amine (~40 µL). The polymer was precipitated in methanol (~30 mL), centrifuged (3900 rpm, 4 min), then the supernatant was decanted. The solid obtained was redissolved in DCM (~5 mL), then the precipitation in methanol was repeated a further two times. The product was dried under vacuum overnight, then analyzed by ^{1}H NMR, SEC, DSC, and TGA.

2.2 Polymerization of monomers using 1,5,7-Triazabicyclo[4.4.0]dec-5-ene catalyst

In a nitrogen-filled glovebox, trimethylene carbonate (1.02 g, 10 mmol, 100 equiv.) benzene dimethanol (13.8 mg, 0.1 mmol, 1 equiv.) and 1,5,7-Triazabicyclo[4.4.0]dec-5-ene (TBD, 13.9 mg, 0.1 mmol, 1 equiv.) were added to a polymerization vial and dissolved in THF (10 mL). After stirring for 10 minutes, the reaction mixture was quenched with excess benzoic acid in THF (0.5 M, ~0.5 mL). The polymer was precipitated in cold diethyl ether (0 °C, ~30 mL), centrifuged (3900 rpm, 4 min), then the supernatant was decanted. The solid obtained was redissolved in DCM (~5 mL), then the precipitation in diethyl ether was repeated a further two times. The product was dried under vacuum overnight, then analyzed by ^{1}H NMR, SEC, DSC, and TGA

. 2.3 Polymerization of monomers uing ZnMg heterobimetallic catalyst.

In a nitrogen-filled glovebox, epsilon decalactone (1.50 g, 8.81 mmol, 750 equiv.) benzene dimethanol (12.8 mg, 0.0881 mmol, 7.5 equiv.) and ZnMg (11.6 mg, 0.0117 mmol, 1 equiv.) were added to a polymerization vial and dissolved in toluene. After stirring for 30 minutes, the reaction mixture had solidified. The reaction was quenched with excess with excess benzoic acid in THF (0.5 M, ~0.5 mL). The polymer was precipitated in methanol (~30 mL), centrifuged (3900 rpm, 4 min), then the supernatant was decanted. The solid obtained was redissolved in DCM (~5 mL), then the precipitation in methanol was repeated a further two times. The product was dried under vacuum overnight, then analyzed by ^{1}H NMR, SEC, DSC, and TGA.

2.4 Evaluation of Kinetics of Solid-State Chemical Recycling to Monomer

Exemplary method for PE-6a

In a nitrogen-filled glovebox, stock solutions of PE-6a (1.00 M, 100 mg of PE-6a in 1.00 mL of THF) and $Zn(Oct)_2$ (1.00 x 10^{-2} M,10.6 mg of $Zn(Oct)_2$ in 3.00 mL of THF) were prepared. The PE-6a stock solution (100 µL, 0.1 mmol, 1000.0 equiv.) was added to a vial containing $Zn(Oct)_2$ (10 µL, 1.00 x 10^{-4} mmol, 1.0 equiv.) . The $Zn(Oct)_2$-PE-6a solution was thoroughly mixed before being dropcast (*ca* 3 drops, total mass once dry 1 – 2 mg) onto Platinum TGA crucibles. The solvent was allowed to evaporate before the crucible was loaded into the TGA instrument for monitored solid-state depolymerization.

The polymer samples were analyzed using the following common method:

N_2 flow of 25.0 mL min^{-1}

Isotherm at 130 °C for 120 minutes

The catalyst loading, reaction temperature and length of the isotherm were varied as required.

To account for any residual solvent loss from the polymer films, which will also be detected as a mass loss by TGA, data from the first 0.5 minutes of the run was removed from the analysis. The mass at 0.5 minutes was then taken as the polymer initial mass ($mass_0$) and used to calculate the change in mass of the sample using the following formula:

$$\boldsymbol{mass\ fraction} = \frac{\boldsymbol{mass_t}}{\boldsymbol{mass_0}}$$

Equation S1

Where $mass_t$ = mass at time t.

Conversion for the reaction was calculated as:

$$\boldsymbol{Conversion} = \mathbf{1} - \boldsymbol{mass\ fraction}$$

Equation S2

For Eyring analysis, isotherms were held at 5 different temperatures between 90 – 190 °C (polymer dependent). Each isotherm was repeated in triplicate.

Based on mechanistic studies, the depolymerization was shown to proceed via chain-end backbiting, Therefore, the depolymerization rate law (variation of fraction conversion with time; s^{-1}) can be expressed as:

$$\boldsymbol{rate} = \boldsymbol{k_d}\,[\boldsymbol{Zn(OR)_2}]$$

Equation S3

Where k_d is the depolymerization rate constant and [$Zn(OR)_2$] is the concentration of *in-situ*-formed zinc polymer alkoxide. The depolymerization was shown to exhibit a first order dependence on $Zn(Oct)_2$ and $Zn(Oct)_2$ is assumed to activate 2 x chains per metal. Therefore the rate equation can be expressed as:

$$\boldsymbol{rate} = \boldsymbol{k_d}\ \mathbf{2}\,[\boldsymbol{Zn(Oct)_2}]$$

Equation S4

To fit the mass *vs* time data, as expressed in equation S1, a sigmoidal function (named logistic 1 function, in Origin 2023 software package) was fitted directly to the data:

$$\boldsymbol{y} = \frac{\boldsymbol{a}}{\mathbf{1} + \boldsymbol{e}^{-\boldsymbol{k}(\boldsymbol{x}-\boldsymbol{x_c})}}$$

Equation S5

Where y = conversion, x = time. The parameters k (growth constant), a (final conversion) and xc (time at maximum of the fit) are determined from the fit of the data, as indicated in Fig. S1.

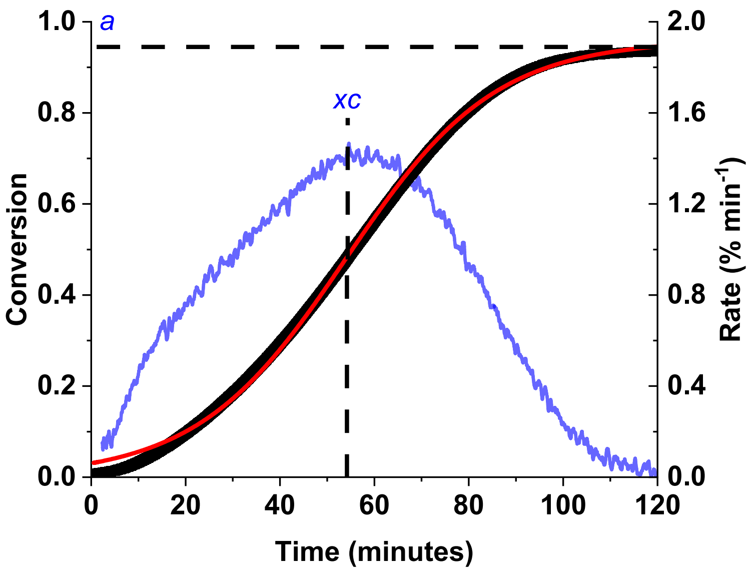

Figure S1. Example data for recycling of polyesters and polycarbonates showing conversion vs time (black curve), measured rate (blue curve), fitted logistic curve ($y = \frac{a}{1+e^{-k(x-x_c)}}$, red curve), a and xc parameters. Fits for all polymers and temperatures are shown in Fig. S34, 37, 40 – 50

The rate of the reaction at any point of the fit can be determined by taking the derivative of the Equation S5:

$$rate = \frac{dy}{dx} = \frac{kae^{-k(x-x_c)}}{(1+e^{-k(x-x_c)})^2}$$

Equation S6

k_{obs} was taken as the maximum rate of the fit, i.e., where x = xc:

$$k_{obs} = \frac{dy}{dx}\,(\max of\ fit) = \frac{ka}{4}$$

Equation S7

From experiments conducted with PE-7b, k_{obs} was found to have a linear dependence on $[Zn(Oct)_2]_0$:

$$k_{obs} = k'_d[Zn(Oct)_2]_0$$

Equation S8

Assuming all polymers follow the same rate law, k_d can be approximated as:

$$k_d = \frac{k_{obs}}{2\,[Zn(Oct)_2]_0}$$

Equation S9

2.5 Larger Scale Depolymerization of Polyester to determine Lactone Selectivity

In a nitrogen-filled glovebox, a stock solution of $Zn(Oct)_2$ (1.00 x 10 $^{-2}$ M, 10.6 mg of $Zn(Oct)_2$ in 3.00 mL of THF) was prepared. The $Zn(Oct)_2$ solution (1 mL, 1.00 x 10 $^{-2}$ mmol, 1.0 equiv.) was added to a Schlenk containing PE-6a (1.00 g, 9.99 mmol, 1000.0 equiv.) The PE-6a and $Zn(Oct)_2$ were stirred until the polymer fully dissolved, before the solvent was removed *in-vacuuo*. A short-path distillation was connected to the Schlenk and the reaction mixture was heated to 130 °C with magnetic stirring under vacuum (0.1–1 mbar). After 2 h, a clear oil was collected which was determined to be valerolactone (990 mg, 9.69 mmol, 97% yield)

N.B. the depolymerization may also be performed without prior dissolution of the polymer/catalyst mixture

2.6 Depolymerization of polymer blends

In a nitrogen-filled glovebox, PE-6b (451 mg, 3.95 mmol, 50 equiv.) and PE-7c (451 mg, 3.52 mmol, 48 equiv.) were dissolved in a THF solution (*ca* 1 mL) and added to a round-bottomed flask containing $Zn(Oct)_2$ (28 mg, 0.079 mmol, 1 equiv.). The solvent was removed *in-vacuuo* and the flask was loaded onto a Kugelrohr apparatus (Fig. S81). To collect the first fraction, the flask was heated to 90 °C under 15 – 20 mbar pressure. After 2 h, a clear oil was collected which was determined to be 6b (436 mg, 3.81 mmol, 97% yield). To collect the second fraction, the reaction flask was heated to 190 °C under 1 – 8 mbar pressure. After 2 h, a clear oil was collected which was determined to be 7a (438 mg, 3.42 mmol, 97% yield). All samples were characterised by ^{1}H NMR spectroscopy and GC-MS.

2.7 Integrated Friedman Isoconversional analysis

As outlined in section 2.4, depolymerization of PVL catalysed by $Zn(Oct)_2$ (1:1000) was performed at 100, 110, 120, 130 and 140 °C in triplicate and the mass(%) of conversion was calcualated for each reaction. For each temperature, the time taken for the depolymerization to reach 10, 20, 30, 40, 50, 60, 70 and 80% conversion was recored. The ln time vs 1/Temperature was plotted and the activation parameter, E_a, extracted from the gradient of the graph using the integrated Friedman equation:

$$\ln t_{conv} = \ln\left(\frac{y}{A}\right) + \frac{E_a}{RT}$$

Equation S10

2.8 Determination of thermodynamic parameters of bulk polymerization

In a nitrogen-filled glovebox, 6c’ (1.14 g, 10 mmol, 100 equiv.) was added to a vial containing $Sn(Oct)_2$ (40.1 mg, 0.1 mmol, 1 equiv.) and benzyl alcohol (10.8 mg, 0.1 mmol, 1 equiv.). The reaction mixture was transferred to a DSC pan and heated until equilibrium was reached. The pan was then pierced and soaked in deuterated chloroform containing benzoic acid (quenching agent) and mesitylene (NMR

standard). The monomer conversion and equilibrium concentration was determined using ^1H NMR spectroscopy.

3. Monomer, polymer and catalyst properties

Table S1. Monomer properties for bulk polymerization

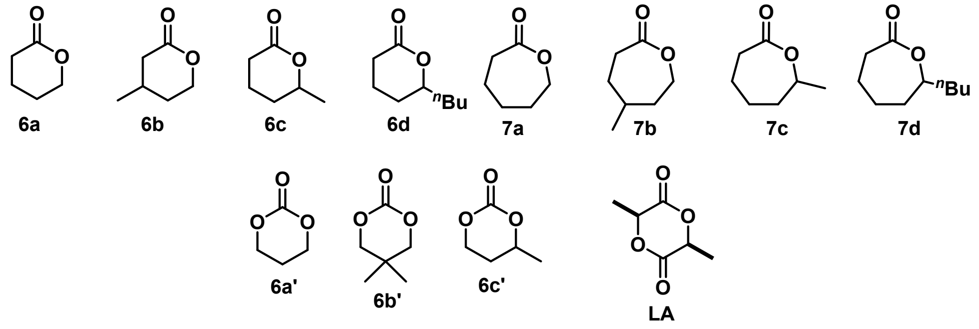

Mon.	[bulk] [a] (M)	$\Delta H_p°$ (kJ mol^{-1})	$\Delta S_p°$ 1M (J mol^{-1})	ΔS_p bulk (J mol^{-1})[b]	$\Delta G_d^{130°C}$ (kJ mol^{-1})[c]	K_d 130 °C[d]	Ref
6a[e]	9.99	-27.4	-65.0	-45.9	8.9	0.070	5
6b	8.76	-13.8	-46.0	-28.0	2.5	0.47	1
6c	8.76	-19.3	-62.2	-44.2	1.5	0.64	1
6d	5.87	-18.0	-57.0	-42.3	1.0	0.75	1
7a	8.76	-13.9	-10.4	+7.6	17.0	0.0063	6
7b	7.79	-18.5	-23.5	-6.4	15.9	0.0087	Fig. S140
7c	7.79	-21.0	-18.8	-1.7	19.3	0.0023	Fig. S141 [f]
7d	5.87	-25.4	-29.9	-15.2	20.3	0.0032	Fig. S142
6a’	9.79	n.d.	n.d.	n.d.	n.d.	n.d.	-
6b’	7.68	-25.0	-51.8	-34.8	11.0	0.038	7
6c’	8.61	-17.2	-40.8	-22.9	7.9	0.093	7
LA[g]	13.8	-29.1	-43.8	-22.0	20.2	0.0024	8

All polymerization parameters are given for bulk monomer polymerization, where the monomer state is liquid (l) and the polymer state is amorphous (a), unless otherwise stated.[a] bulk concentration of polymer calculated as [moles of monomer repeat unit]/[volume of polymer] assuming density of all polymers = 1.0 g/mL.[b] Calculated according to method in [9]: ΔS_p bulk = $\Delta S_p°$ 1M + R* ln ([polymer bulk]$_0$).[c] $\Delta G_d^{130°C} = -\Delta G_p^{130°C}$. $\Delta G_p^{130°C} = \Delta H_p° - 403.14 * \Delta S_p°$ bulk. [d] *exp*^ ($\Delta G_d^{130°C}$/R*403.14).[e] monomer state = *l,* polymer state = crystalline (c) .[f]determined from monomer containing 5 mol% α-methylcaprolactone. [g] bulk concentration determined from lactic acid repeat unit, M_w = 72.04 g mol^{-1}.

Table S2. Synthesis data for polyesters and polycarbonates

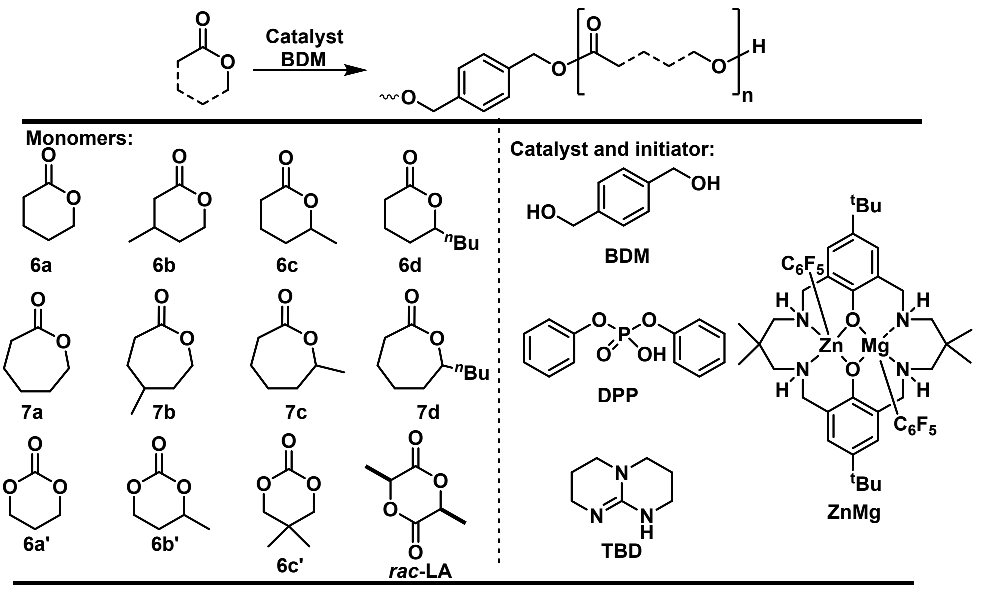

Polymer	$[cat]_0$: $[BDM]_0$: $[Mon]_0$	Cat.	Temp. (°C)	$[Mon]_0$ (M)	$M_{n,SEC}$ [a] [$Đ_M$] (g mol^{-1})	DP_{NMR} [b]	T_g [c] (°C)	T_m[d] (°C)	T_d[e] (°C)
PE-6a	1:1:100	DPP	Rt	bulk	17500 [1.09]	104	-55	53	324
PE-6b	1:1:100	DPP	Rt	bulk	23100 [1.10]	125	-54	-	320
PE-6c	1:1:125	DPP	60	bulk	21000 [1.10]	106	-41	(54)	315
PE-6d	1:1:125	DPP	60	bulk	18300 [1.22]	107	-61	-	326
PE-7a	1:1:100	DPP	60	bulk	24900 [1.39]	113	-62	54	383
PE-7b	1:1:100	DPP	Rt	bulk	22800 [1.39]	111	-61	-	373
PE-7c	1:1:100	DPP	60	bulk	21100 [1.06]	93	-46	-	328
PE-7d	1:7.5:750	ZnMg	80	3.4 M[f]	30400 [1.29]	109	-53	-	332
PC-6a	1:1:100	TBD	Rt	2.0 M[g]	16700 [1.70]	121	-20	-	222
PC-6b	1:1:100	TBD	Rt	2.0 M[g]	22200 [1.20]	108	28	106	227
PC-6c	1:1:100	TBD	Rt	1.0 M[g]	13600 [1.26]	104	-3	-	209
rac-PLA (-)	1:15:1500	ZnMg	Rt	2.0 M[f]	27000 [1.35]	105	53	(60)	303

[a] See Figs. S93 – 104. Calculated by SEC relative to polystyrene standards in tetrahydrofuran (THF) eluent; $Đ_M = M_w/M_n$. [b] See Figs. S105 – 116. Determined by ^{1}H NMR spectroscopy ($CDCl_3$) by relative integration of benzyl protons of BDM initiator (δ = 5.09 ppm) vs ester moiety (δ = 5.0 – 3.8 ppm) in isolated polymers. [c] See Figs. S117 – 128. T_g = glass transition reported from second heating cycles in DSC. [d] See Figs. S117 – 128. T_m = melt transition reported from second heating cycles in DSC. Bracketed values indicated melt transition observed only on first heating cycle.[e] See Figs. S129 – 139. T_d = degradation onset measured under N_2 flow (25 ml min^{-1}) under dynamic heating (10 °C min^{-1}). [f] in toluene solvent.[g] in THF solvent

Table S3. Data for depolymerization reactions carried out with laboratory glassware under vacuum

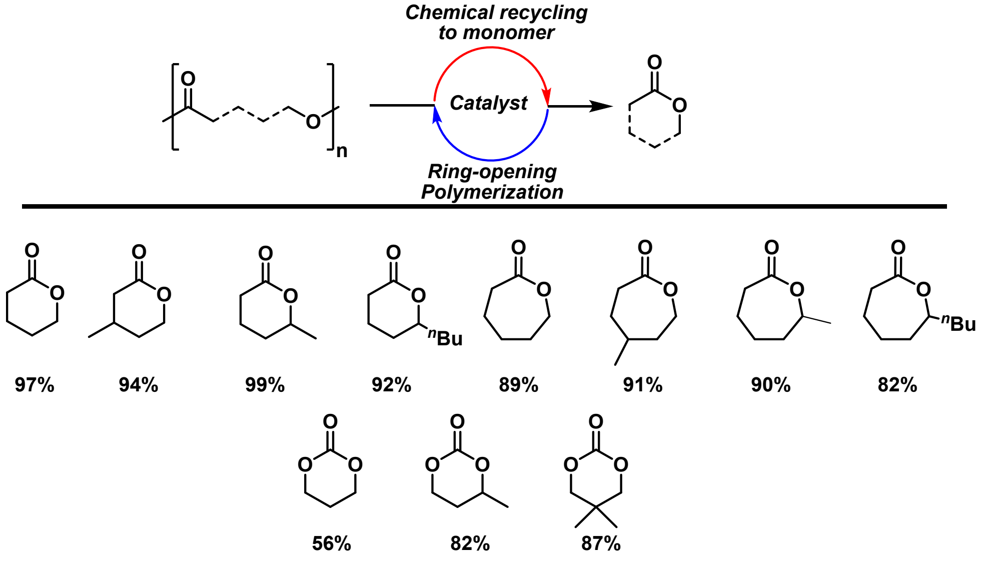

Polymer	$[cat]_0$:<br>$[Polymer]_0$	Temp. (°C)	Time (h)[a]	Yield (%)[b]	Selectivity (%) [c]
PE-6a	1: 1000	130	2	97	>99
PE-6b	1: 1000	130	1	94	>99
PE-6c	1: 100	160	2	99	>99
PE-6d	1: 100	160	2	92	>99
PE-7a	1: 100	160	6	89	>99
PE-7b	1: 100	160	6	91	>99
PE-7c	1: 100	190	6	90	>99
PE-7d	1: 100	190	14	82	99[d]
PC-6a[e]	1: 160	160	6	56[f]	98
PC-6b[e]	1: 1000	160	5	82	>99
PC-6c	1: 160	160	6	87	>99
rac-PLA[e]	1: 100	160	6	74	99[g]

Catalyst = $Zn(Oct)_2$. Reactions were performed by solvent casting (THF) the catalyst:polymer mixture into a reaction flask with distillation kit attached. The solvent was removed under vacuum at room temperature before the reaction mixture was heated under vacuum.[a] All times are approximate.[b] Yield = moles of recovered product/moles of polymer. [c] Selectivity for monomer, determined by GC-MS.[d] High selectivity was observed by GC-MS but trace impurities were detected by ^{1}H NMR spectroscopy which were difficult to quantify.[e] sublimation kit used to collect solid monomer.[f] Additional analysis of of the reaction flask *via* ^{1}H NMR spectroscopy suggests the depolymerization is selective for formation of cyclic carbonate monomer with no evidence for formation of larger heterocycles (Fig. S21).[g] the 20% *meso*-lactide formed

4. Additional information

^{1}H NMR spectra and GC- Chromatograms of isolated monomers

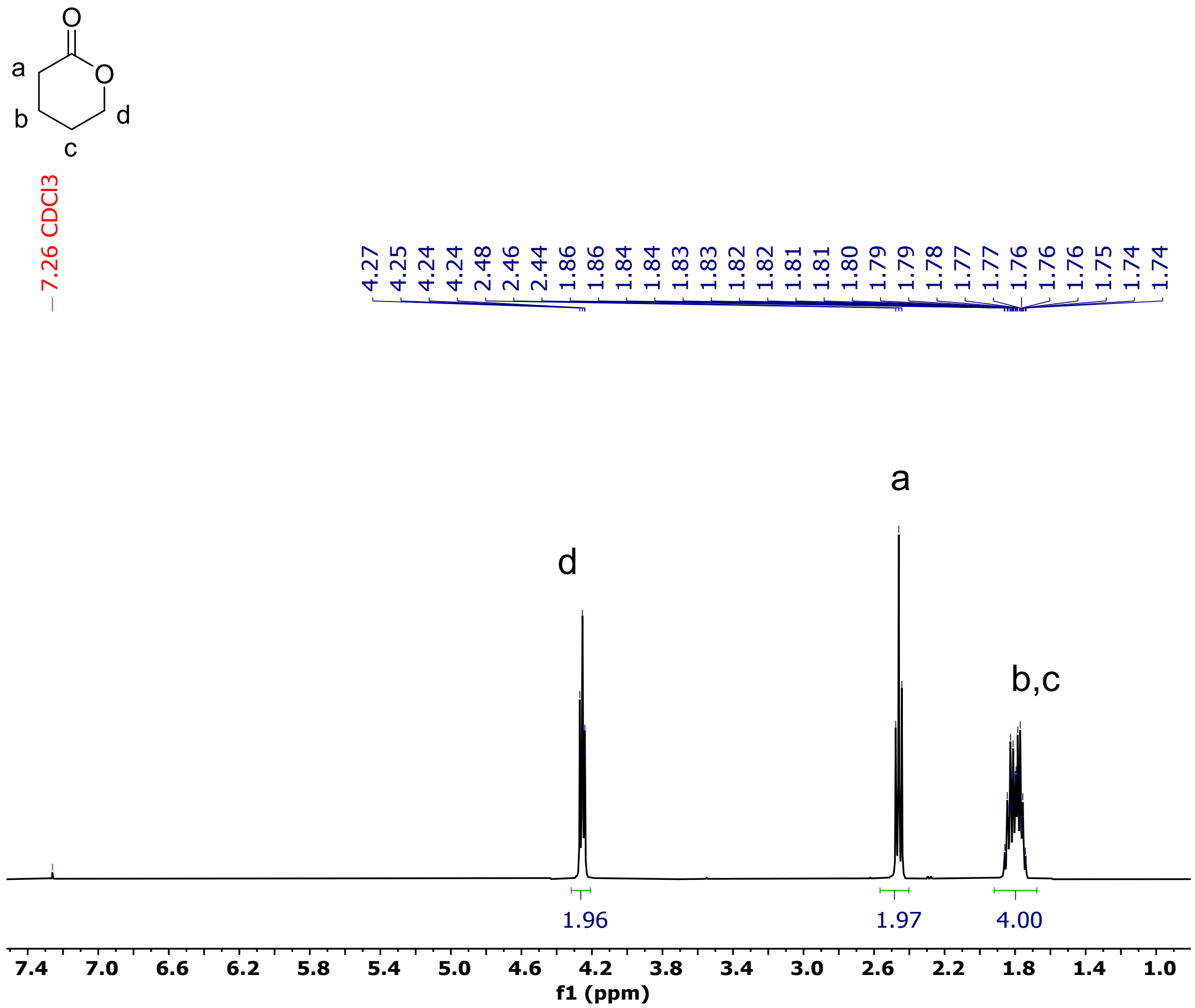

Figure S2. ^{1}H NMR spectrum (400 MHz, $CDCl_3$, 298K) of 6a isolated from recycling of PE-6a ($[Zn(Oct)_2]_0$:$[PE\text{-}6a]_0$ 1:1000, 130 °C)

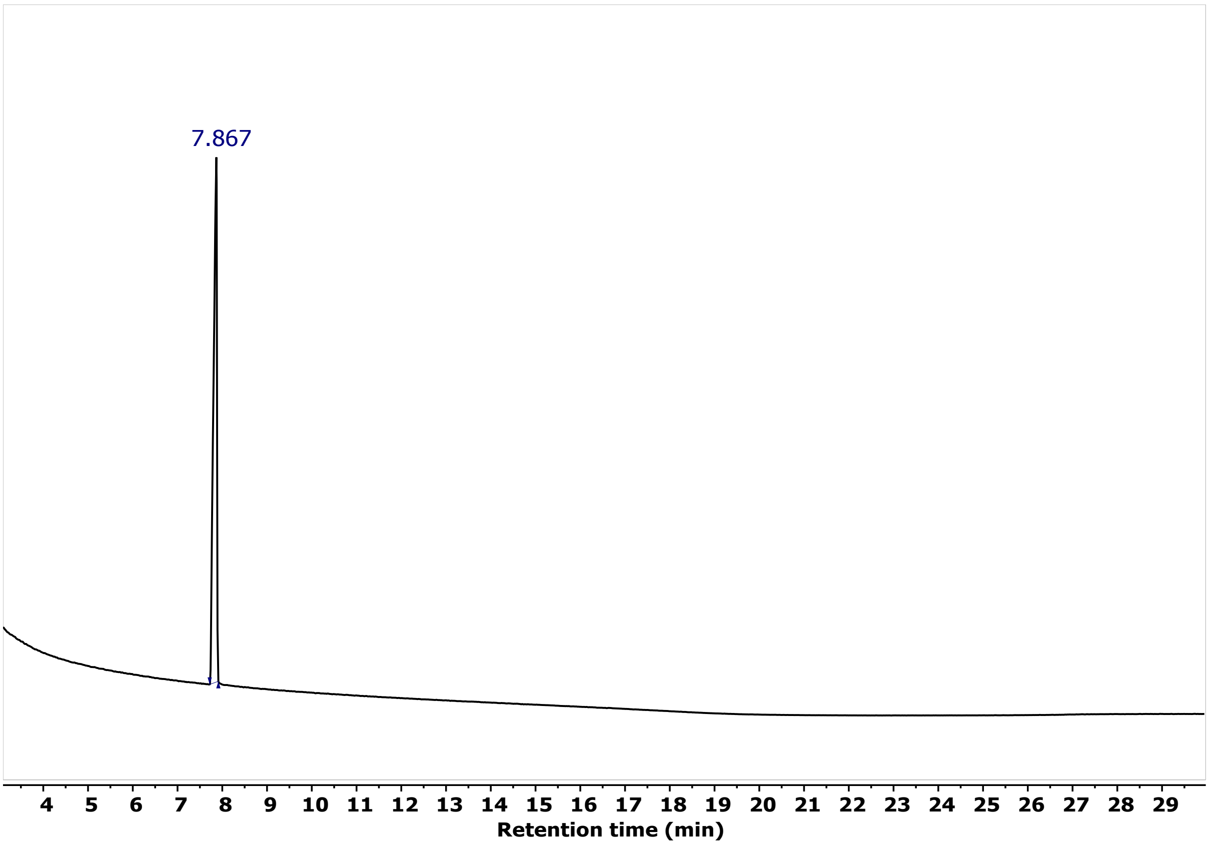

Figure S3. GC of of 6a isolated from recycling of PE-6a ($[Zn(Oct)_2]_0$:$[PE\text{-}6a]_0$ 1:1000, 130 °C); pure sample of 6a elutes at 7.87 minutes.

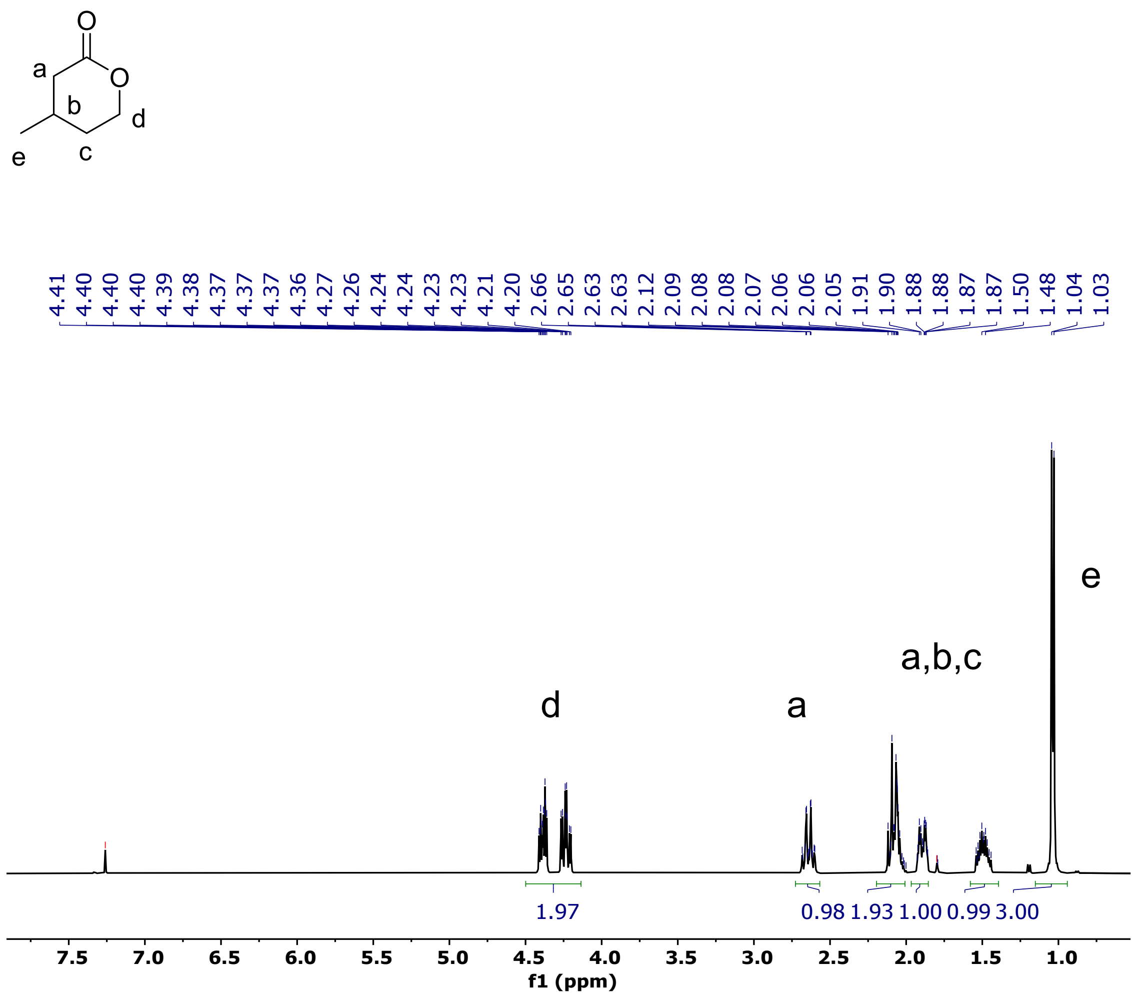

Figure S4. ^{1}H **NMR spectrum (400 MHz,** $CDCl_3$**, 298K) of 6b isolated from recycling of PE-6b (**$[Zn(Oct)_2]_0$**:**$[PE\text{-}6b]_0$ **1:1000, 130 °C).**

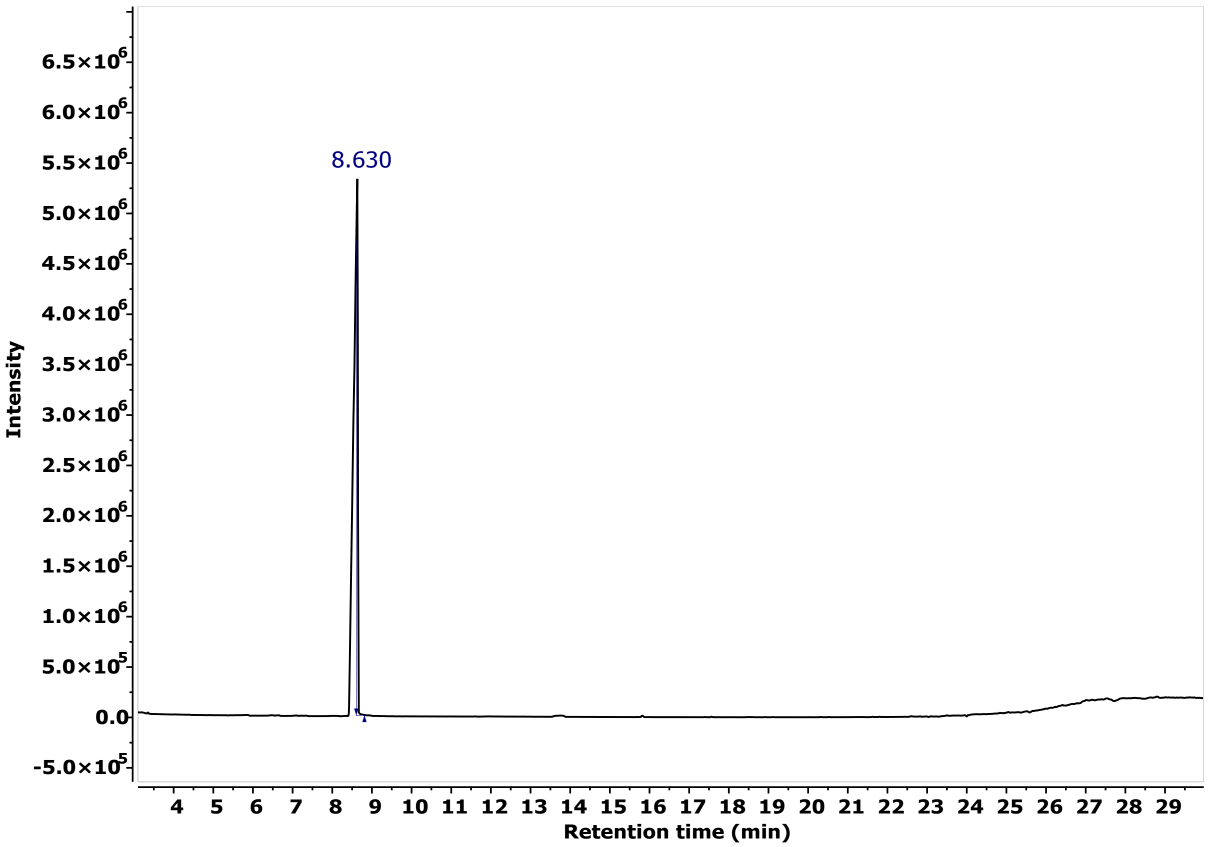

Figure S5. GC of of 6b isolated from recycling of PE-6b ($[Zn(Oct)_2]_0$:$[PE\text{-}6b]_0$ 1:1000, 130 °C). Pure sample of 6b elutes at 8.63 minutes.

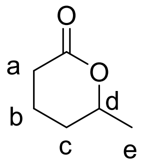

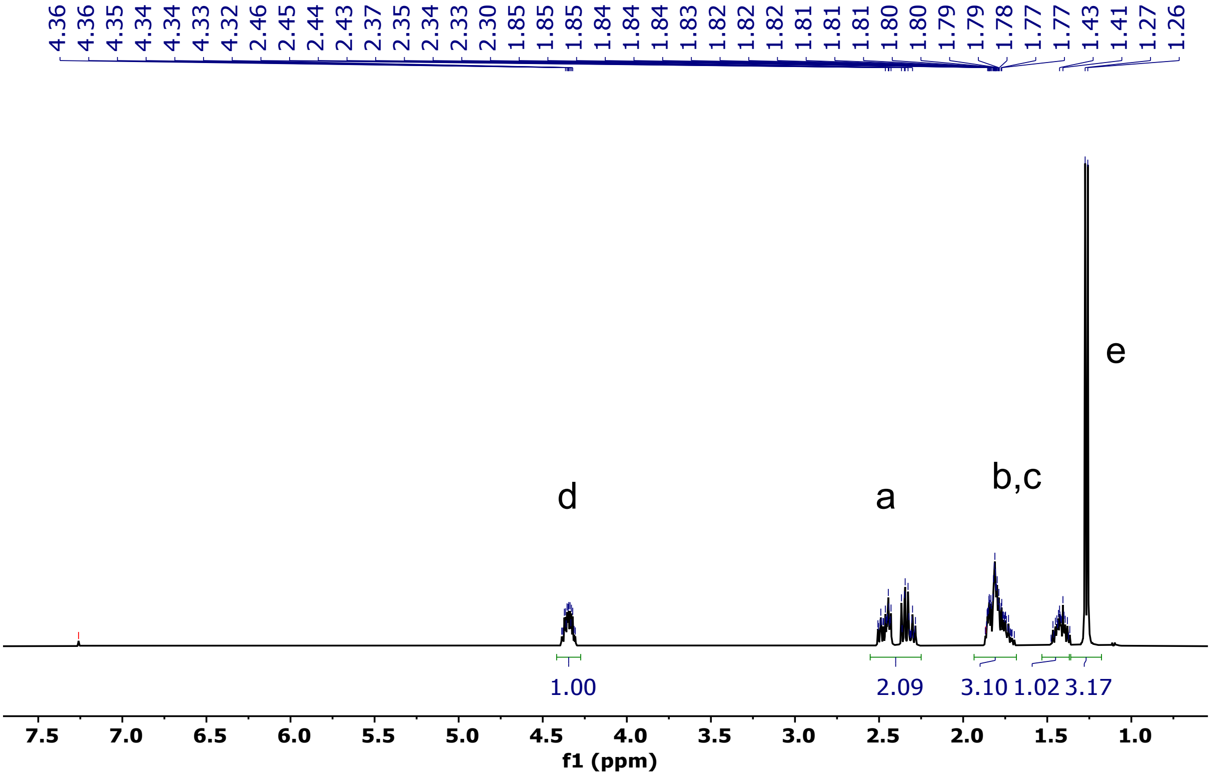

Figure S6. ^{1}H NMR spectrum (400 MHz, $CDCl_3$, 298K) of 6c isolated from recycling of PE-6c ($[Zn(Oct)_2]_0$:$[PE\text{-}6c]_0$ 1:100, 160 °C)

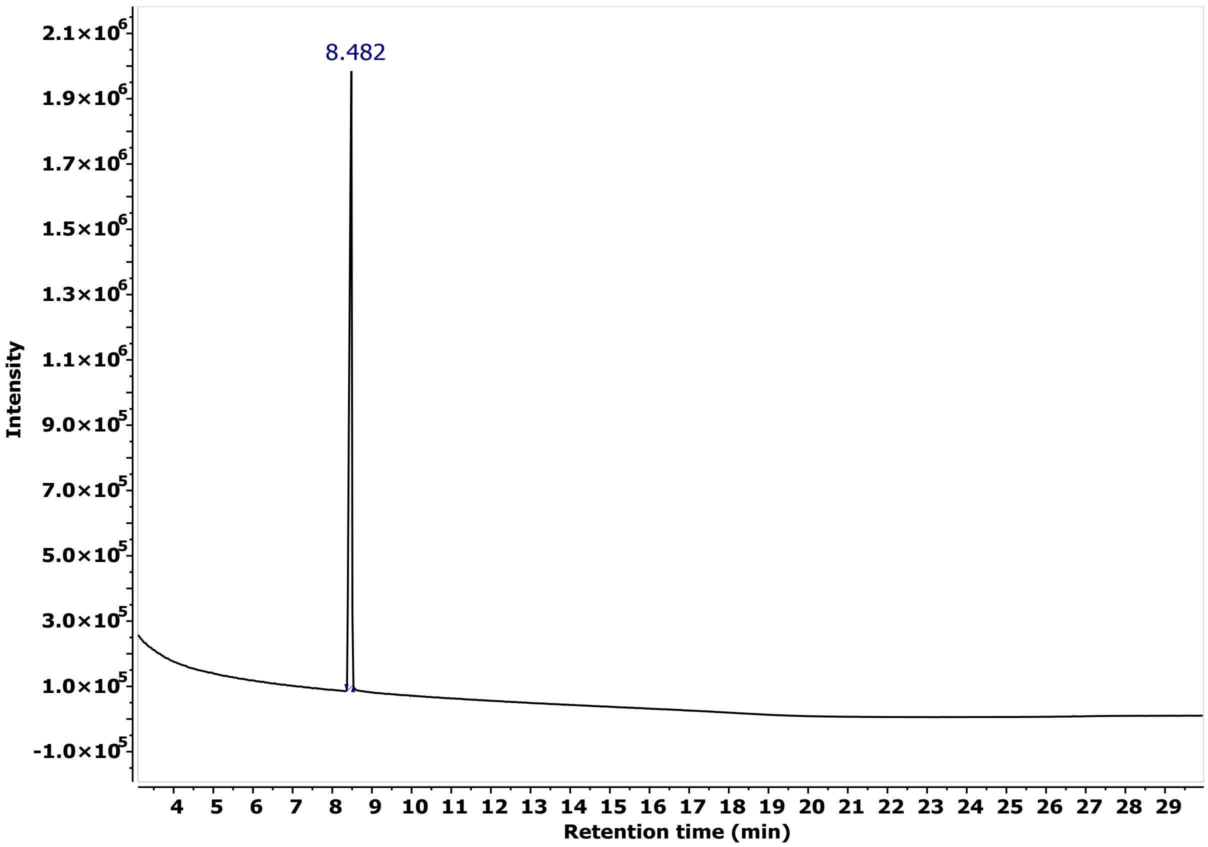

Figure S7. GC of 6c isolated from recycling of of PE-6c ($[Zn(Oct)_2]_0$:$[PE\text{-}6c]_0$ 1:100, 160 °C). Pure sample of 6c elutes at 8.48 minutes.

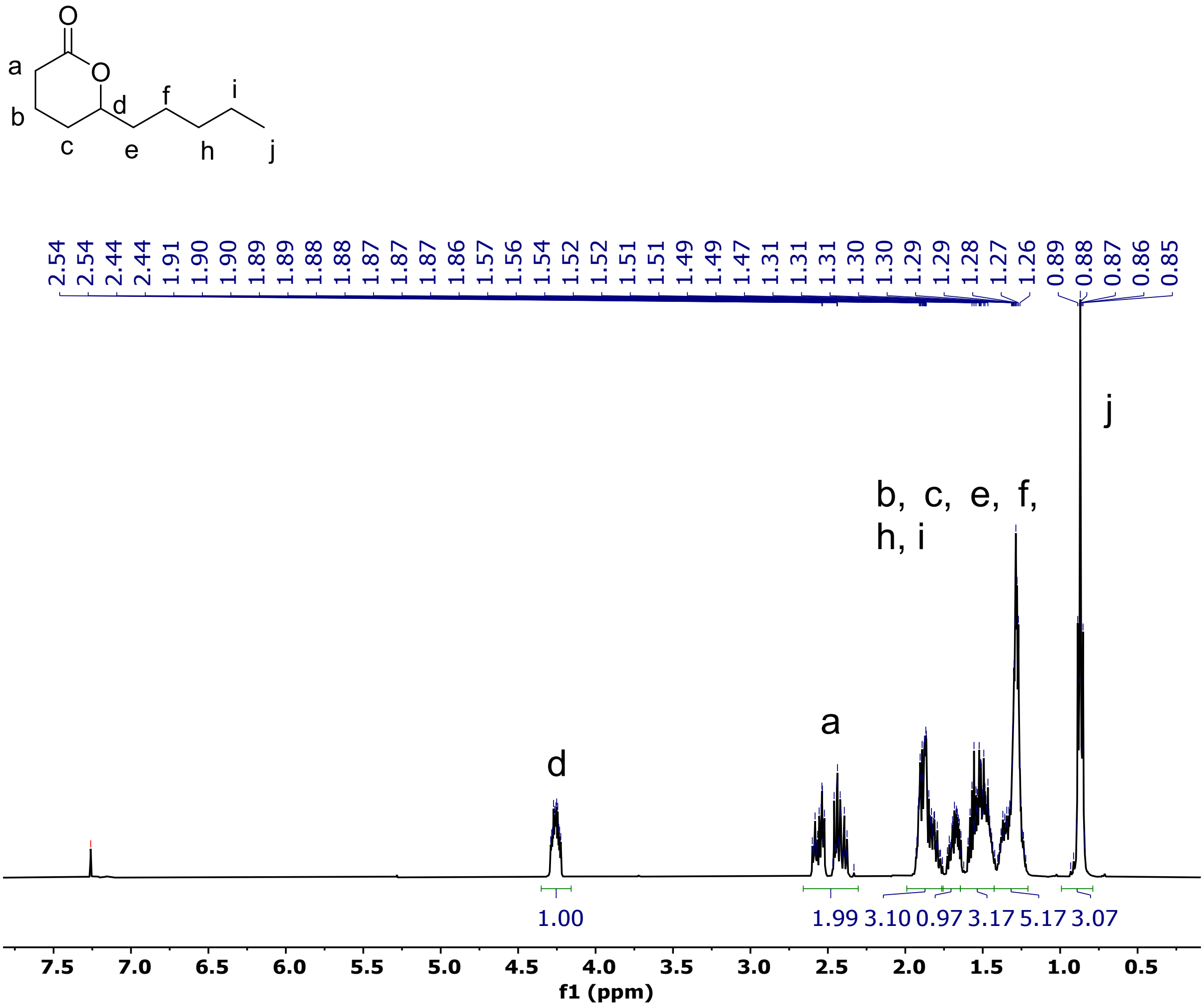

Figure S8. ^{1}H NMR spectrum (400 MHz, $CDCl_3$, 298K) of 6c isolated from recycling of PE-6d ($[Zn(Oct)_2]_0$:$[PE\text{-}6d]_0$ 1:100, 160 °C).

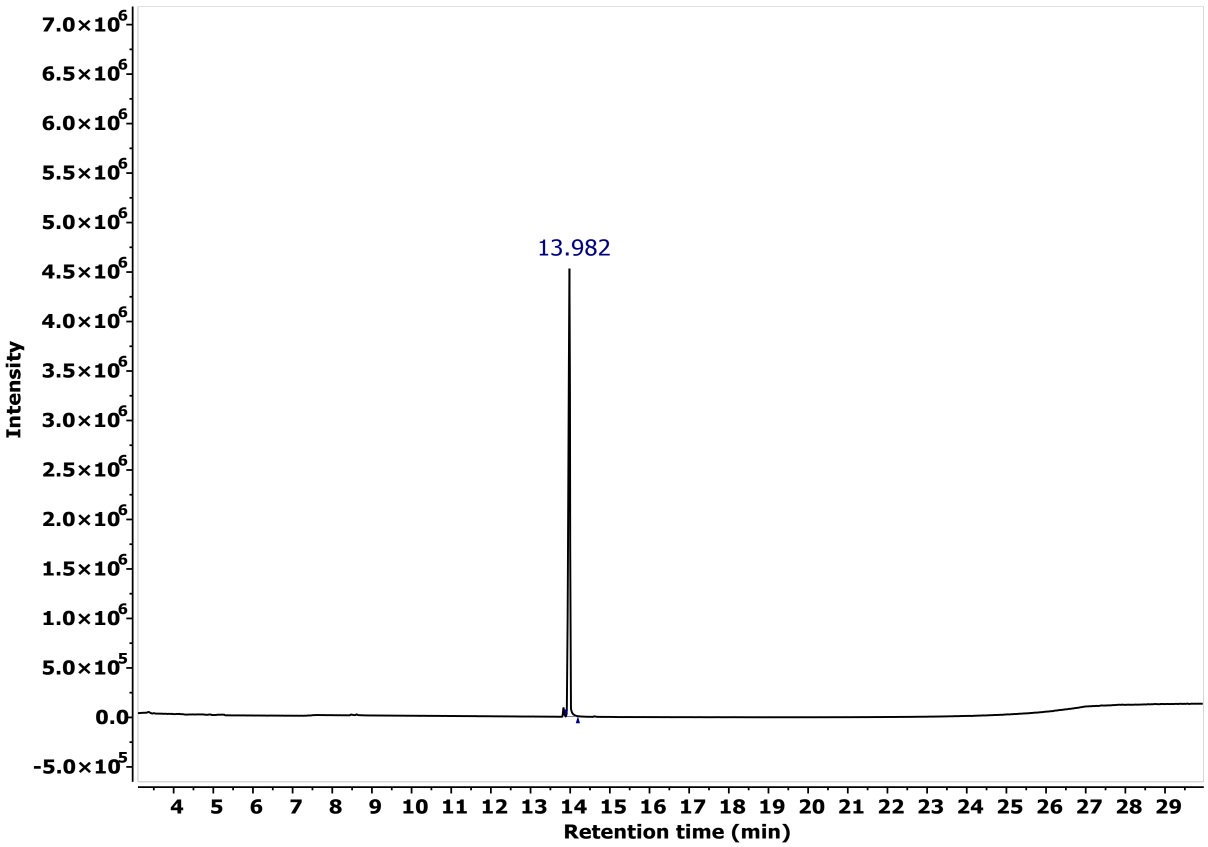

Figure S9. GC of 6d isolated from recycling of of PE-6d ($[Zn(Oct)_2]_0$:$[PE\text{-}6d]_0$ 1:100, 160 °C). Pure sample of 6d elutes at 13.98 minutes.

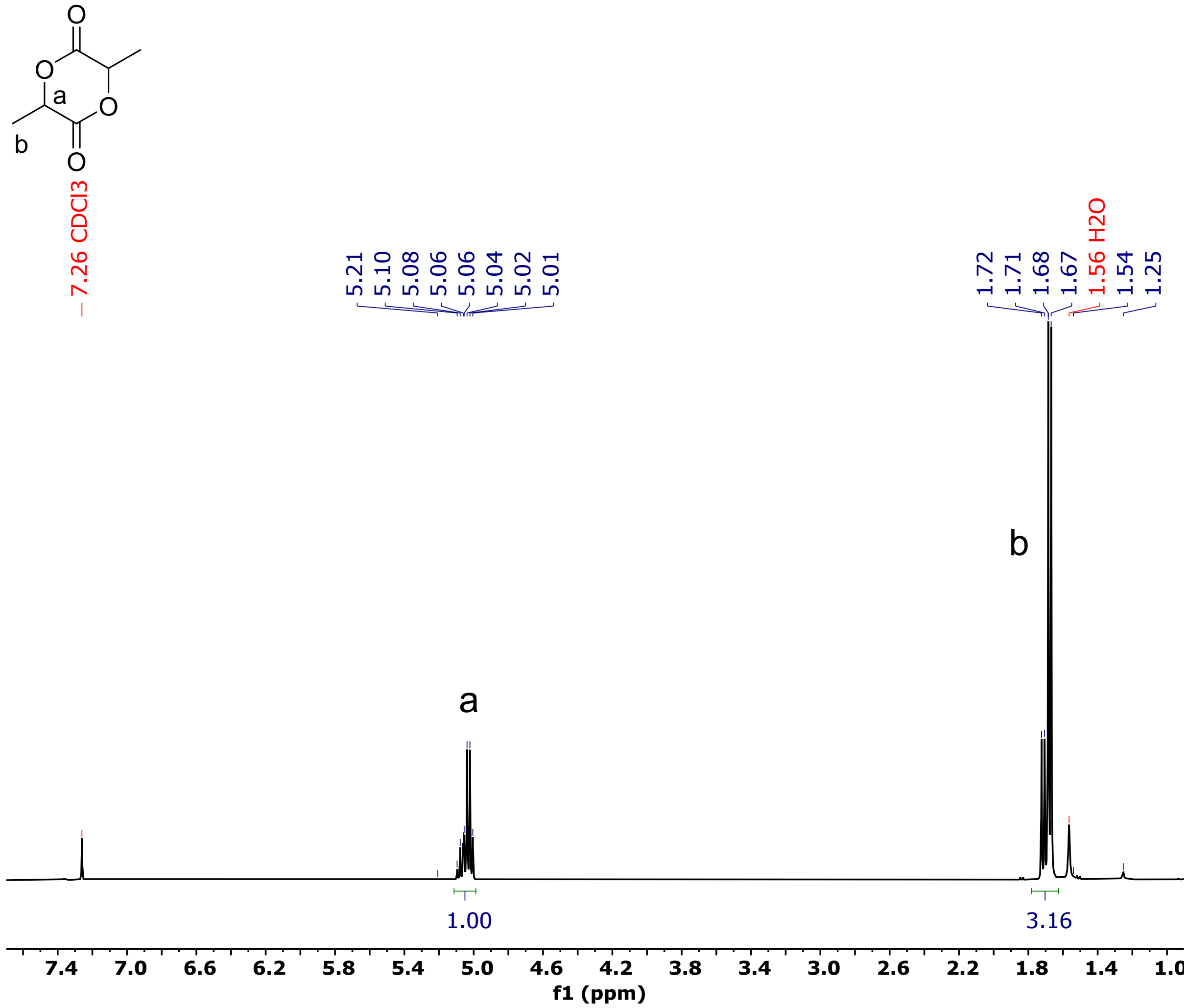

Figure S10. ^{1}**H NMR spectrum (400 MHz, $CDCl_3$, 298K) of rac-LA isolated from recycling of *rac*-PLA ($[Zn(Oct)_2]_0$:$[$*rac*-$PLA]_0$ 1:100, 160 °C) From integration of the methyl protons, *meso*-content estimated to be approximately 20%.**

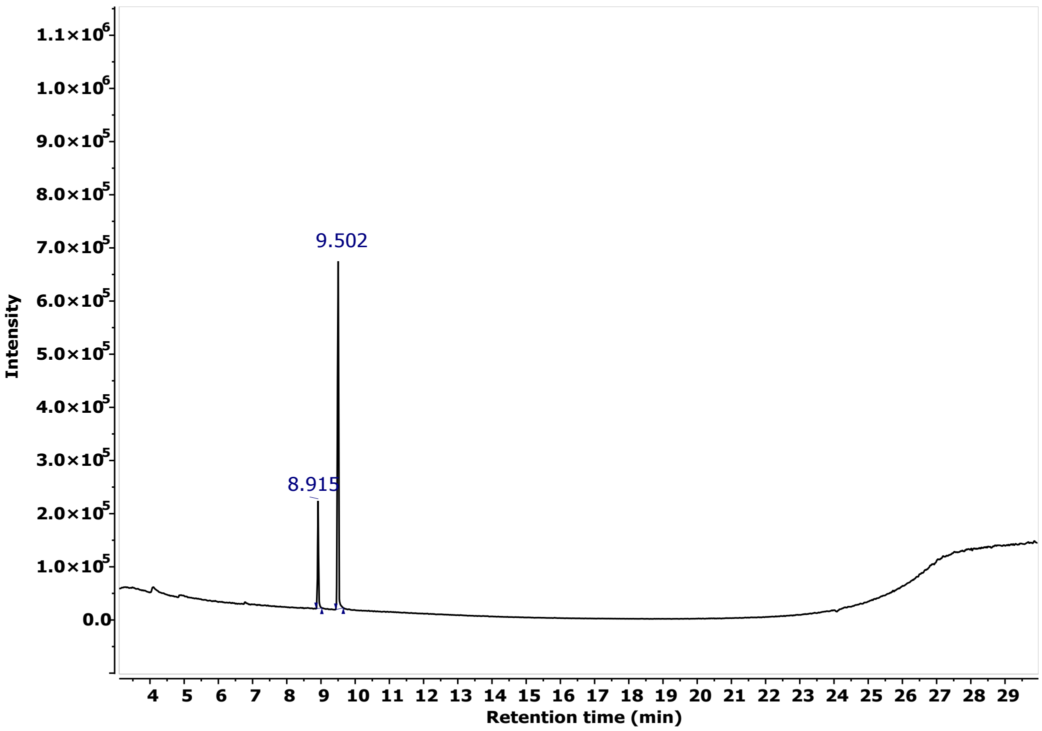

Figure S11. GC of rac-LA isolated from recycling of *rac*-PLA ($[Zn(Oct)_2]_0$:$[rac\text{-}PLA]_0$ 1:100, 160 °C) δ 5.05 (q, J = 6.7 Hz, 2H, L,L + D,L HCMe), 1.66 (d, J = 7.1 Hz, trace (6% meso), D,L HCMe)δ 1.60 (d, J = 6.7 Hz, 6H, L,L HCMe). Pure samples of *meso*-lactide elute at 8.92 minutes, pure samples of *L,L*-lactide elute at 9.50 minutes.

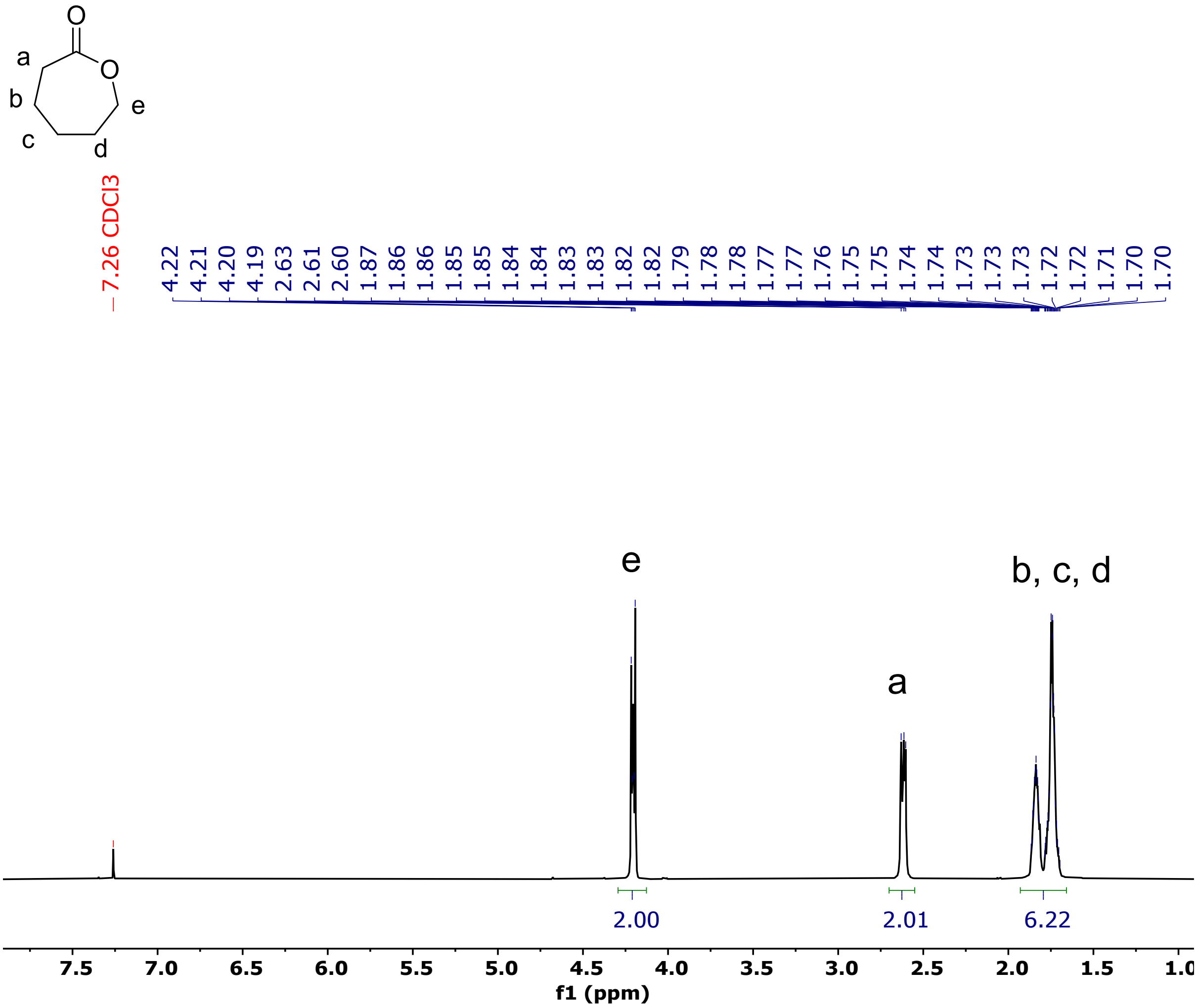

Figure S12. ^{1}H NMR spectrum (400 MHz, $CDCl_3$, 298K) of 7a isolated from recycling of PE-7a ($[Zn(Oct)_2]_0$:$[PE\text{-}7a]_0$ 1:100, 160 °C)

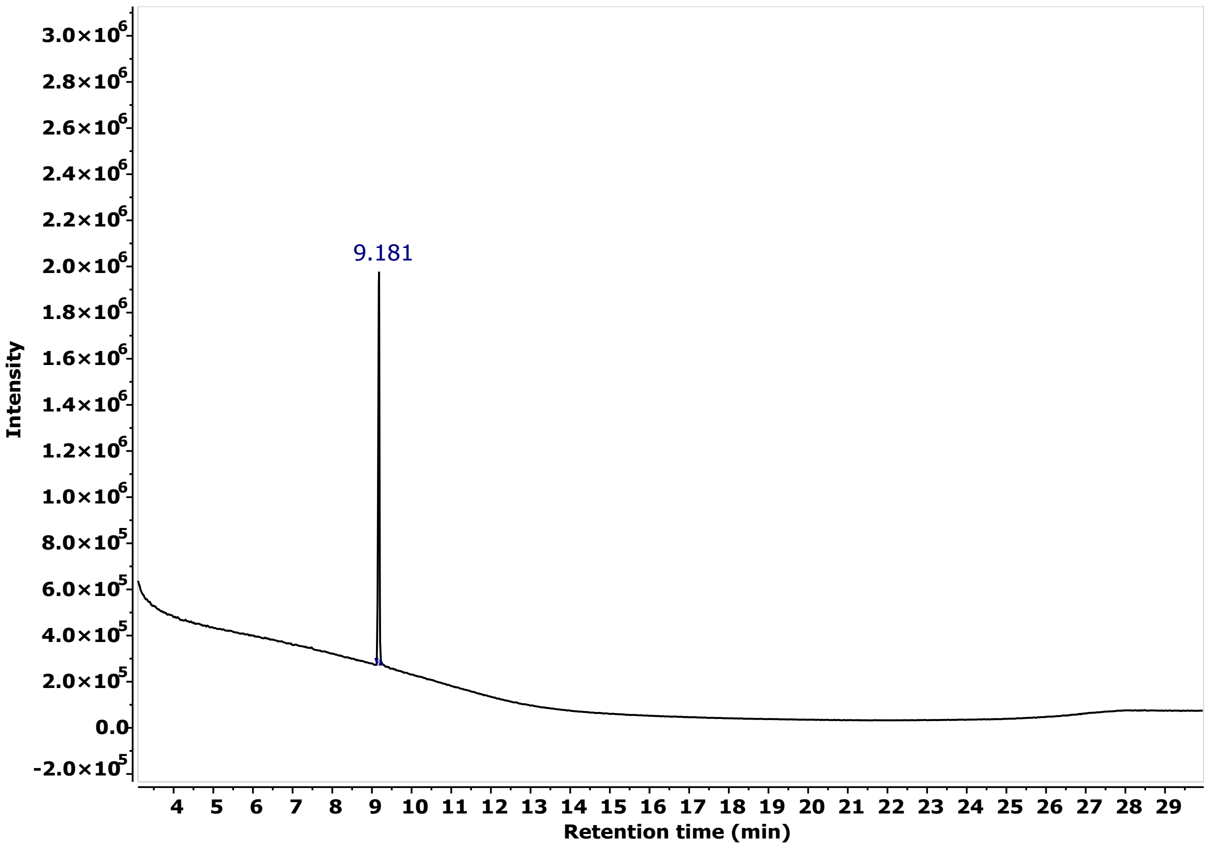

Figure S13. GC of 7a isolated from recycling of PE-7a ($[Zn(Oct)_2]_0$:$[PE\text{-}7a]_0$ 1:100, 160 °C)

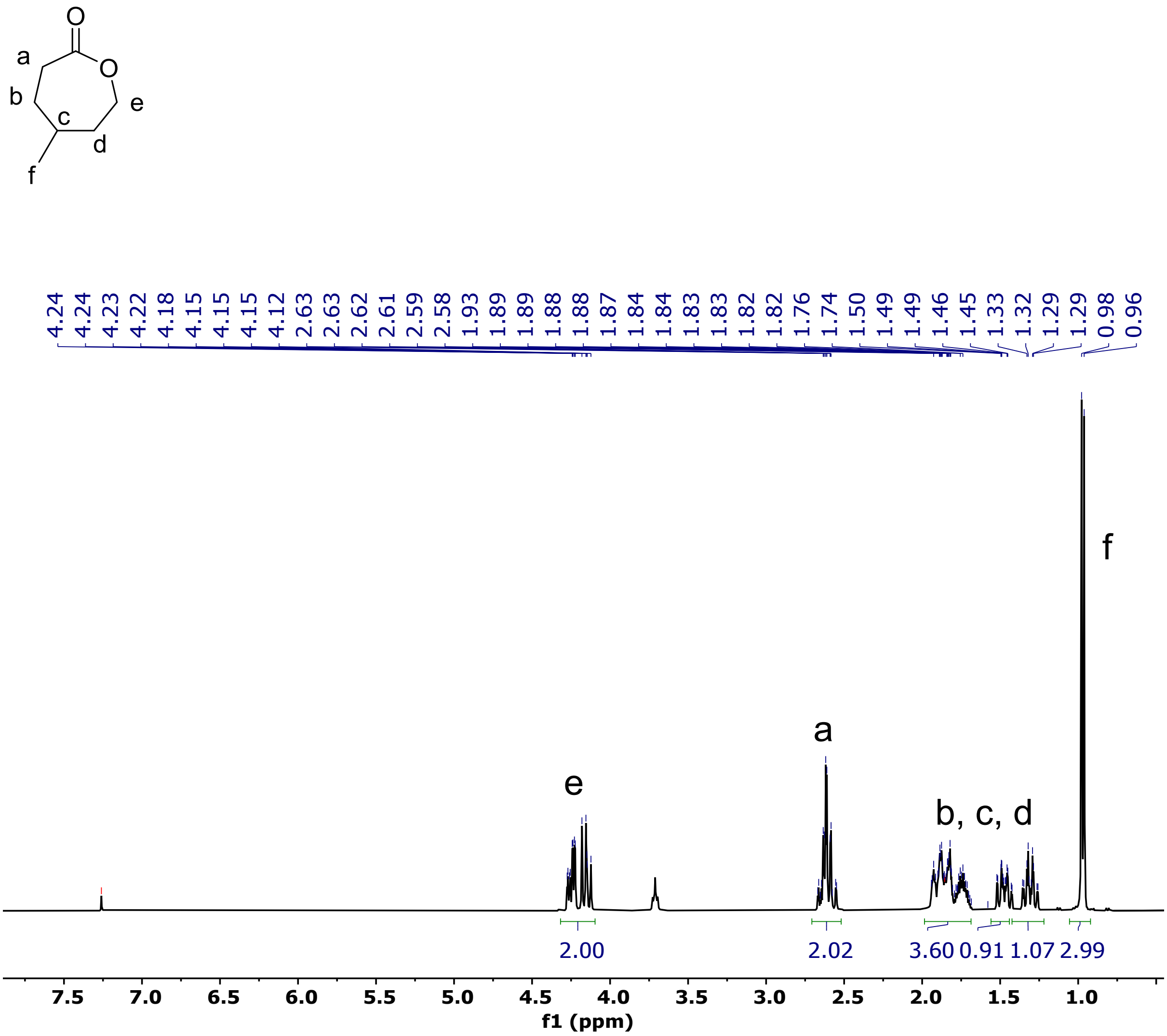

Figure S14. ^1H NMR spectrum (400 MHz, $CDCl_3$, 298K) of 7b isolated from recycling of PE-7b ($[Zn(Oct)_2]_0$:$[PE\text{-}7b]_0$ 1:100, 160 °C). Residual THF at δ = 3.75 ppm.

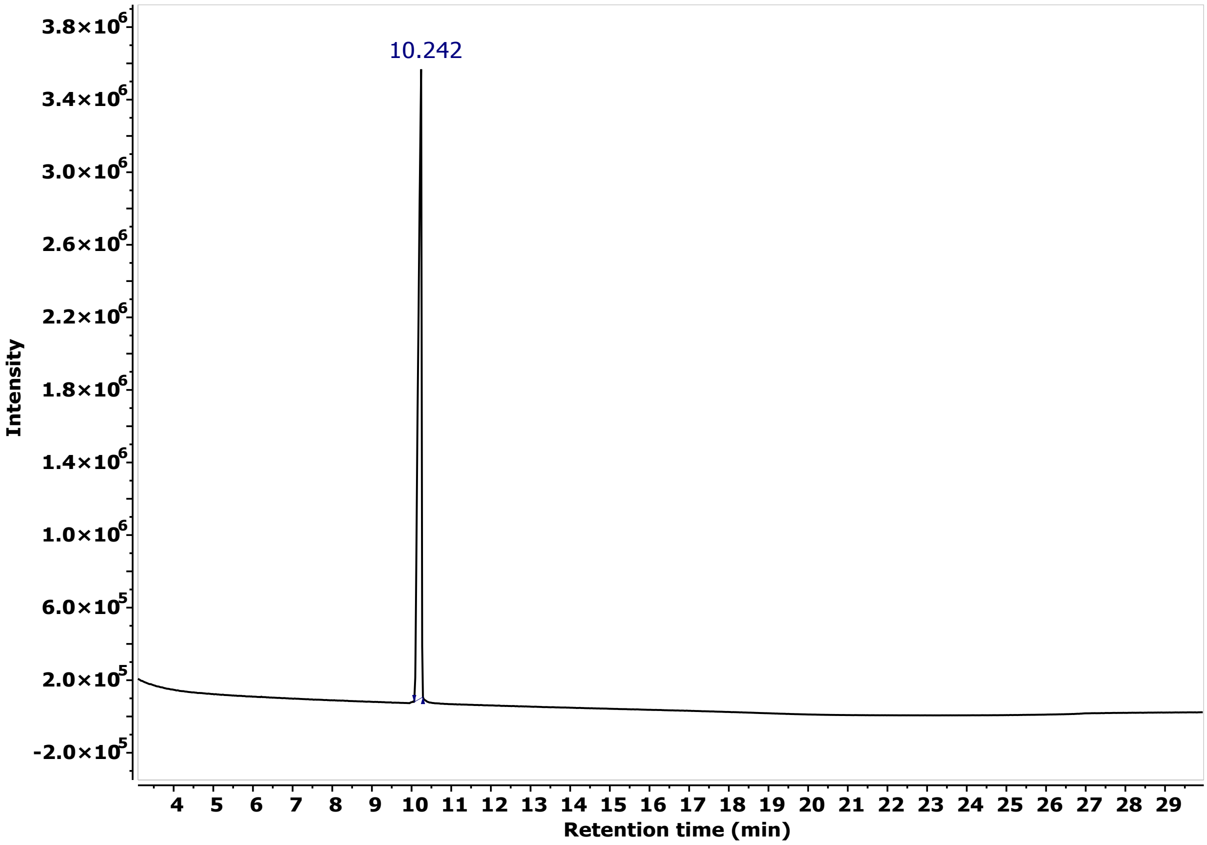

Figure S15. GC of 7b isolated from recycling of PE-7b ($[Zn(Oct)_2]_0$:$[PE\text{-}7b]_0$ 1:100, 160 °C)

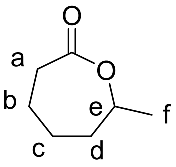

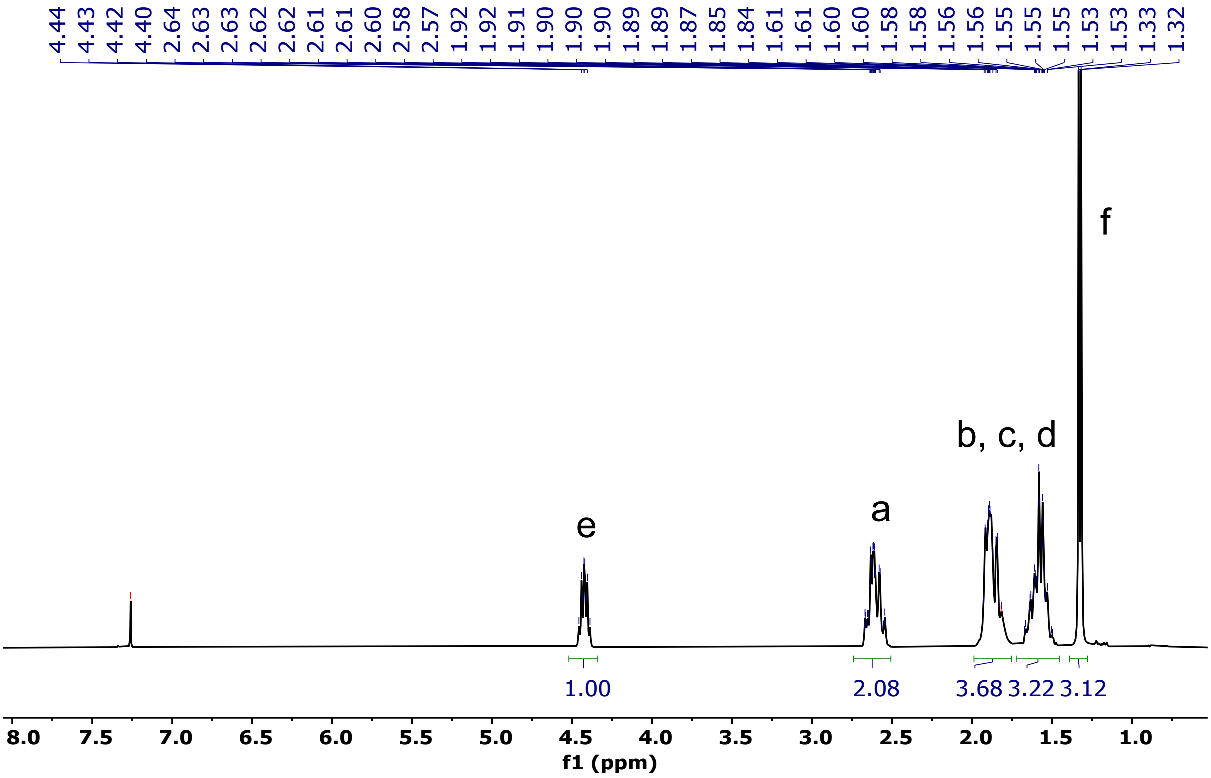

Figure S16. ^1H **NMR spectrum (400 MHz,** $CDCl_3$**, 298K) of 7a isolated from recycling of PE-7c (**$[Zn(Oct)_2]_0$**:**$[PE\text{-}7c]_0$ **1:100, 160 °C)**

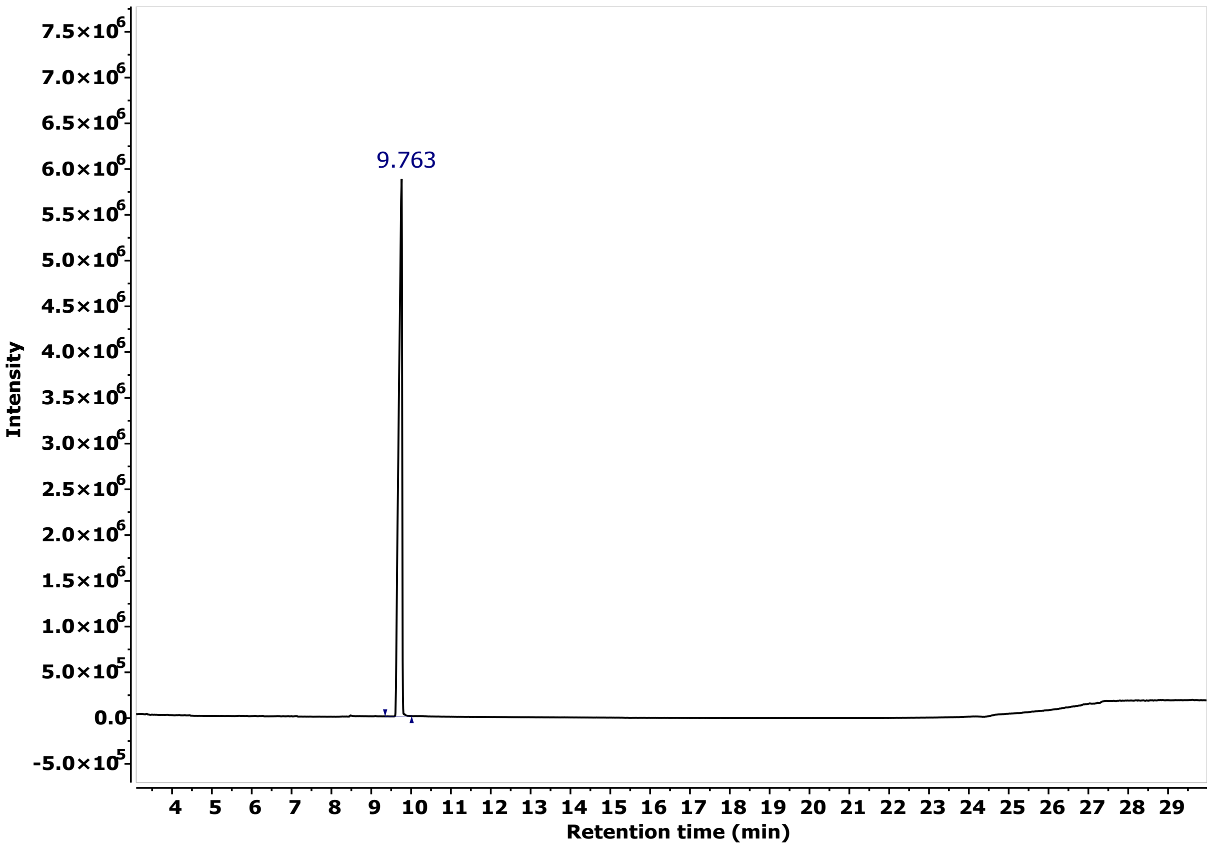

Figure S17. GC of 7c isolated from recycling of PE-7c ($[Zn(Oct)_2]_0$:$[PE\text{-}7c]_0$ 1:100, 160 °C)

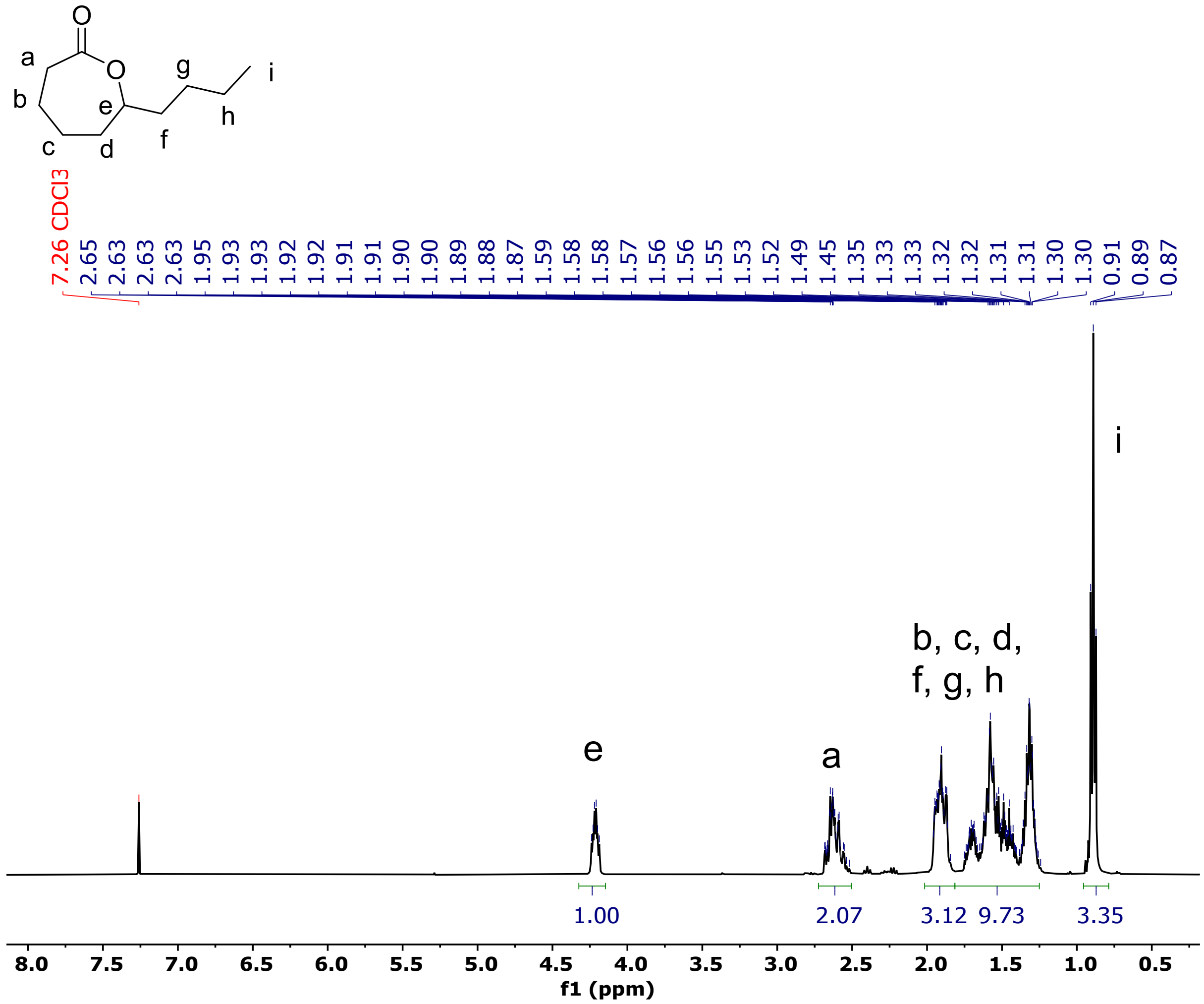

Figure S18. ^{1}H NMR spectrum (400 MHz, $CDCl_3$, 298K) of 7d isolated from recycling of PE-7d ($[Zn(Oct)_2]_0$:$[PE\text{-}7d]_0$ 1:100, 190 °C)

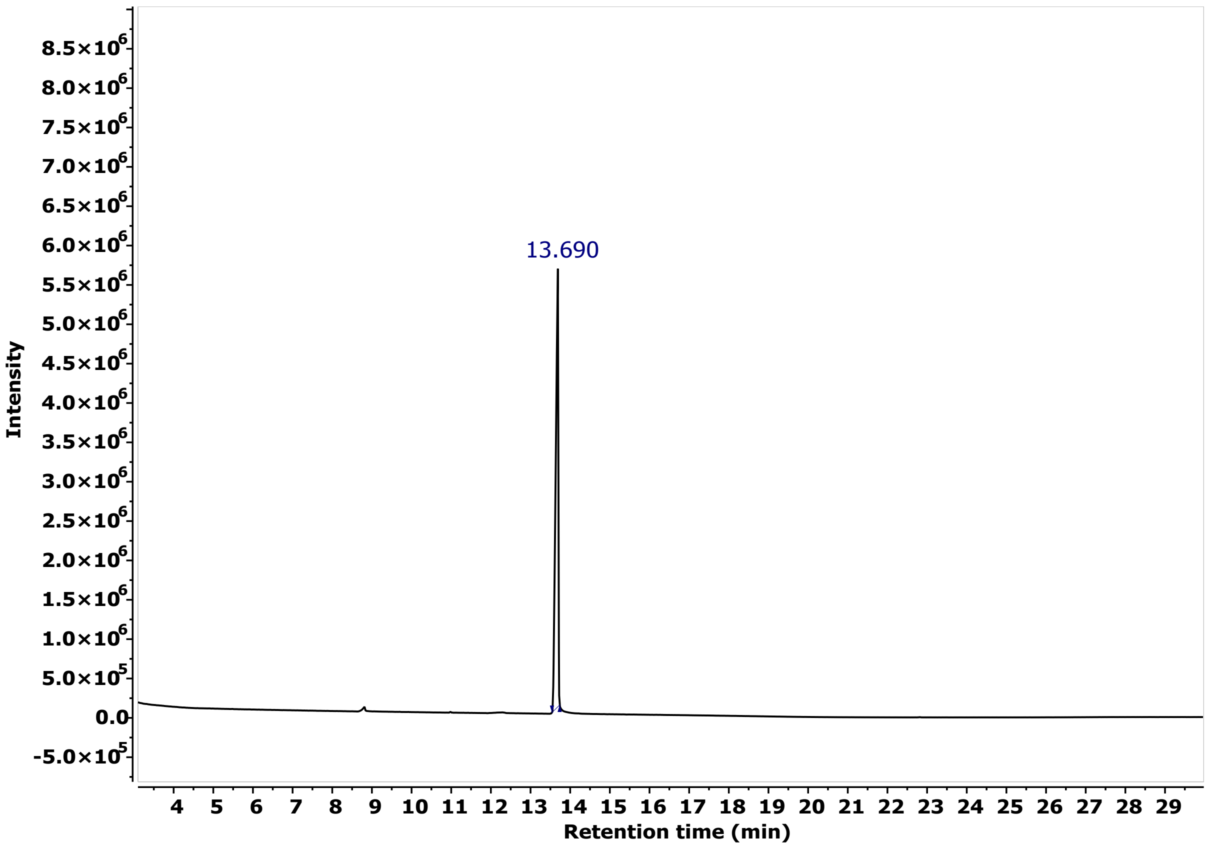

Figure S19. GC of PE-7d isolated from recycling of PE-7d ($[Zn(Oct)_2]_0$:$[PE\text{-}7d]_0$ 1:100, 190 °C)

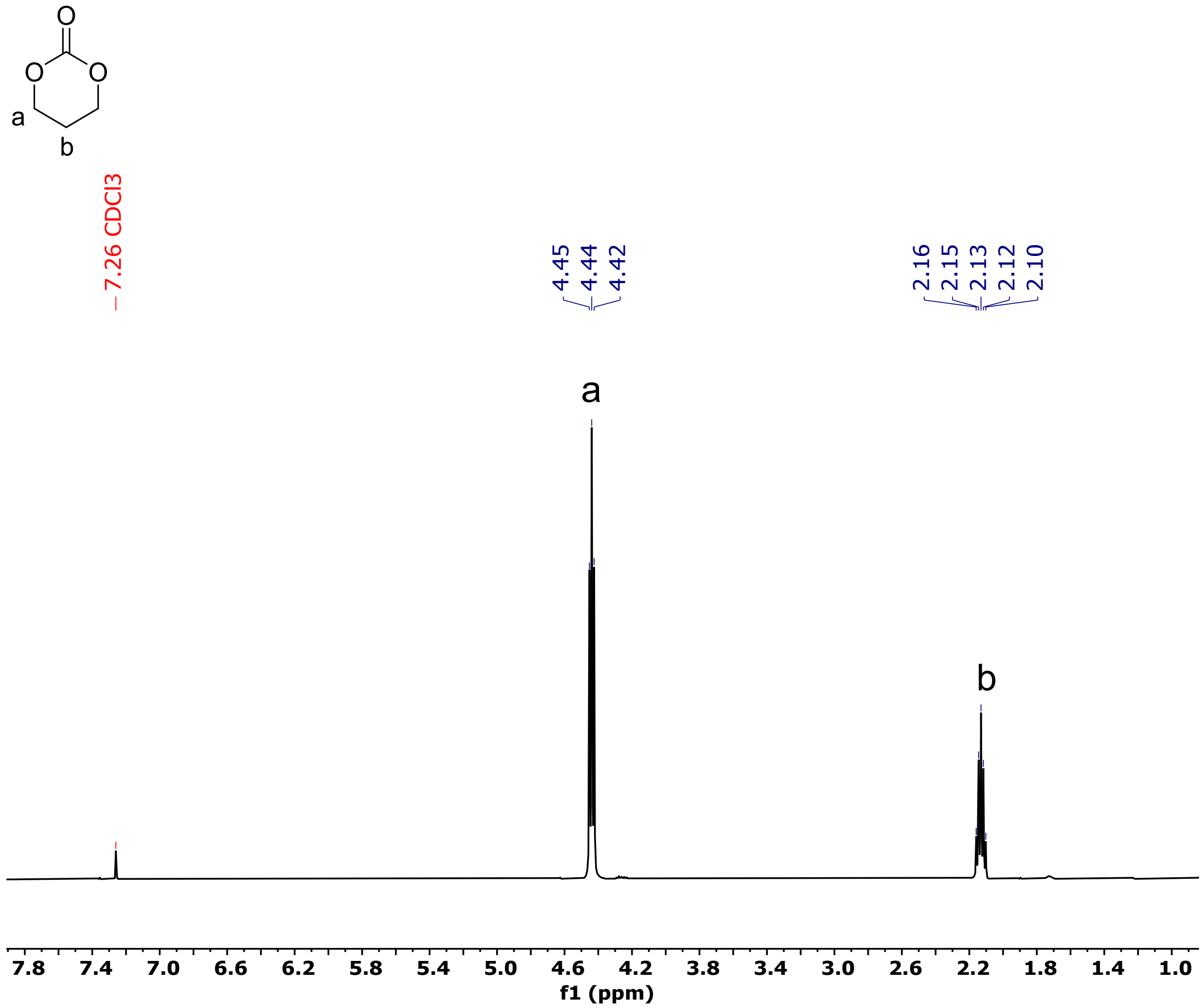

Figure S20. **^{1}H NMR spectrum (400 MHz, $CDCl_3$, 298K) of 6a' isolated from recycling of PC-6a ($[Zn(Oct)_2]_0$:$[PC\text{-}6a]_0$ 1:100, 160 °C)**

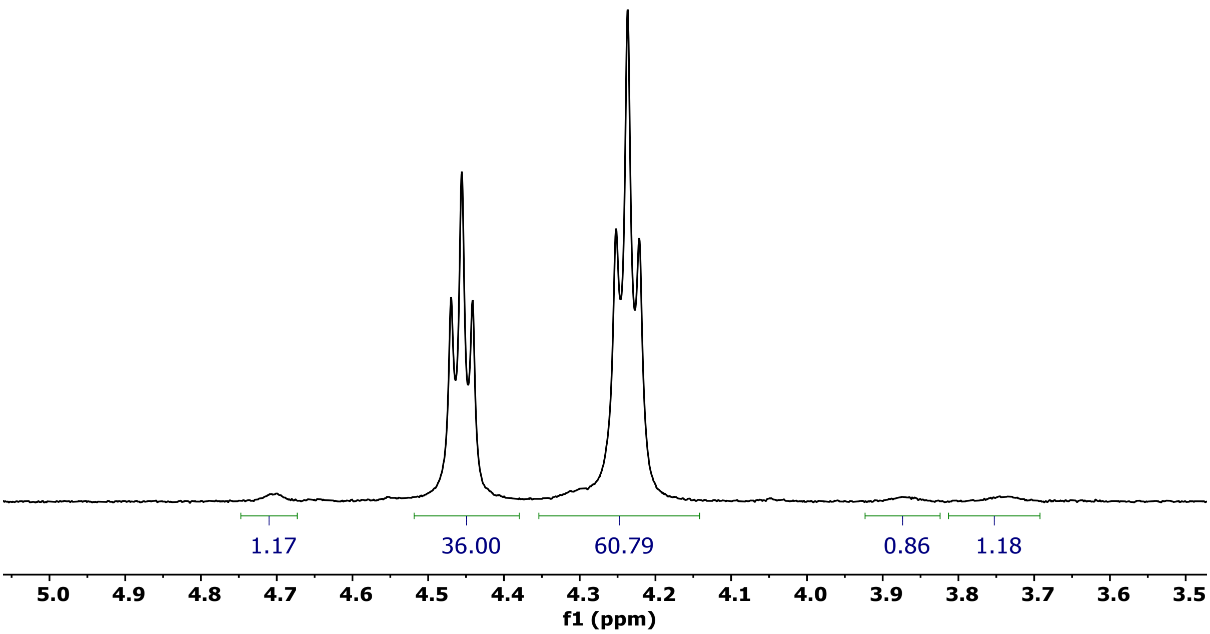

Figure S21. **^{1}H NMR spectrum (400 MHz, $CDCl_3$, 298K) of the polymer and catalyst mixture after depolymerization of PC-6a. ($[Zn(Oct)_2]_0$:$[PC\text{-}6a]_0$ 1:100, 160 °C). 96% of the mixture is confirmed as 6a' (δ = 4.45 ppm) and PC-6a (δ = 4.25 ppm) with resonances at δ = 4.7, 0.86 and 1.18 ppm attributed to trace amounts of oligomers. There is no evidence for significant formation of larger heterocycles.**

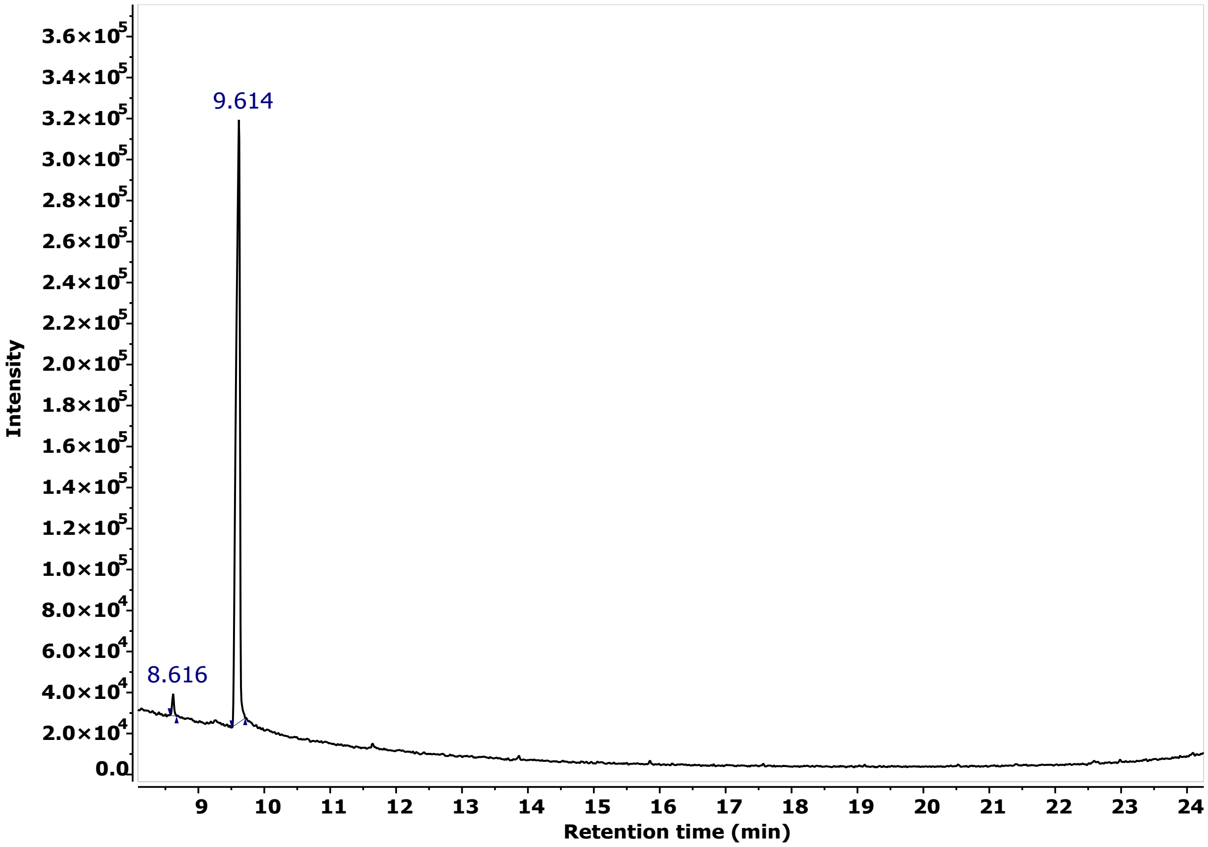

Figure S22. GC of 6a' isolated from recycling of PC-6a ($[Zn(Oct)_2]_0$:$[PC\text{-}6a]_0$ 1:100, 160 °C). Pure sample of 6a' elutes at 9.61 minutes. The additional peak at 8.61 minutes is attributed to residual $[Zn(2\text{-ethylhexanoate})]^+$ as inidicated by MS data which shows an *m/z*¸value *of* 207.04 (m/z_{theo} *of* $[Zn(2\text{-ethylhexanoate})]^+$ = 207.04).

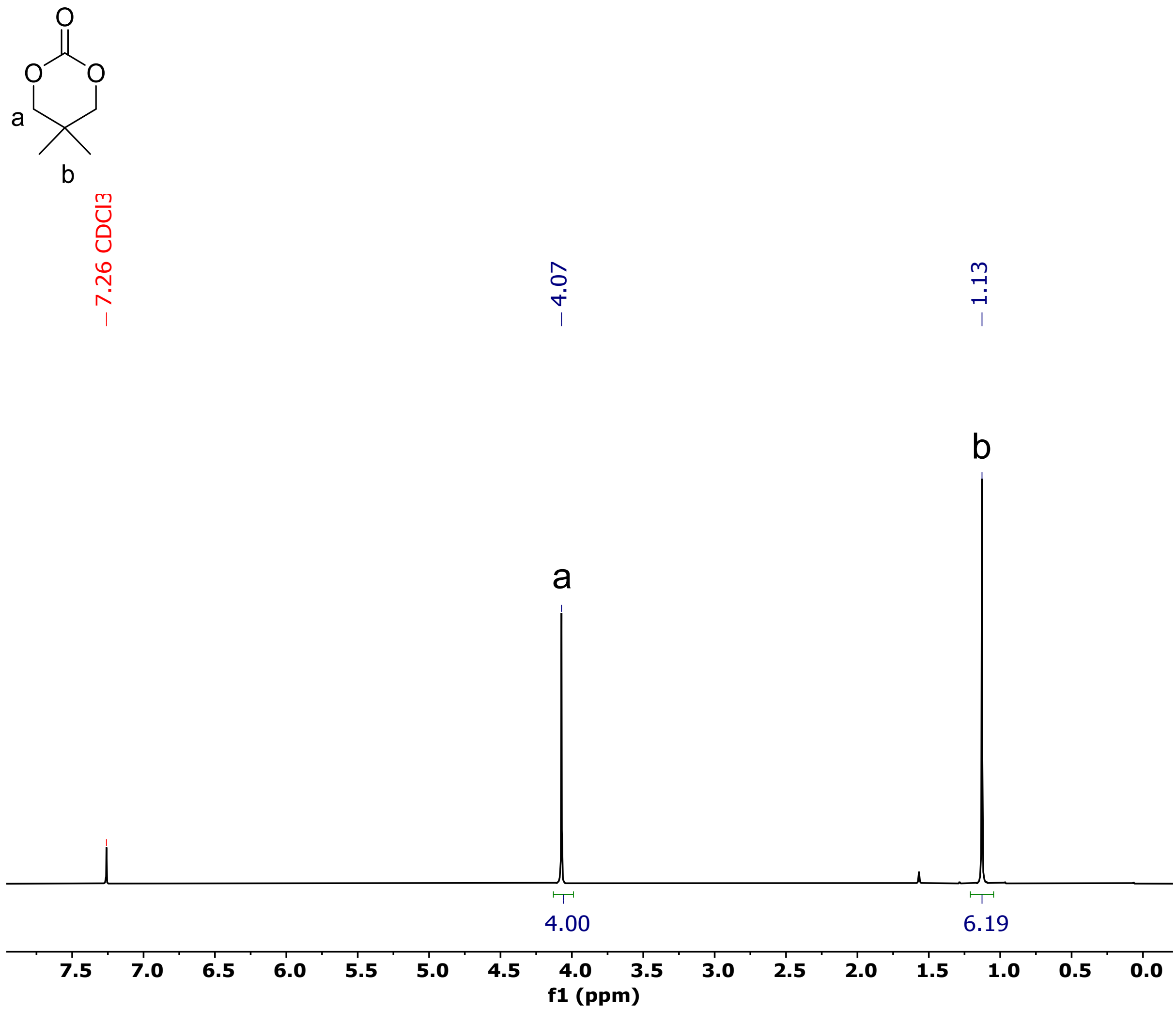

Figure S23. **^{1}H NMR spectrum (400 MHz, $CDCl_3$, 298K) of 6b isolated from recycling of PC-6b ($[Zn(Oct)_2]_0$:$[PC\text{-}6b]_0$ 1:100, 160 °C)**

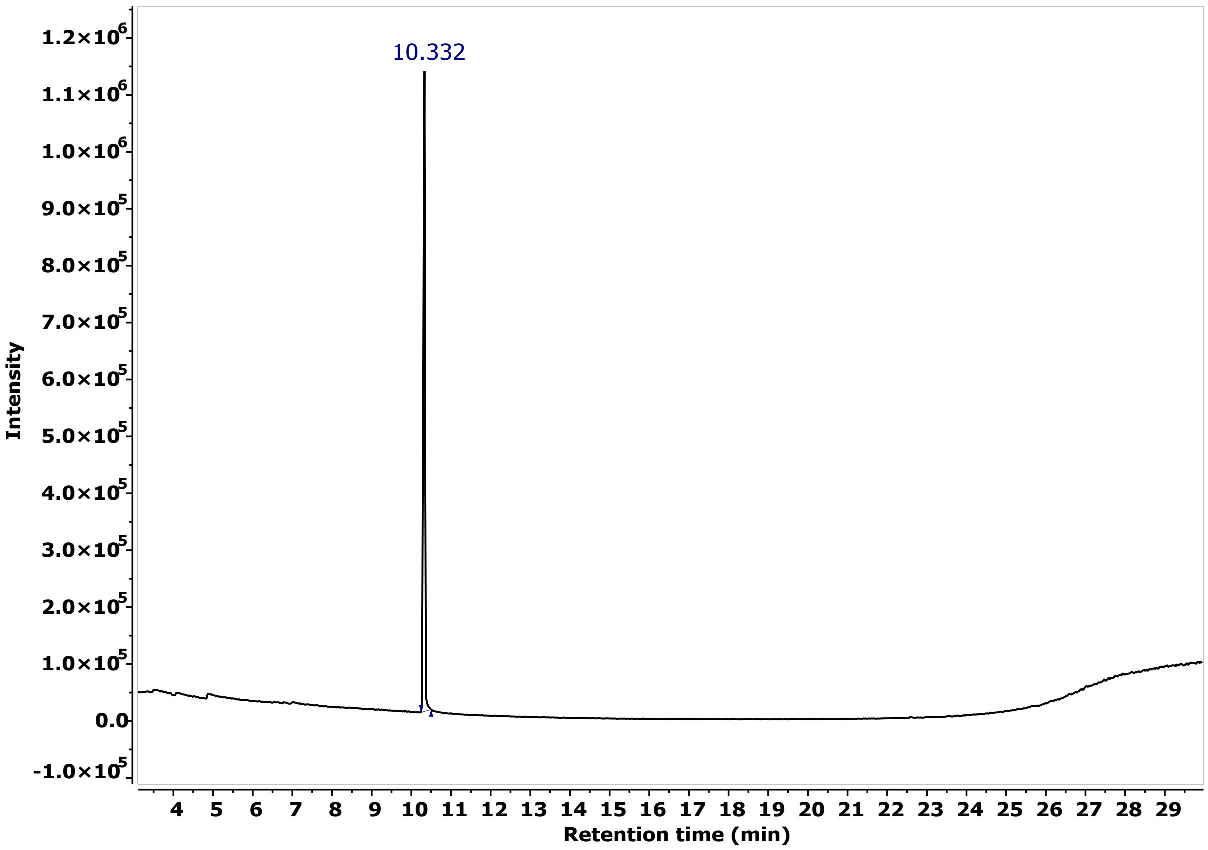

Figure S24. GC of 6b isolated from recycling of PC-6b ($[Zn(Oct)_2]_0$:$[PC\text{-}6b]_0$ 1:100, 160 °C)

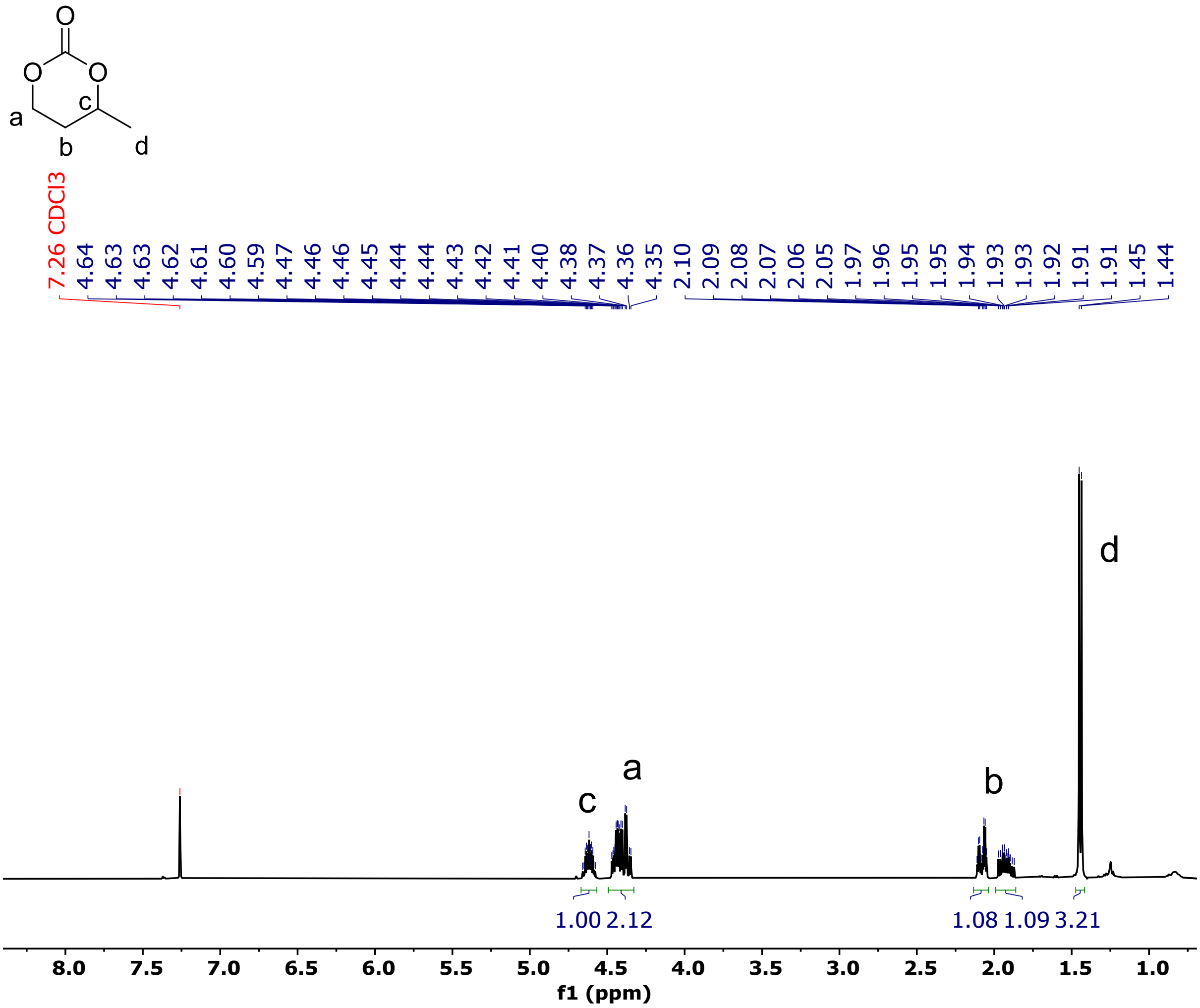

Figure S25. ^{1}H NMR spectrum (400 MHz, $CDCl_3$, 298K) of 6c isolated from recycling of PC-6c ($[Zn(Oct)_2]_0$:$[PC\text{-}6c]_0$ 1:100, 160 °C)

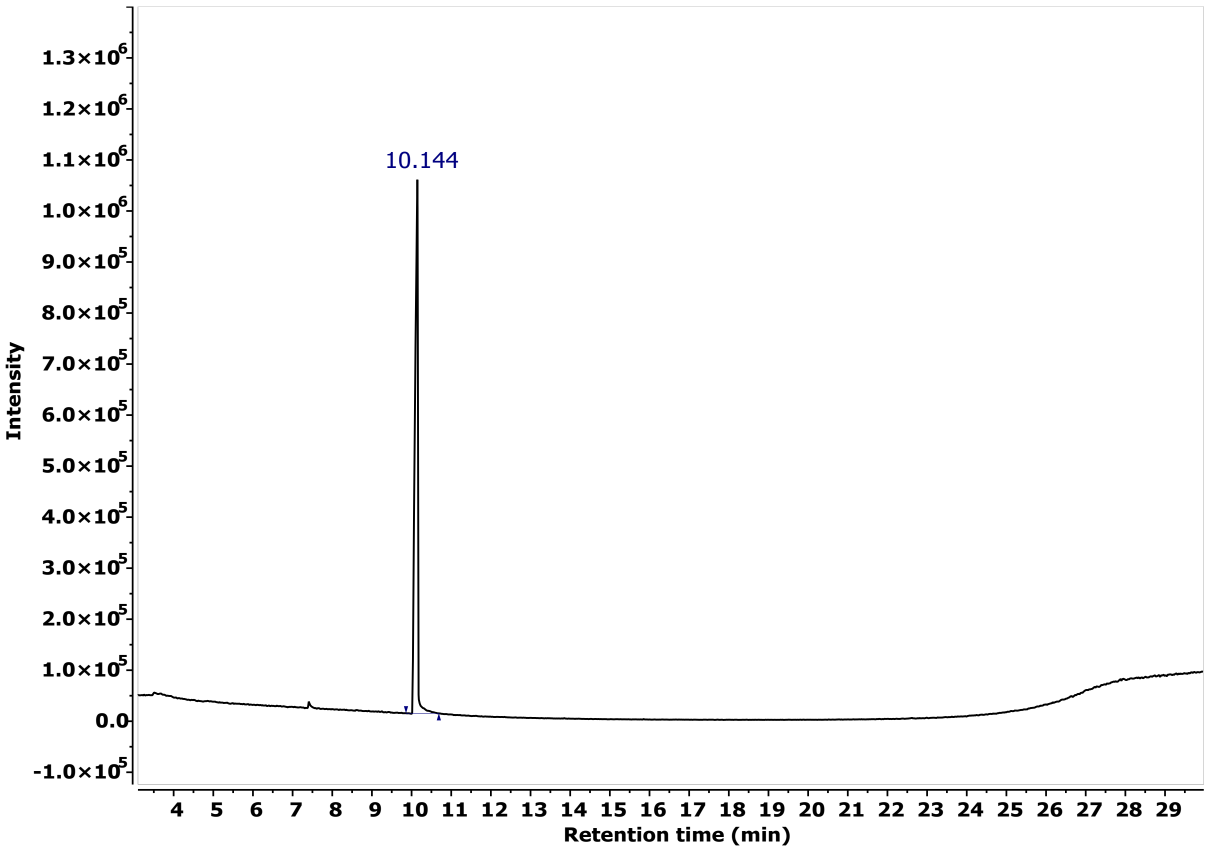

Figure S26. GC of 6c isolated from recycling of PC-6c ($[Zn(Oct)_2]_0$:$[PC\text{-}6c]_0$ 1:100, 160 °C

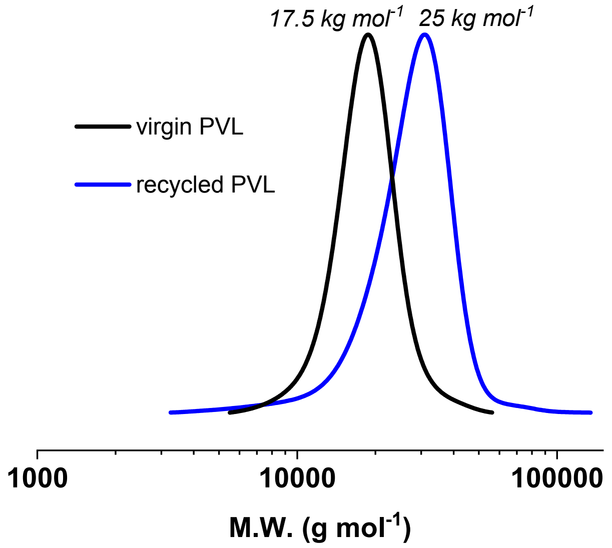

Figure S27. SEC traces for virgin (black) and recycled PE-6a (black). Recycled PE-6a $M_{n,SEC}$ = 25,000 g mol^{-1}, Đ$_M$ = 1.18; Virgin PE-6a $M_{n,SEC}$ = 17,500 g mol^{-1}, Đ$_M$ = 1.18.

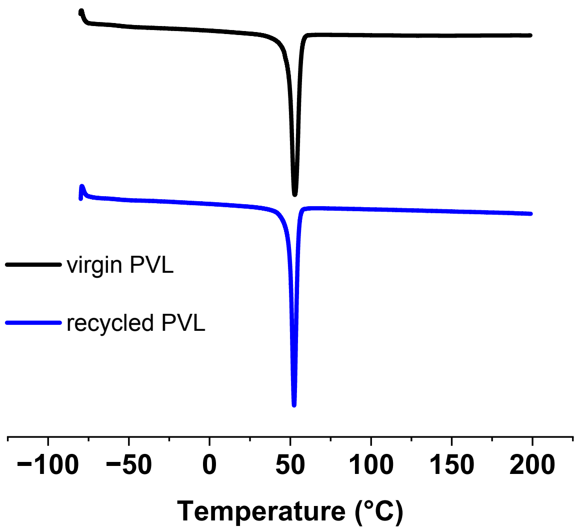

Figure S28. DSC thermogram showing the second heating cycle of the virgin (black) and recycled PE-6a (blue). Recycled PE-6a: T_m = 52 °C, T_g = - 55 °C, T_{d5} = 257 °C; virgin PE-6a T_m = 53 °C, T_g = - 55 °C, T_{d5} = 276 °C).

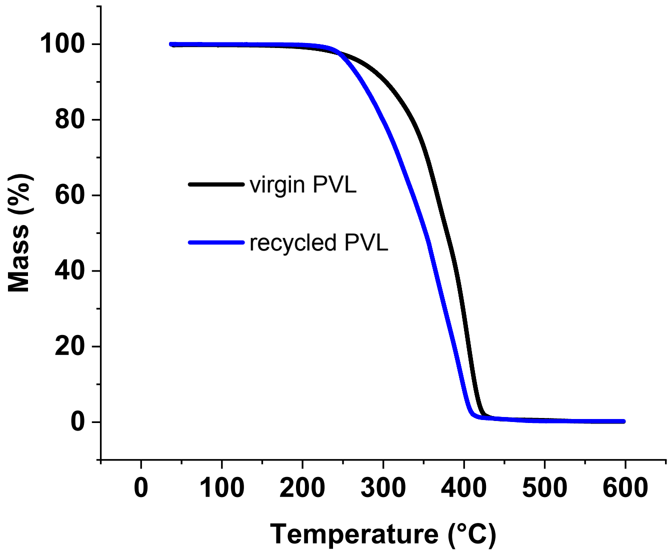

Figure S29. TGA thermogram virgin (black) and recycled PE-6a (blue). Recycled PE-6a: T_{d5} = 257 °C; virgin PE-6a T_m = 53 °C, T_g = - 55 °C, T_{d5} = 276 °C .

Depolymerization data

Recycling of PE-6a

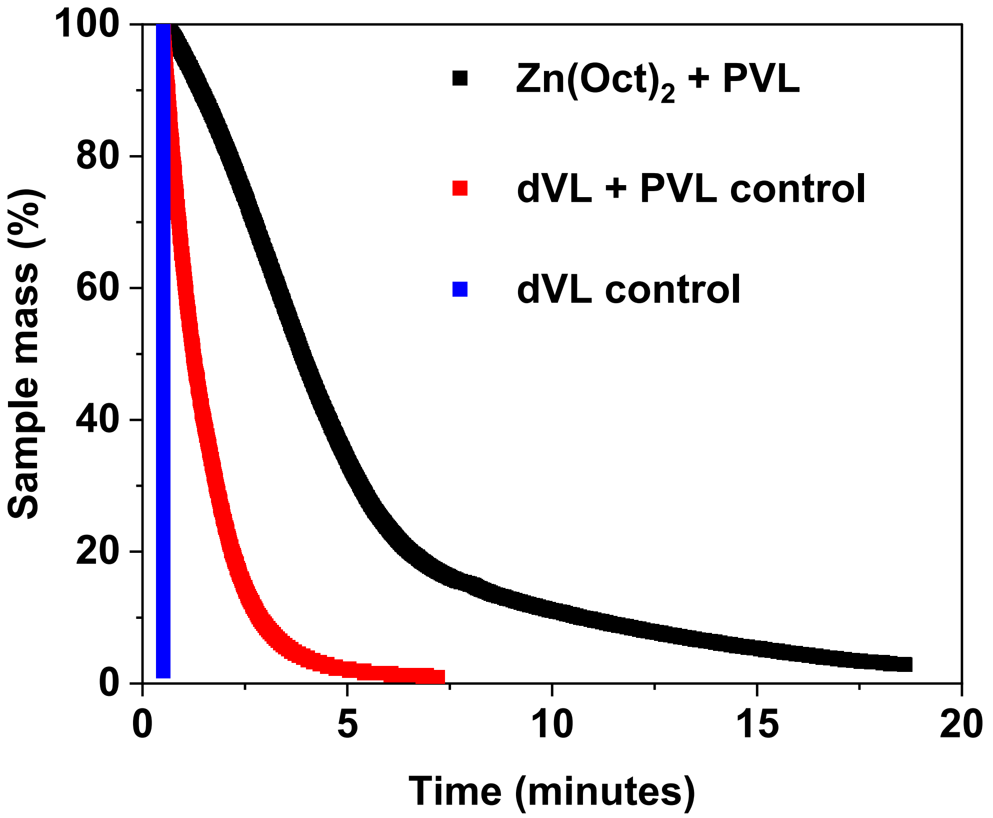

Figure S30. Mass loss vs time for $Zn(Oct)_2$ catalysed depolymerization of PE-6a ($[Zn(Oct)_2]_0$: $[PE\text{-}6a]_0$, 1: 1000, black) , 6a/PE-6a blend (red) and 6a (blue). Reactions and controls were performed at 130 °C.

Table S4. Mass loss data from catalysed depolymerization of PE-6a and control reactions

Entry	Reaction	k[a] (min^{-1})	a[a]	xc[a] (min)	*ka/4* (*secs*)	k_{obs} (s^{-1})[b]
1[c]	$Zn(Oct)_2$ + PE-6a	0.507	0.929	5.848	0.00281	0.0027 ±0.0003
		0.423	0.914	6.661	0.00292	
		0.305	0.913	7.260	0.00226	
2[d]	dVL + PE-6a	2.014	0.922	1.298	0.00774	0.0082± 0.0004
		2.164	0.921	1.217	0.00831	
		2.262	0.923	1.255	0.00870	
3[e]	dVL	11.858	1.07	0.700	0.0531	0.052 ± 0.001
		11.355	1.07	0.710	0.0507	
		11.350	1.07	0.710	0.0507	
4	$Co(Oct)_2$ + PE-6a	0.217	0.867	10.861	0.000785873	0.00077 ±0.0001
		0.200	0.876	11.835	0.000729926	
		0.220	0.881	10.609	0.000806207	

N_2 flow = 25 mL min^{-1}.[a] Determined from logistic fitting of conversion *vs* time profiles from 0 – 90% mass loss.[b] k_{obs} = average of 3 repeats, error = standard deviation from 3 repeats.[c] Reaction conducted by solvent casting $Zn(Oct)_2$:PE-6a solutions ($[Zn(Oct)_2]_0$: $[PE\text{-}6a]_0$ = 1: 1000, $[Zn(Oct)_2]_0$ = 9.98 x 10^{-3} M in bulk polymer, $[PE\text{-}6a\ solution]_0$ = 0.5 M) in TGA crucibles.[d] Reaction conducted by solvent casting dVL: PE-6a solutions ($[\delta Vl]_0$: $[PVL]_0$ = 1: 1, $[PVL + dVL\ solution]_0$ = 0.5 M) in TGA crucibles.[e] Reaction conducted by dropcasting *ca* 10 mg of dVL into crucible. Mass loss calculated for % mass loss of 1 mg.

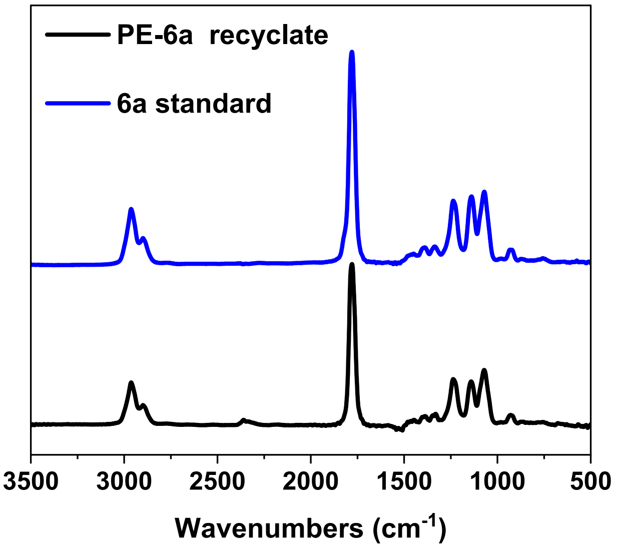

Figure S31. TGA-FTIR gas-phase analysis of PE-6a depolymerization mixture catalysed by $Zn(Oct)_2$ ($[Zn(Oct)_2]_0$:$[PE\text{-}6a]_0$ = 1:1000, 130 °C, black) and an 6a standard (blue).

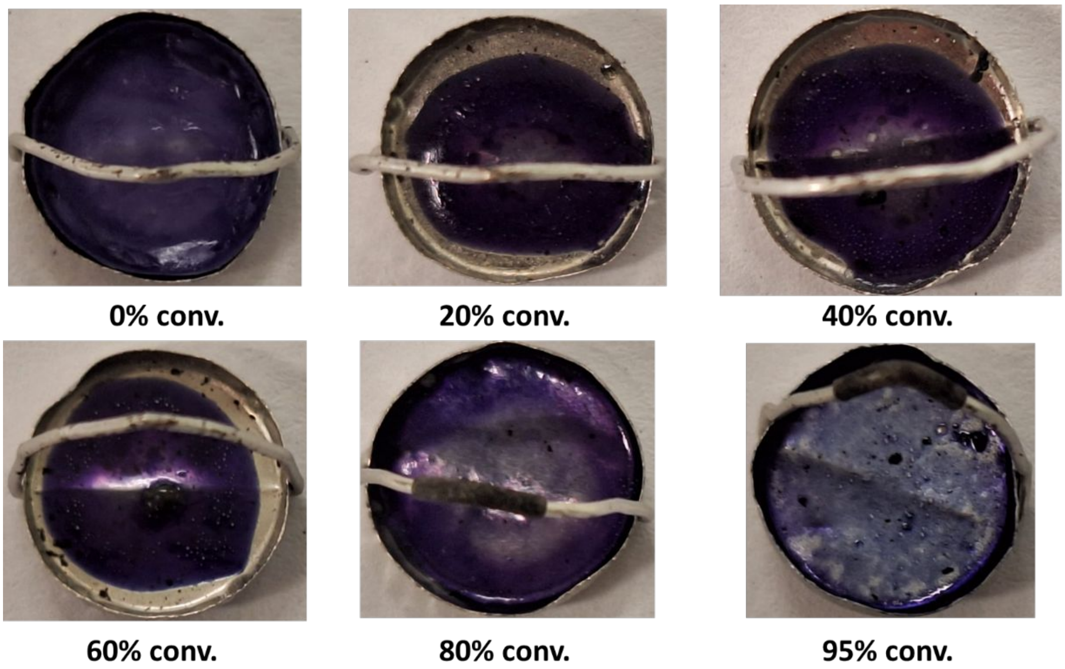

Figure S32. Photographs of the polymer film (1:100, $Co(Oct)_2$:PE-6a) taken throughout the course of the depolymeirzation. Conversion was determined by mass loss in the mass loss *vs* time TGA profile.

Fig. S32 shows that the polymer film remains intact throughout the reaction, resulting in a progressive increase in catalyst concentration. To account for this, a sigmoidal function (named 'slogistic1' in Origin 2023, see section 2.4 for details) widely used to model processes where the catalyst concentration is not constant (e.g. autocatalytic reactions), was used to fit the data.

It is noted that the E_a as determined by Friedman's isonversional analysis is within ±10% across the duration of the reaction (10-80% conversion, Table S5 and Fig. S34). This indicates that kinetics of the depolymerization process can be modelled as a single step, meaning physical effects of changing sample viscosity, molar mass and char formation have limited impact on the depolymerization kinetics. The data also implies that initiation is likely faster than depolymerization; these two steps are chemically distinct and are expected to show different activation energies. Initiation is known to occur rapidly in dilute solutions at temperatures of 80 °C while the depolymerization is conducted at temperatures of 130 °C. The measured E_a is thus proposed to correspond to the depolymerization step.

The sigmoidal function shows excellent fit to the conversion *vs* time data for PE-6a depolymerization across the range temperatures ranges measured (Table S6 and Fig. S33). Determination of activation energy to PE-6a depolymerization through plots of ln k_{obs} vs reciprocal temperature showed values within error to those determined using Friedman isoconversional analysis. E_a's are also within error to closely related systems (Table S7). This data suggests that the logistic gives a reasonable estimation of kinetic parameters.

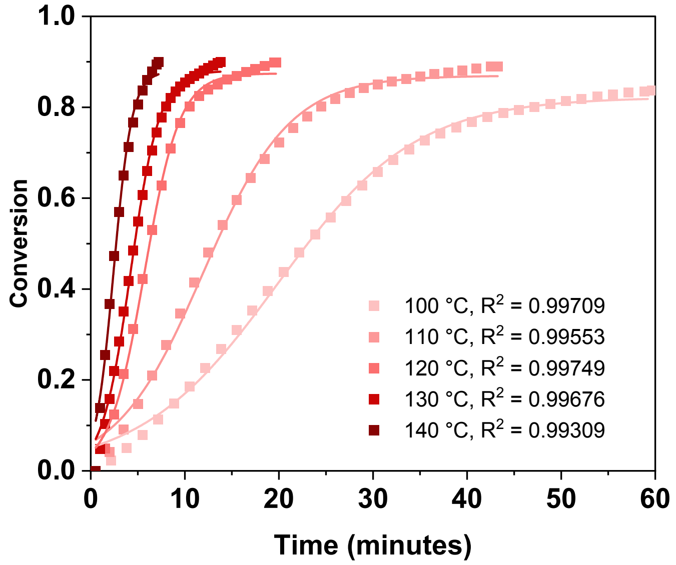

Figure S33. TGA thermograms showing experiments monitoring PE-6a conversion in $Zn(Oct)_2$:PE-6a mixtures (1:1000). The TGAs were run at 100, 110, 120, 130 and 140 °C for 1 h or until >95% conversion

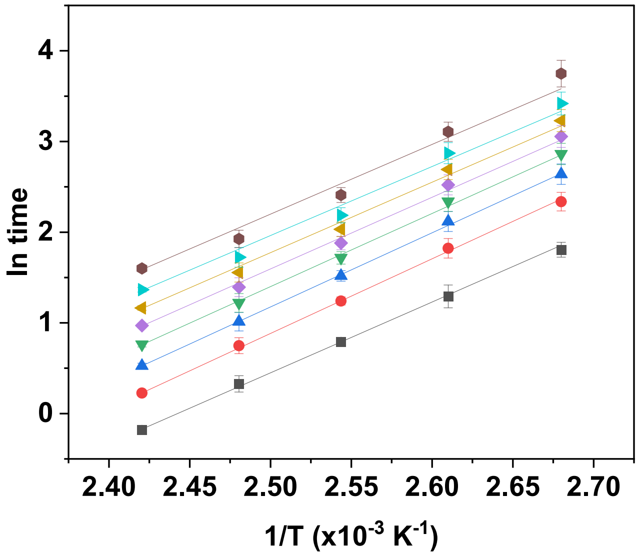

Figure S34. Plot of ln *time* vs 1/T for the recycling of PE-6a. The time was taken at 10, 20, 30, 40, 50, 60, 70, 80% conversion at 100, 110, 120, 130 and 140 °C, and the gradient = E_a/R, where R is the gas constant. The reactions were performed with $[Zn(Oct)_2]_0$: $[PE\text{-}6a]_0$ of 1:1000. $[Zn(Oct)_2]_0$ = 9.99 x10^{-3} M

Table S5. Friedman isoconversional analysis for the depolymerization of PE-6a conducted at temperatures 100-140 °C with 1:1000 $[Zn(Oct)_2]_0$:$[PE\text{-}6a]_0$ loading

Conversion	E_a (kJ mol^{-1})
10	64.9
20	68.4
30	68.0
40	67.0
50	65.7
60	64.5
70	63.3
80	63.8
Average	65.7±1.7

Table S6. Depolymerization of PE-6a at 100 – 140 °C with 1:1000 $[Zn(Oct)_2]_0$:$[PE\text{-}6a]_0$ loading

Entry	Temp. (°C)	*k*[a] (min^{-1})	*a*[a]	*xc*[a] (min)	*ka/4* (*secs*)	k_{obs} [b] (s^{-1})	k_d [c] (mol^{-1} dm^3 s^{-1})	TOF[d] (h^{-1})
1	100	0.170	0.857	15.897	0.000607	0.00047 ±0.00011	0.024 ±0.005	1200 ±200
		0.136	0.823	19.860	0.000465			
		0.146	0.781	21.600	0.000340			
2	110	0.289	0.867	9.625	0.00104	0.00089 ±0.00011	0.045 ±0.006	2200 ±250
		0.224	0.856	11.713	0.000800			
		0.233	0.850	12.288	0.000826			
3	120	0.507	0.893	5.848	0.00189	0.00152 ±0.00031	0.076 ±0.016	3880 ±190
		0.423	0.878	6.661	0.00155			
		0.305	0.793	7.260	0.00112			
4	130	0.727	0.929	3.830	0.00281	0.00267 ±0.00029	0.13 ±0.01	7000 ±400
		0.767	0.914	3.469	0.00292			
		0.595	0.913	4.436	0.00226			
5	140	0.908	0.921	2.570	0.00349	0.00342 ±0.00052	0.17 ±0.03	10470 ±180
		0.866	0.919	2.670	0.00331			
		0.904	0.923	2.518	0.00347			

Reaction conducted by solvent casting $Zn(Oct)_2$:PE-6a solutions ($[Zn(Oct)_2]_0$: $[PE\text{-}6a]_0$ = 1: 1000, $[Zn(Oct)_2]_0$ = 9.98 x 10^{-3} M in bulk polymer) in TGA crucibles. N_2 flow = 25 mL min^{-1}.[a] Determined from logistic fitting of conversion *vs* time profiles from 0 – 90% mass loss.[b] k_{obs} = average of 3 repeats, error = standard deviation from 3 repeats.[c] k_d = k_{obs}/(2 x $[cat]_0$), assuming 2 active chains per metal centre. [d] TOF = moles of PE-6a consumed/time * moles of catalyst. Determined from 0 – 30% conversion.

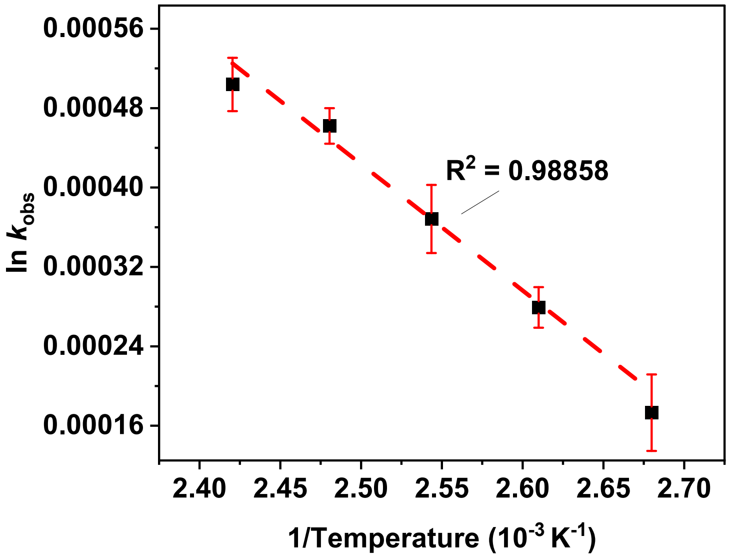

Figure S35. Plot of ln k_{obs} vs 1/T for the recycling of PE-6a. Reactions were performed at 100, 110, 120, 130 and 140 °C with loadings of $[Zn(Oct)_2]_0$: $[PE\text{-}6a]_0$ of 1:1000. $[Zn(Oct)_2]_0$ = 9.99 x10^{-3} M

Table S7. Comparison of activation parameters determined via different methods

Recycling conditions	Method	E_a (kJ mol^{-1})
1:1000, $Zn(Oct)_2$: PE-6a	Logistic fit, arrenhius analysis	63.5 ± 3.9
1: 1000, $Zn(Oct)_2$: PE-6a	Friedman isoconversion analysis	65.6 ± 1.9
1: 50, $ZnCl_2$: PE-6a[10]	Friedman isoconversion analysis	70.2 ± 7.2

$Zn(Oct)_2$ Mechanism: PE-7b depolymerization

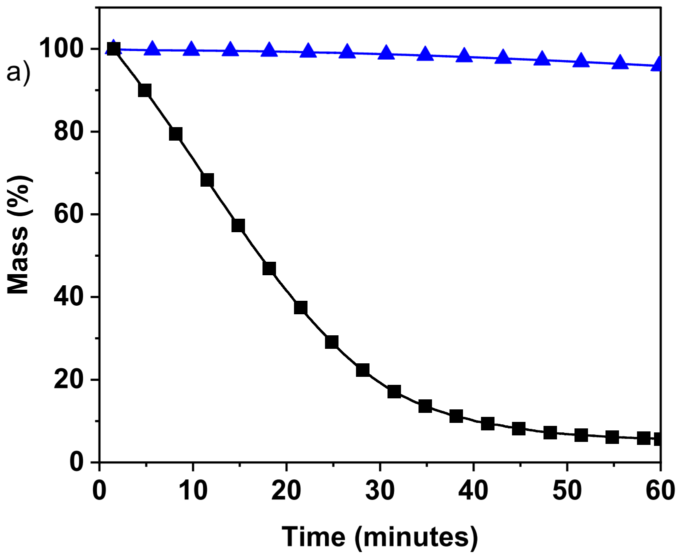

Figure S36. Depolymerization of hydroxyl (square, black) vs acetyl end-capped (triangle, blue) PE-7b. Reaction conducted at $[Zn(Oct)_2]_0$:$[PE\text{-}7b]_0$ = 1:100 at 130 °C.

Table S8. Depolymerization of PE-7b at various $Zn(Oct)_2$ loadings

Entry	Loading	k[a] (min^{-1})	a[a]	xc[a] (min)	$ka/4$ (secs)	k_{obs} [b] (s^{-1})	k_d [c](mol^{-1} dm^3 s^{-1})	TOF[d] (h^{-1})
1	1:100	0.165	0.901	13.32	0.000621	0.000650 ±0.00008	0.0042 ±0.0005	230 ±20
		0.192	0.951	12.03	0.000759			
		0.147	0.933	16.13	0.000571			
2	1:200	0.0834	0.882	28.52	0.000306	0.000314 ±0.000020	0.0040 ±0.0003	200 ±10
		0.0909	0.901	25.29	0.000342			
		0.0784	0.901	30.00	0.000294			
3	1:300	0.0613	0.873	37.73	0.000223	0.000214 ±0.000010	0.0041 ±0.0002	210 ±10
		0.0641	0.823	34.10	0.000220			
		0.0590	0.817	37.49	0.000201			
4	1:600	0.0508	0.681	40.61	0.000144	0.000124 ±0.000023	0.0048 ±0.0009	260 ±20
		0.0479	0.675	48.83	0.000135			
		0.0419	0.528	46.40	0.0000922			
5	1:1000	0.0374	0.518	53.48	0.0000801	0.0000802 ±0.00001	0.0051 ±0.0004	290 ±20
		0.0424	0.500	50.76	0.0000883			
		0.0378	0.457	53.71	0.0000720			

Reactions conducted at 130 °C by solvent casting $Zn(Oct)_2$:PE-7b solutions ($[PE\text{-}7b]_0$ = 7.80 M) in TGA crucibles. N_2 flow = 25 mL min^{-1}.[a] determined from logistic fitting of conversion *vs* time profiles from 0 – 90% mass loss.[b] taken as $k_d = k_{obs}/(2 \times [cat]_0)$, assuming 2 active chains per metal centre. [b] k_{obs} = average of 3 repeats, error = standard deviation from 3 repeats.[c] $k_p = k_{obs}/(2 \times [cat]_0)$, assuming 2 active chains per metal centre. [d] TOF = moles of PE-7b consumed/time * moles of catalyst. Determined from 0 – 30% conversion.

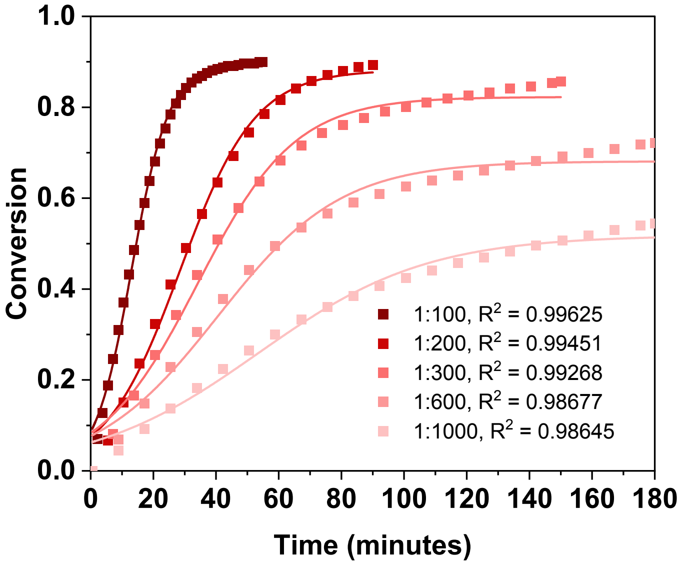

Figure S37. Plots of PE-7b conversion vs time using various $Zn(Oct)_2$ loadings. Experiments were conducted using 1:100, 1:200, 1:300, 1:600 and 1:1000 Zn:PE-7b loadings, at 130 °C; reactions were repeated in triplicate.

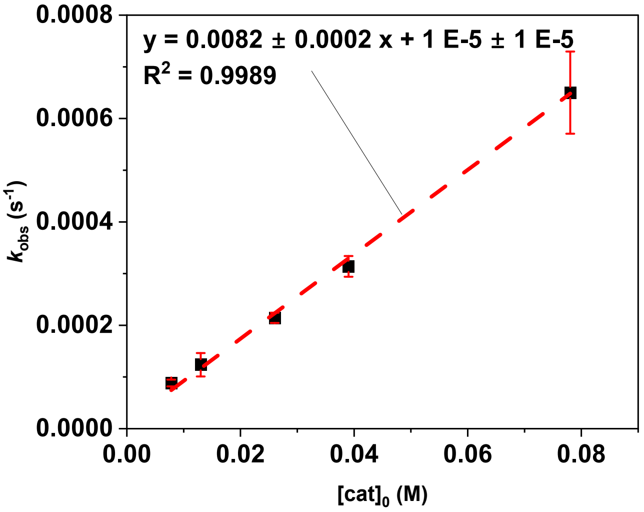

Figure S38. Determination of the dependence of rate of PE-7b recycling on catalyst concentration. Plots of k_{obs} vs $[Zn(Oct)_2]_0$; the errors are determined from triplicate runs as the standard deviations of the mean. Reactions were conducted at 130 °C.

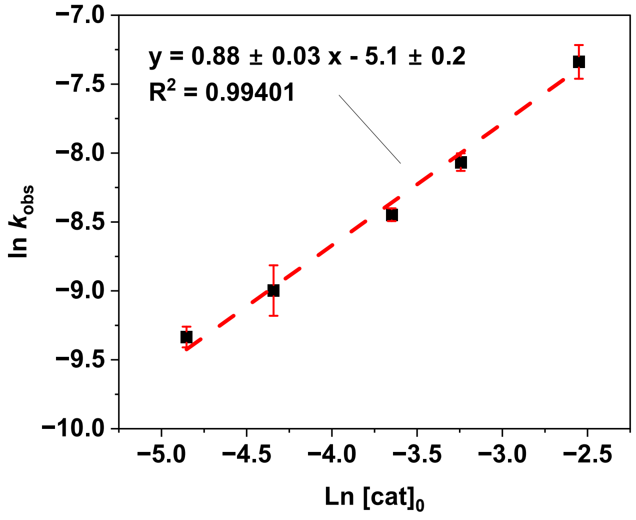

Figure S39. Determination of the dependence of rate of PE-7b recycling on initial catalyst concentration. Plots of ln(k_{obs}) *vs* ln([Zn(Oct)$_2$]$_0$); the errors are determined from triplicate runs as the standard deviations of the mean. Reactions were conducted at 130 °C.

Recycling of PE-6b

Table S9. Depolymerization of PE-6b at 90 – 130 °C with 1:1000 $[Zn(Oct)_2]_0$:$[PE\text{-}6b]_0$ loading

Entry	Temp. (°C)	k[a] (min^{-1})	a[a]	xc[a] (min)	$ka/4$ (secs)	k_{obs} [b] (s^{-1})	k_d [c] ($mol^{-1}\ dm^3\ s^{-1}$)	TOF[d] (h^{-1})
1	90	0.215	0.910	13.56	0.000816	0.000795 ±0.00013	0.045 ±0.007	1850 ±30
		0.206	0.906	13.36	0.000779			
		0.214	0.888	13.32	0.000790			
2	100	0.503	0.952	7.41	0.00200	0.00150 ±0.00036	0.086 ±0.021	2920 ±300
		0.304	0.915	9.78	0.00116			
		0.351	0.912	7.88	0.00133			
3	110	0.626	0.911	4.54	0.00238	0.00215 ±0.00022	0.12 ±0.01	5400 ±1300
		0.578	0.924	4.68	0.00223			
		0.467	0.950	7.37	0.00185			
4	120	1.14	0.956	3.10	0.00456	0.00458 ±0.00100	0.26 ±0.06	8060 ±250
		1.45	0.967	2.75	0.00583			
		1.05	0.775	2.78	0.00337			
5	130	2.19	0.944	1.62	0.00860	0.00973 ±0.00086	0.56 ±0.05	15900 ±1020
		2.52	0.944	1.47	0.00992			
		2.73	0.939	1.37	0.0107			

Reaction conducted by solvent casting $Zn(Oct)_2$: PE-6b solutions ($[Zn(Oct)_2]_0$: $[PE\text{-}6b]_0$ = 1: 1000, $[Zn(Oct)_2]_0 = 8.76 \times 10^{-3}$ M in bulk polymer) in TGA crucibles. N_2 flow = 25 mL min^{-1}.[a] determined from logistic fitting of conversion *vs* time profiles from 0 – 90% mass loss.[b] k_{obs} = average of 3 repeats, error = standard deviation from 3 repeats.[c] $k_d = k_{obs}/(2 \times [cat]_0)$, assuming 2 active chains per metal centre. [d] TOF = moles of PE-6b consumed/time * moles of catalyst. Determined from 0 – 30% conversion.

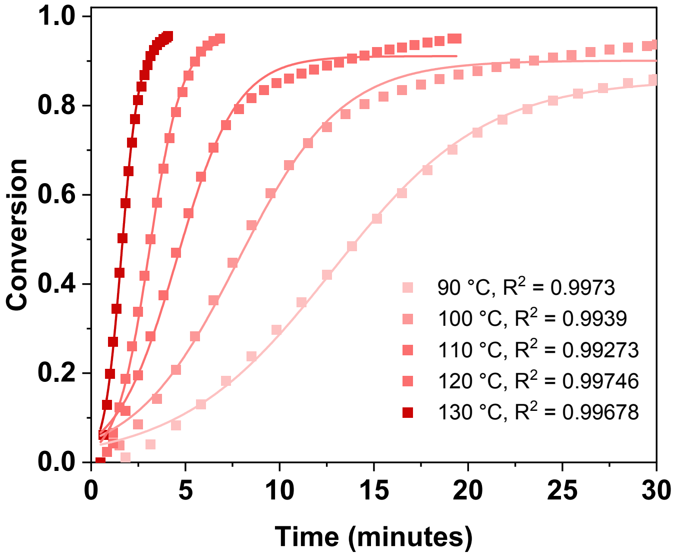

Figure S40. TGA thermograms showing experiments monitoring PE-6b conversion in $Zn(Oct)_2$: PE-6b mixtures (1:1000). The TGAs were run at 90, 100, 110, 120 and 130 °C for 1 h or until >95% conversion

Recycling of PE-6c

Table S10. Depolymerization of PE-6c at 110 – 150 °C with 1:100 [Zn(Oct)$_2$]$_0$:[PE-6c]$_0$ loading

Entry	Temp. (°C)	*k*[a] (min^{-1})	*a*[a]	*xc*[a] (min)	*ka*/4 (secs)	k_{obs} [b] (s^{-1})	k_d [c] (mol^{-1} dm^3 s^{-1})	TOF[d] (h^{-1})
1	110	0.0889	0.923	32.66	0.000342	0.000382 ±0.00003	0.0022 ±0.0002	80 ±10
		0.0994	0.923	29.05	0.000382			
		0.106	0.953	27.00	0.000423			
2	120	0.160	0.873	18.86	0.000583	0.000632 ±0.00005	0.0036 ±0.0003	130 ±10
		0.165	0.905	17.99	0.000623			
		0.174	0.949	17.37	0.000689			
3	130	0.433	0.917	7.34	0.00165	0.00168 ±0.00012	0.0096 ±0.0007	370 ±60
		0.532	0.830	5.29	0.00184			
		0.393	0.946	7.58	0.00155			
4	140	0.577	0.931	5.40	0.00224	0.00226 ±0.00006	0.013 ±0.001	460 ±15
		0.561	0.939	5.49	0.00220			
		0.600	0.934	5.11	0.00233			
5	150	1.24	0.921	2.63	0.00473	0.00465 ±0.0003	0.027 ±0.002	930 ±10
		1.20	0.916	2.62	0.00457			
		1.22	0.918	2.60	0.00465			

Reaction conducted by solvent casting Zn(Oct)$_2$:PE-6c solutions ([Zn(Oct)$_2$]$_0$: [PE-6c]$_0$ = 1: 100, [Zn(Oct)$_2$]$_0$ = 8.76 x 10^{-2} M in bulk polymer) in TGA crucibles. N_2 flow = 25 mL min^{-1}.[a] determined from logistic fitting of conversion *vs* time profiles from 0 – 90% mass loss.[b] k_{obs} = average of 3 repeats, error = standard deviation from 3 repeats.[c] k_d = k_{obs}/(2 x [cat]$_0$), assuming 2 active chains per metal centre. [d] TOF = moles of PE-6c consumed/time * moles of catalyst. Determined from 0 – 30% conversion

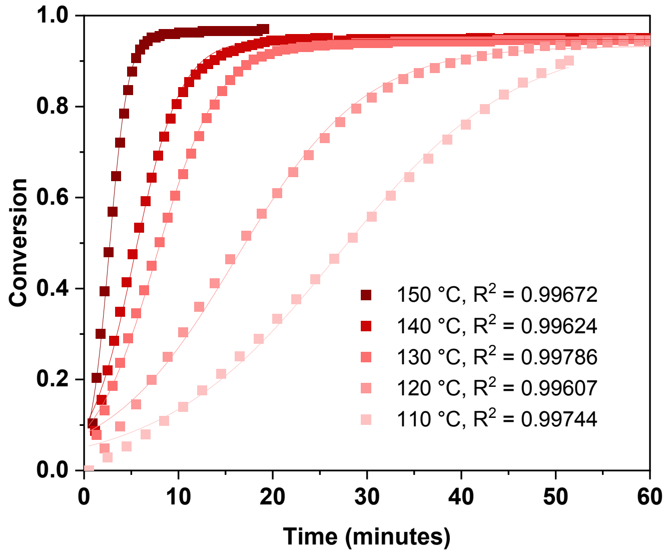

Figure S41. TGA thermograms showing experiments monitoring PE-6c conversion in Zn(Oct)$_2$:PE-6c mixtures (1:100). The TGAs were run at 110, 120, 130, 140 and 150 °C for 1 h or until >95% conversion

Recycling of PE-6d

Table S11. Depolymerization of PE-6d at 120 – 160 °C with 1:100 $[Zn(Oct)_2]_0$:$[PE\text{-}6d]_0$ loading

Entry	Temp. (°C)	k[a] (min^{-1})	a[a]	xc[a] (min)	$ka/4$ (secs)	k_{obs} [b] (s^{-1})	k_d [c] (mol^{-1} dm^3 s^{-1})	TOF[d] (h^{-1})
1	120	0.0634	0.881	45.70	0.000233	0.000233 ±0.000016	0.0020 ±0.0001	50 ±5
		0.0626	0.968	54.33	0.000252			
		0.0625	0.816	43.09	0.000213			
2	130	0.147	0.949	22.72	0.000579	0.000660 ±0.000057	0.0056 ±0.0005	130 ±20
		0.180	0.934	17.12	0.000698			
		0.174	0.965	17.69	0.000700			
3	140	0.316	0.968	10.55	0.00127	0.00126 ±0.00006	0.011 ±0.001	230 ±10
		0.332	0.955	9.81	0.00132			
		0.309	0.917	10.54	0.00118			
4	150	0.628	0.997	5.27	0.00261	0.00240 ±0.00037	0.020 ±0.003	450 ±80
		0.628	1.03	4.78	0.00271			
		0.476	0.947	6.99	0.00188			
5	160	1.04	0.981	3.40	0.00424	0.00412 ±0.00024	0.035 ±0.002	710 ±40
		1.082	0.963	3.16	0.00434			
		0.934	0.972	3.67	0.00378			

Reaction conducted by solvent casting $Zn(Oct)_2$:PE-6d solutions ($[Zn(Oct)_2]_0$: $[PE\text{-}6d]_0$ = 1: 100, $[Zn(Oct)_2]_0$ = 5.87 x 10^{-2} M in bulk polymer) in TGA crucibles. N_2 flow = 25 mL min^{-1}.[a] determined from logistic fitting of conversion *vs* time profiles from 0 – 90% mass loss. [b] k_{obs} = average of 3 repeats, error = standard deviation from 3 repeats.[c] $k_d = k_{obs}/(2 \times [cat]_0)$, assuming 2 active chains per metal centre. [d] TOF = moles of PE-6d consumed/time * moles of catalyst. Determined from 0 – 30% conversion

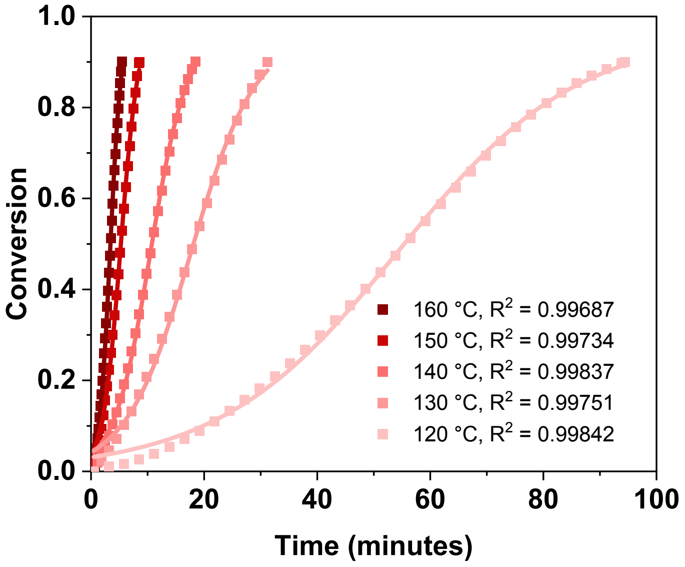

Figure S42. TGA thermograms showing experiments monitoring PE-6d conversion in $Zn(Oct)_2$:PE-6d mixtures (1:100). The TGAs were run at 120, 130, 140, 150, 160 for 2 h or until >95% conversion

Recycling of *rac*-PLA

Table S12. Depolymerization of *rac*-PLA at 120 – 170 °C with 1:100 [$Zn(Oct)_2$]$_0$:[*rac*-PLA]$_0$ loading

Entry	Temp. (°C)	*k*[a] (min^{-1})	*a*[a]	*xc*[a] (min)	*ka*/4 (secs)	k_{obs} [b] (s^{-1})	k_d [c] (mol^{-1} dm^3 s^{-1})	TOF[d] (h^{-1})
1	120	0.0235	0.153	64.80	0.0000150	0.0000137 ±0.000001	0.000049 ±0.000004	7 ±1
		0.0209	0.142	79.60	0.0000123			
		0.0219	0.151	72.70	0.0000138			
2	130	0.0278	0.322	75.30	0.0000373	0.0000383 ±0.000003	0.00014 ±0.00001	10 ±2
		0.0269	0.347	75.35	0.0000388			
		0.0265	0.350	72.83	0.0000386			
3	140	0.0310	0.438	44.82	0.0000566	0.0000492 0.0000056	0.00018 ±0.00002	20 ±2
		0.0266	0.392	61.00	0.0000433			
		0.0266	0.428	61.70	0.0000475			
4	150	0.0258	0.781	47.00	0.0000840	0.0000781 ±0.000007	0.00028 ±0.00003	70 ±10
		0.0256	0.769	47.19	0.0000821			
		0.0235	0.697	58.97	0.0000682			
5	160	0.0636	0.860	22.85	0.000228	0.000213 ±0.00001	0.00077 ±0.00004	160 ±20
		0.0584	0.869	24.15	0.000211			
		0.0554	0.864	23.92	0.000200			
6	170	0.115	0.778	14.52	0.000373	0.000372 ±0.000030	0.0013 ±0.0001	210 ±20
		0.112	0.871	15.13	0.000408			
		0.0921	0.872	17.82	0.000334			

Reaction conducted by solvent casting $Zn(Oct)_2$: *rac*-PLA solutions ([$Zn(Oct)_2$]$_0$: [*rac*-PLA]$_0$ = 1: 100, [$Zn(Oct)_2$]$_0$ = 0.1381 M in bulk polymer) in TGA crucibles. N_2 flow = 25 mL min^{-1}.[a] determined from logistic fitting of conversion *vs* time profiles from 0 – 90% mass loss[b] k_{obs} = average of 3 repeats, error = standard deviation from 3 repeats.[c] k_d = k_{obs}/(2 x [cat]$_0$), assuming 2 active chains per metal centre. [d] TOF = moles of *rac*-PLA consumed/time * moles of catalyst. Determined from 0 – 30% conversion

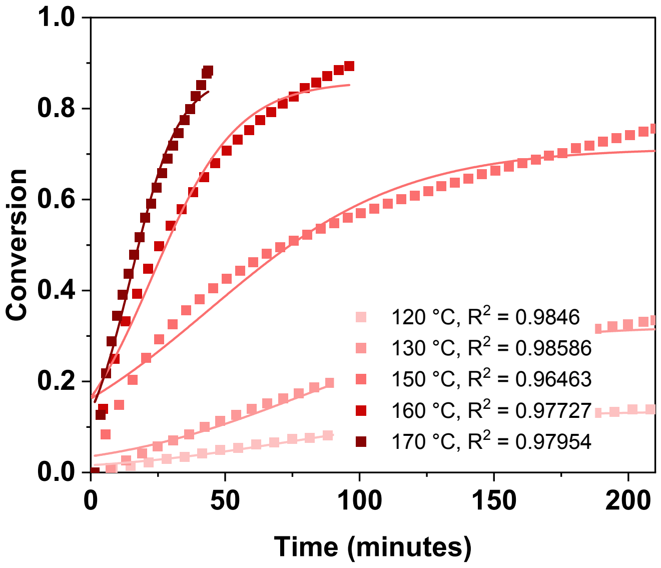

Figure S43. TGA thermograms showing experiments monitoring *rac*-PLA conversion in $Zn(Oct)_2$: *rac*-PLA mixtures (1:100). The TGAs were run at 120, 130, 150, 160 and 170 °C for 4 h or until >95% conversion

Recycling of PE-7a

Table S13. Depolymerization of PE-7a at 120 – 170 °C with 1:100 $[Zn(Oct)_2]_0$:$[PE\text{-}7a]_0$ loading

Entry	Temp. (°C)	k[a] (min^{-1})	a[a]	xc[a] (min)	$ka/4$ (secs)	k_{obs} [b] (s^{-1})	k_d [c] ($mol^{-1}\ dm^3\ s^{-1}$)	TOF[d] (h^{-1})
1	120	0.0390	0.642	66.53	0.000104	0.0000897 ±0.000012	0.00051 ±0.00007	30 ±3
		0.0371	0.487	65.90	0.0000753			
		0.0372	0.577	65.09	0.0000894			
2	130	0.0449	0.895	56.60	0.000167	0.000178 ±0.000025	0.0010 ±0.0001	60 ±10
		0.0556	0.918	42.50	0.000213			
		0.0447	0.831	53.03	0.000155			
3	150	0.0976	0.927	25.56	0.000377	0.000434 ±0.000040	0.0025 ±0.0002	120 ±10
		0.116	0.972	21.92	0.000470			
		0.115	0.953	21.46	0.000456			
4	160	0.246	0.923	9.73	0.000945	0.000752 ±0.00016	0.0043 ±0.0009	220 ±50
		0.137	0.933	16.99	0.000532			
		0.161	0.933	15.31	0.000627			
		0.241	0.897	21.66	0.000903			
5	170	0.296	0.938	8.76	0.00116	0.00181 ±0.00060	0.010 ±0.003	520 ±170
		0.694	0.898	3.41	0.00260			
		0.438	0.915	5.77	0.00167			

Reaction conducted by solvent casting $Zn(Oct)_2$:PE-7a solutions ($[Zn(Oct)_2]_0$: $[PE\text{-}7a]_0$ = 1: 100, $[Zn(Oct)_2]_0$ = 8.76x 10^{-2} M in bulk polymer) in TGA crucibles. N_2 flow = 25 mL min^{-1}.[a] determined from logistic fitting of conversion *vs* time profiles from 0 – 90% mass loss. [b] k_{obs} = average of 3 repeats, error = standard deviation from 3 repeats.[c] $k_d = k_{obs}/(2 \times [cat]_0)$, assuming 2 active chains per metal centre. [d] TOF = moles of PE-7a consumed/time * moles of catalyst. Determined from 0 – 30% conversion

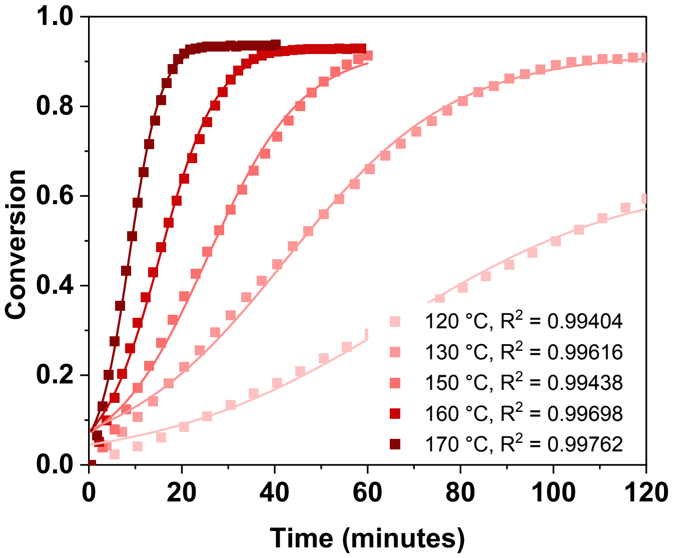

Figure S44. TGA thermograms showing experiments monitoring PE-7a conversion in $Zn(Oct)_2$:PE-7a mixtures (1:100). The TGAs were run at 120, 130, 150, 160 and 170 °C for 2 h or until >95% conversion

Recycling of PE-7b

Table S14. Depolymerization of at 120 – 160 °C with 1:100 $[Zn(Oct)_2]_0$:$[PE\text{-}7b]_0$ loading

Entry	Temp. (°C)	*k*[a] (min^{-1})	*a*[a]	*xc*[a] (min)	*ka*/4 (secs)	k_{obs} [b] (s^{-1})	k_d [c] (mol^{-1} dm^3 s^{-1})	TOF[d] (h^{-1})
1	120	0.0945	0.953	24.16	0.000375	0.000339 ±0.000058	0.0022 ±0.0004	120 ±20
		0.0984	0.940	23.84	0.000385			
		0.0686	0.899	32.62	0.000257			
2	130	0.165	0.901	13.32	0.000621	0.000650 ±0.000079	0.0042 ±0.0005	230 ±20
		0.192	0.951	12.03	0.000759			
		0.147	0.933	16.13	0.000571			
3	140	0.344	0.928	6.82	0.00133	0.00127 ±0.00007	0.0081 ±0.0004	380 ±20
		0.300	0.942	7.66	0.00118			
		0.318	0.972	7.82	0.00129			
4	150	0.415	0.944	5.35	0.00163	0.00186 ±0.00043	0.012 ±0.003	630 ±100
		0.615	0.972	3.98	0.00249			
		0.386	0.901	5.90	0.00145			
5	160	0.851	0.935	2.95	0.00332	0.00379 ±0.00033	0.024 ±0.002	1000 ±100
		1.02	0.953	2.48	0.00405			
		0.995	0.964	2.66	0.00400			

Reaction conducted by solvent casting $Zn(Oct)_2$:PE-7b solutions ($[Zn(Oct)_2]_0$: $[PE\text{-}7b]_0$ = 1: 100, $[Zn(Oct)_2]_0$ = 7.80 x 10^{-2} M in bulk polymer) in TGA crucibles. N_2 flow = 25 mL min^{-1}.[a] determined from logistic fitting of conversion *vs* time profiles from 0 – 90% mass loss. [b] k_{obs} = average of 3 repeats, error = standard deviation from 3 repeats.[c] k_d = k_{obs}/(2 x $[cat]_0$), assuming 2 active chains per metal centre. [d] TOF = moles of PE-7b consumed/time * moles of catalyst. Determined from 0 – 30% conversion

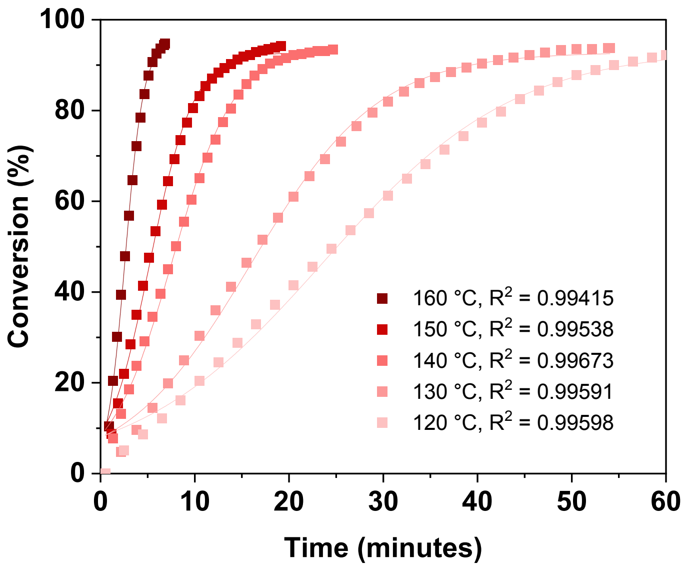

Figure S45. TGA thermograms showing experiments monitoring PE-7b conversion in $Zn(Oct)_2$:PE-7b mixtures (1:100). The TGAs were run at 120, 130, 140, 150 and 160 °C for 1 h or until >95% conversion

Recycling of PE-7c

Table S15. Depolymerization of PE-7c at 130 – 190 °C with 1:100 $[Zn(Oct)_2]_0$:[PE-7c]$_0$ loading

Entry	Temp. (°C)	*k*[a] (min^{-1})	*a*[a]	*xc*[a] (min)	*ka*/4 (secs)	k_{obs} [b] (s^{-1})	k_d [c] (mol^{-1} dm^3 s^{-1})	TOF[d] (h^{-1})
1	130	0.0423	0.132	57.17	0.00233	0.0000214 ±0.0000025	0.00014 ±0.00002	7 ±1
		0.0420	0.140	53.66	0.00244			
		0.0438	0.105	56.56	0.00192			
		0.0419	0.132	57.17	0.00186			
2	160	0.0485	0.590	43.49	0.0119	0.000129 ±0.000007	0.00083 ±0.00005	40 ±4
		0.0493	0.665	47.92	0.0137			
		0.0509	0.623	46.61	0.0132			
3	170	0.0591	0.878	31.59	0.0591	0.000195 ±0.000017	0.0013 ±0.0001	75 ±10
		0.0522	0.895	38.42	0.0522			
		0.0499	0.842	39.74	0.0499			
4	180	0.0921	0.922	19.27	0.0354	0.000315 ±0.000027	0.0020 ±0.0002	130 ±20
		0.0772	0.929	26.73	0.0299			
		0.0765	0.922	26.98	0.0294			
5	190	0.158	0.953	13.14	0.0628	0.000559 ±0.000085	0.0036 ±0.0005	210 ±20
		0.153	0.962	13.06	0.0611			
		0.122	0.865	17.18	0.0440			

Reaction conducted by solvent casting $Zn(Oct)_2$:PE-7c solutions ($[Zn(Oct)_2]_0$: [PE-7c]$_0$ = 1: 100, $[Zn(Oct)_2]_0$ = 7.80 x 10^{-2} M in bulk polymer) in TGA crucibles. N_2 flow = 25 mL min^{-1}.[a] determined from logistic fitting of conversion *vs* time profiles from 0 – 90% mass loss. [b] k_{obs} = average of 3 repeats, error = standard deviation from 3 repeats.[c] k_d = k_{obs}/(2 x [cat]$_0$), assuming 2 active chains per metal centre. [d] TOF = moles of PE-7c consumed/time * moles of catalyst. Determined from 0 – 30% conversion

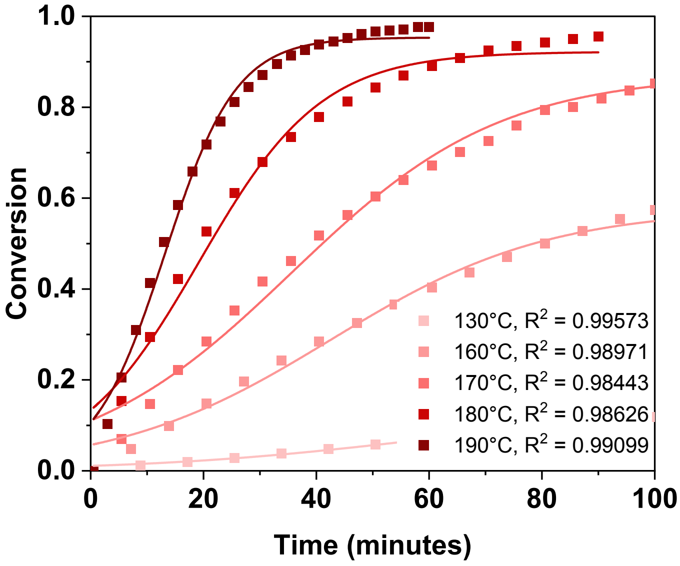

Figure S46. TGA thermograms showing experiments monitoring PE-7c conversion in $Zn(Oct)_2$:PE-7c mixtures (1:100). The TGAs were run at 130, 160, 170, 180 and 190 °C for 2 h minutes or >95% conversion

Recycling of PE-7d

Table S16. Depolymerization of PE-7d at 120 – 170 °C with 1:100 $[Zn(Oct)_2]_0$:$[PE\text{-}7d]_0$ loading

Entry	Temp. (°C)	*k*[a] (min^{-1})	*a*[a]	*xc*[a] (min)	*ka/4* (secs)	k_{obs} [b] (s^{-1})	k_d [c] (mol^{-1} dm^3 s^{-1})	TOF[c] (h^{-1})
1	130	0.0206	0.0947	89.25	0.00000812	0.00000762 ±3.8E-07	0.000062 ±0.000003	2 ±1[d]
		0.0239	0.0751	91.15	0.00000749			
		0.0236	0.0734	96.93	0.0000072			
2	150	0.0270	0.266	83.57	0.0000298	0.0000273 ±0.0000018	0.00023 ±0.00002	9 ±1[d]
		0.0262	0.236	86.47	0.0000257			
		0.0259	0.246	84.84	0.0000266			
3	160	0.0282	0.395	78.21	0.0000465	0.0000456 ±0.0000010	0.00039 ±0.00001	14 ±1
		0.0286	0.371	76.41	0.0000442			
		0.0288	0.385	73.94	0.0000462			
4	170	0.0311	0.564	67.73	0.0000731	0.0000698 ±0.0000038	0.00060 ±0.00003	25 ±3
		0.0314	0.493	65.47	0.0000645			
		0.0299	0.577	72.61	0.0000720			
5	180	0.0369	0.673	52.71	0.000103	0.0000991 ±0.0000079	0.00084 ±0.00007	40 ±4
		0.0377	0.674	50.76	0.000106			
		0.0342	0.619	53.71	0.0000881			

Reaction conducted by solvent casting $Zn(Oct)_2$:PE-7d solutions ($[Zn(Oct)_2]_0$: $[PE\text{-}7d]_0$ = 1: 100, $[Zn(Oct)_2]_0$ = 5.87 x 10^{-2} M in bulk polymer) in TGA crucibles. N_2 flow = 25 mL min^{-1}.[a] determined from logistic fitting of conversion *vs* time profiles from 0 – 3 h. [b] k_{obs} = average of 3 repeats, error = standard deviation from 3 repeats.[c] k_d = $k_{obs}/(2 \times [cat]_0)$, assuming 2 active chains per metal centre. [d] TOF = moles of PE-7d consumed/time * moles of catalyst. Determined from 0 – 30% conversion

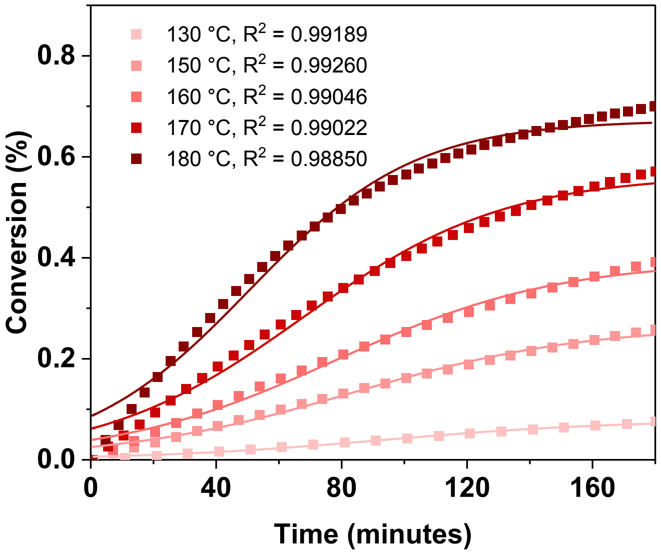

Figure S47. TGA thermograms showing experiments monitoring PE-7d conversion in $Zn(Oct)_2$:PE-7d mixtures (1:100). The TGAs were run at 130, 150, 160, 170 and 180 °C for 3 h

Recycling of PC-6a

Table S17. Depolymerization of PC-6a at 130 – 170 °C with 1:160 $[Zn(Oct)_2]_0$:$[PC-6a]_0$ loading

Entry	Temp. (°C)	k[a] (min^{-1})	a[a]	xc[a] (min)	$ka/4$ (secs)	k_{obs} [b] (s^{-1})	k_d[c] (mol^{-1} dm^3 s^{-1})	TOF[d] (h^{-1})
1	130	0.0263	0.897	71.33	9.82E-05	9.39E-05 ± 4.9E-06	0.0048 ± 0.0002	440 ±40
		0.0260	0.891	70.23	9.65E-05			
		0.0256	0.813	68.75	8.69E-05			
2	140	0.0454	0.882	39.73	0.000167	0.000177 ± 1.3E-05	0.0090 ± 0.0007	800±80
		0.0501	0.935	93.13	0.000195			
		0.0464	0.870	38.99	0.000168			
3	150	0.0921	0.940	22.46	0.000361	0.000319 ± 3.6E-05	0.016 ± 0.002	1400±140
		0.0828	0.932	23.22	0.000321			
		0.0706	0.930	24.30	0.000273			
4	160	0.119	0.923	14.15	0.000459	0.000551 ± 0.00012	0.028 ± 0.006	2400±240
		0.122	0.927	15.07	0.000472			
		0.182	0.950	11.85	0.000721			
5	170	0.329	0.943	6.53	0.00129	0.00132 ± 3.4E-05	0.067 ± 0.002	4700±470
		0.348	0.942	6.20	0.00136			
		0.326	0.953	6.82	0.00129			

Reaction conducted by solvent casting $Zn(Oct)_2$:PC-6a solutions ($[Zn(Oct)_2]_0$: $[PC-6a]_0$ = 1: 1000, $[Zn(Oct)_2]_0$ = 9.98 x 10^{-3} M in bulk polymer) in TGA crucibles. N_2 flow = 25 mL min^{-1}.[a] determined from logistic fitting of conversion *vs* time profiles from 0 – 90% mass loss. [b] k_{obs} = average of 3 repeats, error = standard deviation from 3 repeats.[c] k_d = k_{obs}/(2 x $[cat]_0$), assuming 2 active chains per metal centre. [d] TOF = moles of PC-6a consumed/time * moles of catalyst. Determined from 0 – 30% conversion

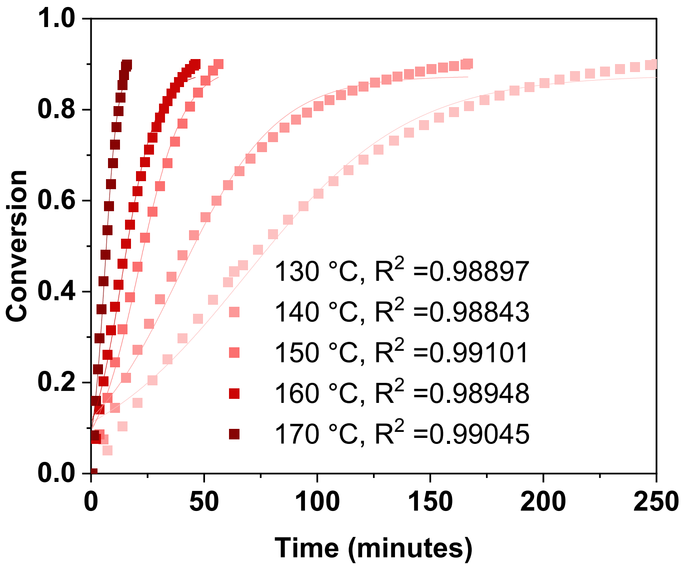

Figure S48. TGA thermograms showing experiments monitoring PC-6a conversion in $Zn(Oct)_2$:PC-6a mixtures (1:1000). The TGAs were run at 130, 140, 150, 160 and 170 °C until >95% conversion

Recycling of PC-6b

Table S18. Depolymerization of PC-6b at 100 – 160 °C with 1:160 [Zn(Oct)$_2$]$_0$:[PC-6b]$_0$ loading

Entry	Temp. (°C)	k[a] (min^{-1})	a[a]	xc[a] (min)	ka/4 (secs)	k_{obs} [b] (s^{-1})	k_d[c] (mol^{-1} dm^3 s^{-1})	TOF[d] (h^{-1})
1	120	0.0411	0.860	54.36	0.000147	0.000129 ± 1.4E-05	0.0084 ± 0.0009	420± 50
		0.0306	0.897	73.00	0.000114			
		0.0342	0.887	68.06	0.000126			
2	130	0.0708	0.916	36.49	0.000270	0.000271 ± 1.2E-05	0.018 ± 0.001	690± 70
		0.0670	0.919	38.21	0.000257			
		0.0752	0.914	31.17	0.000286			
3	140	0.107	0.926	24.43	0.000413	0.000484 ± 0.00012	0.031 ± 0.008	1300±130
		0.101	0.925	26.13	0.000391			
		0.173	0.896	14.61	0.000647			
4	150	0.272	0.917	10.05	0.00104	0.00109 ± 4.3E-05	0.071 ± 0.003	2600±260
		0.299	0.917	9.49	0.00114			
		0.288	0.918	9.96	0.00110			
5	160	0.481	0.923	6.03	0.00185	0.00178 ± 5.1E-05	0.12 ± 0.01	4100±400
		0.448	0.927	6.52	0.00173			
		0.457	0.921	6.17	0.00176			

Reaction conducted by solvent casting Zn(Oct)$_2$:PC-6b solutions ([Zn(Oct)$_2$]$_0$: [PC-6b]$_0$ = 1: 1000, [Zn(Oct)$_2$]$_0$ = 7.68 x 10^{-3} M in bulk polymer) in TGA crucibles. N$_2$ flow = 25 mL min^{-1}.[a] determined from logistic fitting of conversion *vs* time profiles from 0 – 90% mass loss. [b] k_{obs} = average of 3 repeats, error = standard deviation from 3 repeats.[c] k_d = k_{obs}/(2 x [cat]$_0$), assuming 2 active chains per metal centre. [d] TOF = moles of PC-6b consumed/time * moles of catalyst. Determined from 0 – 30% conversion

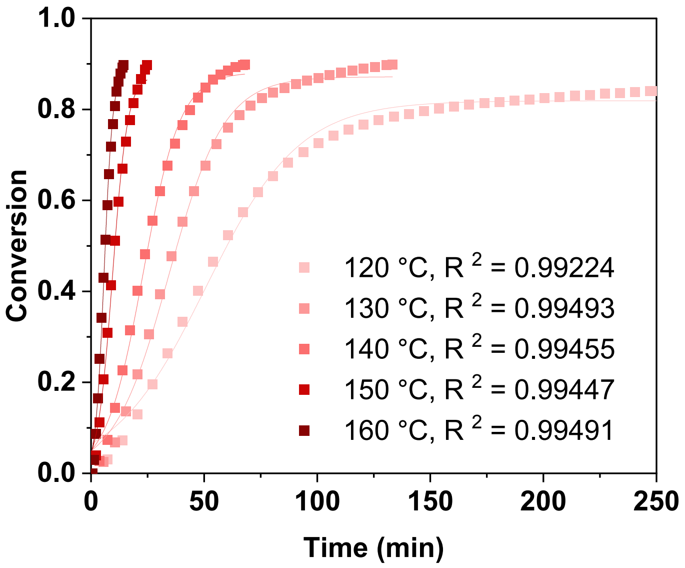

Figure S49. TGA thermograms showing experiments monitoring PC-6b conversion in Zn(Oct)$_2$:PC-6b mixtures (1:1000). The TGAs were run at 120, 130, 140, 150 and 160 °C until >95% conversion

Recycling of PC-6c

Table S19. Depolymerization of PC-6c at 100 – 160 °C with 1:160 $[Zn(Oct)_2]_0$:[PC-6c]$_0$ loading at various temperatures

Entry	Temp. (°C)	*k*[a] (min^{-1})	*a*[a]	*xc*[a] (min)	*ka/4* (secs)	k_{obs} [b] (s^{-1})	k_d[c] (mol^{-1} dm^3 s^{-1})	TOF[d] (h^{-1})
1	120	0.0132	0.844	139.08	4.65E-05	5.77E-05 ± 1.1E-05	0.0033 ± 0.0006	270± 40
		0.0180	0.916	105.21	6.88E-05			
2	130	0.0579	0.919	35.71	0.000222	0.000220 ± 1.3E-05	0.013 ± 0.001	870± 90
		0.0614	0.920	33.66	0.000235			
		0.0540	0.906	35.20	0.000204			
3	140	0.106	0.921	19.43	0.000407	0.000433 ± 1.9E-05	0.025 ± 0.001	1600± 160
		0.119	0.915	17.75	0.000452			
		0.115	0.922	19.20	0.000441			
4	150	0.201	0.920	10.33	0.000774	0.000730 ± 3.1E-05	0.042 ± 0.002	2800± 280
		0.184	0.930	11.41	0.000708			
		0.184	0.920	10.77	0.000708			
5	160	0.365	0.920	5.52	0.00141	0.001423 ± 3.1E-05	0.083 ± 0.001	5300± 530
		0.381	0.930	5.68	0.00147			
		0.363	0.930	5.89	0.00140			

Reaction conducted by solvent casting $Zn(Oct)_2$:PC-6c solutions ($[Zn(Oct)_2]_0$: [PC-6c]$_0$ = 1: 1000, $[Zn(Oct)_2]_0$ = 8.61 x 10^{-3} M in bulk polymer) in TGA crucibles. N_2 flow = 25 mL min^{-1}.[a] determined from logistic fitting of conversion *vs* time profiles from 0 – 90% mass loss. [b] k_{obs} = average of 3 repeats, error = standard deviation from 3 repeats.[c] k_d = k_{obs}/(2 x [cat]$_0$), assuming 2 active chains per metal centre. [d] TOF = moles of PC-6b consumed/time * moles of catalyst. Determined from 0 – 30% conversion

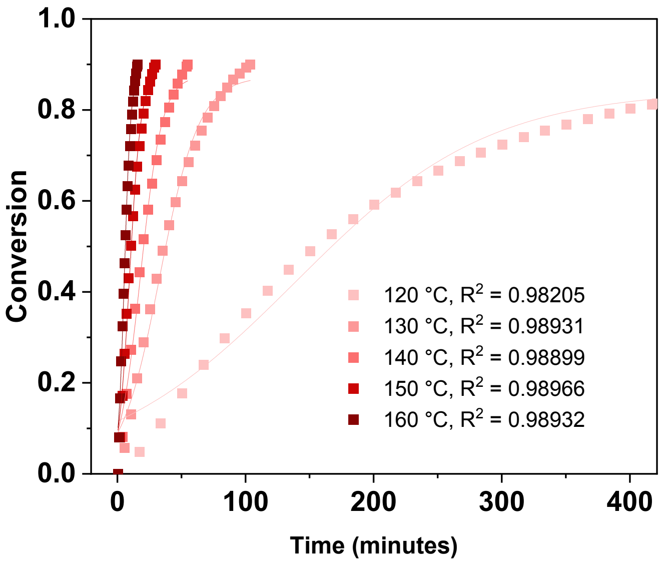

Figure S50. TGA thermograms showing experiments monitoring PC-6c conversion in $Zn(Oct)_2$:PC-6c mixtures (1:1000). The TGAs were run at 120, 130, 140, 150 and 160 °C until >95% conversion

TGA-FTIR data

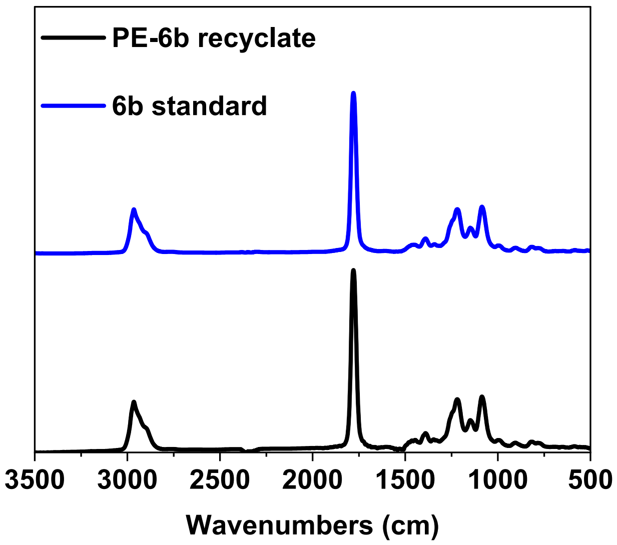

Figure S51. TGA-FTIR gas-phase analysis of PE-6b depolymerization mixture catalysed by $Zn(Oct)_2$ ($[Zn(Oct)_2]_0$:$[PE\text{-}6b]_0$ = 1:1000, 130 °C, black) and an 6b standard (blue).

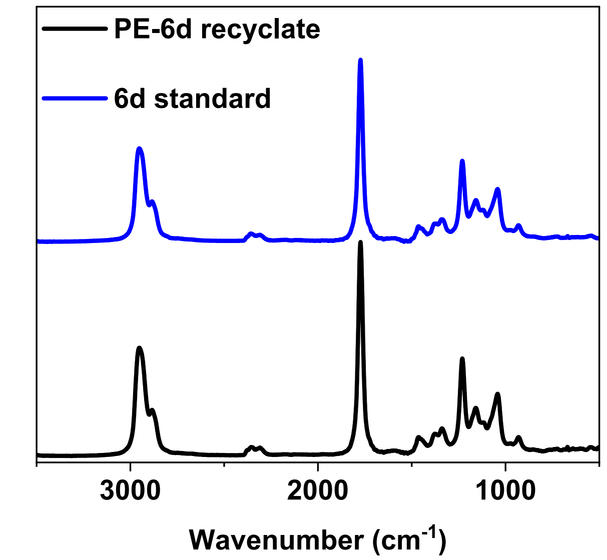

Figure S52. TGA-FTIR gas-phase analysis of PE-6d depolymerization mixture catalysed by $Zn(Oct)_2$ ($[Zn(Oct)_2]_0$:$[PE\text{-}6d]_0$ = 1:100, 130 °C, black) and an 6d standard (blue).

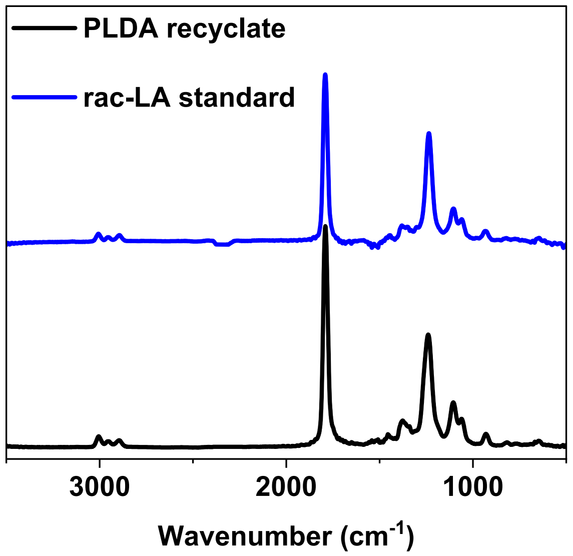

Figure S53. TGA-FTIR gas-phase analysis of *rac*-PLA depolymerization mixture catalysed by $Zn(Oct)_2$ ($[Zn(Oct)_2]_0$:[*rac*-PLA]$_0$ = 1:100, 130 °C, black) and a rac-Lactide standard (blue).

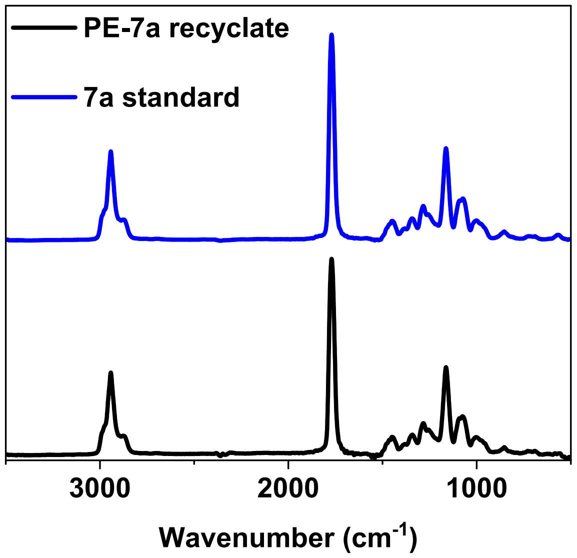

Figure S54. TGA-FTIR gas-phase analysis of PE-7a depolymerization mixture catalysed by $Zn(Oct)_2$ ($[Zn(Oct)_2]_0$:[PE-7a]$_0$ = 1:100, 160 °C, black) and an 7a standard (blue).

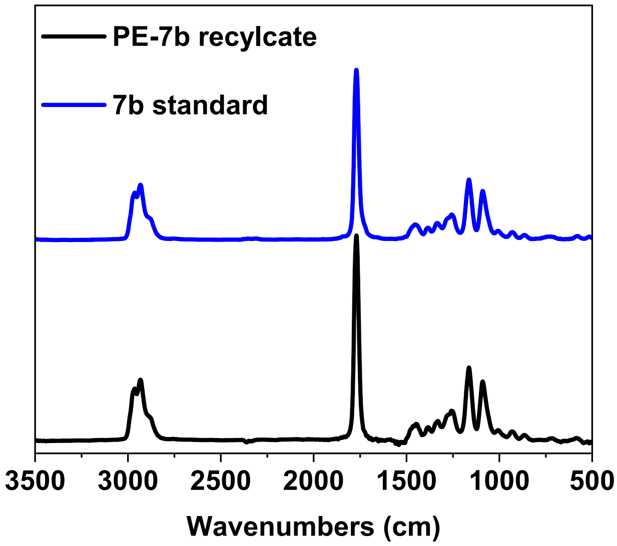

Figure S55. TGA-FTIR gas-phase analysis of PE-7b depolymerization mixture catalysed by $Zn(Oct)_2$ ($[Zn(Oct)_2]_0$:$[PE\text{-}7b]_0$ = 1:100, 160 °C, black) and 7b standard (blue).

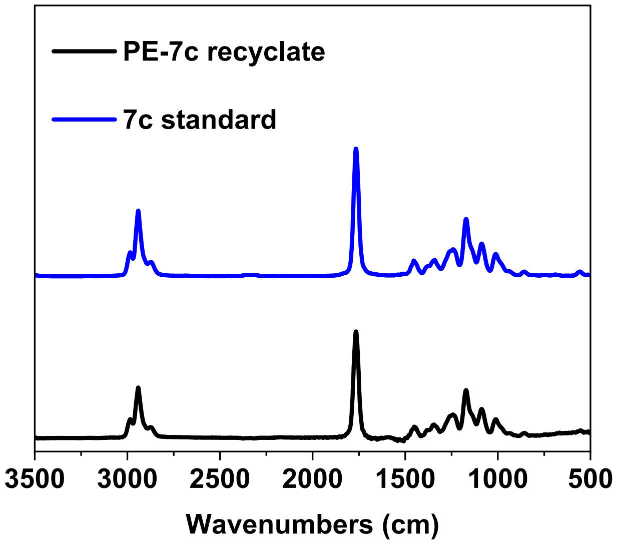

Figure S56. TGA-FTIR gas-phase analysis of PE-7c depolymerization mixture catalysed by $Zn(Oct)_2$ ($[Zn(Oct)_2]_0$:$[PE\text{-}7c]_0$ = 1:100, 190 °C, black) and 7c standard (blue).

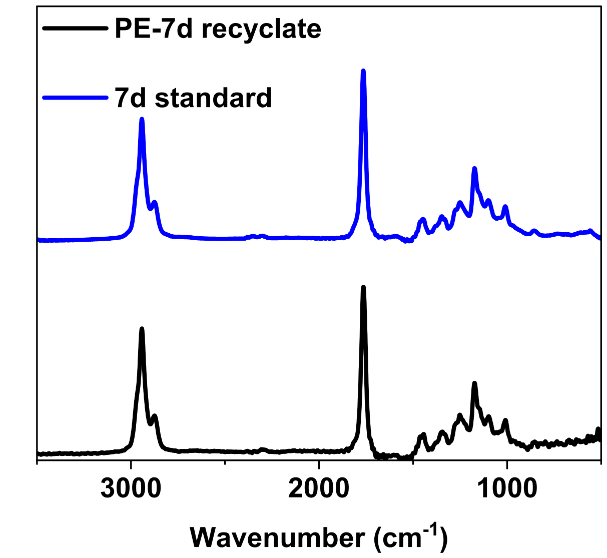

Figure S57. TGA-FTIR gas-phase analysis of PE-7d depolymerization mixture catalysed by $Zn(Oct)_2$ ($[Zn(Oct)_2]_0$:$[PE\text{-}7d]_0$ = 1:100, 190 °C, black) and PE-7d standard (blue).

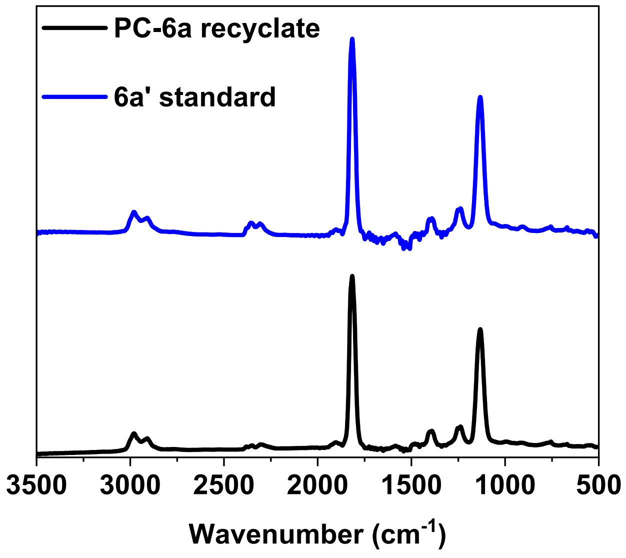

Figure S58. TGA-FTIR gas-phase analysis of PC-6a depolymerization mixture catalysed by $Zn(Oct)_2$ ($[Zn(Oct)_2]_0$:$[PC\text{-}6a]_0$ = 1:1000, 160 °C, black) and PC-6a standard (blue).

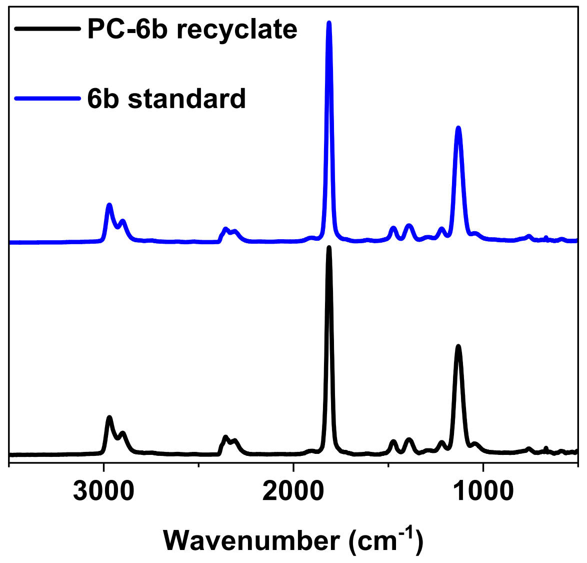

Figure S59. TGA-FTIR gas-phase analysis of PC-6b depolymerization mixture catalysed by $Zn(Oct)_2$ ($[Zn(Oct)_2]_0$:$[PC\text{-}6b]_0$ = 1:1000, 160 °C, black) and 6b' standard (blue).

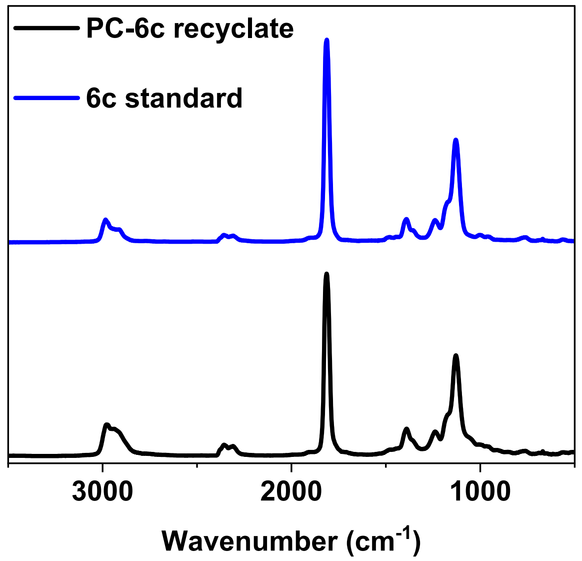

Figure S60. TGA-FTIR gas-phase analysis of PC-6c depolymerization mixture catalysed by $Zn(Oct)_2$ ($[Zn(Oct)_2]_0$:$[PC\text{-}6c]_0$ = 1:1000, 160 °C, black) and 6c' standard (blue).

Table S20. Summary of depolymerization data for all polymers at 130 °C

Entry	Polymer	Loading	$[Zn(Oct)_2]_0$[a]	k_{obs} [b] (s^{-1})	k_d [c] (mol^{-1} dm^3 s^{-1})	TOF[d] (h^{-1})
1	PE-6a	1: 1000	9.98 x 10^{-3}	0.00267 ±0.00029	0.13 ±0.01	7000 ±400
2	PE-6b	1: 1000	8.76 x 10^{-3}	0.00973 ±0.00086	0.56 ±0.05	15900 ±1020
3	PE-6c	1: 1000	8.76 x 10^{-2}	0.00168 ±0.00012	0.0096 ±0.0007	370 ±60
4	PE-6d	1: 1000	5.87 x 10^{-2}	0.000660 ±0.000057	0.0056 ±0.0005	130 ±20
5	PE-7a	1: 100	8.76 x 10^{-2}	0.000178 ±0.000025	0.0010 ±0.0001	60 ±10
6	PE-7b	1: 100	7.80 x 10^{-2}	0.00417 ±0.00051	0.0042 ±0.0005	230 ±20
7	PE-7c	1: 100	7.80 x 10^{-2}	0.0000214 ±2.5E-07	0.00014 ±0.00002	7 ±1
8	PE-7d	1: 100	5.87 x 10^{-2}	0.00000762 ±3.8E-07	0.000062 ±0.000003	2 ±1[d]
9	PC-6a	1: 1000	9.79 x 10^{-3}	0.0000939 ± 4.9E-06	0.0048 ± 0.0002	440 ±40
10	PC-6b	1: 1000	7.68 x 10^{-3}	0.000271 ± 1.2E-05	0.018 ± 0.001	690± 70
11	PC-6c	1: 1000	8.61 x 10^{-3}	0.000220 ± 1.3E-05	0.013 ± 0.001	870± 90

Reactions conducted at 130 °C by solvent casting $Zn(Oct)_2$:polymer solutions solutions in TGA crucibles. N_2 flow = 25 mL min^{-1}.[a] Concentrations assume polymer density is 1 g cm^{-3}.[b] Determined from logistic fitting of conversion *vs* time profiles from 0 – 90% mass loss, k_{obs} = average of 3 repeats, error = standard deviation from 3 repeats.[c] k_d = $k_{obs}/(2 \times [cat]_0)$, assuming 2 active chains per metal centre. [d] TOF = moles of polymer consumed/time * moles of catalyst. Determined from 0 – 30% conversion

Eyring analysis

Table S21: Recycling barriers: ΔH‡, ΔS‡ and ΔG‡ determined from Eyring analysis

Entry	Polymer	y = mx + c[a]	$R^{2[a]}$	ΔH‡ [b] (kJ mol^{-1})	ΔS‡ [c] (J mol^{-1})	ΔG‡ [d] (kJ mol^{-1})
1	PE-6a	-7453 ± 568<br>10.4 ± 1.4	0.98722	62.0 ± 4.7	-111 ± 15	107 ± 8
2	PE-6b	-9205 ± 858<br>16.2 ± 2.2	0.97461	76.5 ± 7.1	-63 ± 9	102 ± 8
3	PE-6c	-9844 ± 876<br>13.6 ± 2.2	0.97681	81.8 ±7.3	-85 ± 14	116 ± 9
4	PE-6d	-11552 ± 719<br>17.3 ± 1.7	0.98851	96.0 ± 6.0	-53 ± 5	118 ± 6
5	*rac*-PLA	-10731 ± 719<br>11.5 ± 1.7	0.98207	89.2 ± 6.0	-102 ± 15	130 ± 9
6	PE-7a	-8586 ± 650<br>8.3 ± 1.6	0.98121	71.4 ± 5.4	-128 ± 24	123 ± 11
7	PE-7b	-9699 ± 368<br>12.6 ± 0.9	0.9957	80.6 ± 3.1	-93 ± 7	118 ± 4
8	PE-7c	-9378 ± 380<br>8.4 ± 0.9	0.99510	78.0 ± 3.2	-127 ± 13	129 ± 6
9	PE-7d	-9408 ± 531<br>7.8 ± 1.2	0.99052	78.2 ± 4.4	-133 ± 21	132 ± 10
10	PC-6a	-11500 ± 269<br>17.2 ± 1.6	0.99836	95.6 ± 2.2	-55 ± 5	118 ± 3
11	PC-6b	-10620 ± 408<br>16.3 ± 2.1	0.99599	88.3 ± 3.4	-61 ± 8	113 ± 5
12	PC-6c	-10620 ± 408<br>15.7 ± 1.6	0.99628	87.3 ± 3.1	-67 ± 7	114 ± 4

[a] Taken from Figs. S57 – S69. [a] ΔH‡ = - m x 8.314. [b] ΔS‡ = 8.314 (c - ln k_b/h) where k_b = Boltzmann constant, h = planck constant [c] ΔG‡ = ΔH‡ - 403.14 x ΔS‡

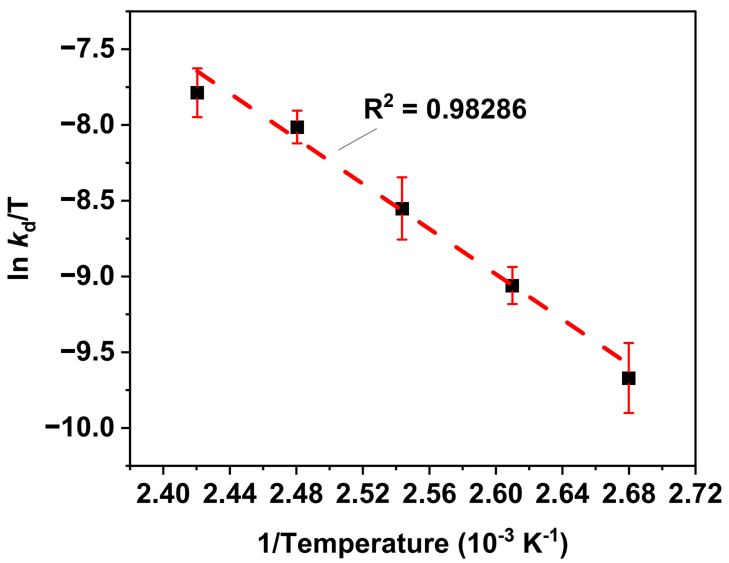

Figure S61. Plot of ln (k_d/T) *vs* 1/T for the recycling of PE-6a. Reactions were performed at 100, 110, 120, 130 and 140 °C with loadings of $[Zn(Oct)_2]_0$: $[PE\text{-}6a]_0$ of 1:1000. $[Zn(Oct)_2]_0$ = 9.98 x10^{-3} M

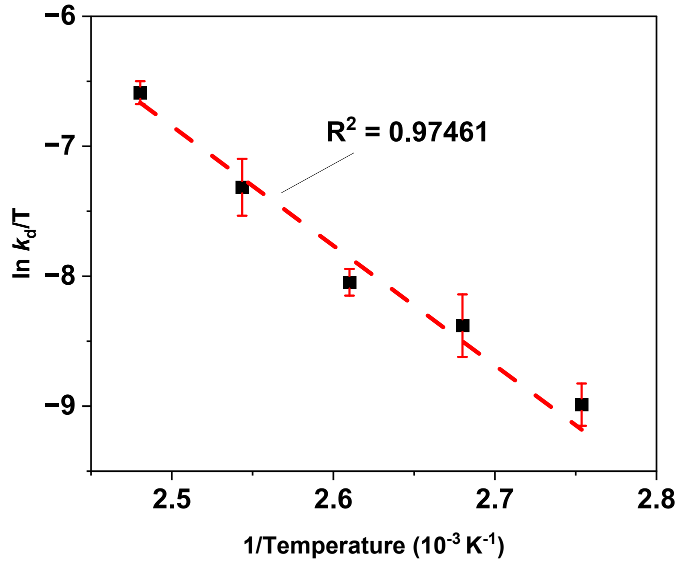

Figure S62. Plot of ln (k_d/T) *vs* 1/T for the recycling of PE-6b. Reactions were performed at 90, 100, 110, 120 and 130 °C with loadings of $[Zn(Oct)_2]_0$: $[PE\text{-}6b]_0$ of 1:1000. $[Zn(Oct)_2]_0$ = 8.76 x10^{-3} M

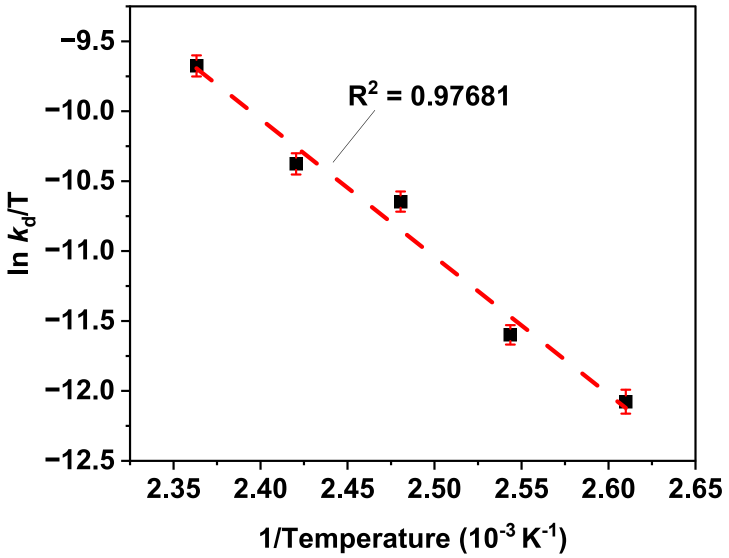

Figure S63. Plot of ln (k_d/T) *vs* 1/T for the recycling of PE-6c. Reactions were performed at 90, 110, 120, 130 and 140 °C with loadings of $[Zn(Oct)_2]_0$: $[PE\text{-}6c]_0$ of 1:100. $[Zn(Oct)_2]_0$ = 8.76 x10^{-2} M

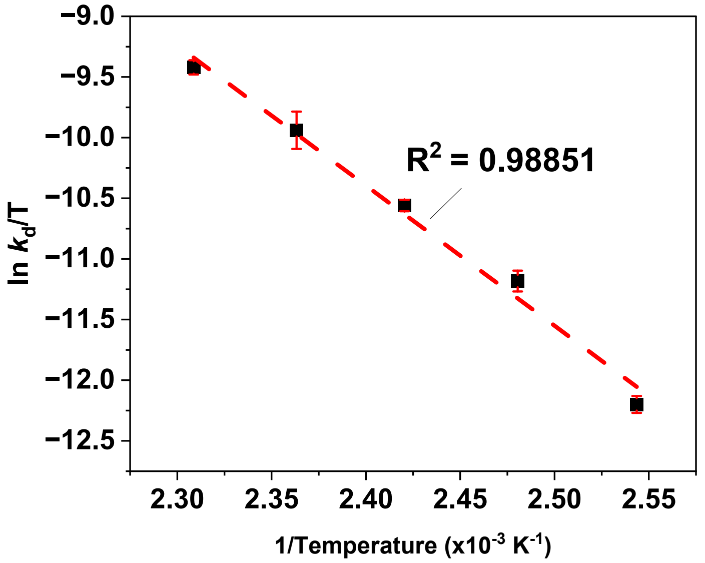

Figure S64. Plot of ln (k_d/T) *vs* 1/T for the recycling of PE-6d. Reactions were performed at 120, 130, 140, 150 and 160 °C with loadings of $[Zn(Oct)_2]_0$: $[PE\text{-}6d]_0$ of 1:100. $[Zn(Oct)_2]_0$ = 5.87 x10^{-2} M

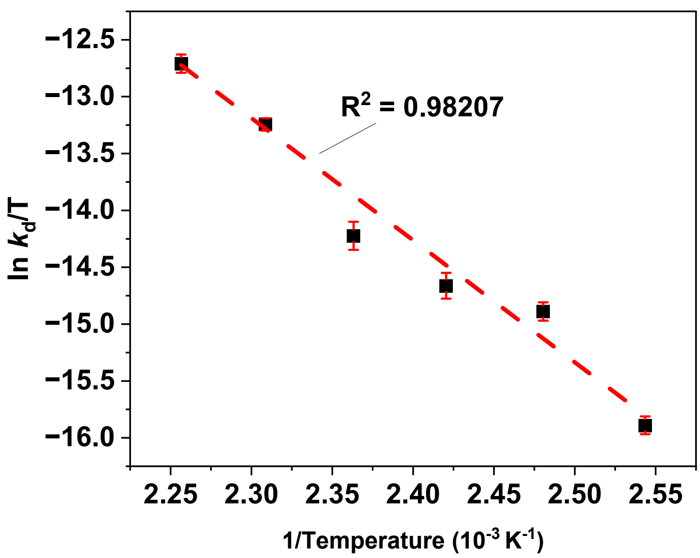

Figure S65. Plot of ln (k_d/T) *vs* 1/T for the recycling of *rac*-PLA. Reactions were performed at 120, 130, 150, 160 and 170 °C with loadings of $[Zn(Oct)_2]_0$: [*rac*-PLA]$_0$ of 1:100. $[Zn(Oct)_2]_0$ = 0.138 M

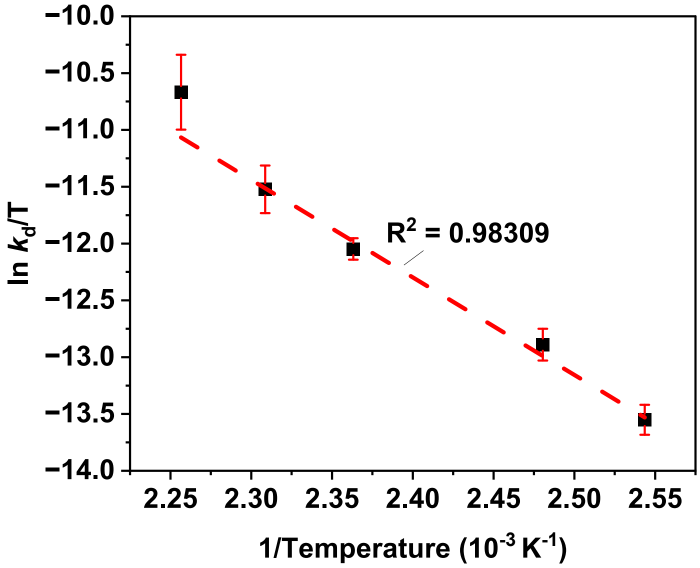

Figure S66. Plot of ln (k_d/T) *vs* 1/T for the recycling of PE-7a. Reactions were performed at 120, 130, 150, 160 and 170 °C with loadings of $[Zn(Oct)_2]_0$: [PE-7a]$_0$ of 1:100. $[Zn(Oct)_2]_0$ = 8.76 x 10^{-2} M

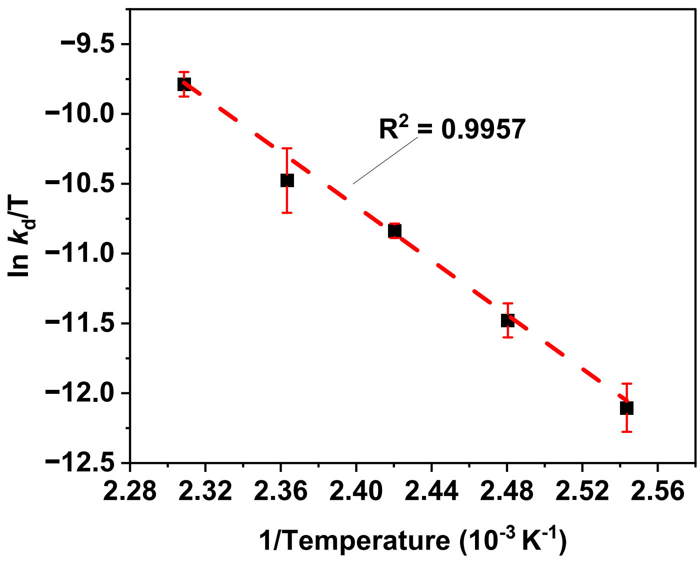

Figure S67. Plot of ln (k_d/T) *vs* 1/T for the recycling of PE-7b. Reactions were performed at 120, 130, 140, 150 and 160 °C with loadings of $[Zn(Oct)_2]_0$: $[PE\text{-}7b]_0$ of 1:100. $[Zn(Oct)_2]_0$ = 7.80 x 10^{-2} M

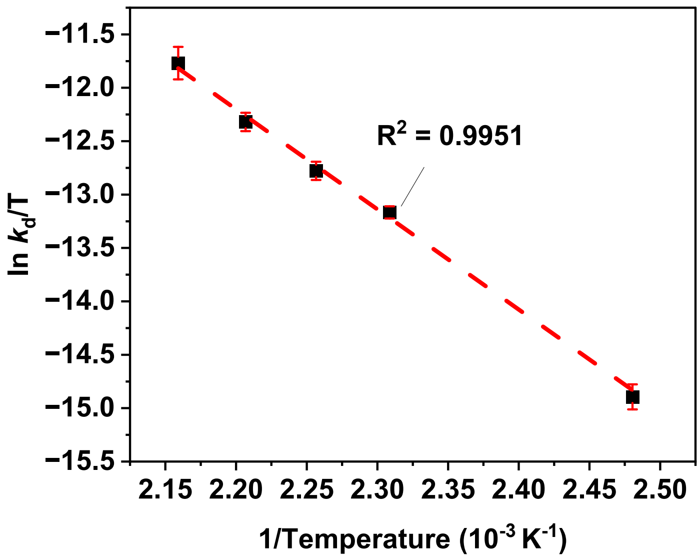

Figure S68. Plot of ln (k_d/T) *vs* 1/T for the recycling of PE-7c. Reactions were performed at 130, 160, 170, 180 and 190 °C with loadings of $[Zn(Oct)_2]_0$: $[PE\text{-}7c]_0$ of 1:100. $[Zn(Oct)_2]_0$ = 7.80 x 10^{-2} M

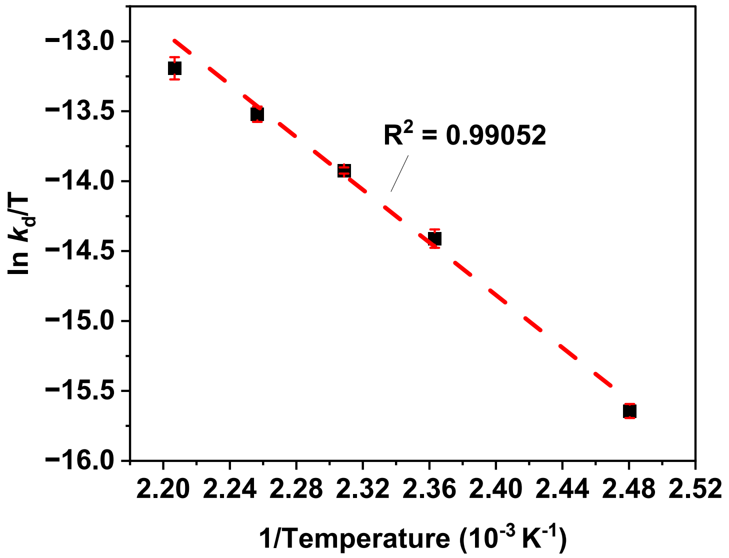

Figure S69. Plot of ln (k_d/T) *vs* 1/T for the recycling of PE-7d. Reactions were performed at 130, 150, 160, 170 and 180 °C with loadings of $[Zn(Oct)_2]_0$: $[PE\text{-}7d]_0$ of 1:100. $[Zn(Oct)_2]_0$ = 5.87 x 10^{-2} M

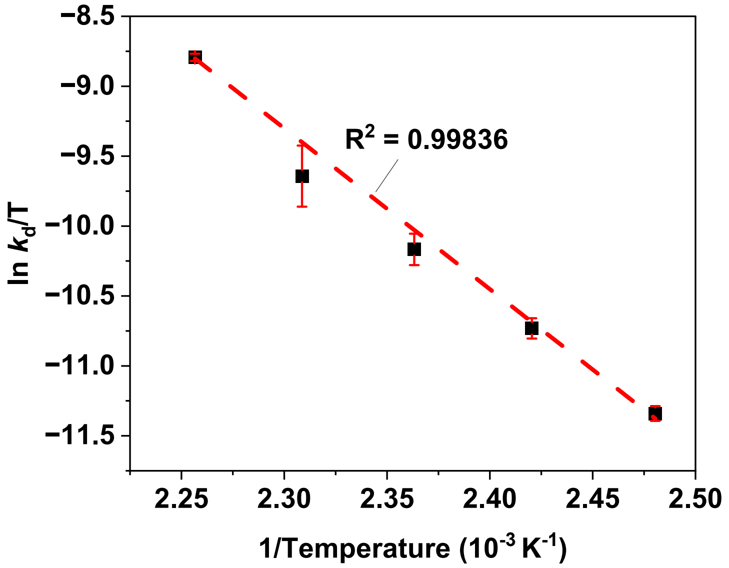

Figure S70. Plot of ln (k_d/T) *vs* 1/T for the recycling of PC-6a. Reactions were performed at 130, 140, 150, 160 and 170 °C with loadings of $[Zn(Oct)_2]_0$: $[PC\text{-}6a]_0$ of 1:1000. $[Zn(Oct)_2]_0$ = 9.80 x 10^{-3} M

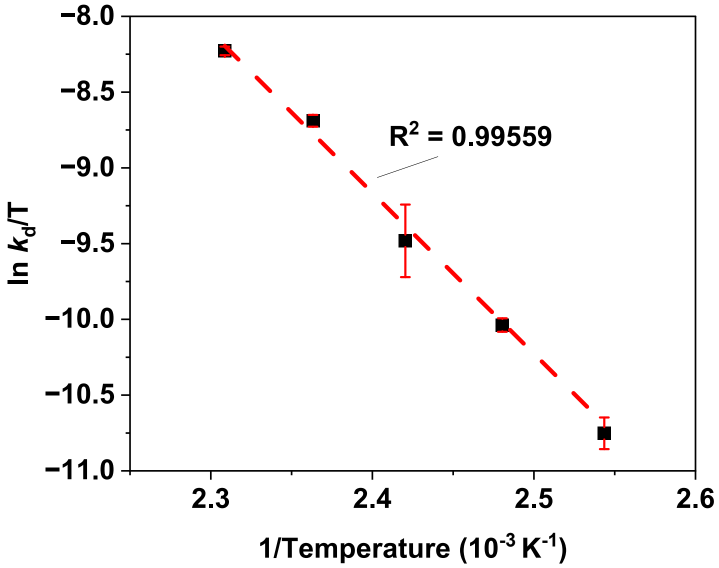

Figure S71. Plot of ln (k_d/T) *vs* 1/T for the recycling of PC-6b. Reactions were performed at 100, 120, 130, 140 and 160 °C with loadings of [Zn(Oct)$_2$]$_0$: [PC-6b]$_0$ of 1:1000. [Zn(Oct)$_2$]$_0$ = 7.68 x 10^{-2} M

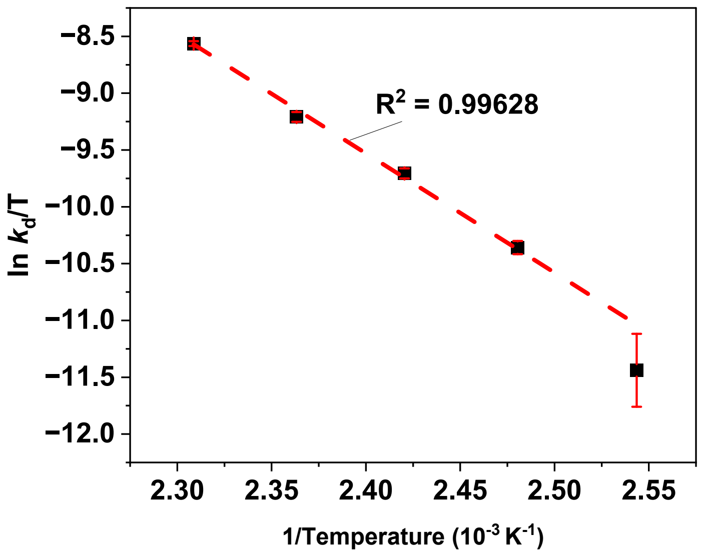

Figure S72. Plot of ln (k_d/T) *vs* 1/T for the recycling of PC-6c. Reactions were performed at 120, 130, 140, 150 and 160 °C with loadings of [Zn(Oct)$_2$]$_0$: [PC-6c]$_0$ of 1:1000. [Zn(Oct)$_2$]$_0$ = 8.61 x 10^{-3} M

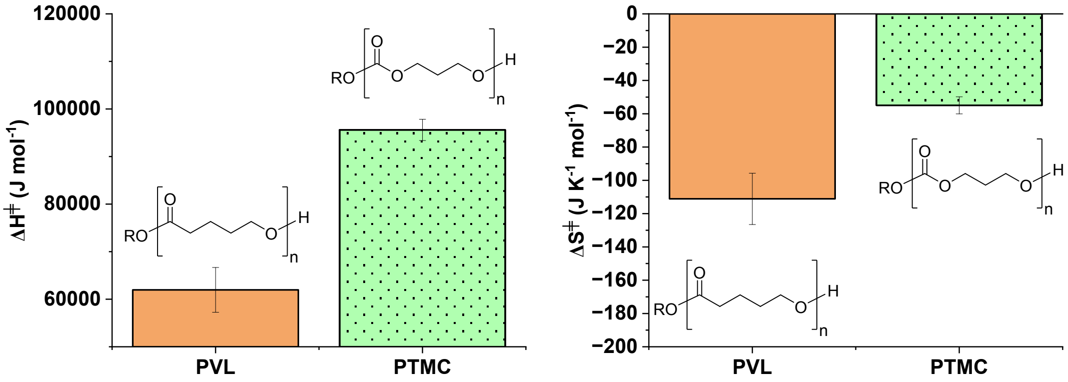

Figure S73. Comparison of Δ*H*‡ and Δ*S*‡ for PE-6a vs PC-6a. Given the comparable Δ*S*‡ terms, the overall lower barrier heights observed at 130 °C for PC-6a are driven by a decrease in the Δ*H*‡ term

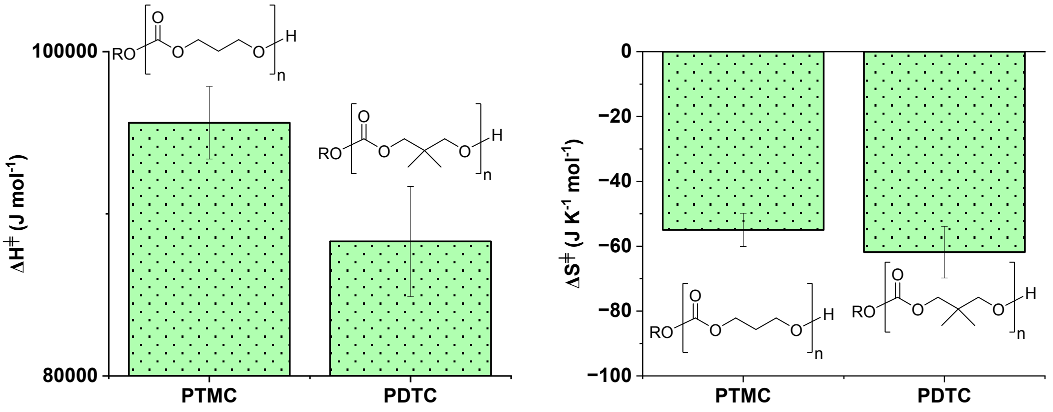

Figure S74. Comparison of Δ*H*‡ and Δ*S*‡ for PC-6a and PC-6b. Given the comparable Δ*H*‡ terms, the overall lower barrier heights observed at 130 °C for PC-6a are driven by a decrease in the Δ*S*‡ term

DFT

Density Functional theory (DFT) calculations were performed using Gaussian16 suite of codes (revision C.01).[11] Geometries were fully optimised without any symmetry or geometry constraints. The nature of all the stationary points as minima or transition states (first-order saddle points) on the potential energy surface was verified by calculations of the vibrational frequency spectrum. Geometry optimisations were carried out using the m06l functional with empirical dispersion correction factor, GD3, applied.[12] The 6-31+G(d) basis set was used for O atoms and the 6-31G(d,p) basis set was used for C and H atoms.[13] The SDD pseudopotential was used for Zn atoms and the solvent effects were modelled using conductor-like polarizable continuum model in ethyl acetate.[14] Goodvibes software[15] was used to apply temperature and concentration correction factors to calculated free enthalpies at 403.14 K and $[Zn]_0$ = 9.98 x 10^{-3} M for methyl 5-hydroxypentanoate and 5-methoxy-5-oxopentyl 5-hydroxypentanoate and $[Zn]_0$ = 8.76 x 10^{-2} M for methyl 6-hydroxyhexanoate. All transition state structures and intermediates can be found on figshare using the following DOI: 10.6084/m9.figshare.29110646.

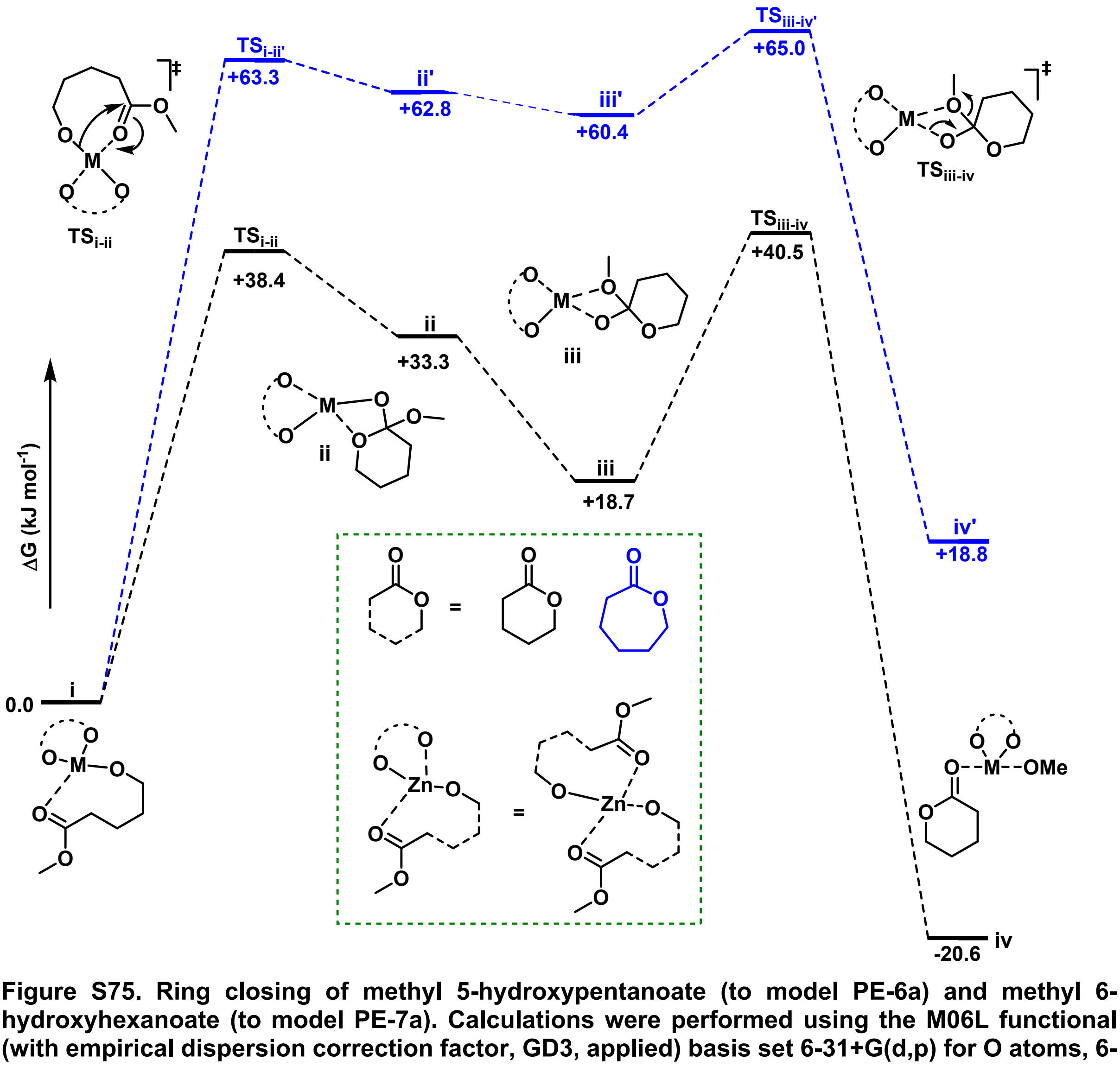

Figure S75. Ring closing of methyl 5-hydroxypentanoate (to model PE-6a) and methyl 6-hydroxyhexanoate (to model PE-7a). Calculations were performed using the M06L functional (with empirical dispersion correction factor, GD3, applied) basis set 6-31+G(d,p) for O atoms, 6-31G(d,p) for C and H atoms, and basis sets and pseudo potential SDD for Zn atoms. Solvent effects were modelled using the CPCM continuum model for ethylacetate. Goodvibes temperature and concentration factors applied.

Table S22. Computed intermediates and transition states for the backbiting of methyl 5-hydroxypentanoate and methyl 6-hydroxyhexanoate

Structure	G (Hartree)	G temperature corrected (Hartree)[a]	ΔG‡ (kJ mol^{-1})
**Methyl 5-hydroxypentanoate ring closure**			
i	-1148.722474	-1148.746709	0
TSi-ii	-1148.708652	-1148.732103	38.4
ii	-1148.71084	-1148.734038	33.3
iii	-1148.716673	-1148.739605	18.7
TSiii-iv	-1148.707479	-1148.731287	40.5
iv	-1148.731536	-1148.754543	-20.6
**Methyl 6-hydroxyhexanoate ring closure**			
i’	-1227.318831	-1227.318831	0
TSi-ii’	-1227.294726	-1227.294726	63.3
ii’	-1227.272558	-1227.295844	62.8
iii’	-1227.295844	-1227.29407	60.4
TSiii-iv’	-1227.29407	-1227.311669	65.0
iv’	-1227.311669	-1227.295844	18.8

[a]Temperature correction using goodvibes software

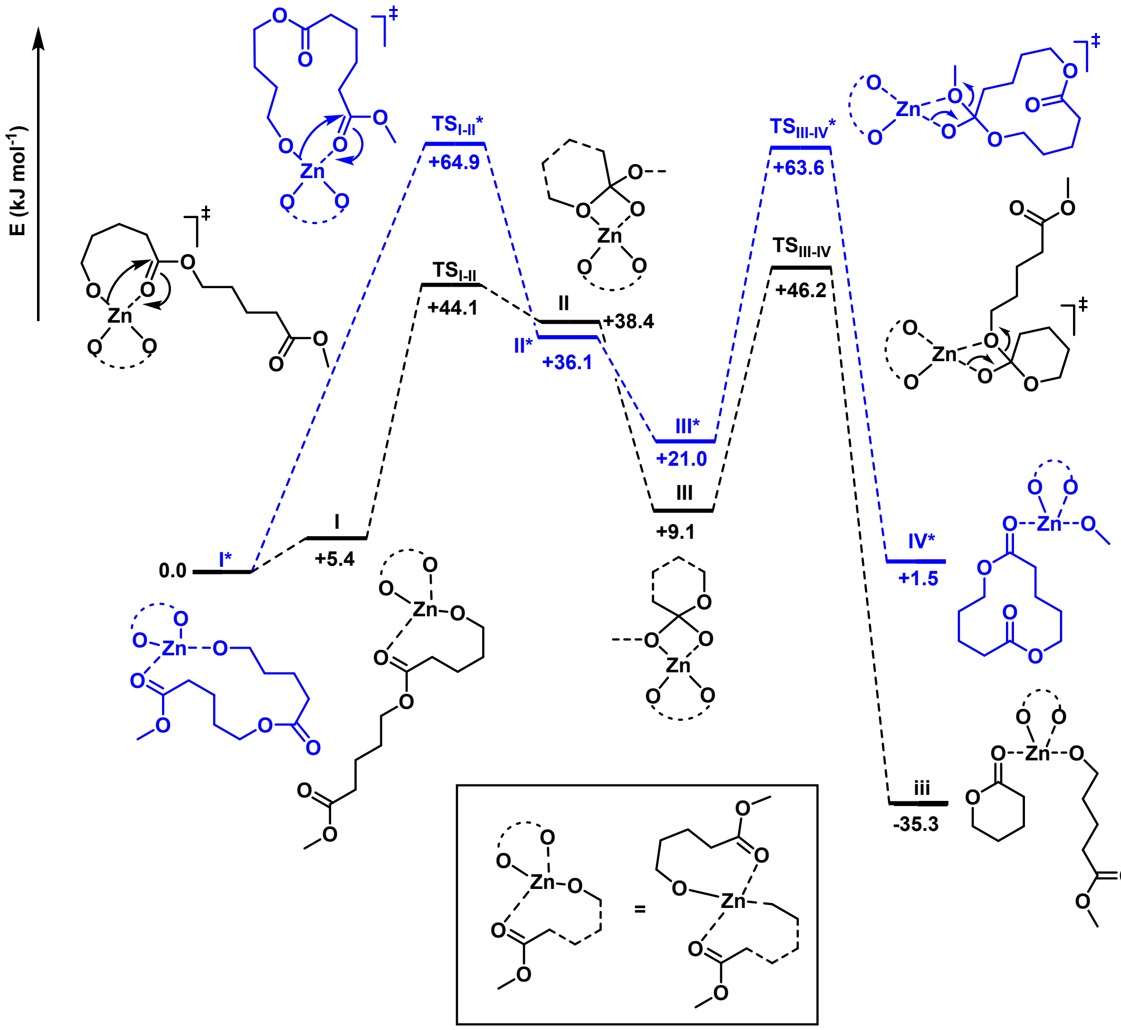

Figure S76. Ring closing of 5-methoxy-5-oxopentyl 5-hydroxypentanoate (to model PE-6a) towards 6a and towards $6a_2$ cyclic dimer. Calculations were performed using the M06L functional (with empirical dispersion correction factor, GD3, applied) basis set 6-31+G(d,p) for O atoms, 6-31G(d,p) for C and H atoms, and basis sets and pseudo potential SDD for Zn atoms. Solvent effects were modelled using the CPCM continuum model for ethylacetate. Goodvibes temperature and concentration factors applied.

Table S23. Computed intermediates and transition states for the backbiting of 5-methoxy-5-oxopentyl 5-hydroxypentanoate (to model PE-6a) towards 6a and towards 6a$_2$ cyclic dimer

Structure	G (Hartree)	G temperature and concentration corrected (Hartree)[a]	ΔG‡ (kJ mol^{-1})
**Ring closure to 6a$_2$ dimer**			
I*	-1494.402504	-1494.430227	0.0
TS$_{I-II}$*	-1494.37789	-1494.405511	64.9
II*	-1494.389154	-1494.416473	36.1
III*	-1494.395177	-1494.422236	21.0
TS$_{III-IV}$*	-1494.379018	-1494.405991	63.6
IV*	-1494.401891	-1494.429652	1.5
**Ring closure to 6a monomer**			
I	-1494.401245	-1494.428177	5.4
TS$_{I-II}$	-1494.387321	-1494.413415	44.1
II	-1494.389784	-1494.4156	38.4
III	-1494.40009	-1494.426777	9.1
TS$_{III-IV}$	-1494.387048	-1494.412629	46.2
IV	-1494.417097	-1494.443686	-35.3

Linear-Free Energy Relationships

Table S24. Data for depolymerization of PE-6a, PE-6b, PE-7a, PE-7b, PC-6b at 1: 1000 $Zn(Oct)_2$:polymer

Entry	Polymer	k[a] (min^{-1})	a[a]	xc[a] (min)	*ka/4 (secs)*	k_{obs} [b] (s^{-1})	k_d [c] (mol^{-1} dm^3 s^{-1})	TOF[d] (h^{-1})
1	PE-6a	0.727	0.929	3.830	0.00281	0.00267 ±0.00029	0.13 ±0.01	7000 ±400
		0.767	0.914	3.469	0.00292			
		0.595	0.913	4.436	0.00226			
2	PE-6b	2.19	0.944	1.62	0.00860	0.00973 ±0.00086	0.56 ±0.05	15900 ±1020
		2.52	0.944	1.47	0.00992			
		2.73	0.939	1.37	0.0107			
3	PE-7a	0.0074	0.852	302.55	0.00002627	0.000028 ±0.000002	0.0016 ±0.00001	100 ±10
		0.00753	0.889	293.59	0.00002788			
		0.00794	0.944	281.08	0.00003122			
4	PE-7b	0.0374	0.518	53.48	0.0000801	0.0000802 ±0.00001	0.0051 ±0.0004	290 ±20
		0.0424	0.500	50.76	0.0000883			
		0.0378	0.457	53.71	0.0000720			
5	PC-6b	0.07075	0.916	36.49	0.00027	0.000270 ±0.0000012	0.018 ±0.0001	700 ±10
		0.06704	0.919	38.21	0.000257			
		0.07517	0.914	31.17	0.000286			

Reaction conducted by solvent casting $Zn(Oct)_2$:polymer solutions ($[Zn(Oct)_2]_0$: $[polymer]_0$ = 1: 1000) ,in TGA crucibles. N_2 flow = 25 mL min^{-1}.[a] Determined from logistic fitting of conversion *vs* time profiles from 0 – 90% mass loss.[b] k_{obs} = average of 3 repeats, error = standard deviation from 3 repeats.[c] k_d = k_{obs}/(2 x $[cat]_0$), assuming 2 active chains per metal centre. [d] TOF = moles of polymer consumed/time * moles of catalyst. Determined from 0 – 30% conversion.

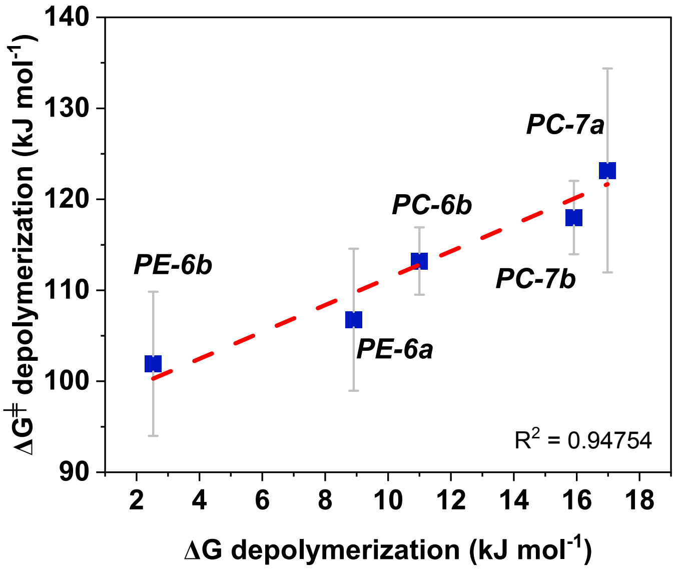

Figure S77. ΔG‡ *vs* ΔG for polymers with primary alkoxide chain ends at 130 °C.

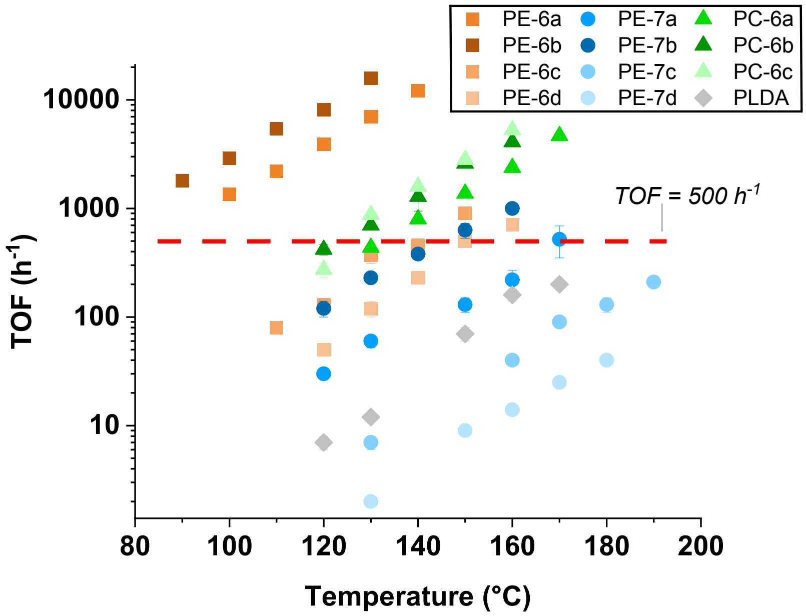

Figure S78. TOF *vs* temperature for all polymers in this study.

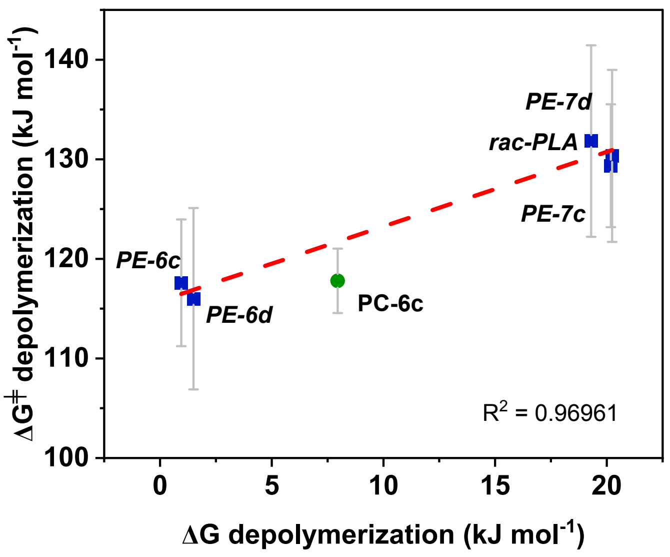

Figure S79. ΔG‡ *vs* ΔG for polymers with secondary alkoxide chain ends. Note. PC-6c is shown on plot (green circle) for reference but is not included in the line of best fit as the polymer has both secondary and primary chain ends.

PE-6b and PE-7c Blend Recycling

Table S25. Calculated rate constants from kinetic parameters of depolymerization, ΔH‡ and ΔS‡

Polymer	k_d*[a] at 90 °C (mol^{-1} dm^3 s^{-1})	k_d* [a] at 120 °C (mol^{-1} dm^3 s^{-1})	k_d*[a] at 190 °C (mol^{-1} dm^3 s^{-1})
PE-6b	0.038 [0.045 ± 0.007]	0.29 [0.26 ± 0.05]	11
PE-7c	0.000010	0.000078	0.0034 [0.0036 ± 0.0005]
PE-6c	0.00048 [0.00036 ± 0.00025]	0.0041	0.21

[a] $k_d^* = \frac{k_b T}{h} \times e^{-(\frac{\Delta H‡}{RT})} \times e^{(\frac{\Delta S‡}{R})}$. Bracketed values indicate measured rate constants at given temperature

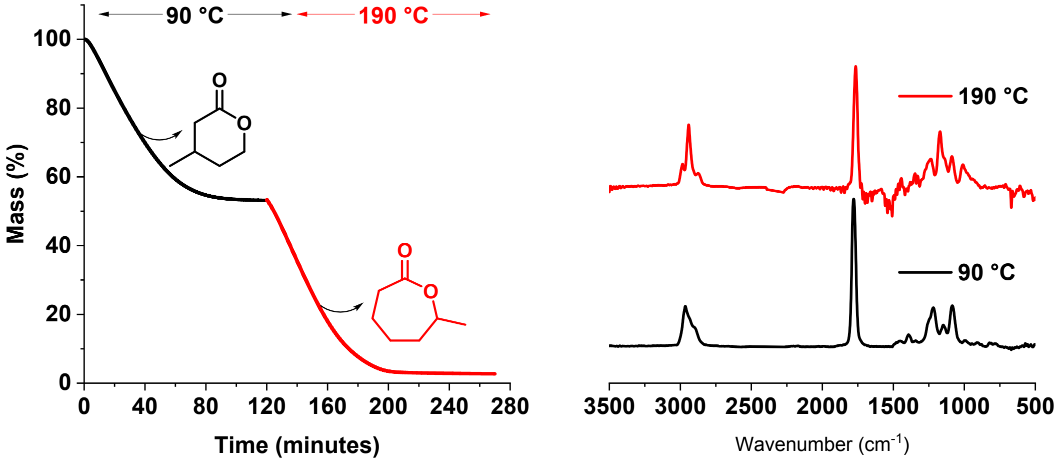

Figure S80. TGA-IR thermograms showing experiments monitoring mass loss in $Zn(Oct)_2$:PE-6b:PE-7c mixtures (molar ratio = 1: 50: 48, PE-6b: PE-7c = 1:1 by mass). The TGAs was run with an isotherm at 90 for 2 h followed by an isotherm at 190 °C 2.5 h

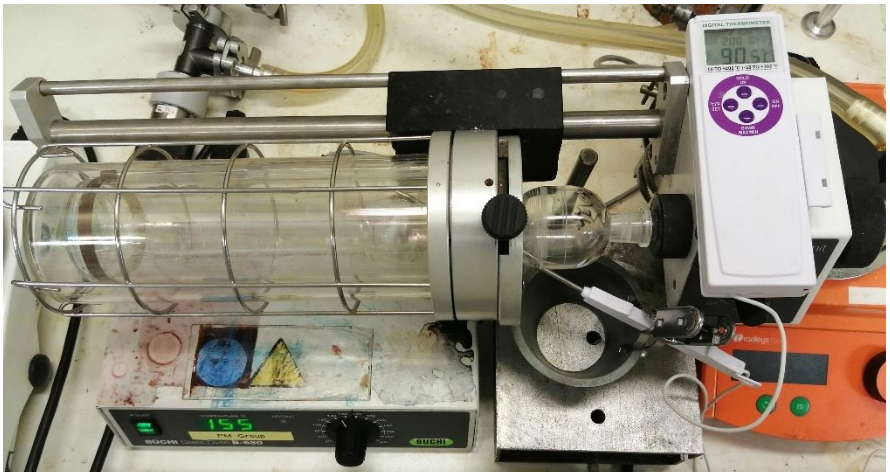

Figure S81. Kugelrohr apparatus used to depolymerize blends of polyester

Table S26. Data for the stepwise recycling of PE-6b:PE-7c blend

Entry	Temp. (°C)	Target mass (g)	Mass recovered (g) [yield]	Major product	6b: 7c[a]
1	90	0.450	0.436 [97%]	6b	>99:0
2	190	0.450	0.438 [97%]	7c	2:98

Reaction conducted by solvent casting $Zn(Oct)_2$: PE-6b: PE-7c solutions ($[Zn(Oct)_2]_0$: $[PE\text{-}6b]_0$: $[PE\text{-}7c]_0$ = 1: 50: 48) in round bottom flask to form thin films before separation using Kugelrohr apparatus. Isotherms at each temperature were held for 1-2 h. . [a] Target mass at 90 °C = initial mass of PE-6b, target mass at 190 °C = initial mass of PE-7c.[b] Yield = mass recovered/target mass for given isotherm.[c] Determined by GC-MS.

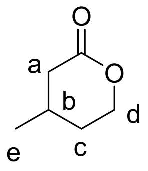

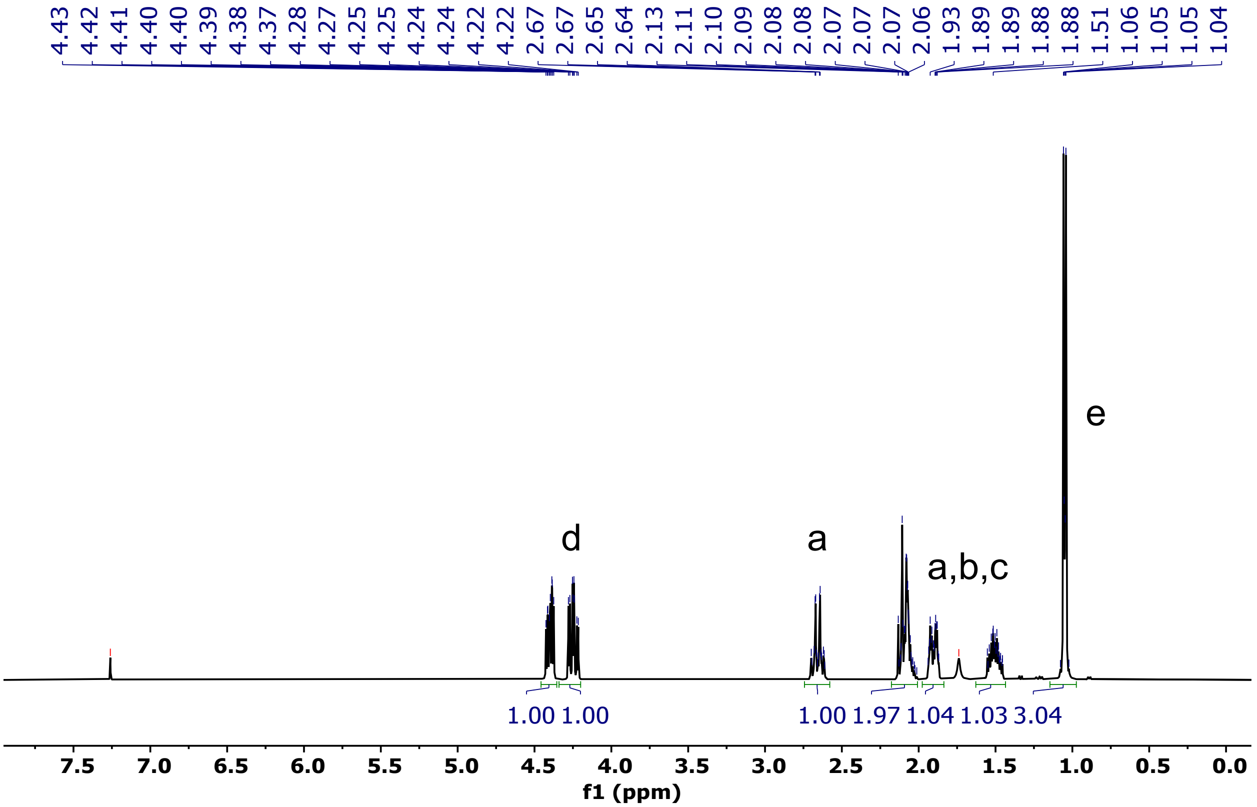

Figure S82. ^{1}H NMR spectrum (400 MHz, $CDCl_3$, 298K) of distillate isolated from recycling of PE-6b: PE-7c blend at 90 °C ($[Zn(Oct)_2]_0$:$[PE\text{-}6b]_0$:$[PE\text{-}7c]_0$ 1:50:48, 2 h, 15 – 20 mbar)

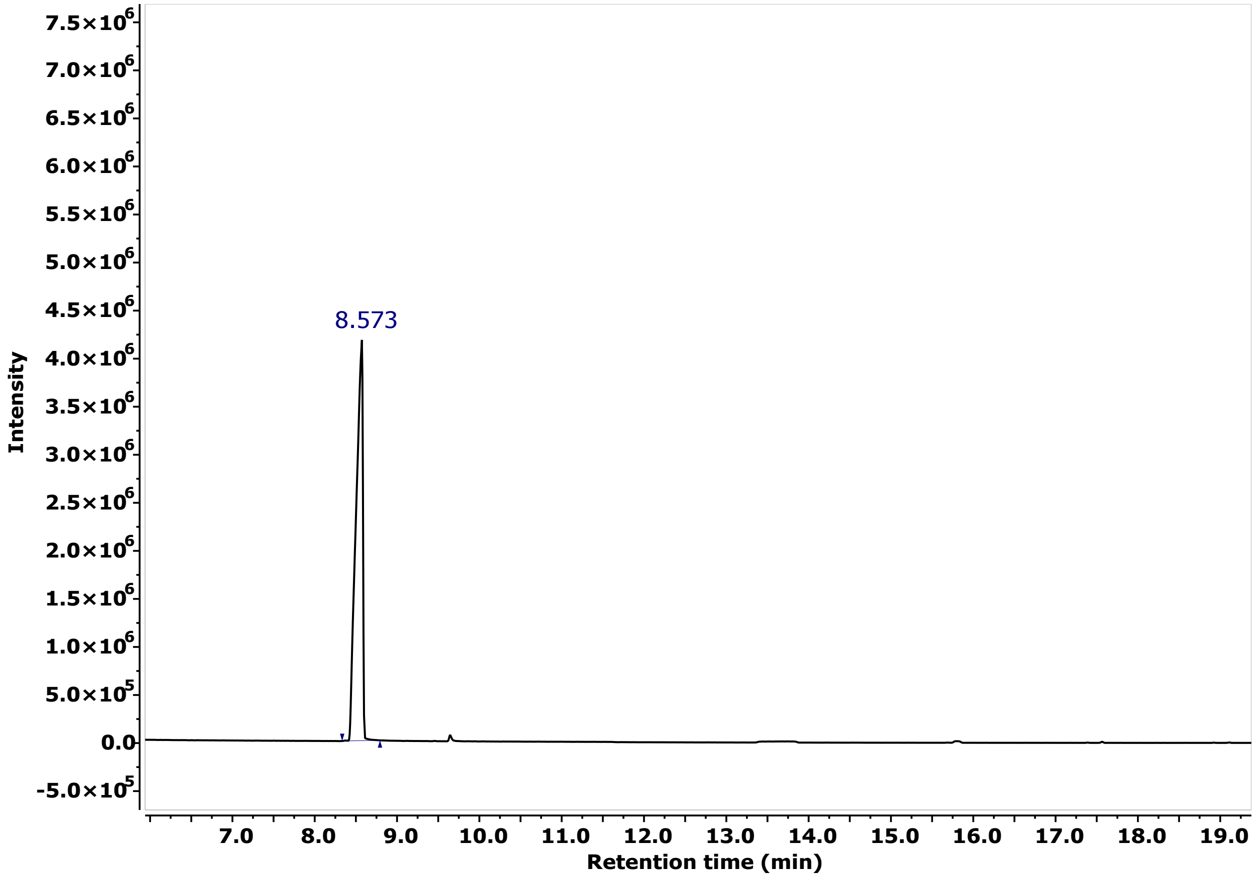

Figure S83. GC of distillate isolated from recycling of PE-6b: PE-7c blend at 90 °C ([Zn(Oct)$_2$]$_0$:[PE-6b]$_0$:[PE-7c]$_0$ 1:50:50, 2 h, 15 – 20 mbar)

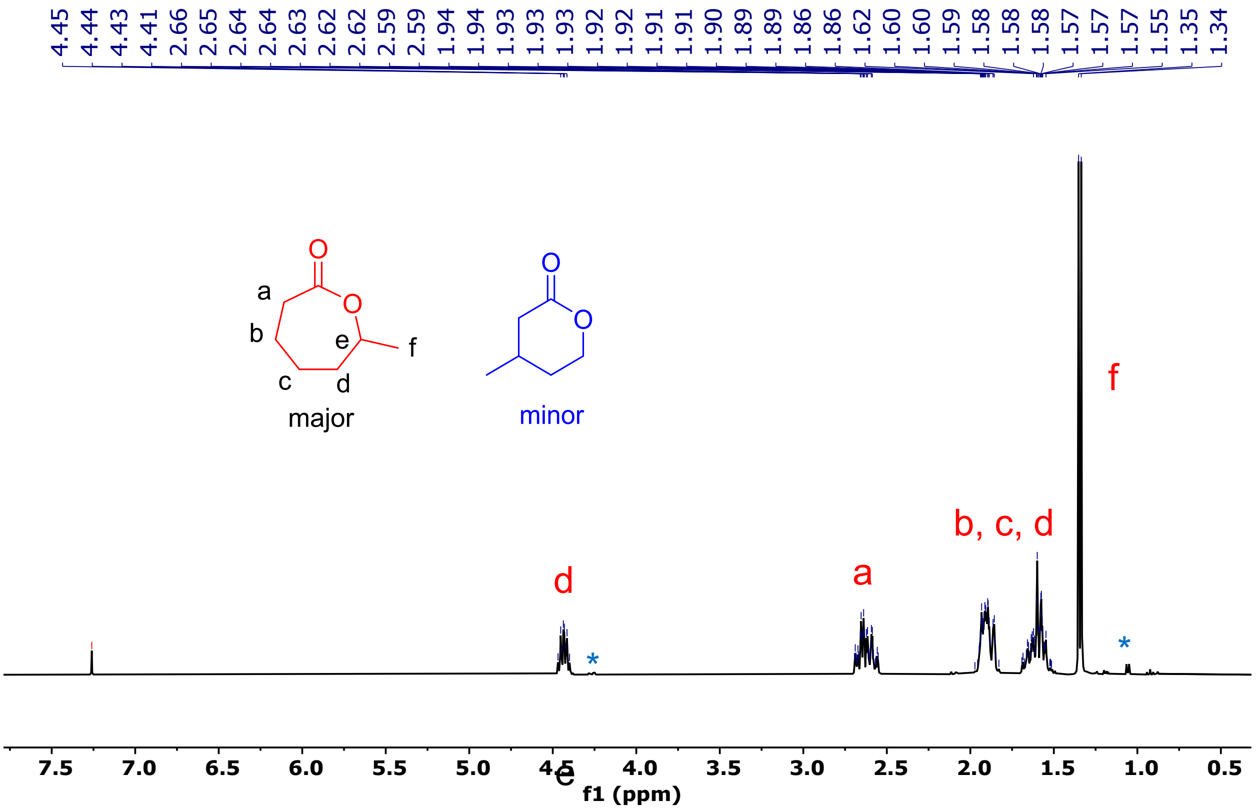

Figure S84. **^{1}H NMR spectrum (400 MHz, $CDCl_3$, 298K) of distillate isolated from recycling of PE-6b: PE-7c blend at 190 °C ($[Zn(Oct)_2]_0$:$[PE\text{-}6b]_0$:$[PE\text{-}7c]_0$ 1:50:50, 2 h, 1 – 8 mbar). * shows trace amounts of 6b**

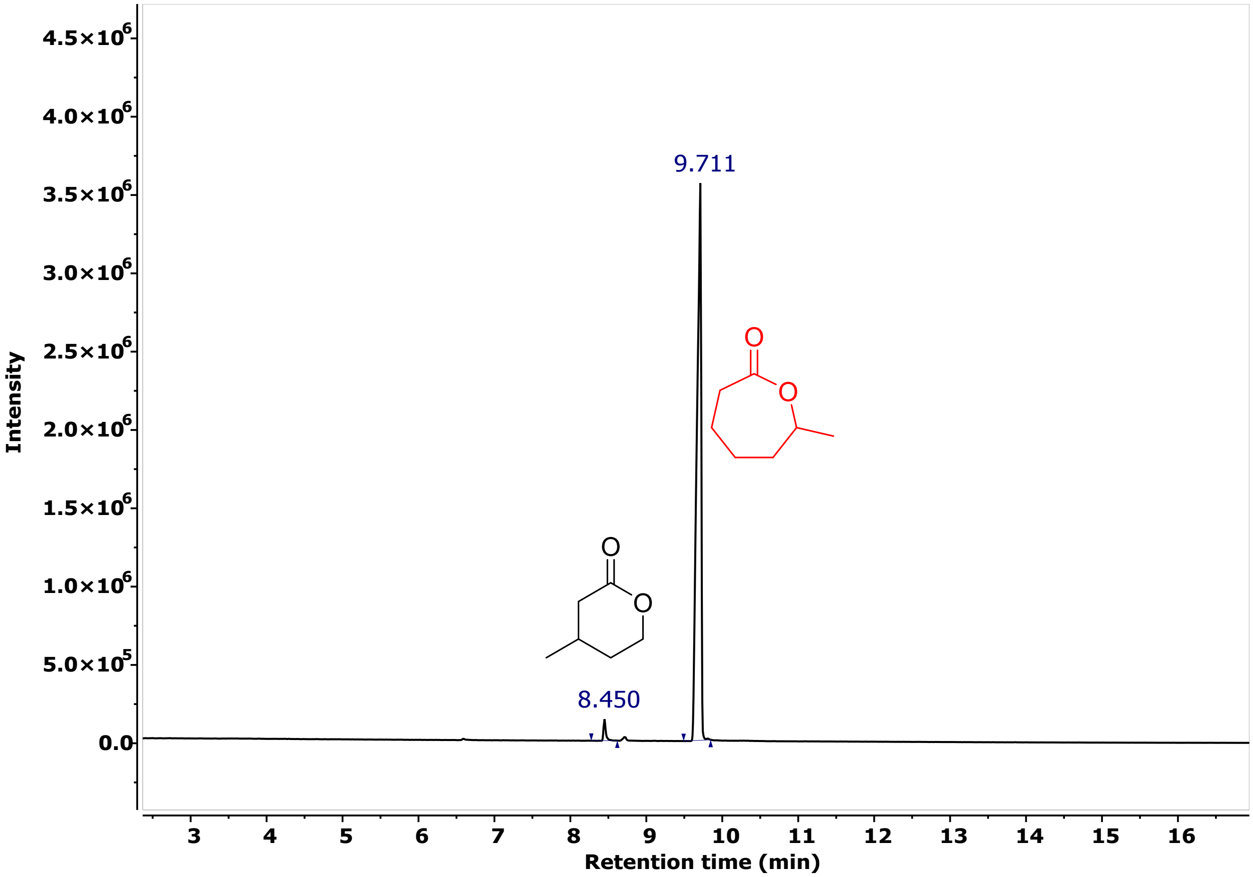

Figure S85. GC of distillate isolated from recycling of PE-6b: PE-7c blend at 190 °C ([Zn(Oct)$_2$]$_0$:[PE-6b]$_0$:[PE-7c]$_0$ 1:50:50, 1 – 8 mbar, 2 h)

PE-6b, PE-6c and PE-7c blend recycling

Table S27. Data for the stepwise recycling of PE-6b:PE-6c:PE-7c blend

Entry	Temp. (°C)	Target mass[a] (g)	Mass recovered (g) [yield][b]	Major Product[c]	6b: 6c: 7c[c]
1	90	0.450	0.463 [102%]	6b	91: 9: 0
2	120	0.404	0.494 [110%]	6c	24: 69: 7
3	190	0.450	0.330 [73%]	7c	2: 9: 89

Reaction conducted by solvent casting $Zn(Oct)_2$: PE-6b: PE-7c solutions ($[Zn(Oct)_2]_0$: $[PE\text{-}6b]_0$: $[PE\text{-}6c]_0$: $[PE\text{-}7c]_0$ = 1: 35: 33: 33) in round bottom flask to form thin films before separation using Kugelrohr apparatus. Isotherms at each temperature were held for 1-2 h. [a] Target mass at 90 °C = initial mass of PE-6b, target mass at 120 °C = initial mass of PE-6c, target mass at 190 °C = initial mass of PE-7c.[b] Yield = Mass recovered/target mass for given isotherm.[c] Determined by GC-MS.

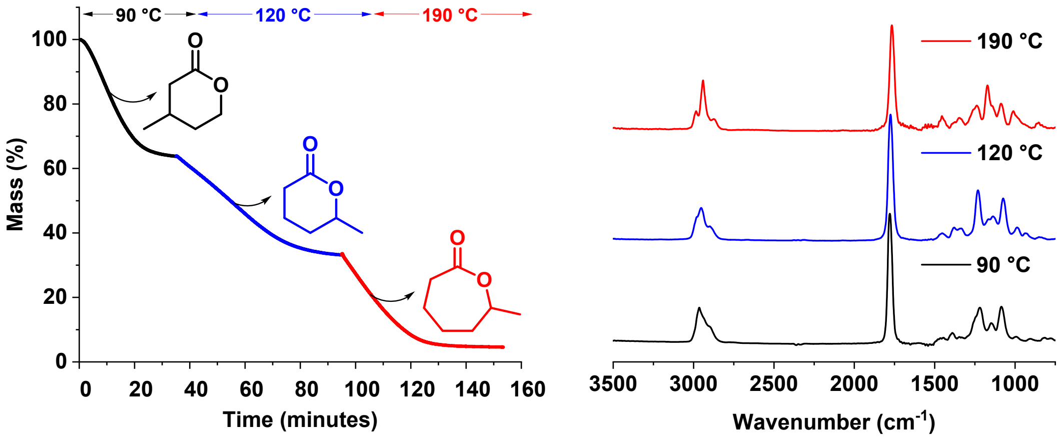

Figure S86. TGA thermograms showing experiments monitoring mass loss in $Zn(Oct)_2$:PE-6b:PE-7c mixtures (molar ratio = 1: 35: 33: 33, PE-6b: PE-7c = 1:1 by mass). The TGAs was run with an isotherm at 90 for 2 h followed by an isotherm at 190 °C 2.5 h

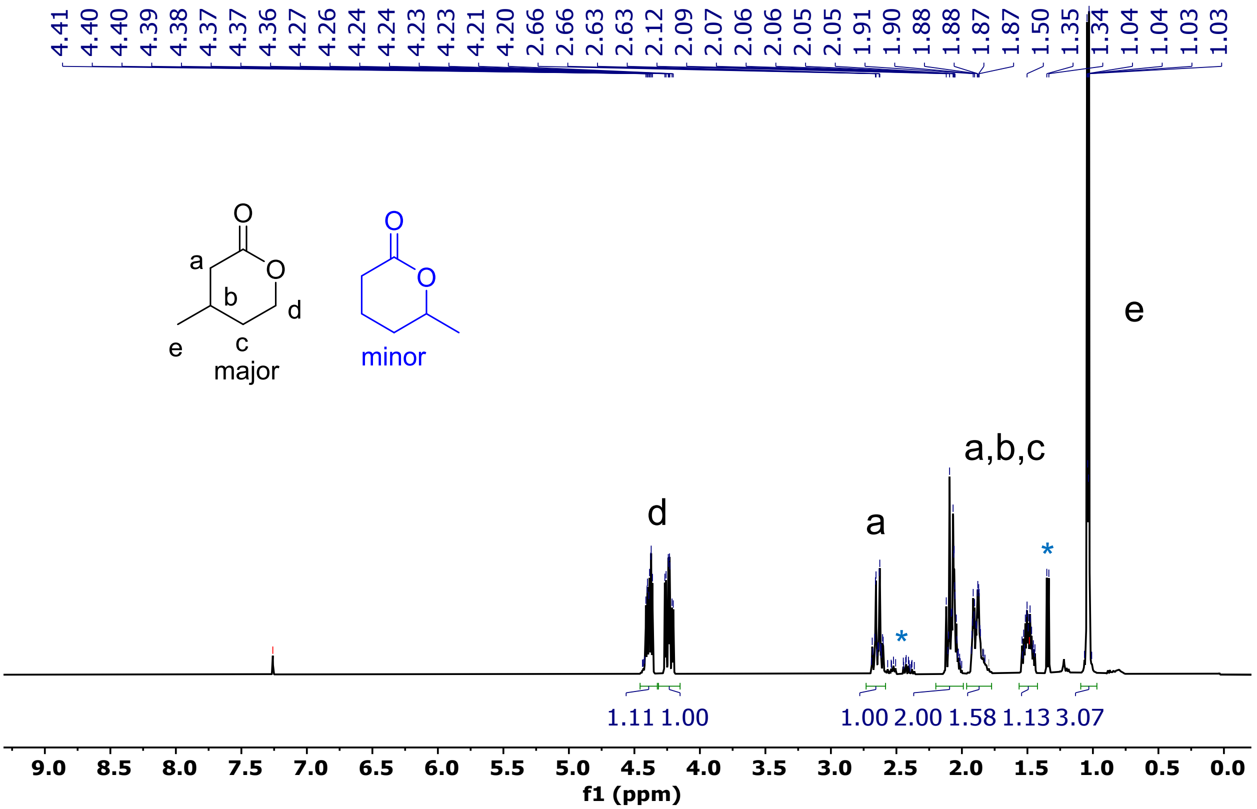

Figure S87. **^{1}H NMR spectrum (400 MHz, $CDCl_3$, 298K) of distillate isolated from recycling of PE-6b: PE-6c: PE-7c blend at 90 °C ($[Zn(Oct)_2]_0$:$[PE\text{-}6b]_0$:$[PE\text{-}6c]_0$:$[PE\text{-}7c]_0$ 1:33:33:33, 2 h, 15 – 20 mbar). Trace 6c highlighted with** *

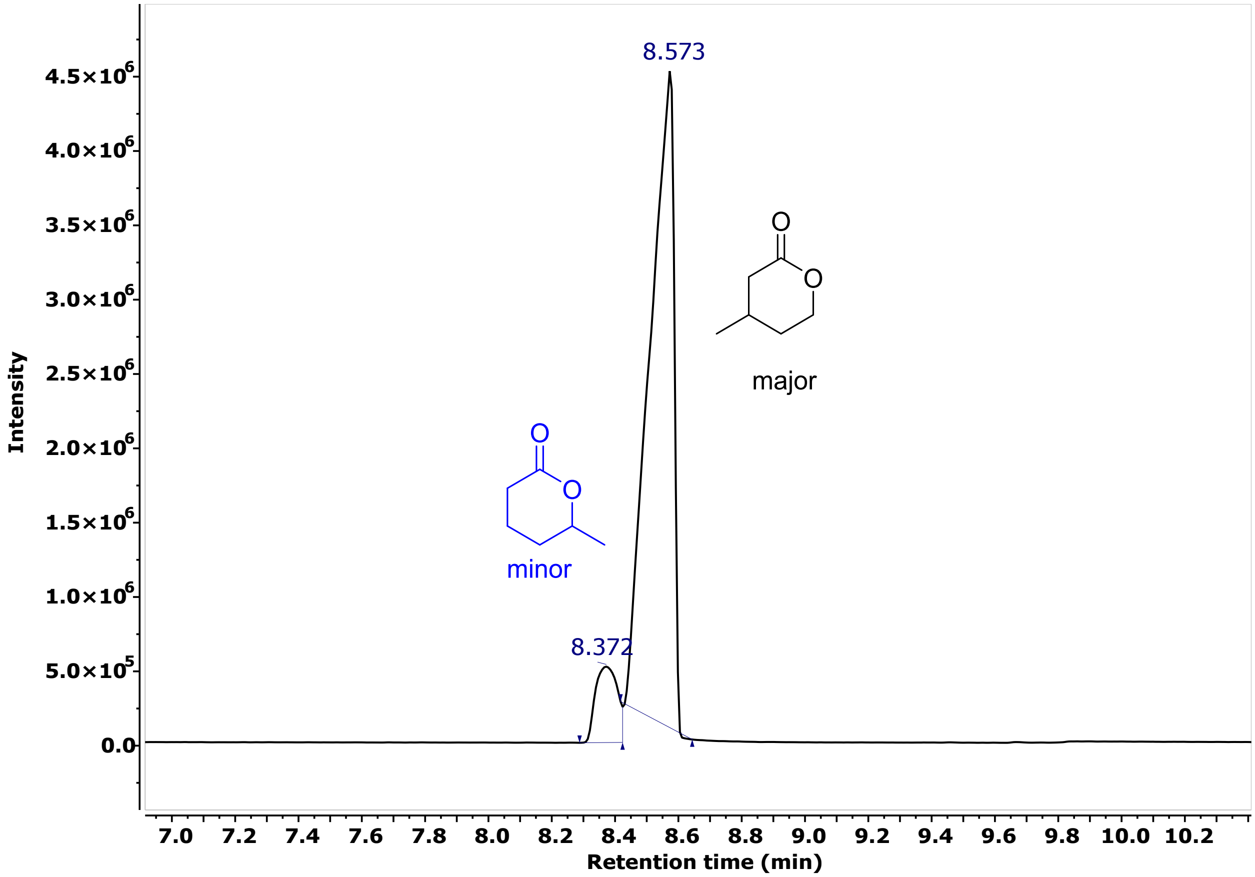

Figure S88. GC of distillate isolated from recycling of PE-6b: PE-6c: PE-7b blend at 90 °C ([Zn(Oct)$_2$]$_0$:[PE-6b]$_0$:[PE-6c]$_0$:[PE-7c]$_0$ 1:33:33:33, 2 h, 15 – 20 mbar)

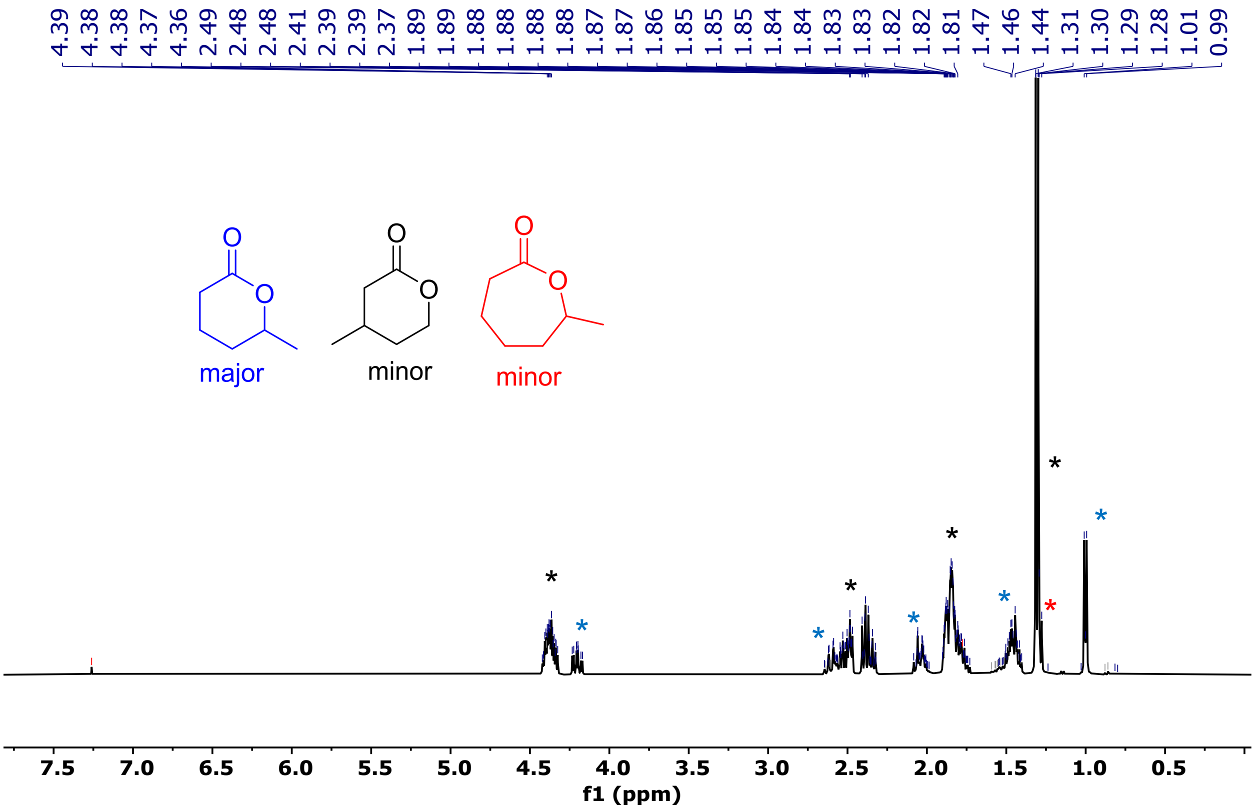

Figure S89. ^{1}H **NMR (400 MHz,** $CDCl_3$**, 298K) spectrum of distillate isolated from recycling of PE-6b: PE-6c: PE-7c blend at 130 °C (**$[Zn(Oct)_2]_0:[PE\text{-}6b]_0:[PE\text{-}6c]_0:[PE\text{-}7c]_0$ **1:33:33:33, 2 h, 15 – 20 mbar). Trace trace 6b highlighted with *; trace 7c highlighted with ***

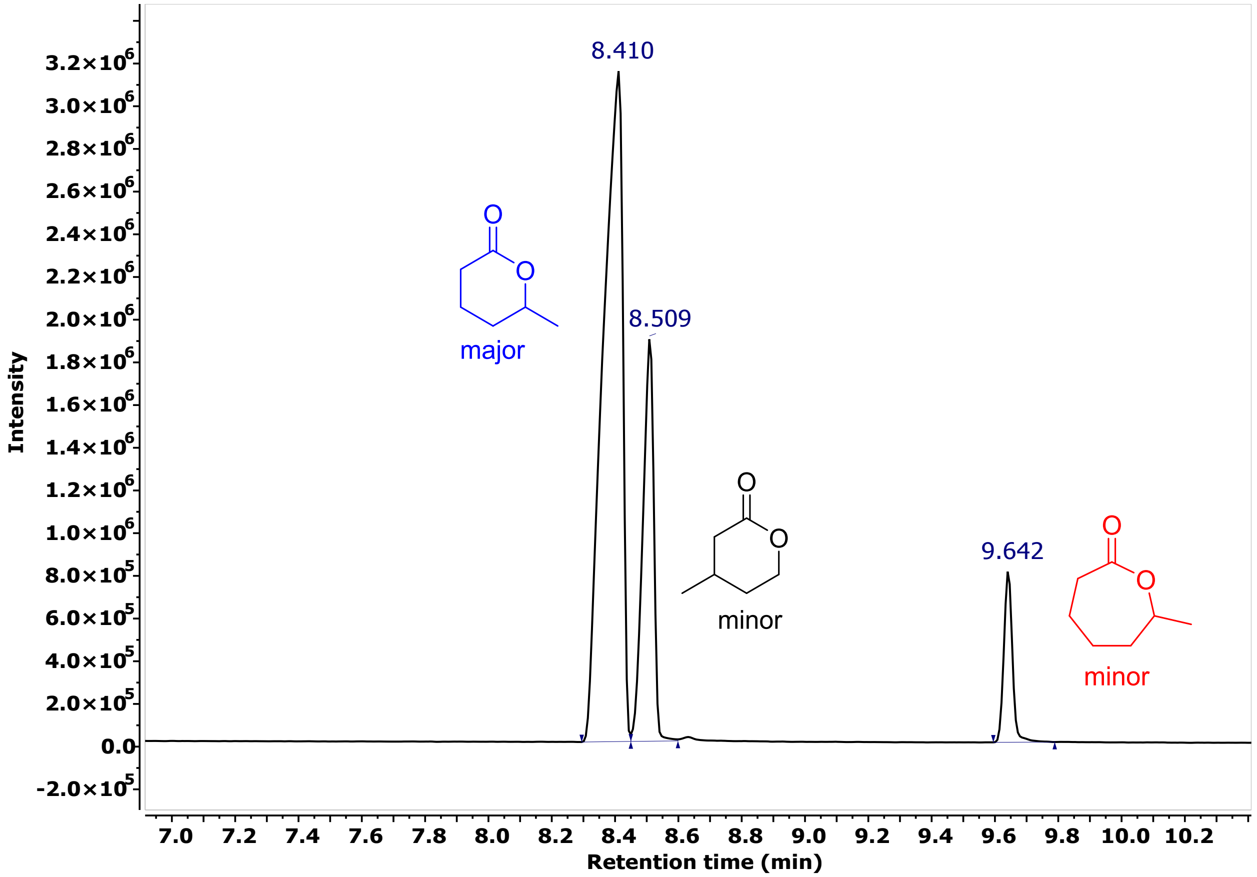

Figure S90. GC of distillate isolated from recycling of PE-6b: PE-6c: PE-7c blend at 130 °C ($[Zn(Oct)_2]_0$:$[PE\text{-}6b]_0$:$[PE\text{-}6c]_0$:$[PE\text{-}7c]_0$ 1:33:33:33, 15 – 20 mbar, 130 °C)

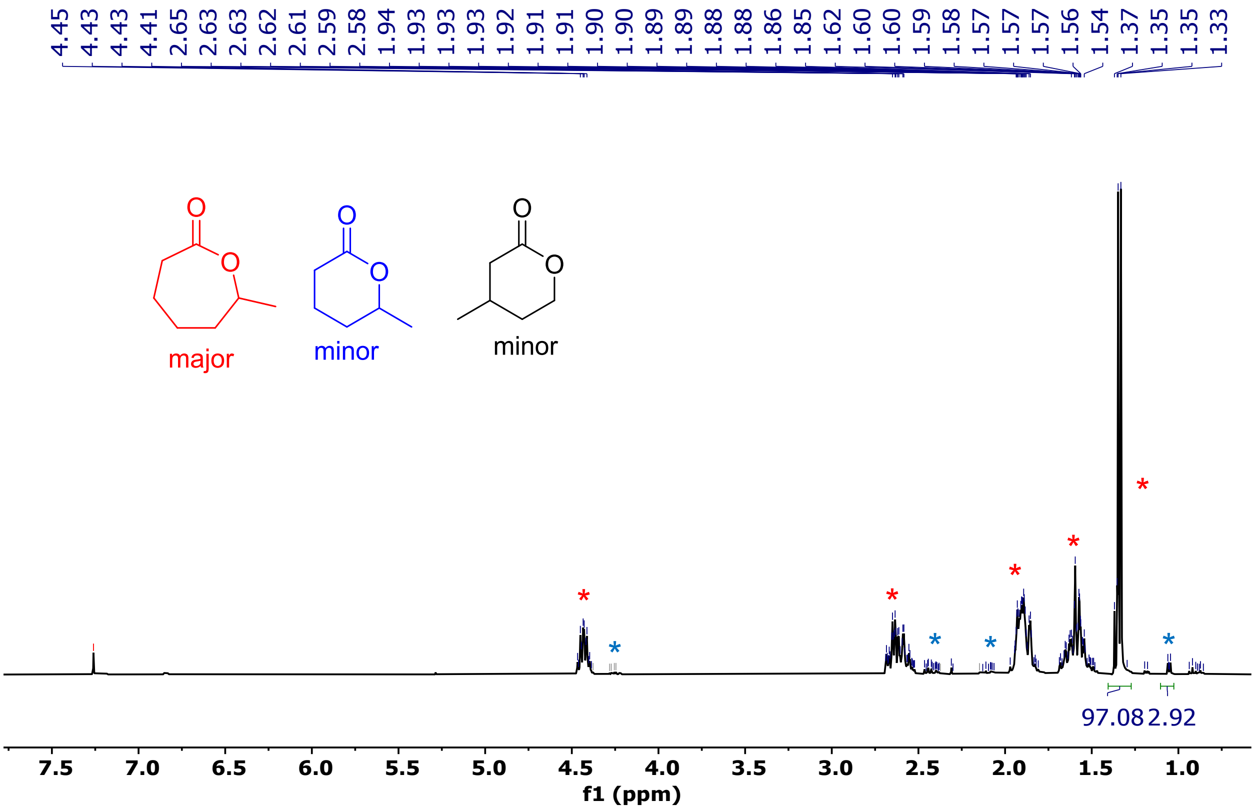

Figure S91. **^{1}H NMR (400 MHz, $CDCl_3$, 298K) spectrum of distillate isolated from recycling of PE-6b: PE-6c: PE-7c blend at 190 °C ($[Zn(Oct)_2]_0$:$[PE\text{-}6b]_0$:$[PE\text{-}6c]_0$:$[PE\text{-}7c]_0$ 1:33:33:33, 2 h, 1 – 8 mbar). Trace 6c highlighted with *; trace 6b highlighted with ***

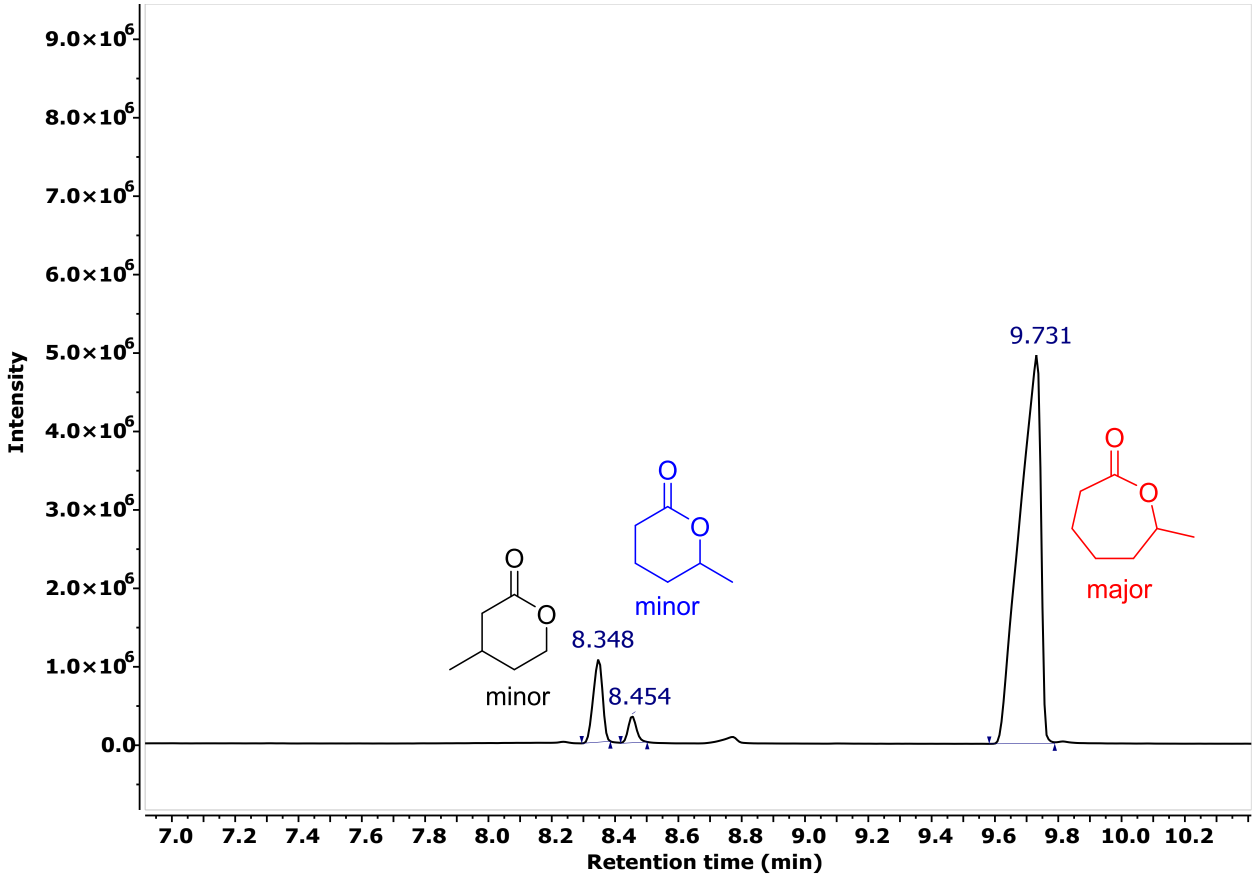

Figure S92. GC of distillate isolated from recycling of PE-6b: PE-6c: PE-7c blend at 190 °C ([Zn(Oct)$_2$]$_0$:[PE-6b]$_0$:[PE-6c]$_0$:[PE-7c]$_0$ 1:33:33:33, 1 – 8 mbar, 2 h)

5. Polymer characterisation

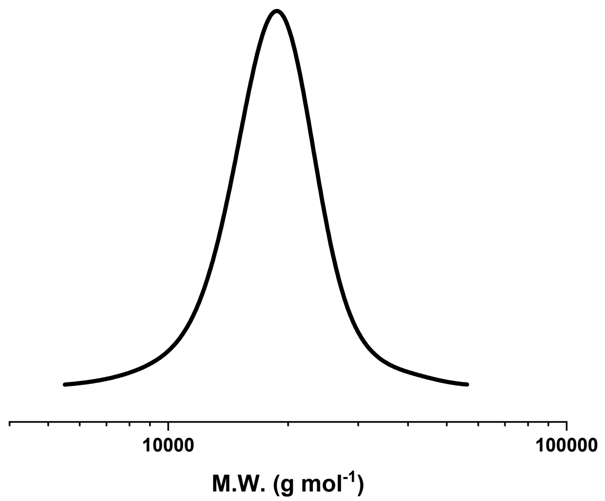

Figure S93. SEC trace of PE-6a. $M_{n,SEC}$ = 17500 g mol $^{-1}$, $Đ_M$ = 1.09 in THF against polystyrene standards

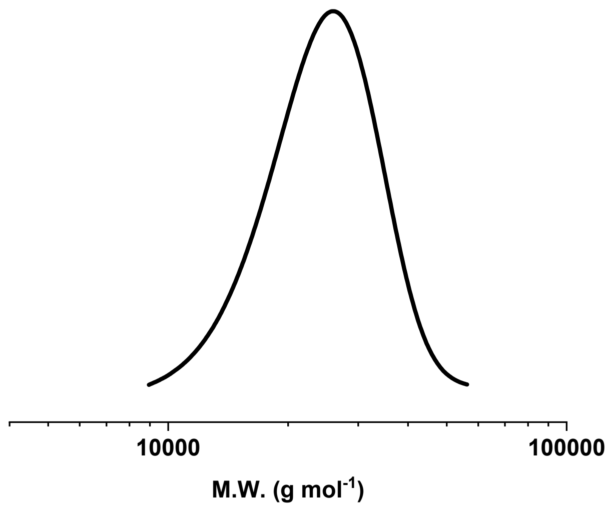

Figure S94. SEC trace of PE-6b. $M_{n,SEC}$ = 23100 g mol $^{-1}$, $Đ_M$ = 1.10 in THF against polystyrene standards

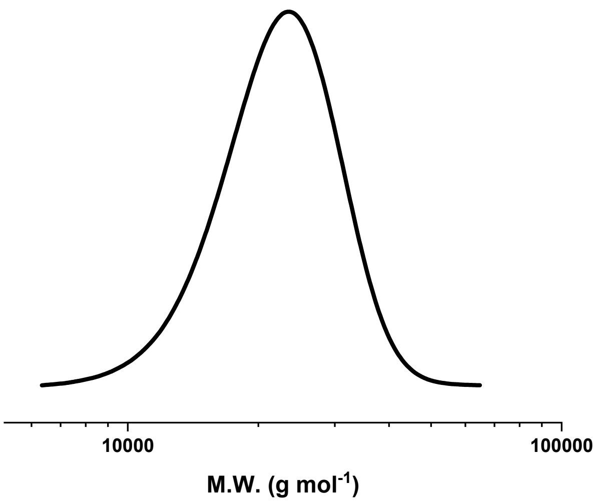

Figure S95. SEC trace of PE-6c. $M_{n,SEC}$ = 21000 g mol $^{-1}$, $Đ_M$ = 1.10 in THF against polystyrene standards

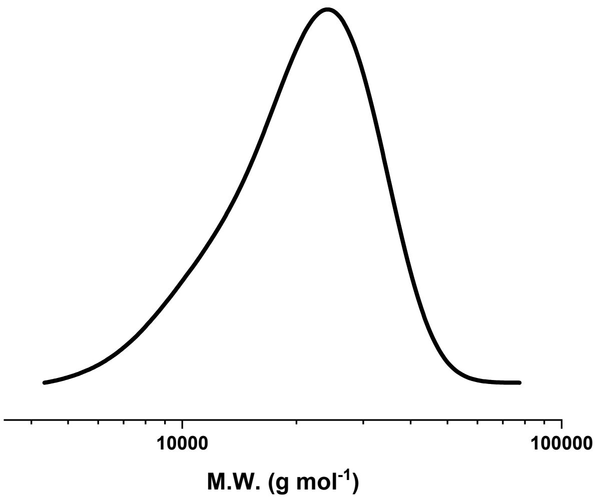

Figure S96. SEC trace of PE-6d. $M_{n,SEC}$ = 18300 g mol $^{-1}$, $Đ_M$ = 1.22 in THF against polystyrene standards

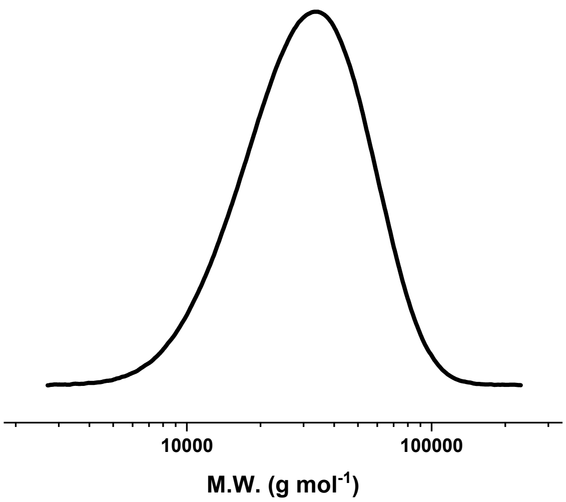

Figure S97. SEC trace of PE-7a. $M_{n,SEC}$ = 24900 g mol $^{-1}$, $Đ_M$ = 1.39 in THF against polystyrene standards

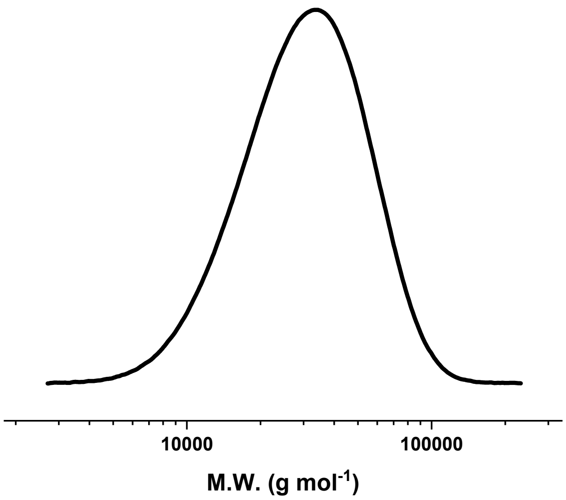

Figure S98. SEC trace of PE-7b. $M_{n,SEC}$ = 22800 g mol $^{-1}$, $Đ_M$ = 1.39 in THF against polystyrene standards

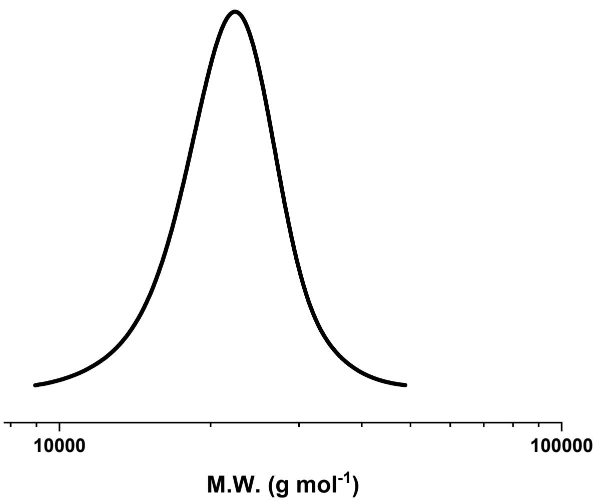

Figure S99. SEC trace of PE-7c. $M_{n,SEC}$ = 21100 g mol $^{-1}$, $Đ_M$ = 1.06 in THF against polystyrene standards

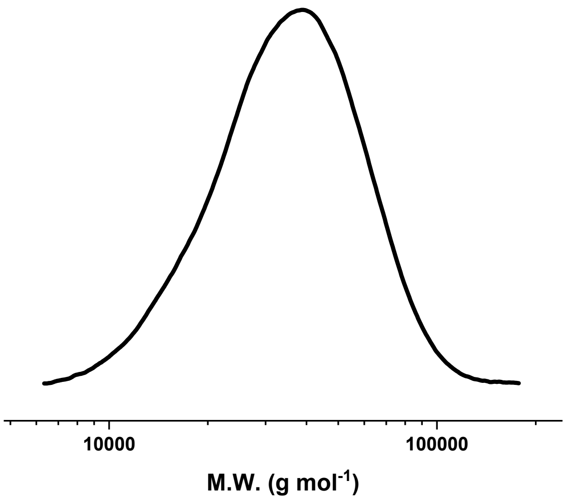

Figure S100. SEC trace of PE-7d. $M_{n,SEC}$ = 30400 g mol $^{-1}$, $Đ_M$ = 1.29 in THF against polystyrene standards

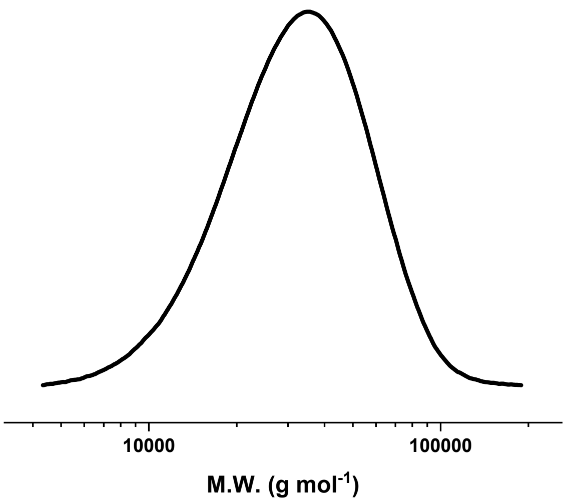

Figure S101. SEC trace of *rac*-PLA. $M_{n,SEC}$ = 27000 g mol $^{-1}$, $Đ_M$ = 1.35 in THF against polystyrene standards

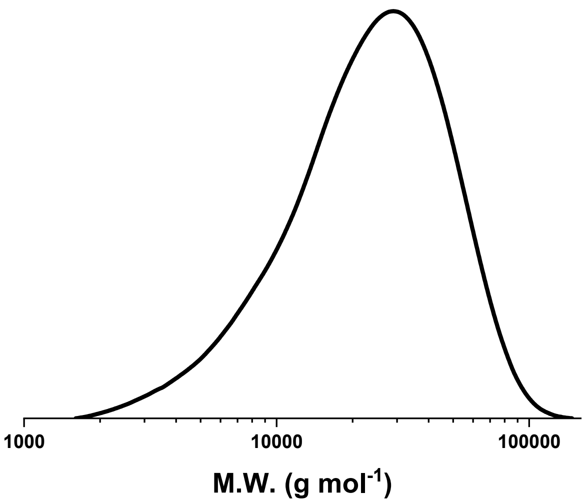

Figure S102. SEC trace of PC-6a. $M_{n,SEC}$ = 16700 g mol $^{-1}$, $Đ_M$ = 1.70 in THF against polystyrene standards

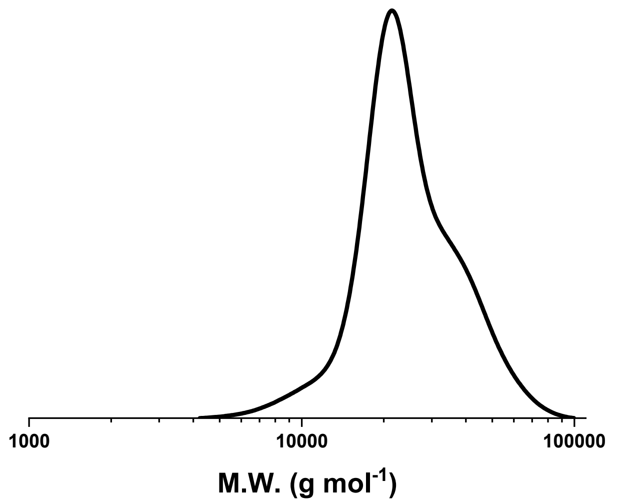

Figure S103. SEC trace of PC-6b. $M_{n,SEC}$ **= 22200 g mol**$^{-1}$**,** $Đ_M$ **= 1.20**

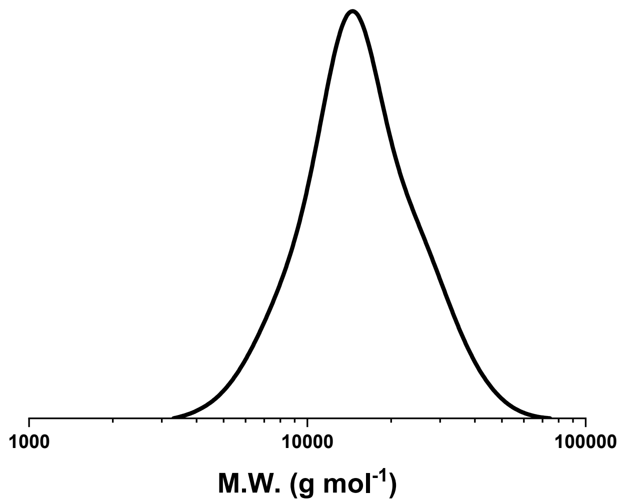

Figure S104. SEC trace of PC-6c. $M_{n,SEC}$ **= 13600 g mol**$^{-1}$**,** $Đ_M$ **= 1.26**

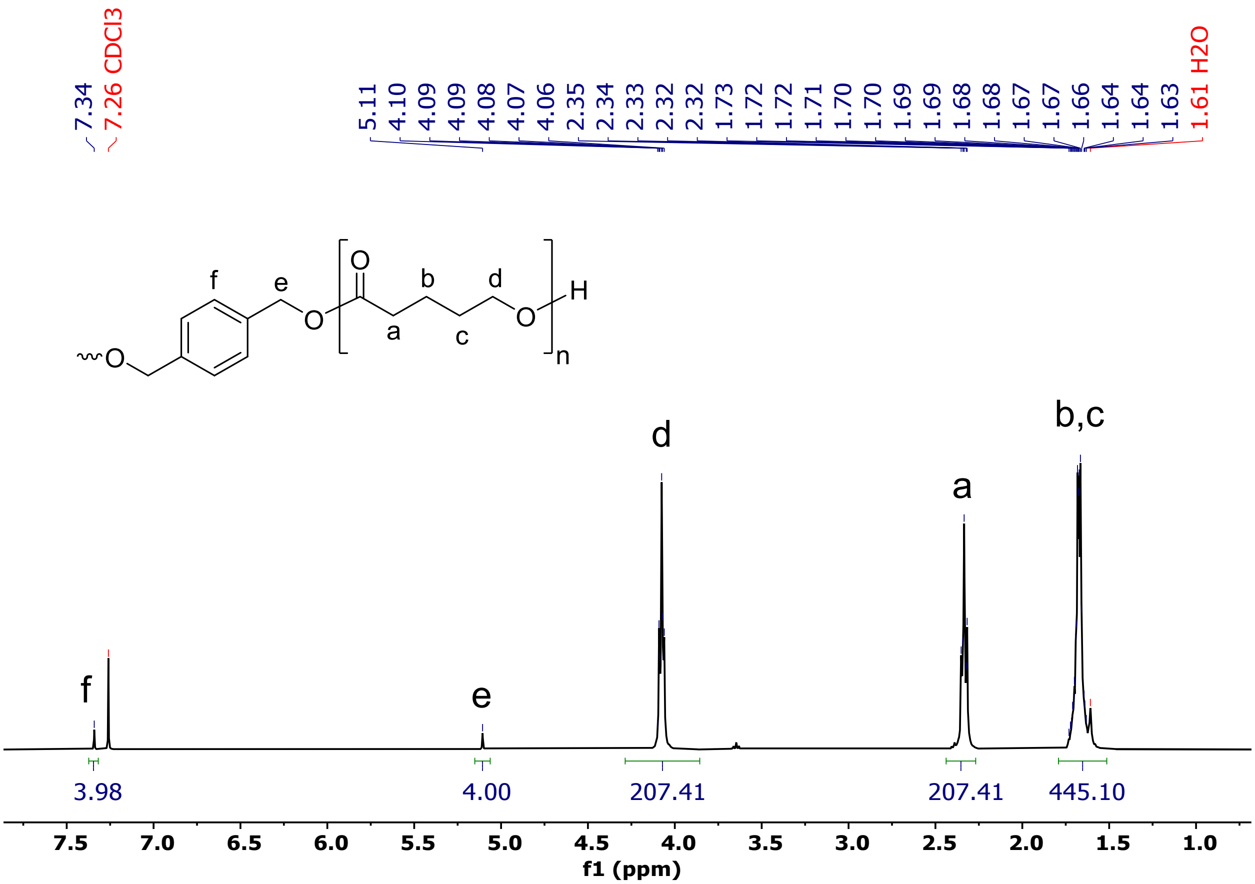

Figure S105. ^{1}H NMR spectrum (400 MHz, $CDCl_3$, 298K) of PE-6a. DP_{NMR} =104 determined from relative integration of H_d and H_e.

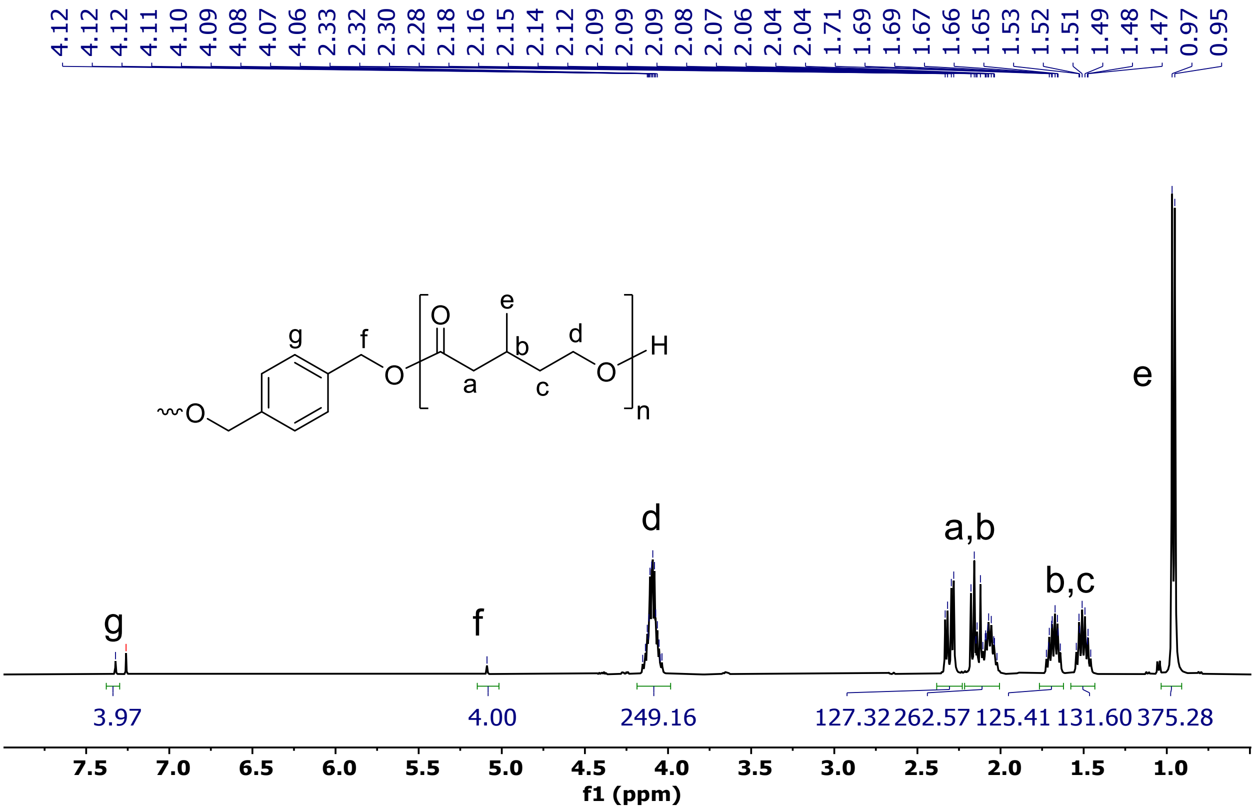

Figure S106. **^{1}H NMR spectrum (400 MHz, $CDCl_3$, 298K) of PE-6b. DP_{NMR} =125 determined from relative integration of H_d and H_f.**

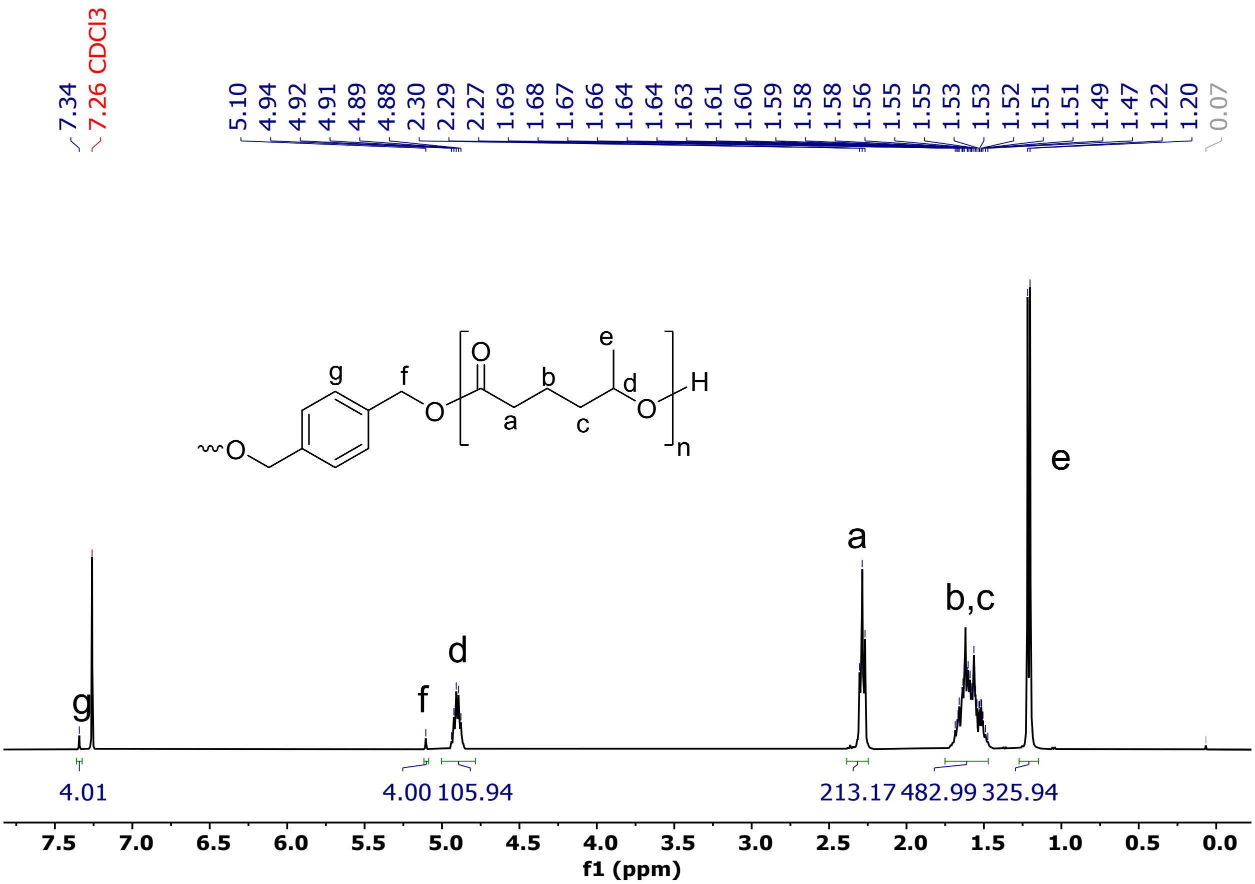

Figure S107. **^{1}H NMR spectrum (400 MHz, $CDCl_3$, 298K) of PE-6c. DP_{NMR} =106 determined from relative integration of H_d and H_f**

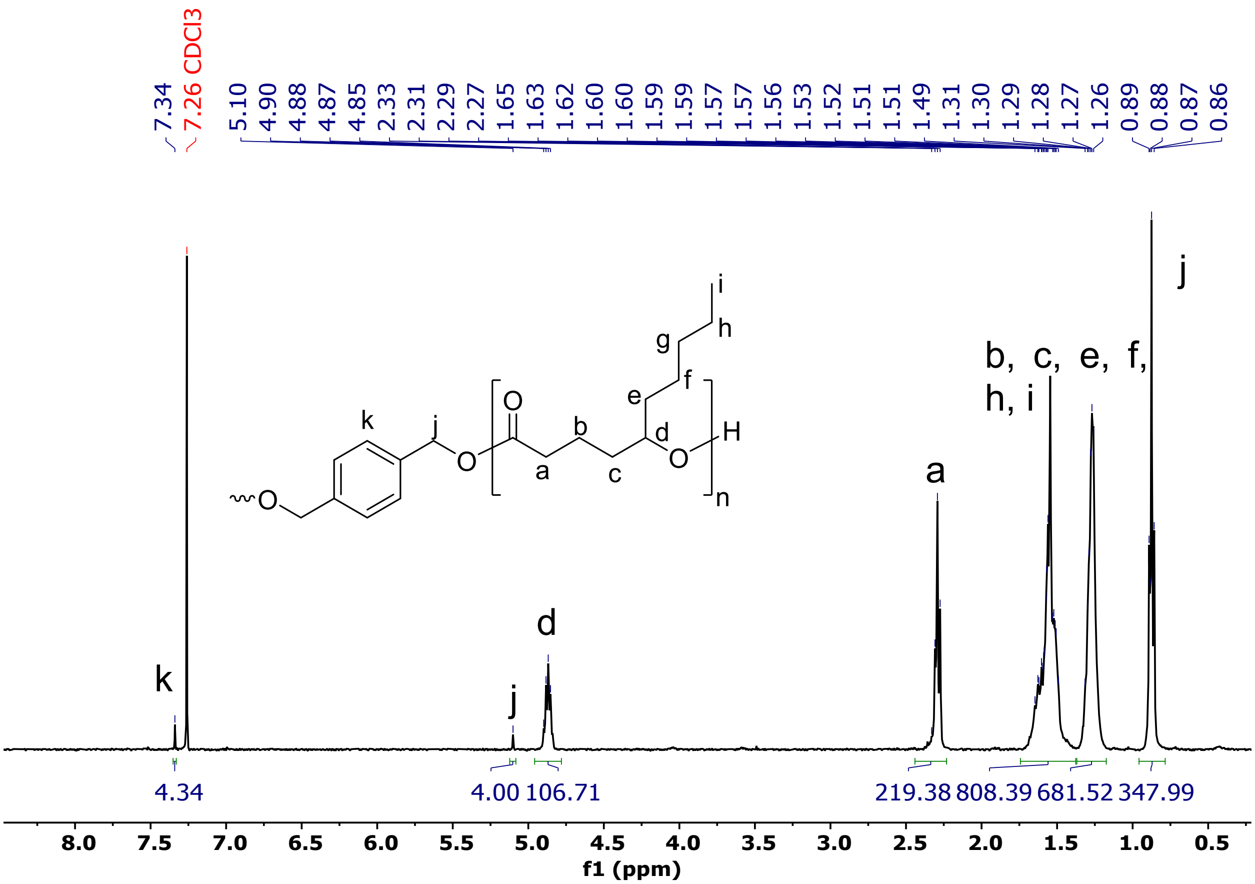

Figure S108. ^{1}H NMR spectrum (400 MHz, $CDCl_3$, 298K) of PE-6d. DP_{NMR} =107 determined from relative integration of H_j and H_d.

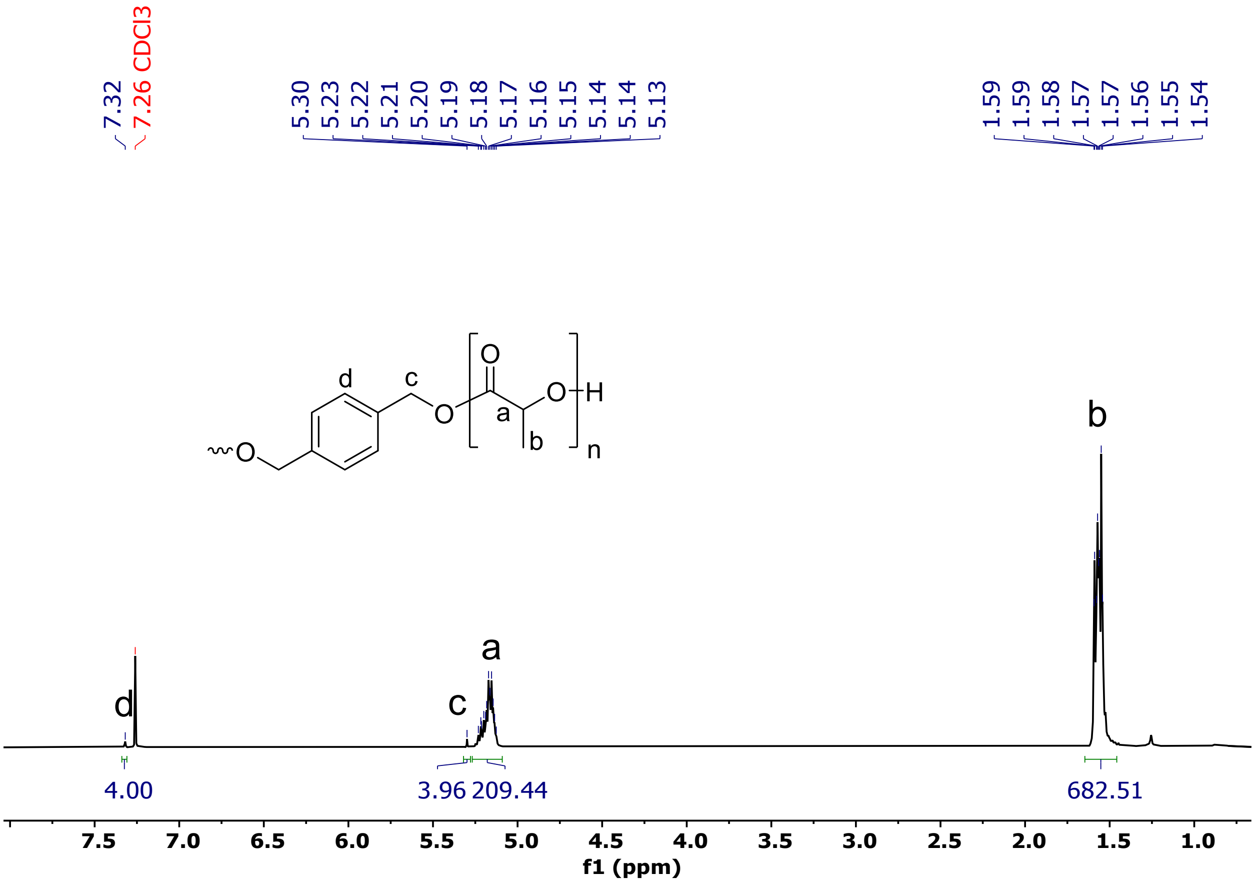

Figure S109. ^1H **NMR spectrum (400 MHz, $CDCl_3$, 298K) of *rac*-PLA.** DP_{NMR} **=105 determined from relative integration of** H_d **and** H_a**.**

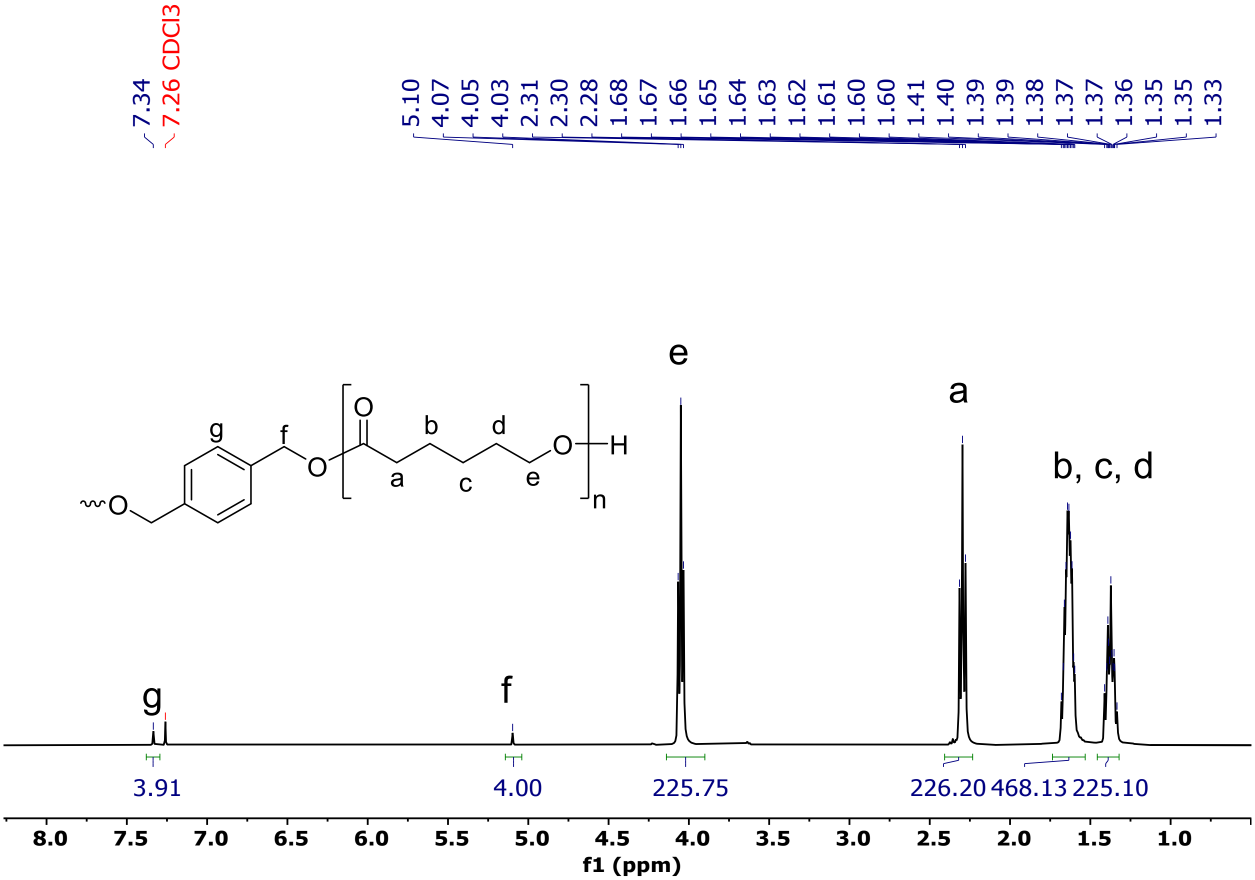

Figure S110. **^{1}H NMR spectrum (400 MHz, $CDCl_3$, 298K) of PE-7a. DP_{NMR} =112 determined from relative integration of H_f and H_e**

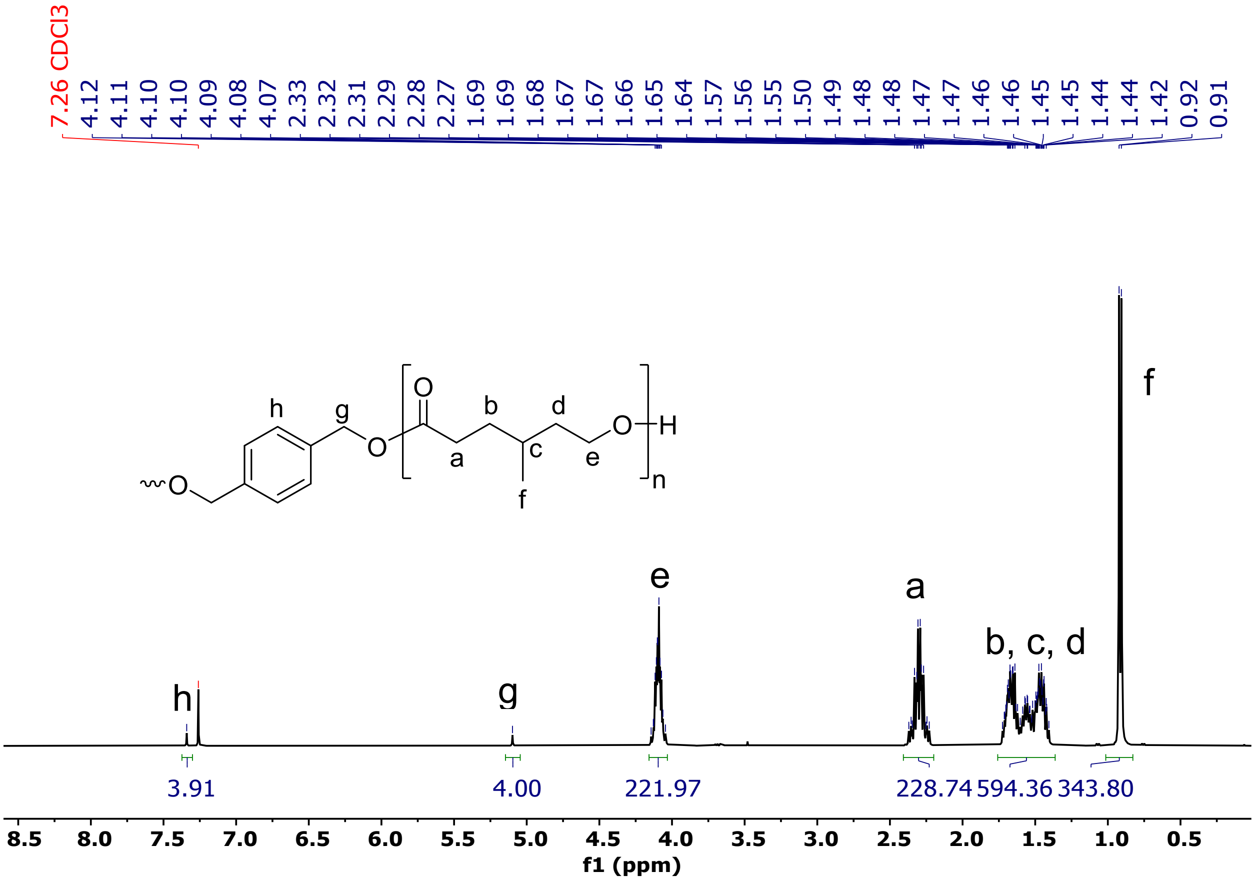

fFigure S111. **^{1}H NMR spectrum (400 MHz, $CDCl_3$, 298K) of PE-7b.** ***DP_{NMR}*** **=111**

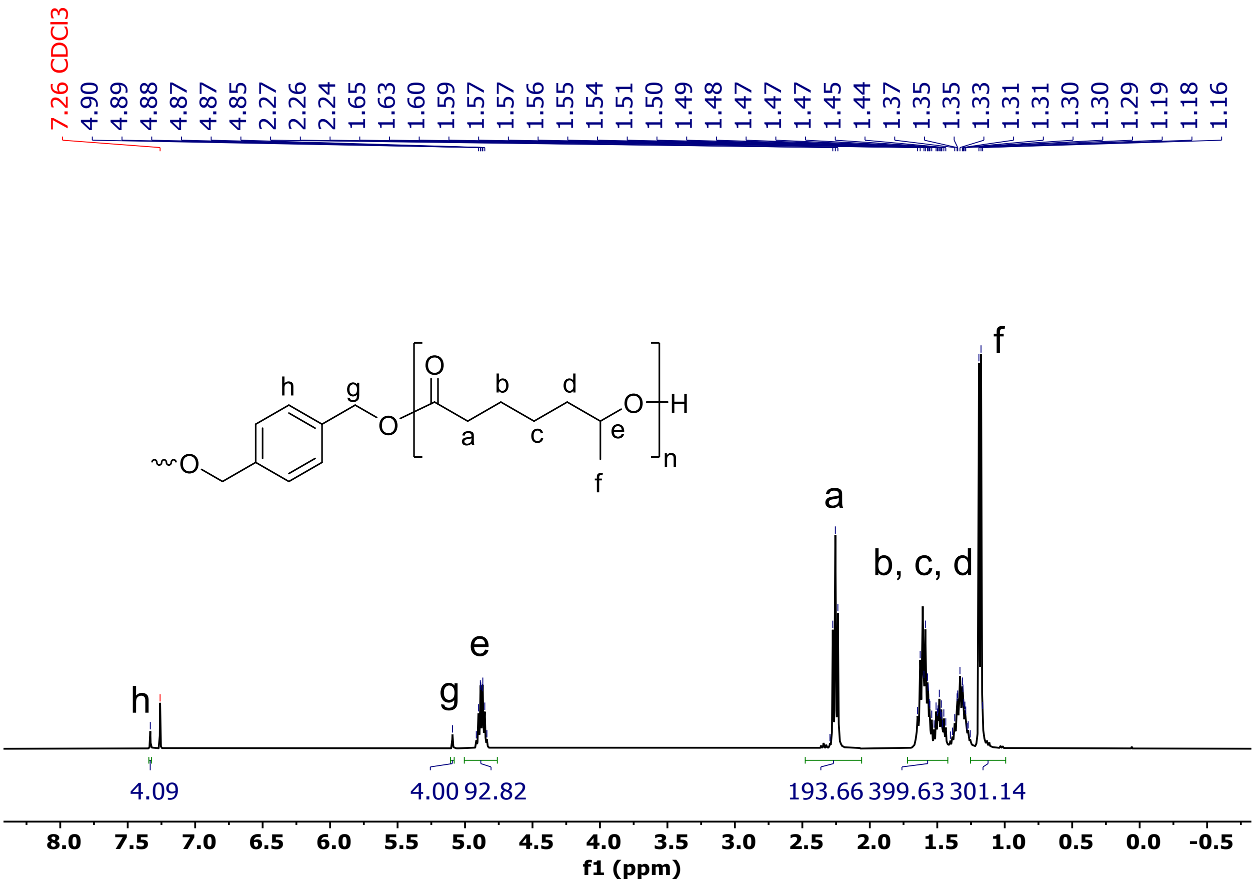

Figure S112. **^{1}H NMR spectrum (400 MHz, $CDCl_3$, 298K) of PE-7c. DP_{NMR} =93 determined from relative integration of H_g and H_e**

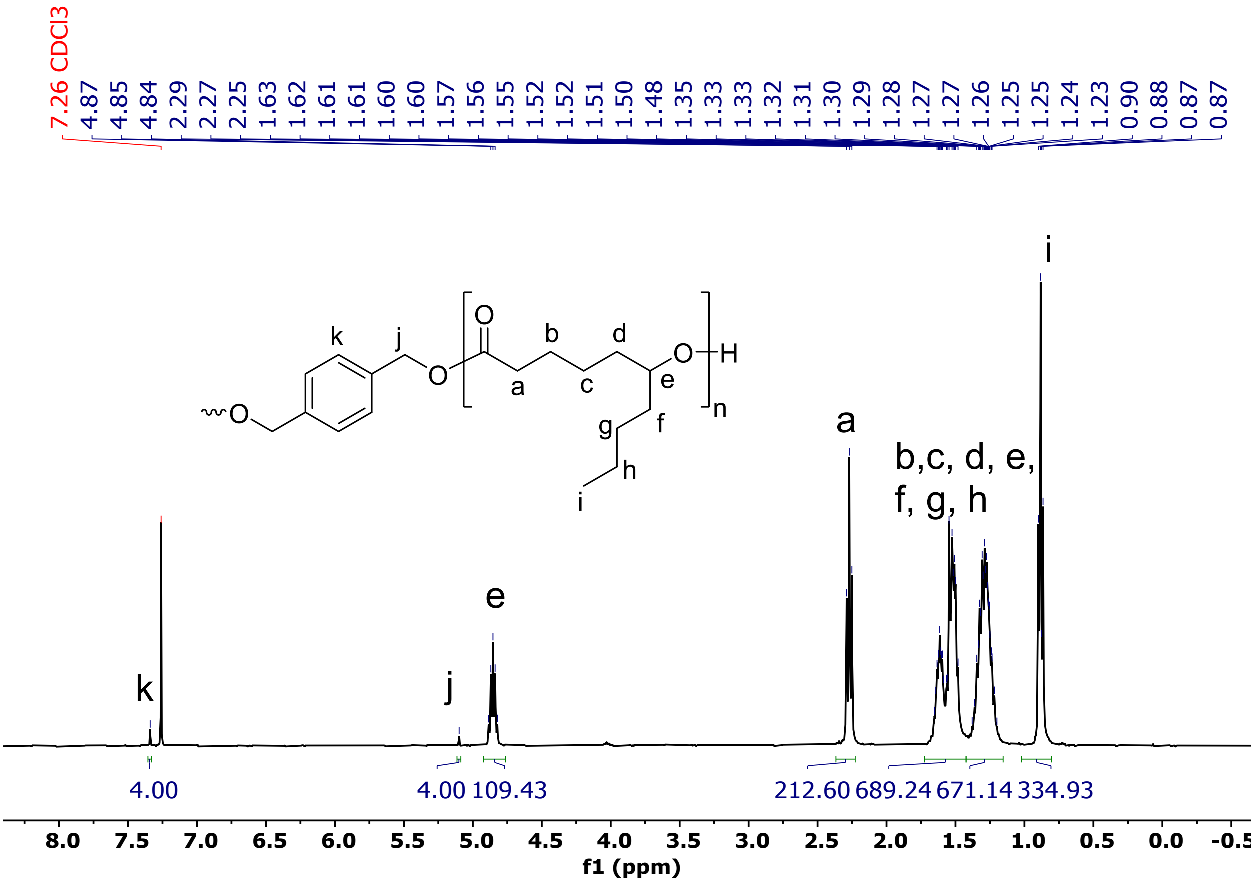

Figure S113. **^{1}H NMR spectrum (400 MHz, $CDCl_3$, 298K) of PE-7d. DP_{NMR} =109 determined from relative integration of H_j and H_e**

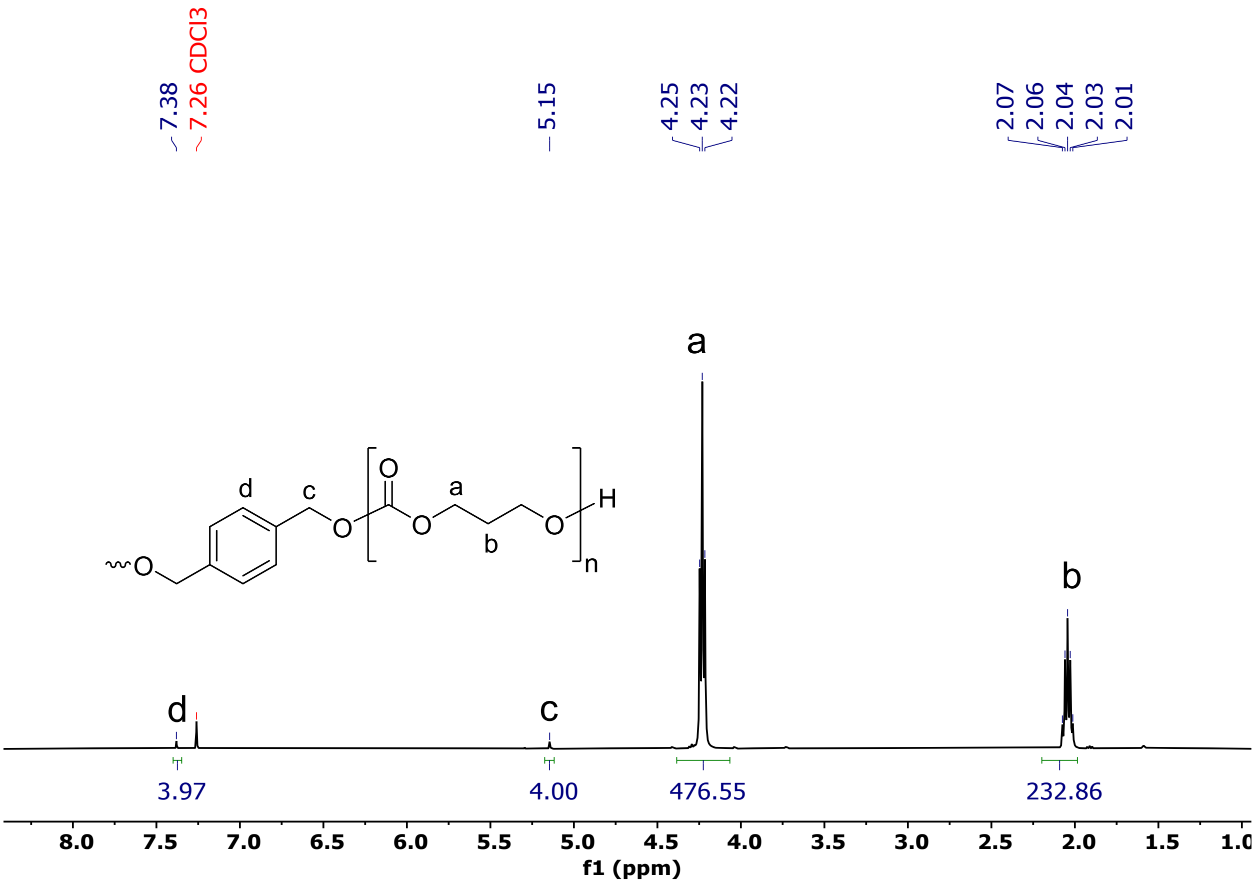

Figure S114. **^{1}H NMR spectrum (400 MHz, $CDCl_3$, 298K) of PC-6a.** ***DP_{NMR}*** **=116 determined from relative integration of** ***H_c*** **and** ***H_a***

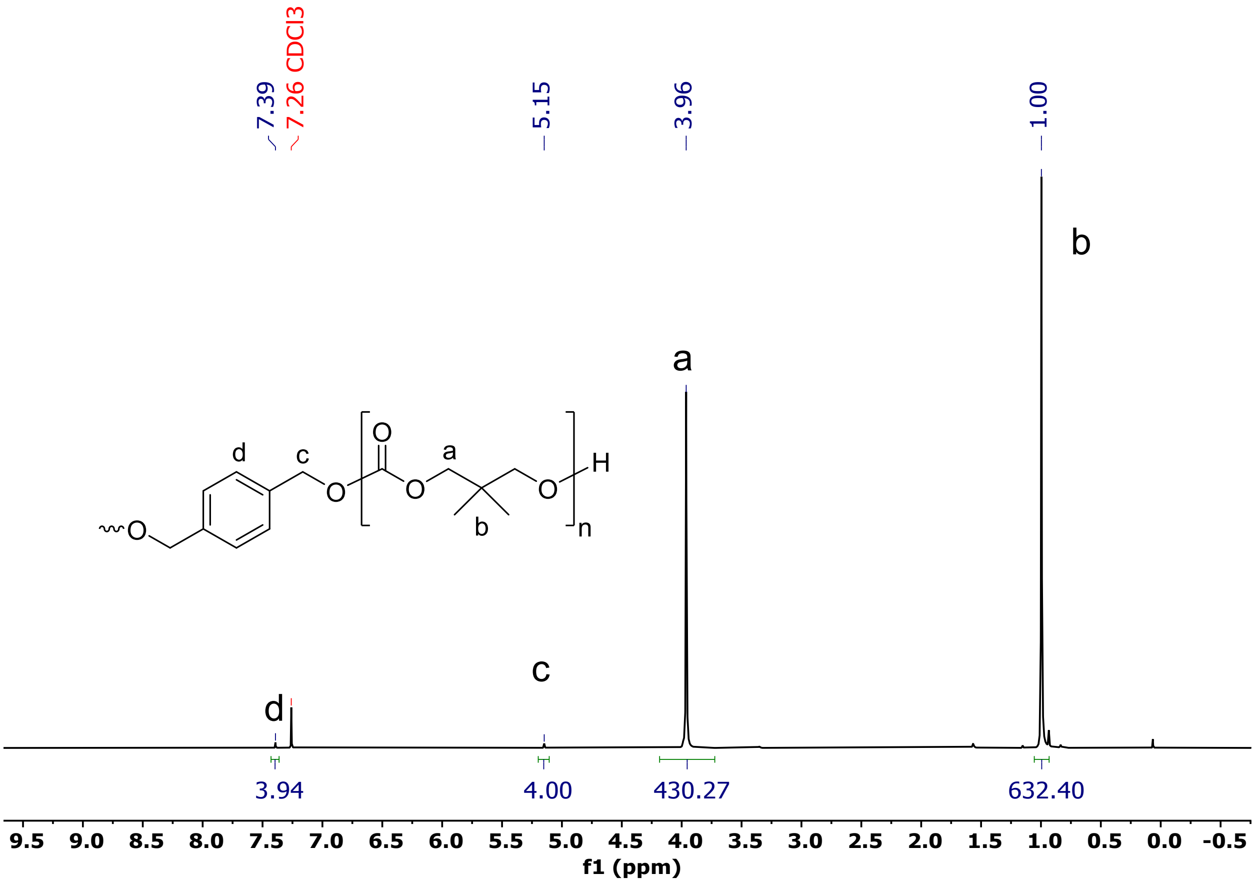

Figure S115. ^{1}H **NMR spectrum (400 MHz,** $CDCl_3$**, 298K) of PC-6b.** DP_{NMR} **=108 determined from relative integration of** H_c **and** H_a

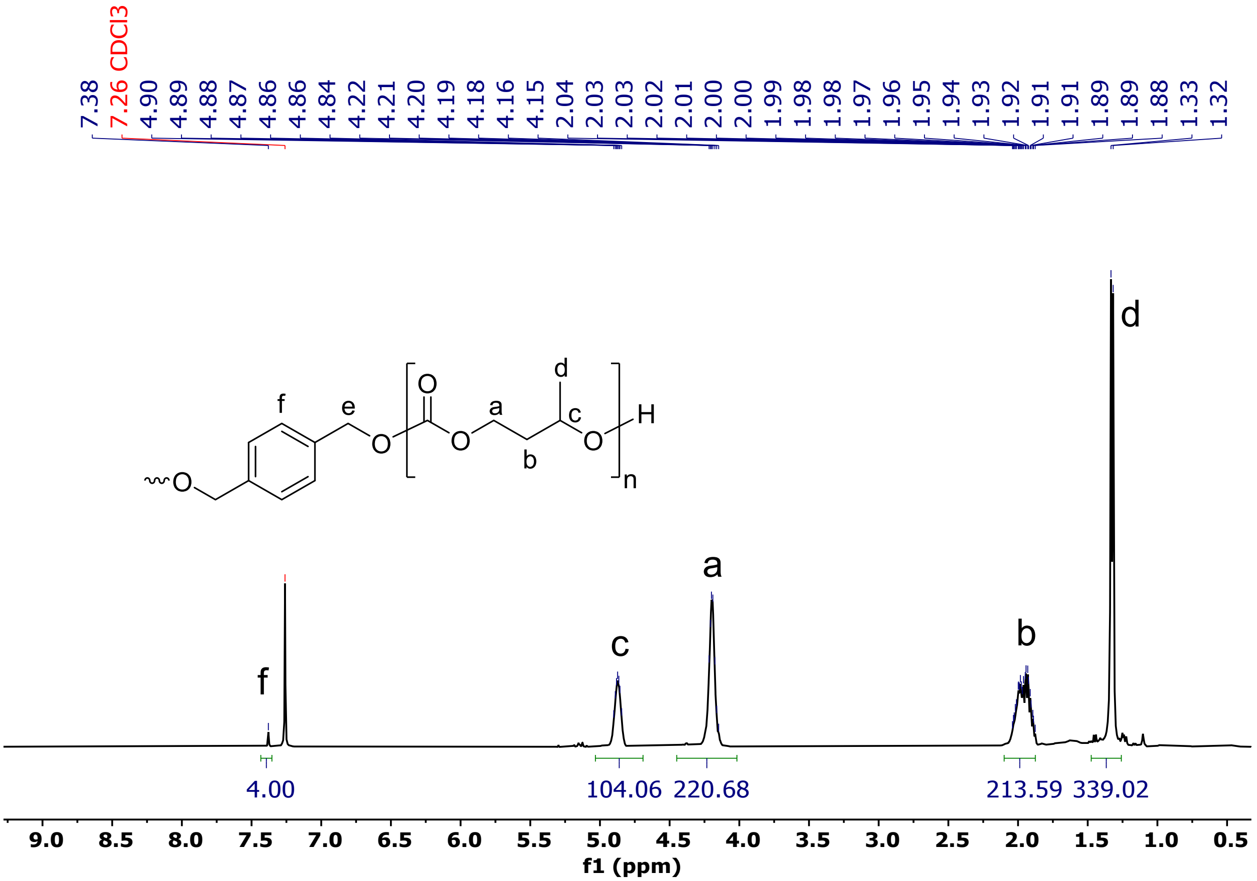

Figure S116. **^{1}H NMR spectrum (400 MHz, $CDCl_3$, 298K) of PC-6c. DP_{NMR} =105 determined by integration of H_f and H_c (N.B. H_e likely obscured by H_c)**

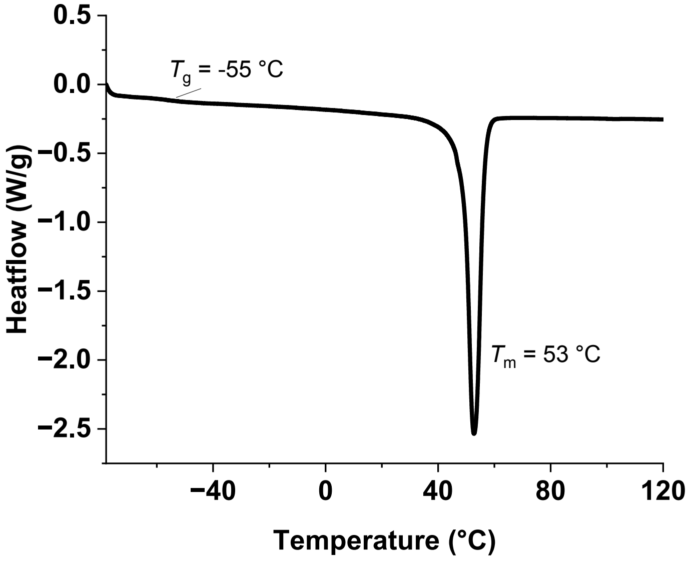

Figure S117. DSC thermogram of PE-6a, 2nd heating cycle, T_g = -55 °C, T_m = 53 °C

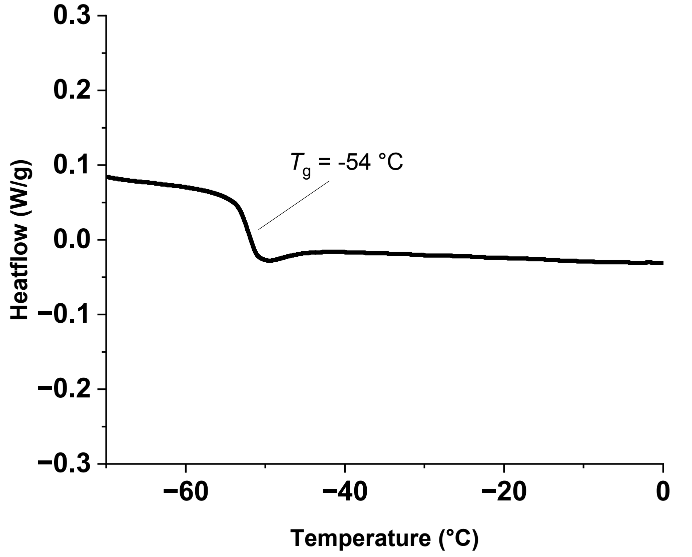

Figure S118. DSC thermogram of PE-6b, 2nd heating cycle, T_g = -54 °C, T_m = *not observed*

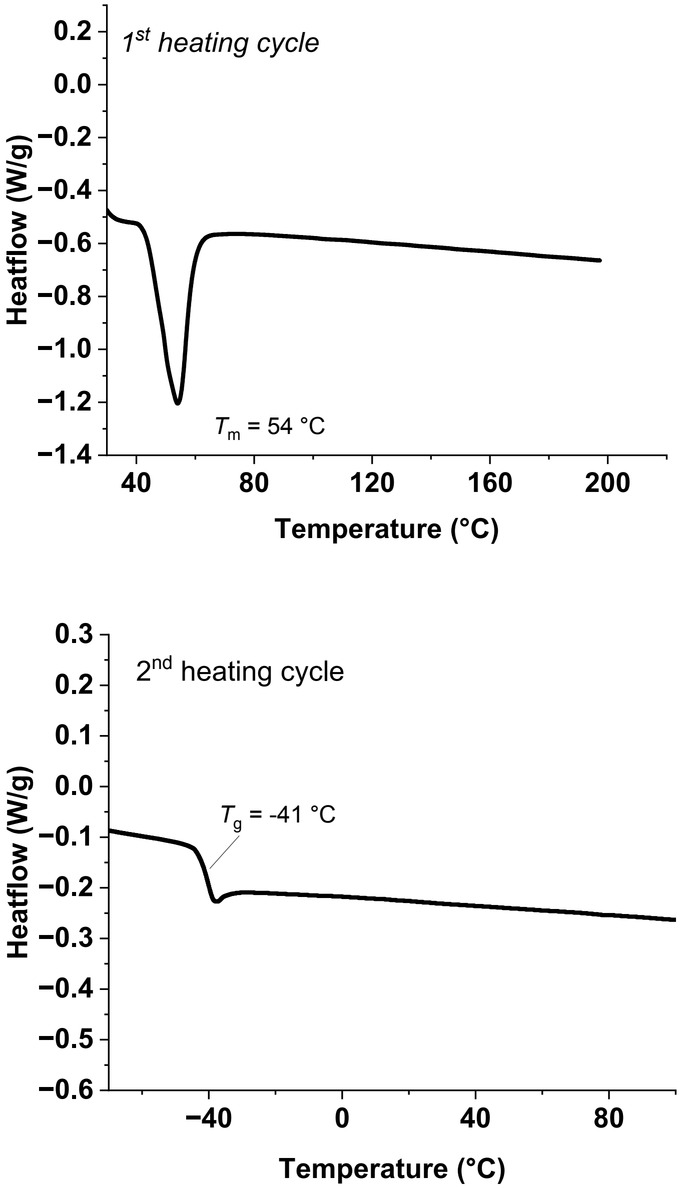

Figure S119. DSC thermograms of PE-6c, 1st + 2nd heating cycle, T_g = -41 °C, T_m = 54 °C (first cycle only)

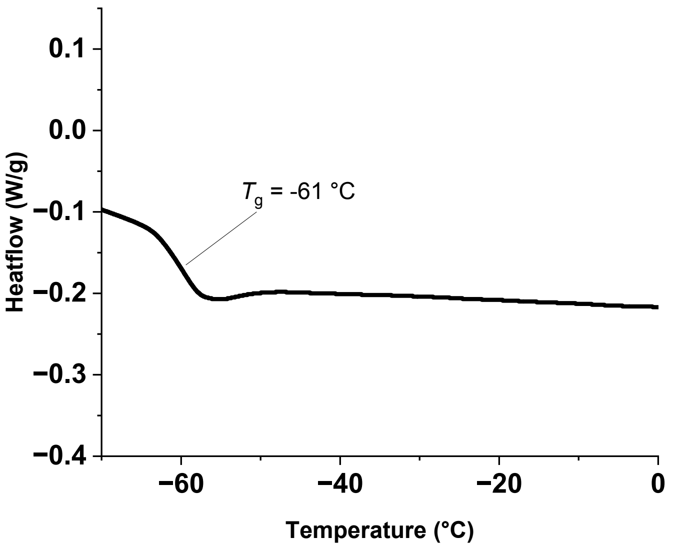

Figure S120. DSC thermogram of PE-6d, 2nd heating cycle, T_g = -61 °C, T_m = *not observed*

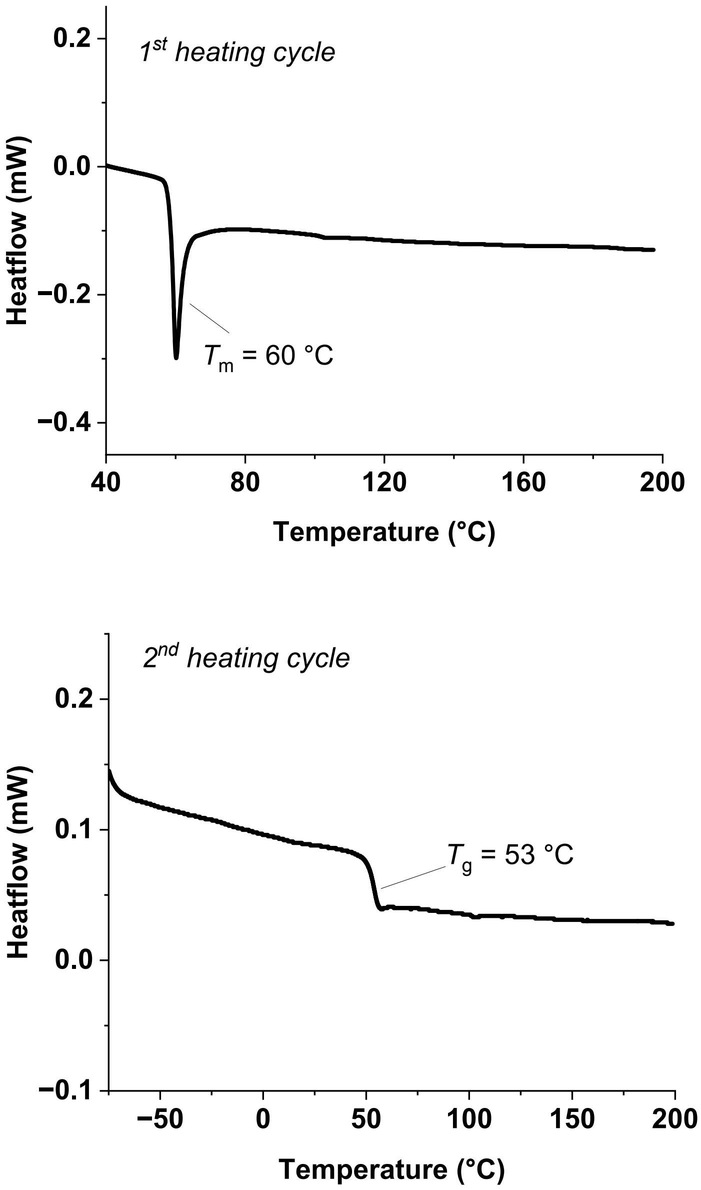

Figure S121. DSC thermogram of *rac*-PLA, 1st and 2nd heating cycle, T_g = 53 °C, T_m = 60 °C (first cycle only)

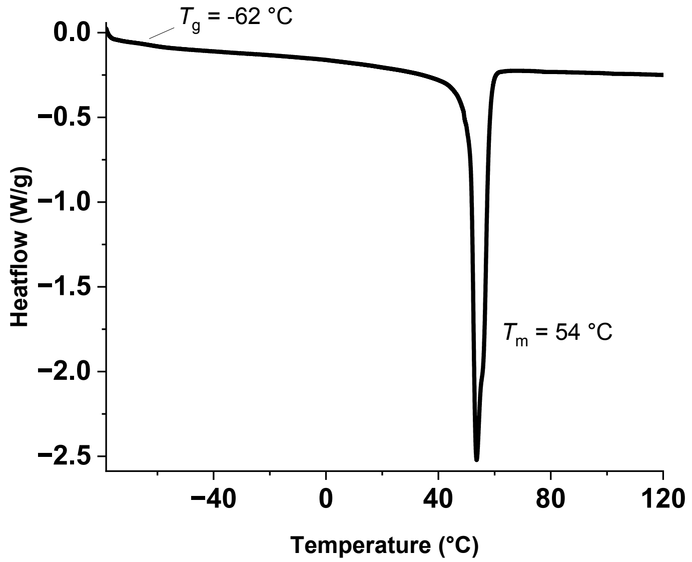

Figure S122. DSC thermogram of PE-7a, 2nd heating cycle, T_g = -61 °C, T_m = 53 °C

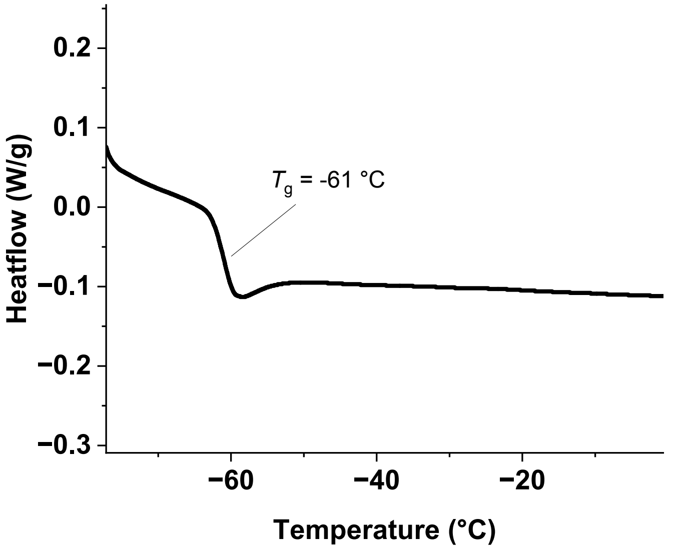

Figure S123. DSC thermogram of PE-7b, 2nd heating cycle, T_g = -61 °C, T_m =*not observed*

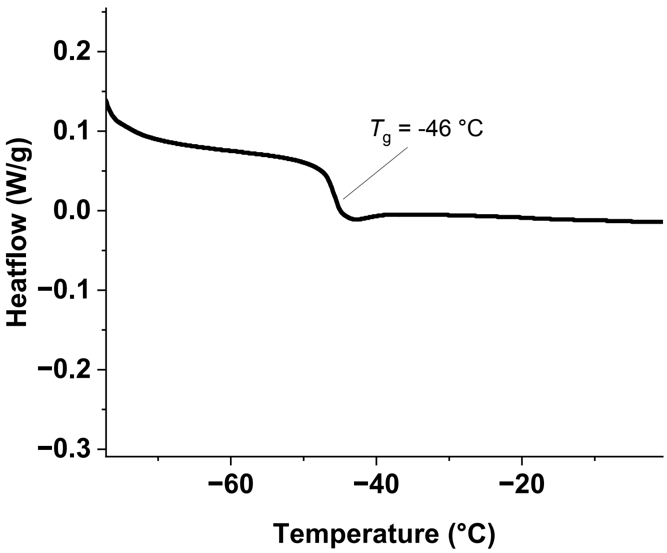

Figure S124. DSC thermogram of PE-7c, 2nd heating cycle, T_g = -46 °C, T_m = *not observed*

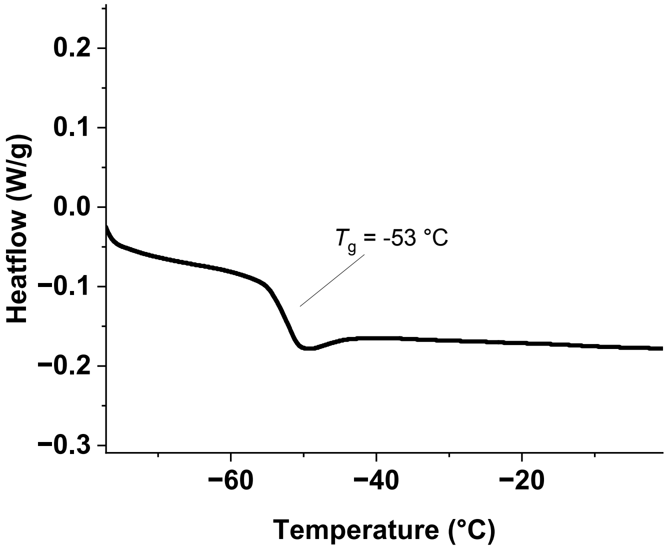

Figure S125. DSC thermogram of PE-7d, 2nd heating cycle, T_g = -53 °C, T_m = *not observed*

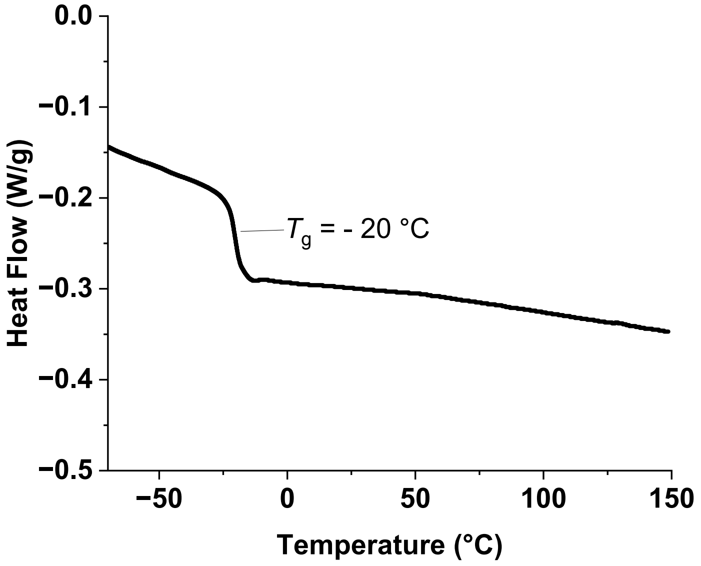

Figure S126. DSC thermogram of PC-6a, 2nd heating cycle, T_g = -20 °C, T_m = *not observed*

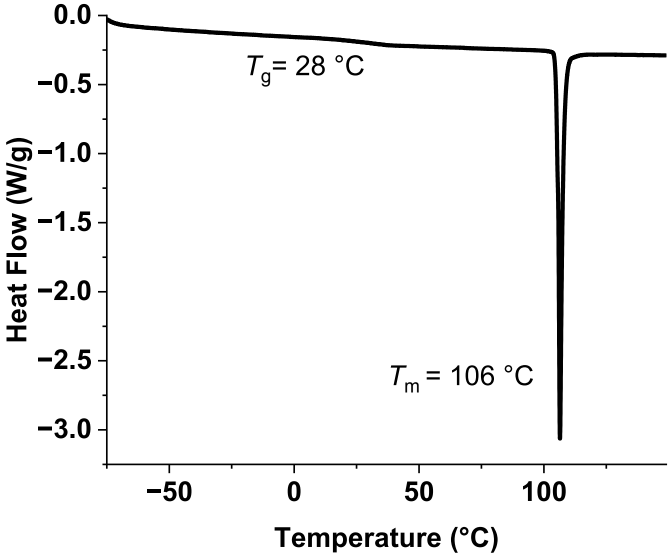

Figure S127. DSC thermogram of PC-6b, 2nd heating cycle, T_g = 28 °C, T_m = 106 °C

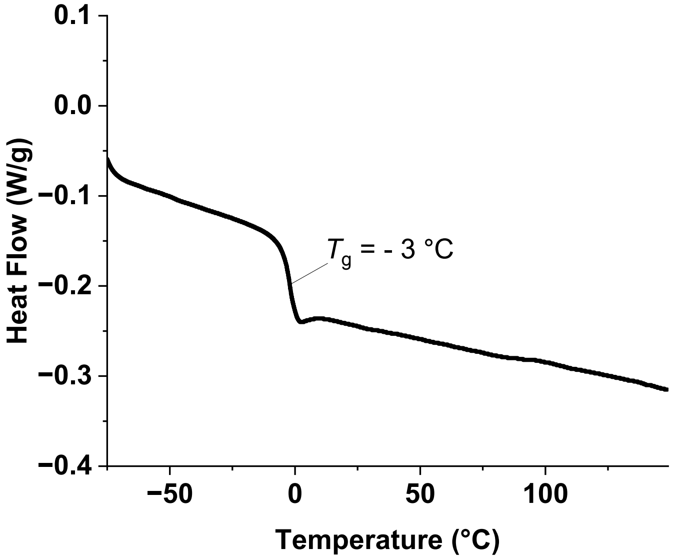

Figure S128. DSC thermogram of PC-6c, 2nd heating cycle, T_g = -3 °C, T_m = *not observed*

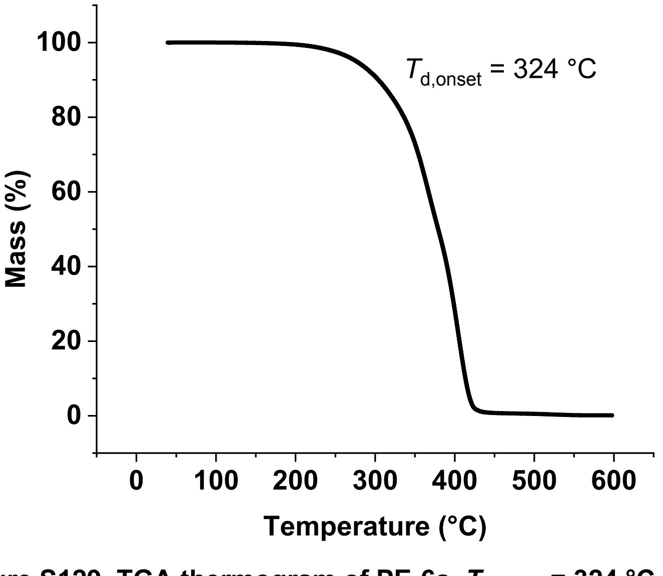

Figure S129. TGA thermogram of PE-6a, $T_{d,onset}$ = 324 °C

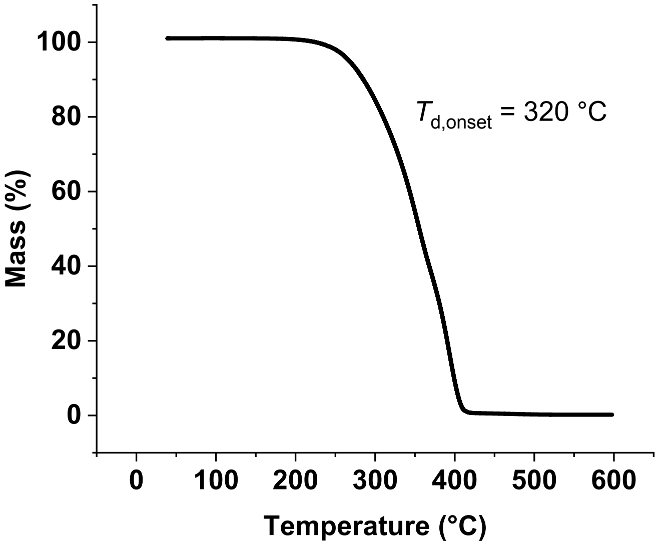

Figure S130. TGA thermogram of PE-6b, $T_{d,onset}$ = 320 °C

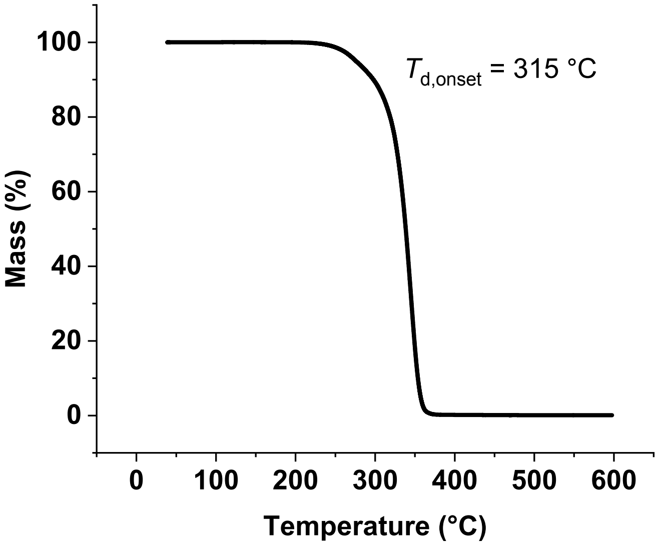

Figure S131. TGA thermogram of PE-6c, $T_{d,onset}$ = 315 °C

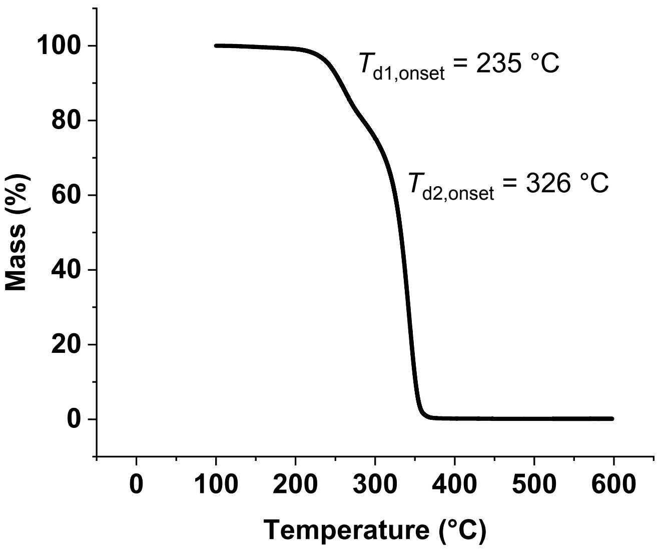

Figure S132. TGA thermogram of PE-6d, $T_{d,onset}$ = 326 °C

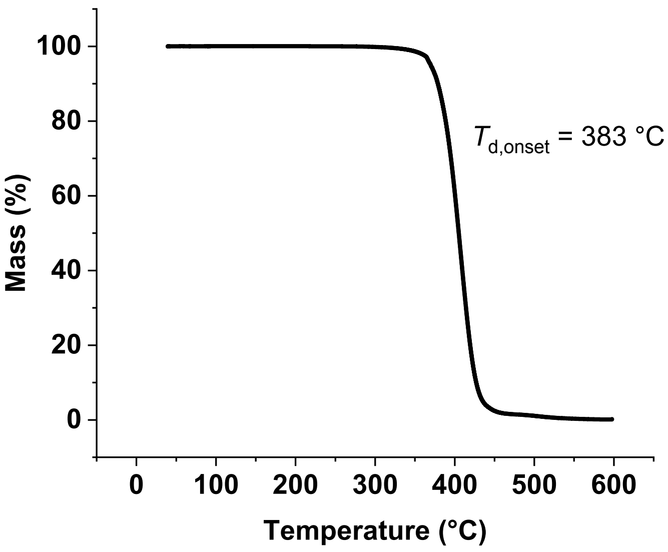

Figure S133. TGA thermogram of PE-7a, $T_{d,onset}$ = 383 °C

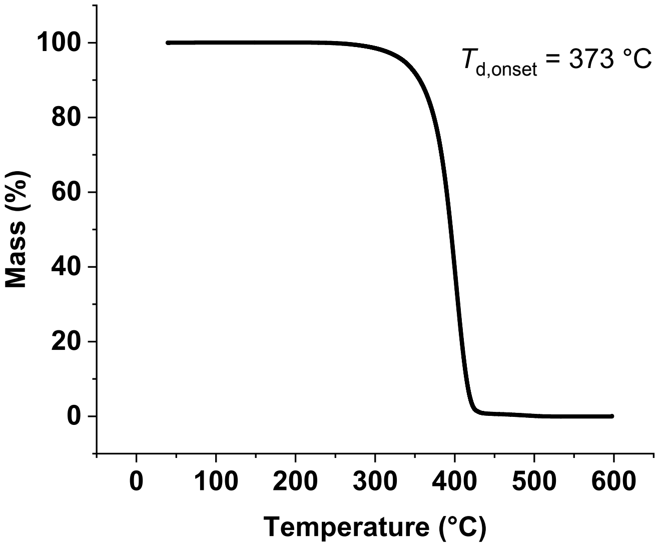

Figure S134. TGA thermogram of PE-7b, $T_{d,onset}$ = 373 °C

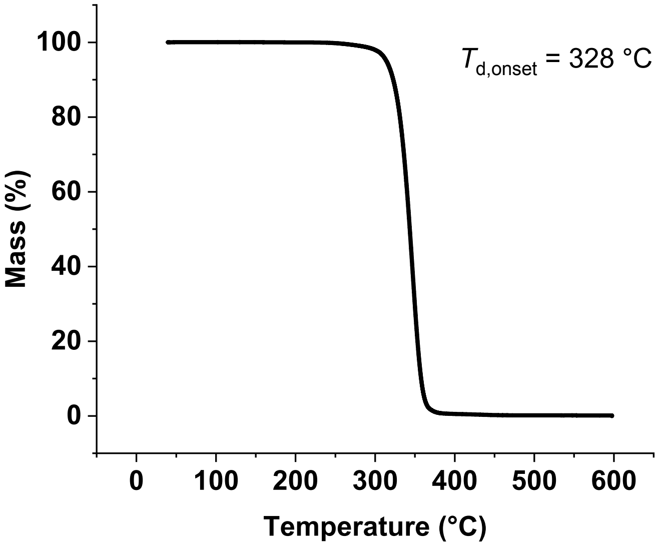

Figure S135. TGA thermogram of PE-7c, $T_{d,onset}$ = 328 °C

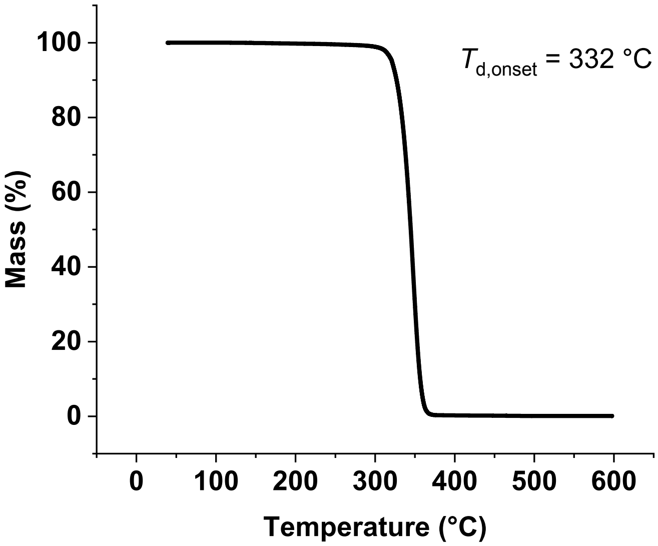

Figure S136. TGA thermogram of PE-7d, $T_{d,onset}$ = 332 °C

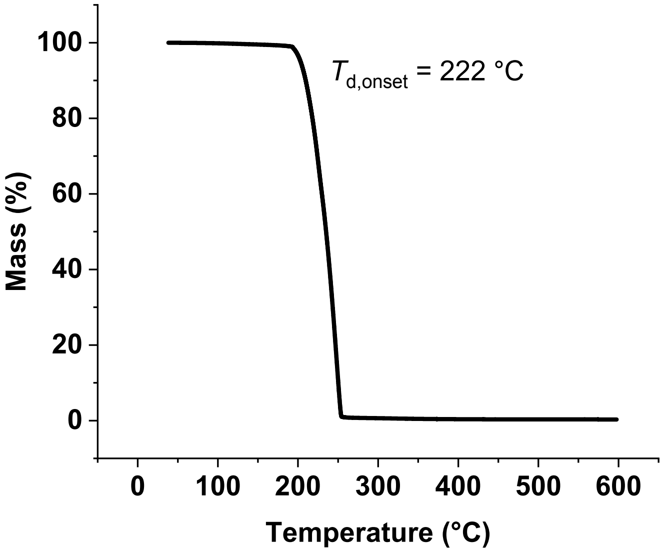

Figure S137. TGA thermogram of PC-6a, $T_{d,onset}$ = 222 °C

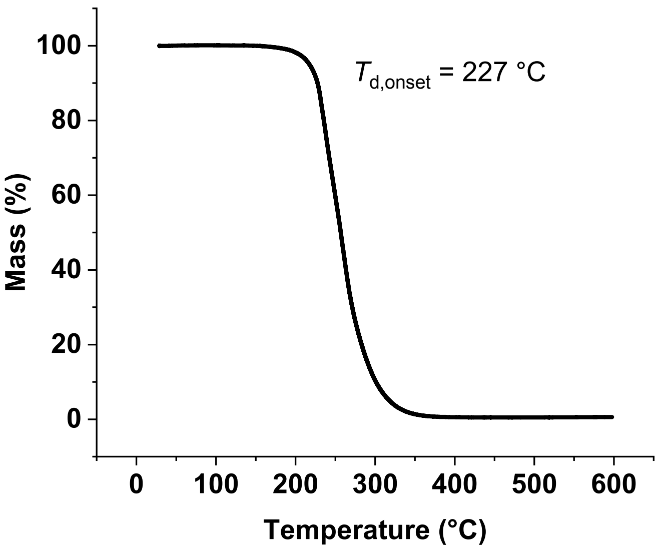

Figure S138. TGA thermogram of PC-6b. $T_{d,onset}$ = 227 °C

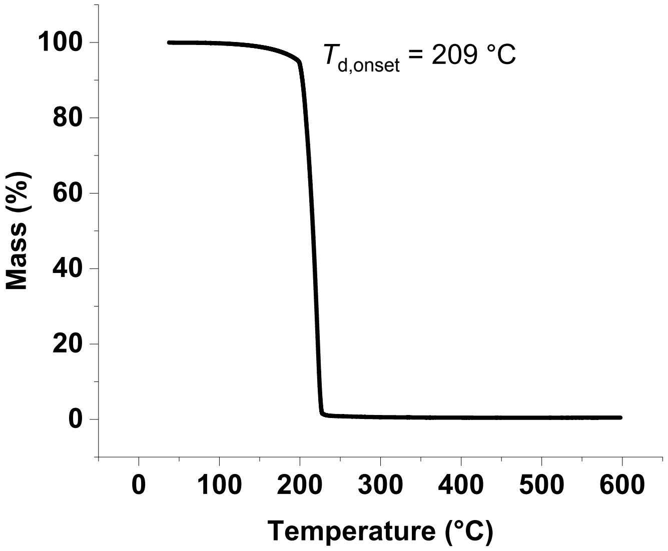

Figure S139. TGA thermogram of PC-6c. $T_{d,onset}$ = 209 °C

6. Monomer/polymer equilibrium measurements

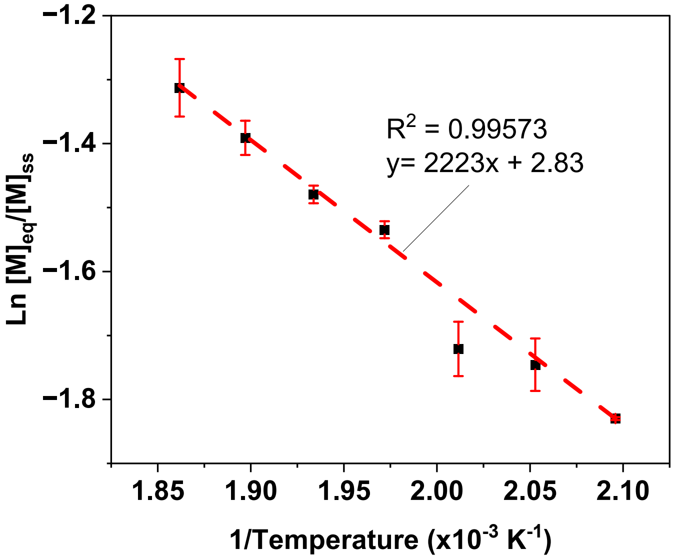

Figure S140. Plot of ln $[M]_{eq}/[M]_{ss}$ *vs* 1/T for the ROP of 7b. Reactions were performed at 200 – 250 °C, $[Sn(Oct)_2]_0$: $[BnOH]_0$: $[7b]_0$ = 1: 1: 2000, $[M]_{ss}$ = 1.0 M

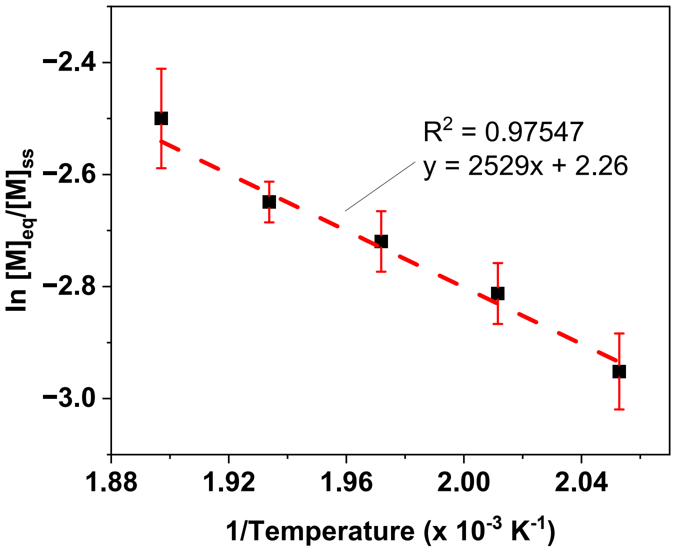

Figure S141. Plot of ln $[M]_{eq}/[M]_{ss}$ *vs* 1/T for the ROP of 7a. Reactions were performed at 210 – 250 °C, $[Sn(Oct)_2]_0$: $[BnOH]_0$: $[7a]_0$ = 1: 1: 500, $[M]_{ss}$ = 1.0 M. *N.B. monomer contains 5% α-methylcaprolactone*

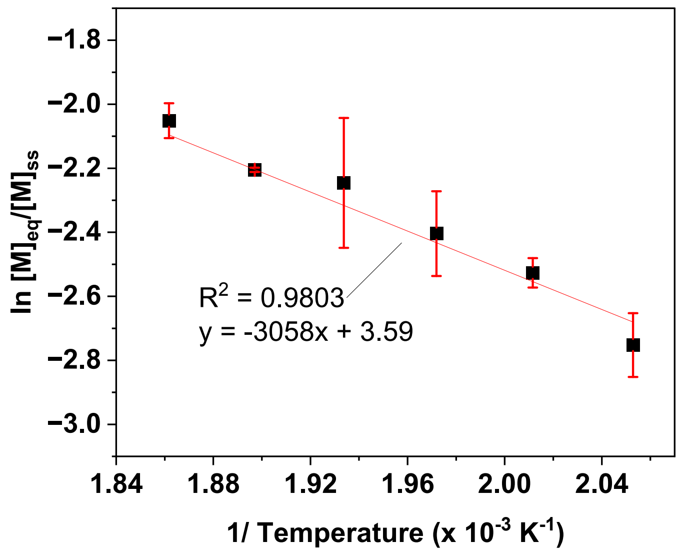

Figure S142. Plot of ln $[M]_{eq}/[M]_{ss}$ *vs* 1/T for the ROP of 7d. Reactions were performed at 200 – 250 °C in neat monomer, $[Sn(Oct)_2]_0$: $[BnOH]_0$: $[7d]_0$ = 1: 1: 100. Error bars are standard deviation of three repeats

7. References

(1) Schneiderman, D. K.; Hillmyer, M. A. Aliphatic polyester block polymer design. *Macromolecules* **2016**, *49* (7), 2419-2428. DOI: 10.1021/acs.macromol.6b00211.
(2) Watts, A.; Kurokawa, N.; Hillmyer, M. A. Strong, resilient, and sustainable aliphatic polyester thermoplastic elastomers. *Biomacromolecules* **2017**, *18* (6), 1845-1854.
(3) Martello, M. T.; Hillmyer, M. A. Polylactide–Poly(6-methyl-ε-caprolactone)–Polylactide Thermoplastic Elastomers. *Macromolecules* **2011**, *44* (21), 8537-8545. DOI: 10.1021/ma201063t.
(4) Olsson, J. V.; Hult, D.; Cai, Y.; García-Gallego, S.; Malkoch, M. Reactive imidazole intermediates: simplified synthetic approach to functional aliphatic cyclic carbonates. *Polym. Chem.* **2014**, *5* (23), 6651-6655. DOI: https://doi.org/10.1039/C4PY00911H.
(5) Yevstropov, A. A.; Lebedev, B. V.; Kulagina, T. G.; Lebedev, N. K. The calorimetric study in the 13·8–340°K range of δ-valerolactone, its polymer, and of the δ-valerolactone polymerization. *Polym. Sci. USSR* **1982**, *24* (3), 628-636. DOI: https://doi.org/10.1016/0032-3950(82)90053-3.
(6) Duda, A.; Kowalski, A.; Libiszowski, J.; Penczek, S. Thermodynamic and Kinetic Polymerizability of Cyclic Esters. *Macromol. Symp.* **2005**, *224* (1), 71-84. DOI: https://doi.org/10.1002/masy.200550607.
(7) McGuire, T. M.; Ning, D.; Williams, C. K. Using Differential Scanning Calorimetry to Accelerate Polymerization Catalysis: A Toolkit for Miniaturized and Automated Kinetics Measurements. *ACS Catal.* **2025**, *15* (9), 6760-6771. DOI: 10.1021/acscatal.5c01758.
(8) Duda, A.; Penczek, S. Thermodynamics of L-lactide polymerization. Equilibrium monomer concentration. *Macromolecules* **1990**, *23* (6), 1636-1639. DOI: 10.1021/ma00208a012.
(9) Olsén, P.; Odelius, K.; Albertsson, A.-C. Thermodynamic presynthetic considerations for ring-opening polymerization. *Biomacromolecules* **2016**, *17* (3), 699-709. DOI: 10.1021/acs.biomac.5b01698.

(10) Diment, W. T.; Gowda, R. R.; Chen, E. Y. X. Unraveling the Mechanism of Catalyzed Melt-Phase Polyester Depolymerization via Studies of Kinetics and Model Reactions. *J. Am. Chem. Soc.* **2024**, *146* (37), 25745-25754. DOI: 10.1021/jacs.4c08127.
(11) *Gaussian 16 Rev. C.01*; Wallingford, CT, 2016. (accessed.
(12) Zhao, Y.; Truhlar, D. G. A new local density functional for main-group thermochemistry, transition metal bonding, thermochemical kinetics, and noncovalent interactions. *J. Chem. Phys.* **2006**, *125* (19).
(13) Hehre, W. J.; Ditchfield, R.; Pople, J. A. Self—consistent molecular orbital methods. XII. Further extensions of Gaussian—type basis sets for use in molecular orbital studies of organic molecules. *J. Chem. Phys.* **1972**, *56* (5), 2257-2261.
(14) Marenich, A. V.; Cramer, C. J.; Truhlar, D. G. Universal solvation model based on solute electron density and on a continuum model of the solvent defined by the bulk dielectric constant and atomic surface tensions. *J. Phys. Chem. B* **2009**, *113* (18), 6378-6396.
(15) Luchini, G.; Alegre-Requena, J. V.; Funes-Ardoiz, I.; Paton, R. S. GoodVibes: automated thermochemistry for heterogeneous computational chemistry data. *F1000Research* **2020**, *9* (291), 291.
